# Supplementary material for: Evolving Patterns of Nutritional Deficiencies Burden in Low- and Middle-Income Countries: Findings from the 2019 Global Burden of Disease Study
Source: Nutrients. 2022 Feb 22;14(5):931. doi: 10.3390/nu14050931 (PMC8912291; doi:10.3390/nu14050931)
Supplement: Supplementary file 1 [file nutrients-14-00931-s001.zip › nutrients-1566662-supplementary.pdf]

Supplementary Online Content

The content of this document and the legends of eTables and eFigures are listed as bellows.

SECTION 1 ETABLES..58

SECTION 2 EFIGURES..63

ETABLE LEGENDS

Table S1: Sex-specific incidence and disability-adjusted life-years (DALYs) counts, crude rate, age standardized rate of overall nutritional deficiencies in global and low-and middle-income countries (LMICs) by calendar year, 1990-2019.

Table S2: Sex-specific incidence counts, crude rate, age standardized rate of main subcategories in global and low-and middle-income countries (LMICs) by calendar year, 1990-2019.

Table S3: Sex-specific disability-adjusted life-years (DALYs) counts, crude rate, age standardized rate of main subcategories in global and low-and middle-income countries (LMICs) by calendar year, 1990-2019.

Table S4: Age- and Sex-specific incidence rate and disability-adjusted life-years (DALYs) rate of overall nutritional deficiencies and high-risk subcategories in low-and middle-income countries (LMICs) by calendar year, 1990-2019.

Table S5: Sex-specific incidence counts, crude rate, age-standardized rate in 2019 and the estimated annual percentage change (EAPC) for age-standardized rate of nutritional deficiencies and its main subcategories from 1990 to 2019 at national level in low-and middle-income countries (LMICs).

Table S6: Sex-specific disability-adjusted life-years (DALYs) counts, crude rate, age-standardized rate in 2019 and the estimated annual percentage change (EAPC) for age-standardized rate of nutritional deficiencies and its main subcategories from 1990 to 2019 at national level in low-and middle-income countries (LMICs).

EFIGURES LEGENDS

Figure S1: The trend of age-sex specific incidence rates and DALYs rates for high-risk subcategories in low-and middle-income countries (LMICs) from 1990 to 2019.

Figure S2: Age-standardized incidence rate (ASIR) in 2019 and its estimated annual percentage change (EAPC) during 1990-2019 due to protein-energy malnutrition for all ages stratified by sex at national level in low-and middle-income countries (LMICs).

Figure S3: Age-standardized incidence rate (ASIR) and Age-standardized DALYs rate (ASDR) in 2019 and their estimated annual percentage change (EAPC) during 1990-2019 due to iodine deficiency stratified by sex at national level in low-and middle-income countries (LMICs).

Figure S4: Age-standardized incidence rate (ASIR) and Age-standardized DALYs rate (ASDR) in 2019 and their estimated annual percentage change (EAPC) during 1990-2019 due to vitamin A deficiency stratified by sex at national level in low-and middle-income countries (LMICs).

Figure S5: Age-standardized DALYs rate (ASDR) in 2019 and its estimated annual percentage change (EAPC) in 1990-2019 due to dietary iron deficiency stratified by sex at national level in low-and middle-income countries (LMICs).

Figure S6: The correlation between sex-specific age-standardized incidence rates (ASIR) and age-standardized DALYs rates (ASDR) for nutritional deficiencies (NDs) with Gender development index (GDI) in low-and middle-income countries (LMICs) in 2019.

Figure S7: The correlation between sex-specific age-standardized incidence rates (ASIR) and age-standardized DALYs rates (ASDR)for nutritional deficiencies with Gender development index (GDI) in 2019.

SECTION 1 ETABLES

Table S1. Sex-specific incidence and disability-adjusted life-years (DALYs) counts, crude rate, age standardized rate of overall nutritional deficiencies in global and low-and middle-income countries (LMICs) by calendar year, 1990-2019.

Year	Measure	Number (thousand)						Crude rate per100,000						Age standardized rate per 100,000					
		Global			LMICs			Global			LMICs			Global			LMICs		
		Both	Female	Male	Both	Female	Male	Both	Female	Male	Both	Female	Male	Both	Female	Male	Both	Female	Male
1990	Incidence	119100.9	56092.1	63008.8	68352.1	30973.1	37379.0	2226.2	2111.8	2339.1	1627.5	1493.4	1758.4	2025.7	1933.7	2119.9	1554.4	1421.9	1686.4
	DALYs	87847.4	46388.6	41458.8	60503.1	33230.0	27273.1	1642.1	1746.5	1539.1	1440.6	1602.2	1283.0	1496.9	1620.3	1381.0	1359.5	1530.1	1198.3
1991	Incidence	120168.9	56447.0	63721.9	69384.4	31221.5	38162.8	2211.9	2093.7	2328.4	1622.8	1479.3	1762.7	2030.7	1934.8	2128.7	1557.2	1415.8	1697.8
	DALYs	85773.7	45407.2	40366.5	59548.3	32758.6	26789.7	1578.8	1684.2	1475.0	1392.8	1552.2	1237.4	1451.5	1574.6	1335.8	1321.7	1489.9	1162.6
1992	Incidence	121207.9	56774.3	64433.6	70528.2	31547.3	38980.9	2198.0	2075.5	2318.6	1621.1	1469.6	1768.8	2037.6	1937.3	2139.7	1564.0	1414.5	1712.2
	DALYs	83628.4	44346.1	39282.3	58591.1	32257.7	26333.5	1516.5	1621.1	1413.5	1346.7	1502.7	1194.9	1407.5	1529.2	1292.9	1286.5	1452.0	1129.8
1993	Incidence	122268.2	57095.9	65172.3	71760.7	31937.3	39823.4	2185.3	2057.9	2310.7	1621.5	1462.7	1776.1	2045.8	1940.7	2152.7	1573.6	1416.8	1729.1
	DALYs	81651.9	43294.8	38357.1	57606.4	31691.3	25915.1	1459.4	1560.5	1359.9	1301.9	1452.0	1155.8	1367.9	1485.6	1257.0	1253.0	1413.3	1101.2
1994	Incidence	123387.2	57432.8	65954.3	73067.0	32384.2	40682.7	2174.2	2041.2	2304.9	1421.6	1132.4	1784.3	2054.8	1944.1	2167.0	1393.5	1113.1	1747.4
	DALYs	79906.0	42366.4	37539.6	56588.6	31101.7	25486.9	1408.0	1505.7	1311.9	1257.8	1401.7	1117.8	1332.5	1446.8	1224.9	1220.2	1375.2	1073.4
1995	Incidence	124544.5	57764.8	66779.7	74420.3	32873.9	41546.5	2164.0	2024.6	2301.1	1421.4	1126.6	1792.6	2060.8	1943.6	2179.3	1399.7	1111.5	1765.7
	DALYs	82173.1	43357.4	38815.6	58048.6	31725.9	26322.7	1427.8	1519.6	1337.5	1269.3	1406.6	1135.8	1370.6	1477.8	1268.9	1256.1	1403.4	1115.4
1996	Incidence	125829.3	58134.1	67695.2	76080.6	33561.8	42518.8	2156.1	2009.3	2300.4	1424.5	1124.0	1805.3	2069.1	1944.5	2194.8	1407.7	1111.2	1787.8
	DALYs	80529.9	42579.8	37950.0	56482.5	30980.3	25502.2	1379.9	1471.7	1289.6	1215.2	1351.3	1082.8	1331.7	1440.0	1228.9	1201.4	1349.2	1060.3
1997	Incidence	127256.1	58555.0	68701.2	78146.0	34509.6	43636.4	2150.8	1996.1	2302.9	1429.5	1122.6	1823.8	2082.5	1950.1	2215.8	1417.1	1111.3	1815.0
	DALYs	78676.9	41662.3	37014.6	55446.4	30430.1	25016.3	1329.7	1420.3	1240.7	1174.1	1306.1	1045.6	1294.8	1401.2	1193.6	1168.5	1312.5	1030.8
1998	Incidence	128726.3	59012.4	69714.0	80356.9	35561.2	44795.7	2146.7	1984.6	2306.0	1434.2	1120.5	1844.0	2095.5	1955.7	2236.0	1425.7	1110.5	1843.0
	DALYs	76621.8	40631.2	35990.6	54332.8	29817.1	24515.7	1277.8	1366.4	1190.5	1132.8	1259.7	1009.2	1255.0	1358.8	1155.9	1133.9	1272.8	1000.5
1999	Incidence	130190.8	59513.4	70677.4	82450.8	36555.9	45894.9	2142.7	1974.9	2307.9	1437.0	1117.2	1861.5	2107.7	1962.1	2254.0	1432.1	1108.9	1867.2
	DALYs	74627.3	39660.3	34967.0	53132.2	29150.6	23981.6	1228.2	1316.1	1141.8	1091.1	1212.6	972.7	1216.1	1318.3	1118.1	1092.2	1224.1	969.6
2000	Incidence	131561.9	60043.0	71518.9	84156.4	37329.7	46826.7	2137.3	1966.2	2305.8	1702.7	1529.2	1872.0	2114.8	1966.0	2264.2	1711.1	1538.6	1883.2
	DALYs	71339.2	38045.1	33294.0	52043.1	28572.4	23470.7	1159.0	1245.8	1073.4	1053.0	1170.5	938.3	1158.6	1257.6	1063.4	1069.2	1196.8	945.7
2001	Incidence	132754.8	60560.2	72194.6	85448.6	37903.6	47545.0	2129.0	1957.1	2298.3	1703.7	1529.4	1873.8	2117.0	1967.8	2266.8	1716.6	1544.3	1888.7
	DALYs	69322.0	37057.5	32264.5	50993.8	28024.4	22969.5	1111.7	1197.6	1027.1	1016.7	1130.8	905.3	1119.0	1216.1	1025.4	1036.6	1160.3	916.6

2002	Incidence	133870.8	61075.3	72795.6	86538.7	38416.3	48122.5	2119.4	1947.9	2288.4	1700.4	1527.0	1869.9	2117.4	1968.9	2266.2	1716.8	1546.3	1886.9
	DALYs	67348.9	36158.4	31190.4	49949.2	27540.1	22409.2	1066.2	1153.2	980.5	981.5	1094.7	870.8	1079.6	1177.0	985.6	1004.3	1126.5	885.4
2003	Incidence	135008.2	61612.9	73395.2	87536.1	38890.1	48646.0	2110.2	1939.6	2278.5	1695.4	1523.1	1864.0	2116.2	1969.0	2263.8	1714.7	1546.3	1882.7
	DALYs	62556.8	33902.3	28654.4	46938.9	26101.0	20837.9	977.8	1067.2	889.6	909.1	1022.2	798.5	992.4	1091.9	896.5	926.0	1048.4	807.4
2004	Incidence	136221.0	62176.4	74044.5	88564.2	39352.3	49212.0	2102.3	1932.0	2270.3	1691.1	1518.7	1859.9	2116.6	1970.2	2263.4	1713.1	1545.5	1880.3
	DALYs	60856.4	33120.4	27736.0	46059.2	25711.6	20347.6	939.2	1029.1	850.4	879.5	992.3	769.0	957.0	1056.3	861.2	897.3	1018.9	779.3
2005	Incidence	137610.1	62790.7	74819.4	89746.8	39826.5	49920.3	2097.1	1926.1	2266.0	1689.8	1514.9	1861.3	2118.7	1971.8	2266.1	1714.8	1545.1	1884.0
	DALYs	59589.2	32545.3	27043.8	45499.4	25483.0	20016.3	908.1	998.3	819.0	856.7	969.3	746.3	928.8	1027.7	833.2	875.4	996.2	758.0
2006	Incidence	139383.1	63541.5	75841.6	91246.8	40361.0	50885.8	2097.2	1924.0	2268.4	1694.1	1513.1	1871.6	2124.5	1975.5	2274.2	1722.4	1546.9	1897.6
	DALYs	58543.8	32095.4	26448.4	45042.7	25308.9	19733.8	880.9	971.8	791.1	836.3	948.8	725.8	903.9	1003.0	808.0	855.8	976.0	738.6
2007	Incidence	141445.6	64389.2	77056.3	92996.9	40942.1	52054.9	2100.8	1924.1	2275.4	1702.2	1512.6	1888.4	2133.8	1981.3	2287.1	1734.1	1549.9	1918.1
	DALYs	57422.3	31588.4	25833.8	44497.7	25068.0	19429.7	852.9	944.0	762.8	814.5	926.1	704.8	877.4	976.1	781.8	834.3	953.1	718.4
2008	Incidence	143440.4	65164.9	78275.5	94755.8	41489.6	53266.2	2103.4	1922.3	2282.5	1710.5	1511.0	1906.6	2143.3	1986.2	2301.3	1746.0	1551.5	1940.1
	DALYs	56479.0	31192.5	25286.4	44069.8	24904.3	19165.5	828.2	920.2	737.3	795.5	907.0	686.0	854.1	953.2	758.1	815.4	933.6	700.1
2009	Incidence	145021.5	65709.3	79312.2	96297.3	41931.1	54366.2	2100.7	1914.2	2285.1	1715.2	1505.9	1921.1	2148.4	1985.9	2312.0	1753.8	1549.5	1958.0
	DALYs	55483.8	30744.4	24739.3	43600.5	24699.4	18901.1	803.7	895.6	712.8	776.6	887.0	667.9	830.9	929.5	735.3	796.4	913.2	682.2
2010	Incidence	145900.1	65896.7	80003.4	97416.1	42205.9	55210.1	2088.1	1896.0	2278.2	1712.0	1494.8	1925.9	2143.5	1975.2	2312.9	1753.5	1541.1	1965.9
	DALYs	55428.4	30726.6	24701.8	43512.5	24675.9	18836.6	793.3	884.1	703.4	764.7	873.9	657.1	822.7	920.0	728.2	784.9	900.3	672.1
2011	Incidence	145334.8	65298.8	80036.0	97517.5	41921.5	55596.0	2055.1	1855.7	2252.5	1690.9	1464.2	1914.5	2115.0	1938.9	2292.3	1735.5	1513.3	1957.8
	DALYs	54796.1	30467.9	24328.2	43296.4	24608.1	18688.3	774.8	865.9	684.7	750.8	859.5	643.6	805.8	903.1	711.3	771.2	885.8	659.0
2012	Incidence	143292.7	63874.1	79418.6	96571.4	41000.1	55571.3	2002.1	1793.1	2209.1	1652.4	1412.4	1889.2	2064.7	1878.4	2252.2	1700.2	1464.3	1936.4
	DALYs	53453.4	29881.0	23572.4	42790.2	24397.7	18392.5	746.8	838.9	655.7	732.2	840.5	625.3	778.0	875.7	683.0	752.6	866.3	641.0
2013	Incidence	140554.0	62096.0	78458.1	95221.7	39860.8	55360.9	1940.7	1722.3	2157.3	1607.9	1354.5	1858.1	2008.3	1811.8	2206.0	1658.4	1408.6	1908.8
	DALYs	52827.3	29643.4	23183.9	42536.9	24339.3	18197.6	729.4	822.2	637.5	718.3	827.1	610.8	761.6	859.7	666.1	738.8	852.7	627.0
2014	Incidence	138139.2	60550.6	77588.6	94125.2	38932.9	55192.3	1885.5	1659.8	2109.5	1568.8	1305.3	1829.2	1960.5	1756.2	2166.2	1621.5	1361.1	1882.7
	DALYs	52196.5	29414.9	22781.6	42331.7	24314.3	18017.4	712.5	806.3	619.4	705.5	815.2	597.1	745.9	844.9	649.5	726.2	840.6	613.7
2015	Incidence	137319.9	59929.9	77390.0	93949.8	38657.6	55292.2	1853.2	1623.8	2080.8	1546.0	1279.2	1810.0	1929.6	1721.3	2139.3	1600.6	1336.5	1865.6
	DALYs	51668.4	29215.8	22452.6	42200.6	24313.8	17886.8	697.3	791.6	603.7	694.4	804.5	585.5	731.4	830.5	634.7	715.4	829.9	602.7
2016	Incidence	138178.9	60893.2	77285.8	95176.0	40077.0	55099.0	1843.7	1631.0	2054.8	1546.5	1309.0	1781.6	1919.9	1726.7	2114.3	1601.2	1365.8	1838.1
	DALYs	51253.9	29110.2	22143.8	42204.3	24428.2	17776.0	683.9	779.7	588.7	685.8	797.9	574.8	717.9	818.2	620.0	706.9	823.2	592.3
2017	Incidence	140482.2	62969.0	77513.2	97907.4	42615.3	55292.1	1853.8	1667.8	2038.5	1570.8	1374.0	1765.8	1939.8	1771.6	2109.2	1624.8	1429.3	1822.2
	DALYs	50840.2	29016.1	21824.0	42189.0	24541.9	17647.1	670.9	768.5	574.0	676.9	791.3	563.6	705.7	807.5	606.3	697.7	816.1	581.1
2018	Incidence	148190.5	66941.6	81248.9	104821.2	46210.6	58610.6	1935.0	1754.0	2114.7	1661.1	1471.2	1849.3	2034.0	1871.8	2197.6	1716.3	1529.0	1905.6

	DALYs	50179.8	28689.4	21490.3	41921.0	24416.7	17504.2	655.2	751.7	559.3	664.3	777.3	552.3	691.3	791.4	593.3	684.5	801.1	569.6
2019	Incidence	162197.5	73336.5	88861.0	116480.4	51405.6	65074.9	2096.3	1901.6	2289.7	1824.1	1616.8	2029.6	2207.7	2034.4	2382.1	1883.2	1681.5	2086.6
	DALYs	49775.1	28456.6	21318.6	41777.8	24321.3	17456.5	643.3	737.9	549.3	654.2	765.0	544.5	680.1	777.9	584.4	674.4	788.3	562.1

Table S2. Sex-specific incidence counts, crude rate, age standardized rate of main subcategories in global and low-and middle-income countries (LMICs) by calendar year, 1990-2019.

Year	Main subcategories	Number (thousand)						Crude rate per100,000						Age standardized Incidence rate per 100,000					
		Global			LMICs			Global			LMICs			Global			LMICs		
		Both	Female	Male	Both	Female	Male	Both	Female	Male	Both	Female	Male	Both	Female	Male	Both	Female	Male
1990	Protein-energy malnutrition	111389.2	51625.2	59764.0	60980.0	26711.1	34268.9	2082.1	1943.6	2218.6	1452.0	1287.9	1612.1	1896.6	1781.6	2013.2	1409.1	1250.4	1566.6
	Iodine deficiency	7711.7	4466.9	3244.8	7372.0	4262.0	3110.1	144.1	168.2	120.5	175.5	205.5	146.3	129.1	152.1	106.7	145.3	171.5	119.7
	Vitamin A deficiency	877376.3	336074.1	541302.2	812672.3	306373.8	506298.6	16400.0	12652.9	20094.8	19350.6	14772.3	23817.5	17323.2	13456.5	21073.8	18069.0	13760.7	22282.0
1991	Protein-energy malnutrition	112482.0	51937.9	60544.1	62035.1	26916.6	35118.5	2070.4	1926.4	2212.3	1450.9	1275.4	1622.1	1903.4	1782.8	2025.2	1413.6	1244.3	1581.3
	Iodine deficiency	7686.9	4509.1	3177.8	7349.3	4304.9	3044.3	141.5	167.2	116.1	171.9	204.0	140.6	127.3	151.9	103.5	143.6	171.5	116.5
	Vitamin A deficiency	877061.3	337469.5	539591.8	813258.1	308217.8	505040.3	16144.0	12517.2	19716.9	19021.4	14604.1	23327.5	17063.9	13325.5	20686.7	17796.6	13634.0	21863.8
1992	Protein-energy malnutrition	113557.2	52233.8	61323.4	63212.7	27209.8	36002.9	2059.2	1909.5	2206.7	1453.0	1267.5	1633.6	1912.1	1785.9	2039.4	1422.4	1243.5	1599.0
	Iodine deficiency	7650.7	4540.5	3110.2	7315.5	4337.4	2978.1	138.7	166.0	111.9	168.2	202.0	135.1	125.4	151.4	100.3	141.7	171.1	113.2
	Vitamin A deficiency	879156.4	339260.2	539896.2	816224.3	310471.4	505752.9	15942.6	12402.2	19427.6	18761.3	14462.7	22948.5	16863.3	13217.2	20393.4	17592.0	13535.0	21553.1
1993	Protein-energy malnutrition	114657.5	52532.7	62124.9	64482.5	27575.8	36906.7	2049.3	1893.4	2202.6	1322.9	1047.7	1646.1	1922.3	1790.1	2055.3	1290.0	1018.2	1619.0
	Iodine deficiency	7610.7	4563.2	3047.4	7278.2	4361.5	2916.7	136.0	164.5	108.0	53.3	38.2	130.1	123.5	150.6	97.3	39.7	28.1	110.1
	Vitamin A deficiency	856884.7	332880.5	524004.2	796014.7	305011.7	491003.1	15315.3	11998.0	18578.4	18807.0	15324.0	21899.0	16212.7	12798.7	19515.7	17608.7	14282.1	20596.2
1994	Protein-energy malnutrition	115810.8	52852.5	62958.3	65820.2	28004.0	37816.2	2040.7	1878.4	2200.2	1323.2	1039.4	1658.6	1933.1	1794.5	2072.3	1297.6	1016.7	1640.1
	Iodine deficiency	7576.4	4580.4	2996.0	7246.8	4380.2	2866.5	133.5	162.8	104.7	51.7	37.3	125.7	121.7	149.6	94.7	38.7	27.5	107.3
	Vitamin A deficiency	837846.2	327432.1	510414.1	778957.3	300472.1	478485.2	14763.4	11637.2	17837.4	18140.4	14919.0	20986.1	15643.8	12427.1	18754.5	17008.7	13925.1	19769.4
1995	Protein-energy malnutrition	116989.0	53171.4	63817.6	67191.5	28478.8	38712.8	2032.7	1863.6	2199.0	1324.2	1033.2	1670.4	1940.7	1795.2	2086.6	1305.1	1015.8	1660.6
	Iodine deficiency	7555.5	4593.4	2962.0	7228.8	4395.1	2833.7	131.3	161.0	102.1	50.8	36.9	122.3	120.1	148.5	92.7	38.0	27.2	105.1
	Vitamin A deficiency	826792.4	325185.8	501606.6	769241.0	298819.1	470422.0	14365.7	11397.2	17284.2	17582.9	14524.7	20297.7	15232.3	12180.7	18182.4	16519.1	13580.1	19169.1
1996	Protein-energy malnutrition	118325.3	53553.3	64772.0	68900.3	29177.4	39722.9	2027.5	1851.0	2201.0	1328.2	1030.3	1686.6	1951.1	1798.0	2104.2	1314.7	1016.5	1685.2
	Iodine deficiency	7504.0	4580.8	2923.2	7180.3	4384.4	2795.9	128.6	158.3	99.3	50.3	36.8	118.7	118.0	146.5	90.6	37.7	27.2	102.6
	Vitamin A deficiency	814352.9	321389.6	492963.3	758149.8	295619.5	462530.4	13953.9	11108.4	16751.4	17050.3	14135.6	19638.3	14806.5	11883.9	17631.4	16047.3	13229.1	18596.6
1997	Protein-energy malnutrition	119857.5	54020.3	65837.1	71067.7	30169.1	40898.6	2025.7	1841.5	2206.9	1334.6	1028.9	1709.4	1967.3	1806.5	2128.0	1326.2	1018.4	1715.6
	Iodine deficiency	7398.7	4534.6	2864.0	7078.3	4340.5	2737.8	125.0	154.6	96.0	49.7	36.6	114.4	115.2	143.6	87.9	37.4	27.2	99.4

	Vitamin A deficiency	797524.1	316807.9	480716.2	742784.2	291622.0	451162.2	13479.2	10799.9	16113.7	16440.3	13720.4	18856.6	14316.2	11567.7	16973.0	15499.9	12852.4	17900.4
1998	Protein-energy malnutrition	121446.3	54534.4	66911.9	73394.0	31275.2	42118.8	2025.3	1834.0	2213.4	1340.6	1026.9	1733.8	1983.2	1815.3	2150.9	1336.9	1019.7	1746.8
	Iodine deficiency	7280.0	4477.9	2802.1	6962.9	4286.0	2676.9	121.4	150.6	92.7	49.1	36.5	110.2	112.2	140.4	85.1	37.1	27.1	96.2
	Vitamin A deficiency	788453.9	315306.1	473147.8	734755.4	290486.0	444269.4	13148.4	10603.9	15651.1	15968.1	13373.5	18288.0	13979.8	11374.3	16498.8	15084.0	12540.3	17406.3
1999	Protein-energy malnutrition	122996.5	55076.2	67920.3	75674.5	32412.5	43262.1	2024.3	1827.6	2217.9	1416.0	1125.9	1754.7	1997.9	1824.3	2171.1	1425.6	1147.4	1773.7
	Iodine deficiency	7194.3	4437.2	2757.1	6880.3	4247.5	2632.8	118.4	147.2	90.0	141.3	176.7	106.8	109.9	137.8	82.9	123.4	154.3	93.5
	Vitamin A deficiency	792337.2	319385.3	472951.9	738621.1	294328.1	444293.0	13040.6	10598.1	15443.9	15168.3	12243.2	18020.4	13883.5	11392.6	16292.5	14507.2	11720.4	17201.9
2000	Protein-energy malnutrition	124369.7	55601.7	68768.0	77275.2	33075.9	44199.4	2020.5	1820.8	2217.1	1563.5	1355.0	1767.0	2006.1	1829.4	2182.3	1588.8	1385.3	1791.0
	Iodine deficiency	7192.3	4441.4	2750.9	6881.1	4253.8	2627.3	116.8	145.4	88.7	139.2	174.3	105.0	108.8	136.7	82.0	122.3	153.4	92.3
	Vitamin A deficiency	785909.7	318799.4	467110.4	732946.7	293847.1	439099.6	12767.8	10439.6	15060.1	14829.6	12037.7	17554.2	13608.3	11238.8	15900.8	14210.2	11556.6	16806.5
2001	Protein-energy malnutrition	125455.5	56054.3	69401.2	78456.4	33582.4	44874.0	2011.9	1811.5	2209.4	1564.3	1355.1	1768.6	2007.7	1830.4	2184.4	1593.8	1390.2	1796.0
	Iodine deficiency	7299.3	4505.9	2793.5	6992.2	4321.2	2671.0	117.1	145.6	88.9	139.4	174.4	105.3	109.4	137.4	82.4	122.8	154.1	92.7
	Vitamin A deficiency	770382.6	316065.9	454316.7	718346.7	291271.2	427075.4	12354.7	10214.3	14463.3	14322.3	11753.1	16831.8	13191.0	11016.1	15296.3	13762.6	11324.4	16150.1
2002	Protein-energy malnutrition	126394.9	56466.3	69928.5	79363.9	33987.9	45376.1	2001.0	1800.9	2198.2	1559.4	1351.0	1763.2	2006.3	1829.6	2182.4	1592.2	1390.1	1792.7
	Iodine deficiency	7476.0	4608.9	2867.0	7174.8	4428.4	2746.4	118.4	147.0	90.1	141.0	176.0	106.7	111.0	139.3	83.8	124.6	156.2	94.2
	Vitamin A deficiency	760524.5	314583.1	445941.4	709503.7	290136.5	419367.2	12040.3	10033.4	14018.4	13941.2	11532.7	16295.6	12879.6	10840.9	14853.8	13433.2	11152.4	15668.0
2003	Protein-energy malnutrition	127332.0	56888.5	70443.4	80154.7	34341.9	45812.8	1990.2	1790.8	2186.9	1552.4	1344.9	1755.5	2003.2	1827.4	2178.3	1587.9	1387.5	1786.6
	Iodine deficiency	7676.2	4724.4	2951.8	7381.4	4548.3	2833.2	120.0	148.7	91.6	143.0	178.1	108.6	113.0	141.7	85.5	126.8	158.8	96.1
	Vitamin A deficiency	751150.2	312368.9	438781.3	701035.8	288239.4	412796.3	11740.7	9833.3	13621.6	13577.7	11288.4	15817.6	12578.9	10640.8	14456.5	13121.8	10957.2	15244.1
2004	Protein-energy malnutrition	128367.6	57350.8	71016.8	81000.5	34699.2	46301.3	1981.1	1782.1	2177.4	1546.7	1339.1	1749.9	2001.9	1826.5	2176.5	1584.4	1384.6	1782.6
	Iodine deficiency	7853.3	4825.6	3027.7	7563.7	4653.0	2910.7	121.2	149.9	92.8	144.4	179.6	110.0	114.8	143.7	87.0	128.7	160.9	97.6
	Vitamin A deficiency	738627.8	308393.6	430234.2	689527.9	284650.5	404877.3	11399.1	9582.7	13191.4	13166.4	10985.5	15302.2	12231.0	10384.8	14020.4	12762.9	10702.9	14783.8
2005	Protein-energy malnutrition	129651.4	57905.7	71745.7	82075.2	35112.5	46962.8	1975.8	1776.2	2172.9	1545.4	1335.6	1751.0	2003.2	1827.4	2178.4	1585.4	1383.5	1785.7
	Iodine deficiency	7958.6	4885.0	3073.7	7671.6	4714.0	2957.5	121.3	149.8	93.1	144.4	179.3	110.3	115.5	144.4	87.7	129.3	161.6	98.3
	Vitamin A deficiency	721335.5	301880.1	419455.4	673473.2	278623.0	394850.3	10992.8	9260.0	12703.6	12680.7	10598.1	14722.2	11812.5	10049.3	13522.3	12329.2	10363.6	14258.6
2006	Protein-energy malnutrition	131398.5	58642.8	72755.7	83548.6	35632.8	47915.9	1977.1	1775.6	2176.1	1551.1	1335.8	1762.4	2009.5	1831.6	2186.8	1593.8	1386.2	1799.8
	Iodine deficiency	7984.6	4898.7	3085.9	7698.2	4728.2	2969.9	120.1	148.3	92.3	142.9	177.3	109.2	115.0	143.9	87.4	128.7	160.7	97.8
	Vitamin A deficiency	704986.4	295728.5	409257.9	658341.3	272953.4	385387.9	10607.7	8954.4	12240.9	12222.6	10232.8	14174.9	11417.6	9733.9	13051.0	11918.0	10040.7	13761.6
2007	Protein-energy malnutrition	133472.2	59497.1	73975.1	85309.6	36220.0	49089.5	1982.4	1777.9	2184.4	1561.5	1338.1	1780.8	2019.7	1838.6	2200.4	1606.7	1390.7	1821.3
	Iodine deficiency	7973.4	4892.2	3081.2	7687.3	4722.0	2965.3	118.4	146.2	91.0	140.7	174.5	107.6	114.1	142.7	86.6	127.4	159.2	96.8
	Vitamin A deficiency	690015.6	290071.9	399943.7	644502.5	267738.0	376764.4	10248.3	8668.2	11809.7	11796.7	9891.3	13667.7	11050.1	9439.9	12612.5	11534.2	9737.2	13299.6
2008	Protein-energy malnutrition	135492.9	60285.9	75207.1	87094.1	36780.5	50313.6	1986.9	1778.4	2193.0	1572.2	1339.5	1801.0	2030.3	1844.9	2215.6	1619.9	1394.0	1844.4
	Iodine deficiency	7947.5	4879.0	3068.5	7661.7	4709.1	2952.5	116.5	143.9	89.5	138.3	171.5	105.7	113.0	141.3	85.7	126.0	157.5	95.6

	Vitamin A deficiency	675461.4	284373.4	391088.0	631111.0	262517.3	368593.7	9905.1	8388.8	11404.0	11392.8	9560.5	13193.7	10698.5	9151.4	12199.9	11169.3	9442.0	12867.1
2009	Protein-energy malnutrition	137091.2	60835.8	76255.3	88652.6	37227.3	51425.3	1985.8	1772.2	2197.1	1579.0	1337.0	1817.2	2036.4	1845.7	2227.1	1629.0	1393.2	1863.4
	Iodine deficiency	7930.3	4873.5	3056.8	7644.8	4703.8	2940.9	114.9	142.0	88.1	136.2	168.9	103.9	112.0	140.3	84.9	124.9	156.3	94.6
	Vitamin A deficiency	657165.3	277243.9	379921.4	614143.8	255950.9	358192.9	9519.3	8076.5	10946.3	10938.8	9192.1	12657.5	10301.0	8825.8	11733.1	10753.3	9108.0	12371.0
2010	Protein-energy malnutrition	137952.6	61005.9	76946.7	89753.6	37484.4	52269.1	1974.3	1755.3	2191.1	1577.3	1327.5	1823.3	2031.8	1835.2	2228.5	1629.2	1385.3	1871.9
	Iodine deficiency	7947.5	4890.9	3056.7	7662.5	4721.5	2941.0	113.7	140.7	87.0	134.7	167.2	102.6	111.7	140.0	84.4	124.3	155.8	94.0
	Vitamin A deficiency	639513.0	270052.2	369460.8	597871.7	249366.2	348505.6	9152.5	7769.9	10520.8	10506.9	8831.5	12157.2	9920.2	8503.1	11296.5	10359.0	8780.7	11911.3
2011	Protein-energy malnutrition	137343.8	60365.7	76978.1	89810.9	37157.4	52653.5	1942.1	1715.5	2166.5	1557.3	1297.8	1813.2	2003.4	1798.6	2208.3	1611.3	1357.2	1864.4
	Iodine deficiency	7991.0	4933.1	3057.9	7706.6	4764.1	2942.5	113.0	140.2	86.1	133.6	166.4	101.3	111.6	140.3	84.0	124.2	156.1	93.4
	Vitamin A deficiency	623000.6	263409.5	359591.1	582677.2	243294.7	339382.5	8809.5	7485.9	10120.3	10103.5	8497.5	11687.0	9565.2	8206.3	10885.5	9990.8	8477.4	11479.7
2012	Protein-energy malnutrition	135259.8	58888.3	76371.6	88821.7	36182.7	52639.0	1889.8	1653.2	2124.3	1519.8	1246.5	1789.5	1953.2	1737.5	2169.0	1576.1	1307.5	1843.9
	Iodine deficiency	8032.9	4985.8	3047.1	7749.6	4817.4	2932.3	112.2	140.0	84.8	132.6	166.0	99.7	111.5	140.9	83.1	124.1	156.8	92.4
	Vitamin A deficiency	603520.9	255509.0	348011.9	564625.9	236031.7	328594.2	8432.3	7172.9	9680.2	9661.1	8131.0	11171.1	9171.3	7875.1	10431.0	9581.5	8139.3	11000.6
2013	Protein-energy malnutrition	132485.0	57056.9	75428.1	87434.7	34989.5	52445.1	1829.3	1582.5	2074.0	1476.4	1189.0	1760.3	1897.0	1670.3	2123.8	1534.6	1251.1	1817.5
	Iodine deficiency	8069.0	5039.1	3029.9	7787.0	4871.3	2915.8	111.4	139.8	83.3	131.5	165.5	97.9	111.2	141.5	82.1	123.9	157.6	91.3
	Vitamin A deficiency	583589.5	247363.8	336225.7	546138.4	228530.9	317607.5	8058.0	6860.8	9244.8	9221.8	7765.6	10660.1	8777.2	7541.3	9978.6	9172.6	7799.7	10523.5
2014	Protein-energy malnutrition	130044.6	55468.3	74576.3	86311.3	34017.8	52293.5	1775.1	1520.5	2027.6	1438.5	1140.5	1733.1	1849.6	1614.3	2085.1	1498.0	1203.1	1792.5
	Iodine deficiency	8094.6	5082.3	3012.4	7813.9	4915.1	2898.8	110.5	139.3	81.9	130.2	164.8	96.1	110.9	141.8	81.1	123.5	158.0	90.2
	Vitamin A deficiency	563341.0	239118.4	324222.6	527316.7	220914.9	306401.9	7689.4	6554.6	8815.0	8788.7	7406.6	10155.0	8388.1	7212.2	9531.6	8767.8	7464.6	10049.7
2015	Protein-energy malnutrition	129214.0	54825.1	74388.9	86123.7	33719.4	52404.3	1743.8	1485.5	2000.1	1417.2	1115.8	1715.5	1819.2	1579.6	2058.9	1477.6	1178.7	1776.3
	Iodine deficiency	8105.9	5104.8	3001.1	7826.2	4938.3	2887.9	109.4	138.3	80.7	128.8	163.4	94.5	110.4	141.7	80.4	123.0	157.8	89.4
	Vitamin A deficiency	543252.4	230882.4	312370.1	508594.3	213288.7	295305.6	7331.4	6255.9	8398.6	8369.3	7057.7	9666.9	8009.3	6889.3	9098.6	8375.8	7138.7	9592.1
2016	Protein-energy malnutrition	130094.8	55803.3	74291.5	87370.8	35153.0	52217.8	1735.9	1494.7	1975.2	1419.7	1148.2	1688.5	1810.3	1586.0	2034.6	1479.3	1209.2	1749.5
	Iodine deficiency	8084.1	5089.9	2994.3	7805.2	4924.0	2881.2	107.9	136.3	79.6	126.8	160.8	93.2	109.6	140.6	79.7	122.0	156.5	88.6
	Vitamin A deficiency	528288.8	224809.3	303479.4	494777.2	207728.9	287048.3	7049.0	6021.6	8068.7	8039.6	6785.0	9281.7	7713.4	6640.7	8756.8	8069.6	6885.6	9233.4
2017	Protein-energy malnutrition	132410.9	57888.6	74522.3	90114.4	37700.2	52414.1	1747.3	1533.2	1959.9	1445.8	1215.5	1673.9	1831.0	1631.8	2030.0	1503.8	1273.9	1734.3
	Iodine deficiency	8071.3	5080.3	2990.9	7793.0	4915.1	2878.0	106.5	134.6	78.7	125.0	158.5	91.9	108.8	139.7	79.2	121.0	155.4	87.9
	Vitamin A deficiency	514410.8	219022.6	295388.2	482082.6	202475.9	279606.7	6788.2	5801.0	7768.5	7734.6	6528.2	8929.7	7438.8	6406.1	8443.4	7785.4	6645.9	8905.4
2018	Protein-energy malnutrition	140101.0	61840.3	78260.7	97009.4	41274.1	55735.3	1829.3	1620.4	2036.9	1537.3	1314.0	1758.6	1925.5	1732.1	2119.0	1595.6	1373.8	1818.3
	Iodine deficiency	8089.4	5101.3	2988.1	7811.8	4936.5	2875.3	105.6	133.7	77.8	123.8	157.2	90.7	108.5	139.6	78.7	120.6	155.2	87.3
	Vitamin A deficiency	500223.0	213238.0	286985.0	468962.9	197178.9	271784.0	6531.6	5587.3	7469.5	7431.6	6277.4	8575.4	7168.3	6178.6	8131.0	7506.4	6415.7	8578.1
2019	Protein-energy malnutrition	154086.0	68206.0	85880.0	108645.9	46439.5	62206.5	1991.4	1768.6	2212.8	1701.4	1460.6	1940.2	2099.4	1894.6	2304.0	1762.8	1526.2	2000.0
	Iodine deficiency	8111.5	5130.5	2981.0	7834.5	4966.1	2868.4	104.8	133.0	76.8	122.7	156.2	89.5	108.3	139.8	78.1	120.3	155.3	86.6

Vitamin A deficiency	489662.7	208933.1	280729.6	459155.8	193227.3	265928.5	6328.5	5417.7	7233.5	7190.5	6077.5	8294.1	6955.6	5999.1	7886.2	7291.5	6238.7	8325.3

Table S3. Sex-specific disability-adjusted life-years (DALYs) counts, crude rate, age standardized rate of main subcategories in global and low-and middle-income countries (LMICs) by calendar year, 1990-2019.

Year	Main subcategories	Number (thousand)						Crude rate per100,000						Age standardized incidence rate per 100,000					
		Global			LMICs			Global			LMICs			Global			LMICs		
		Both	Female	Male	Both	Female	Male	Both	Female	Male	Both	Female	Male	Both	Female	Male	Both	Female	Male
1990	Protein-energy malnutrition	52743.9	26625.8	26118.1	29292.4	15569.7	13722.7	985.9	1002.4	969.6	697.5	750.7	645.5	855.3	882.3	832.8	625.0	672.1	583.4
	Iodine deficiency	2500.0	1396.2	1103.7	2416.0	1342.5	1073.6	46.7	52.6	41.0	57.5	64.7	50.5	46.8	52.9	40.9	58.8	66.4	51.4
	Vitamin A deficiency	1967.4	787.0	1180.4	1729.0	696.1	1032.9	36.8	29.6	43.8	44.7	36.7	52.5	31.9	26.3	37.3	33.6	28.0	38.9
	Dietary iron deficiency	25069.8	14672.6	10397.2	22478.3	13137.5	9340.7	468.6	552.4	386.0	535.2	633.4	439.4	458.5	550.1	368.9	526.3	639.2	416.3
	Other nutritional deficiencies	5566.4	2907.1	2659.3	4587.5	2484.3	2103.2	104.0	109.4	98.7	109.2	119.8	98.9	104.4	108.7	101.1	116.2	124.9	108.5
1991	Protein-energy malnutrition	50606.8	25548.4	25058.4	28206.6	14969.1	13237.5	931.5	947.6	915.6	659.7	709.3	611.4	817.4	843.3	796.0	596.7	640.5	558.3
	Iodine deficiency	2488.8	1404.4	1084.4	2404.3	1350.3	1054.0	45.8	52.1	39.6	56.2	64.0	48.7	46.0	52.4	39.7	57.6	65.7	49.8
	Vitamin A deficiency	1944.7	778.4	1166.3	1710.8	689.4	1021.4	35.8	28.9	42.6	43.5	35.7	51.0	31.3	25.8	36.5	32.9	27.4	38.1
	Dietary iron deficiency	25316.8	14821.4	10495.4	22751.7	13305.3	9446.4	466.0	549.7	383.5	532.1	630.4	436.3	456.6	547.9	367.3	523.6	636.4	413.9
	Other nutritional deficiencies	5416.6	2854.6	2562.1	4474.9	2444.5	2030.3	99.7	105.9	93.6	104.7	115.8	93.8	100.2	105.2	96.3	111.2	120.5	103.0
1992	Protein-energy malnutrition	48468.7	24435.9	24032.9	27185.6	14381.0	12804.6	878.9	893.3	864.8	624.9	669.9	581.0	781.8	805.6	762.2	571.9	612.2	536.6
	Iodine deficiency	2476.4	1411.0	1065.5	2391.5	1356.5	1034.9	44.9	51.6	38.3	55.0	63.2	47.0	45.1	51.8	38.4	56.4	64.9	48.2
	Vitamin A deficiency	1922.8	770.7	1152.1	1693.2	683.5	1009.7	34.9	28.2	41.5	42.2	34.7	49.5	30.7	25.4	35.8	32.3	26.9	37.3
	Dietary iron deficiency	25534.6	14953.1	10581.5	22999.9	13458.9	9541.0	463.0	546.6	380.8	528.7	627.0	432.9	454.4	545.2	365.5	520.6	633.0	411.1
	Other nutritional deficiencies	5225.9	2775.4	2450.4	4320.9	2377.8	1943.2	94.8	101.5	88.2	99.3	110.8	88.2	95.6	101.1	91.0	105.7	115.4	97.0
1993	Protein-energy malnutrition	46566.5	23387.2	23179.3	26206.4	13780.9	12425.5	832.3	842.9	821.8	592.3	631.4	554.2	751.2	771.4	735.2	549.7	585.3	518.9
	Iodine deficiency	2465.0	1416.8	1048.1	2379.6	1362.2	1017.4	44.1	51.1	37.2	53.8	62.4	45.4	44.2	51.2	37.2	55.2	64.0	46.7
	Vitamin A deficiency	1902.6	764.3	1138.2	1676.9	678.6	998.3	34.0	27.5	40.4	41.1	33.9	48.1	30.2	25.0	35.1	31.7	26.6	36.5
	Dietary iron deficiency	25729.6	15072.2	10657.4	23225.8	13600.4	9625.5	459.9	543.3	377.9	524.9	623.1	429.3	452.0	542.3	363.6	517.4	629.4	408.4
	Other nutritional deficiencies	4988.2	2654.3	2334.0	4117.7	2269.3	1848.5	89.2	95.7	82.8	93.1	104.0	82.4	90.3	95.6	85.8	99.3	108.4	91.1
1994	Protein-energy malnutrition	44905.1	22479.4	22425.7	25214.5	13179.9	12034.6	791.3	798.9	783.7	560.5	594.0	527.8	724.8	742.2	711.3	528.2	559.6	501.4
	Iodine deficiency	2456.3	1422.8	1033.5	2370.6	1367.9	1002.7	43.3	50.6	36.1	52.7	61.6	44.0	43.3	50.6	36.2	54.1	63.1	45.3
	Vitamin A deficiency	1885.0	759.8	1125.2	1662.8	675.4	987.4	33.2	27.0	39.3	40.1	33.2	46.7	29.8	24.8	34.6	31.2	26.3	35.9
	Dietary iron deficiency	25911.8	15185.9	10726.0	23437.2	13735.4	9701.7	456.6	539.7	374.8	520.9	619.0	425.5	449.6	539.3	361.8	514.1	625.4	405.6
	Other nutritional deficiencies	4747.7	2518.5	2229.2	3903.5	2143.1	1760.4	83.7	89.5	77.9	86.8	96.6	77.2	85.1	89.8	81.1	93.0	101.2	85.7
1995	Protein-energy malnutrition	47442.0	23543.8	23898.2	26834.4	13821.8	13012.6	824.3	825.2	823.5	586.8	612.8	561.5	774.9	784.8	768.1	578.4	601.6	558.5

	Iodine deficiency	2453.6	1429.7	1023.9	2367.6	1374.6	992.9	42.6	50.1	35.3	51.8	60.9	42.8	42.6	50.1	35.3	53.1	62.3	44.1
	Vitamin A deficiency	1871.1	757.7	1113.4	1651.6	674.0	977.6	32.5	26.6	38.4	39.1	32.6	45.5	29.5	24.6	34.1	30.9	26.1	35.3
	Dietary iron deficiency	26086.6	15298.6	10788.1	23638.6	13867.9	9770.7	453.3	536.2	371.7	516.9	614.8	421.6	447.2	536.4	359.9	510.8	621.5	402.8
	Other nutritional deficiencies	4319.8	2327.6	1992.1	3556.5	1987.5	1568.9	75.1	81.6	68.6	77.8	88.1	67.7	76.3	82.0	71.5	83.3	92.3	75.1
1996	Protein-energy malnutrition	45782.4	22715.6	23066.8	25218.7	13007.1	12211.6	784.5	785.1	783.8	542.6	567.3	518.5	742.2	753.4	734.1	532.3	556.6	511.4
	Iodine deficiency	2439.1	1428.6	1010.5	2352.9	1373.4	979.5	41.8	49.4	34.3	50.6	59.9	41.6	41.7	49.2	34.3	51.9	61.1	42.8
	Vitamin A deficiency	1859.0	757.8	1101.2	1641.8	674.5	967.3	31.9	26.2	37.4	38.3	32.0	44.3	29.2	24.6	33.6	30.5	26.0	34.8
	Dietary iron deficiency	26244.5	15404.2	10840.3	23824.9	13995.0	9829.9	449.7	532.4	368.4	512.6	610.4	417.4	444.8	533.3	358.0	507.2	617.2	399.8
	Other nutritional deficiencies	4204.9	2273.7	1931.3	3444.1	1930.2	1513.9	72.1	78.6	65.6	74.1	84.2	64.3	73.8	79.4	68.9	79.9	88.7	71.9
1997	Protein-energy malnutrition	44006.1	21797.2	22208.9	24194.0	12421.2	11772.9	743.8	743.1	744.4	512.3	533.1	492.1	713.1	722.9	706.2	509.3	530.6	491.2
	Iodine deficiency	2404.8	1415.2	989.6	2318.7	1360.2	958.5	40.6	48.2	33.2	49.1	58.4	40.1	40.5	48.0	33.1	50.2	59.4	41.2
	Vitamin A deficiency	1847.4	759.3	1088.0	1632.5	676.3	956.1	31.2	25.9	36.5	37.4	31.6	43.1	29.0	24.6	33.1	30.3	26.0	34.3
	Dietary iron deficiency	26369.6	15493.5	10876.1	23983.8	14109.9	9873.9	445.7	528.2	364.6	507.9	605.6	412.7	441.9	529.7	355.7	503.2	612.4	396.3
	Other nutritional deficiencies	4049.0	2197.0	1852.0	3317.5	1862.6	1454.9	68.4	74.9	62.1	70.2	79.9	60.8	70.4	76.0	65.5	76.0	84.4	68.2
1998	Protein-energy malnutrition	42119.3	20815.8	21303.5	23177.4	11820.0	11357.5	702.4	700.0	704.7	483.2	499.4	467.5	682.3	690.2	677.0	486.3	503.7	471.8
	Iodine deficiency	2366.3	1398.8	967.5	2280.3	1343.9	936.3	39.5	47.0	32.0	47.5	56.8	38.5	39.2	46.7	31.9	48.5	57.7	39.5
	Vitamin A deficiency	1835.1	761.4	1073.8	1622.4	678.6	943.9	30.6	25.6	35.5	36.6	31.2	41.9	28.7	24.6	32.6	30.0	26.0	33.7
	Dietary iron deficiency	26468.3	15569.9	10898.4	24117.7	14212.7	9905.0	441.4	523.6	360.5	502.8	600.5	407.7	438.8	525.9	353.3	498.9	607.2	392.7
	Other nutritional deficiencies	3832.7	2085.3	1747.4	3135.0	1762.0	1373.0	63.9	70.1	57.8	65.4	74.4	56.5	65.9	71.4	61.0	70.6	78.7	63.1
1999	Protein-energy malnutrition	40303.2	19883.9	20419.3	22175.0	11242.7	10932.3	663.3	659.8	666.8	455.4	467.7	443.4	652.3	658.8	648.1	457.2	463.4	452.0
	Iodine deficiency	2340.6	1388.3	952.3	2254.8	1333.7	921.1	38.5	46.1	31.1	46.3	55.5	37.4	38.2	45.6	30.9	47.9	58.0	38.2
	Vitamin A deficiency	1822.8	763.1	1059.7	1612.4	680.6	931.9	30.0	25.3	34.6	35.8	30.8	40.7	28.5	24.6	32.1	29.2	25.2	33.2
	Dietary iron deficiency	26556.3	15643.7	10912.6	24167.6	14240.2	9927.4	437.1	519.1	356.3	496.3	592.3	402.7	435.8	522.2	351.0	494.6	607.4	389.1
	Other nutritional deficiencies	3604.4	1981.3	1623.1	2932.7	1663.8	1268.9	59.3	65.7	53.0	60.6	70.2	51.5	61.3	67.0	56.0	66.0	74.9	57.4
2000	Protein-energy malnutrition	37164.2	18301.1	18863.1	21078.4	10574.2	10504.2	603.8	599.3	608.2	426.5	433.2	419.9	602.9	606.9	601.1	443.5	451.2	437.7
	Iodine deficiency	2347.9	1395.1	952.8	2262.4	1340.8	921.6	38.1	45.7	30.7	45.8	54.9	36.8	37.7	45.1	30.5	46.4	55.4	37.6
	Vitamin A deficiency	1810.7	763.7	1047.0	1602.3	681.3	921.0	29.4	25.0	33.8	35.1	30.3	39.7	28.3	24.6	31.7	29.5	26.0	32.8
	Dietary iron deficiency	26644.8	15719.7	10925.2	24352.6	14406.3	9946.3	432.9	514.8	352.2	492.7	590.2	397.6	433.0	518.6	348.7	490.4	596.9	385.7
	Other nutritional deficiencies	3371.6	1865.6	1505.9	2747.4	1569.8	1177.6	54.8	61.1	48.6	55.6	64.3	47.1	56.6	62.4	51.3	59.7	67.6	52.3
2001	Protein-energy malnutrition	35258.1	17334.7	17923.4	20089.4	10018.7	10070.6	565.4	560.2	570.6	400.5	404.3	396.9	570.6	573.4	569.8	420.3	425.3	417.0
	Iodine deficiency	2381.8	1415.7	966.1	2297.3	1362.2	935.1	38.2	45.8	30.8	45.8	55.0	36.9	37.7	45.0	30.4	46.3	55.3	37.4
	Vitamin A deficiency	1792.7	761.4	1031.4	1586.6	679.3	907.3	28.8	24.6	32.8	34.2	29.8	38.5	28.0	24.5	31.2	29.2	25.8	32.3
	Dietary iron deficiency	26703.1	15788.6	10914.6	24432.0	14491.5	9940.5	428.2	510.2	347.5	487.1	584.7	391.8	429.7	515.0	345.8	485.6	591.6	381.4

	Other nutritional deficiencies	3186.2	1757.1	1429.1	2588.6	1472.6	1115.9	51.1	56.8	45.5	51.6	59.4	44.0	53.0	58.1	48.2	55.6	62.6	48.8
2002	Protein-energy malnutrition	33501.3	16489.6	17011.7	19207.3	9560.3	9647.0	530.4	525.9	534.8	377.4	380.0	374.9	540.2	543.4	538.8	399.3	403.4	396.7
	Iodine deficiency	2421.5	1438.6	982.9	2339.1	1386.5	952.5	38.3	45.9	30.9	46.0	55.1	37.0	37.7	45.1	30.5	46.3	55.2	37.4
	Vitamin A deficiency	1766.1	756.0	1010.1	1562.8	674.4	888.5	28.0	24.1	31.8	33.2	29.1	37.2	27.6	24.4	30.6	28.7	25.6	31.5
	Dietary iron deficiency	26712.9	15841.0	10872.0	24461.1	14559.6	9901.5	422.9	505.2	341.8	480.6	578.7	384.7	425.6	510.7	341.8	479.9	585.5	375.8
	Other nutritional deficiencies	2947.0	1633.3	1313.8	2378.9	1359.2	1019.7	46.7	52.1	41.3	46.7	54.0	39.6	48.5	53.5	44.0	50.5	57.2	44.3
2003	Protein-energy malnutrition	28706.1	14211.9	14494.2	16197.3	8096.4	8100.9	448.7	447.4	450.0	313.7	317.1	310.4	458.4	465.0	454.0	328.9	335.0	324.9
	Iodine deficiency	2463.3	1462.3	1001.0	2383.2	1412.0	971.2	38.5	46.0	31.1	46.2	55.3	37.2	37.8	45.1	30.6	46.3	55.2	37.5
	Vitamin A deficiency	1734.6	748.6	985.9	1534.5	667.6	866.9	27.1	23.6	30.6	32.1	28.4	35.8	27.0	24.1	29.8	28.1	25.3	30.7
	Dietary iron deficiency	26704.2	15888.6	10815.6	24468.0	14620.6	9847.4	417.4	500.2	335.8	473.9	572.6	377.3	421.2	506.2	337.3	473.8	579.2	369.8
	Other nutritional deficiencies	2948.6	1591.0	1357.6	2355.9	1304.5	1051.4	46.1	50.1	42.1	45.6	51.1	40.3	48.0	51.5	44.8	49.4	54.1	44.9
2004	Protein-energy malnutrition	27124.1	13436.4	13687.7	15395.8	7690.9	7704.8	418.6	417.5	419.7	294.0	296.8	291.2	430.5	437.0	426.2	309.9	315.6	306.3
	Iodine deficiency	2502.7	1484.8	1017.9	2424.5	1435.8	988.7	38.6	46.1	31.2	46.3	55.4	37.4	37.8	45.2	30.6	46.3	55.2	37.5
	Vitamin A deficiency	1702.9	740.8	962.2	1506.1	660.3	845.8	26.3	23.0	29.5	31.1	27.7	34.4	26.5	23.8	29.0	27.5	24.9	29.9
	Dietary iron deficiency	26714.1	15943.6	10770.6	24488.7	14685.7	9803.0	412.3	495.4	330.2	467.6	566.8	370.5	417.1	502.0	333.2	468.0	573.1	364.1
	Other nutritional deficiencies	2812.5	1514.8	1297.6	2244.1	1238.8	1005.3	43.4	47.1	39.8	42.9	47.8	38.0	45.1	48.4	42.2	46.1	50.4	42.0
2005	Protein-energy malnutrition	25868.1	12815.4	13052.7	14815.3	7400.3	7415.1	394.2	393.1	395.3	279.0	281.5	276.5	407.9	414.2	403.8	295.4	300.9	291.9
	Iodine deficiency	2536.3	1504.1	1032.3	2458.7	1455.5	1003.1	38.7	46.1	31.3	46.3	55.4	37.4	37.8	45.1	30.6	46.1	55.0	37.4
	Vitamin A deficiency	1675.3	733.7	941.6	1481.7	654.0	827.7	25.5	22.5	28.5	30.2	27.0	33.2	26.0	23.5	28.3	26.9	24.5	29.1
	Dietary iron deficiency	26774.0	16017.5	10756.5	24554.4	14765.7	9788.8	408.0	491.3	325.8	462.3	561.6	365.0	413.8	498.5	330.1	463.2	567.9	359.6
	Other nutritional deficiencies	2735.4	1474.7	1260.7	2189.2	1207.6	981.6	41.7	45.2	38.2	41.2	45.9	36.6	43.3	46.5	40.4	44.1	48.3	40.2
2006	Protein-energy malnutrition	24790.1	12309.7	12480.4	14301.7	7157.6	7144.1	373.0	372.7	373.3	265.5	268.3	262.8	388.1	394.9	383.2	282.4	288.2	278.4
	Iodine deficiency	2552.6	1514.0	1038.5	2474.5	1465.2	1009.3	38.4	45.8	31.1	45.9	54.9	37.1	37.5	44.8	30.4	45.7	54.4	37.1
	Vitamin A deficiency	1646.8	724.5	922.3	1457.1	646.0	811.1	24.8	21.9	27.6	29.2	26.3	32.1	25.4	23.1	27.6	26.3	24.1	28.4
	Dietary iron deficiency	26861.5	16099.8	10761.7	24644.0	14851.0	9793.0	404.2	487.5	321.9	457.5	556.7	360.2	410.8	495.2	327.4	458.7	562.8	355.7
	Other nutritional deficiencies	2692.8	1447.4	1245.4	2165.5	1189.2	976.3	40.5	43.8	37.3	40.2	44.6	35.9	42.1	45.0	39.4	43.0	46.8	39.4
2007	Protein-energy malnutrition	23676.8	11773.7	11903.1	13742.4	6876.7	6865.6	351.7	351.8	351.5	251.5	254.1	249.1	367.4	374.4	362.4	268.5	274.1	264.7
	Iodine deficiency	2548.4	1512.1	1036.3	2469.8	1462.9	1006.9	37.8	45.2	30.6	45.2	54.0	36.5	37.0	44.1	29.9	44.9	53.5	36.4
	Vitamin A deficiency	1612.3	711.5	900.8	1427.3	634.6	792.7	23.9	21.3	26.6	28.2	25.4	30.9	24.7	22.5	26.8	25.6	23.5	27.6
	Dietary iron deficiency	26943.3	16183.5	10759.7	24725.6	14935.7	9789.9	400.2	483.6	317.7	452.6	551.8	355.1	407.6	491.8	324.2	453.9	557.5	351.3
	Other nutritional deficiencies	2641.5	1407.7	1233.9	2132.6	1158.1	974.5	39.2	42.1	36.4	39.0	42.8	35.4	40.7	43.2	38.5	41.7	44.8	38.8
2008	Protein-energy malnutrition	22702.1	11316.6	11385.5	13269.1	6647.0	6622.1	332.9	333.8	332.0	239.5	242.1	237.0	349.3	356.8	343.8	256.5	262.2	252.6
	Iodine deficiency	2534.5	1504.9	1029.5	2455.5	1455.5	1000.0	37.2	44.4	30.0	44.3	53.0	35.8	36.3	43.3	29.3	43.9	52.4	35.6

	Vitamin A deficiency	1573.7	695.8	877.9	1394.1	620.9	773.2	23.1	20.5	25.6	27.2	24.5	29.8	24.0	21.9	26.0	24.9	22.8	26.7
	Dietary iron deficiency	27031.6	16272.8	10758.9	24813.2	15025.3	9787.9	396.4	480.0	313.7	447.9	547.2	350.4	404.5	488.8	321.1	449.4	552.6	347.0
	Other nutritional deficiencies	2637.1	1402.5	1234.6	2138.0	1155.7	982.3	38.7	41.4	36.0	38.6	42.1	35.2	40.0	42.4	38.0	41.0	44.0	38.4
2009	Protein-energy malnutrition	21698.8	10819.8	10879.0	12773.6	6386.7	6386.9	314.3	315.2	313.4	227.5	229.4	225.7	331.4	338.5	326.2	244.4	249.3	241.3
	Iodine deficiency	2523.0	1500.1	1022.8	2443.5	1450.4	993.1	36.5	43.7	29.5	43.5	52.1	35.1	35.7	42.6	28.8	43.1	51.4	34.8
	Vitamin A deficiency	1533.8	679.0	854.8	1359.8	606.2	753.5	22.2	19.8	24.6	26.1	23.6	28.6	23.3	21.2	25.2	24.1	22.1	25.9
	Dietary iron deficiency	27142.9	16374.9	10768.0	24924.4	15127.8	9796.6	393.2	477.0	310.2	443.9	543.3	346.2	401.9	486.3	318.4	445.5	548.4	343.3
	Other nutritional deficiencies	2585.3	1370.6	1214.7	2099.3	1128.4	970.9	37.4	39.9	35.0	37.4	40.5	34.3	38.7	40.9	36.8	39.6	42.2	37.3
2010	Protein-energy malnutrition	21551.5	10720.7	10830.8	12582.1	6273.5	6308.6	308.4	308.5	308.4	221.1	222.2	220.1	327.0	333.1	322.6	238.3	242.4	235.9
	Iodine deficiency	2525.8	1504.9	1020.8	2446.0	1455.0	991.1	36.1	43.3	29.1	43.0	51.5	34.6	35.3	42.3	28.4	42.5	50.8	34.3
	Vitamin A deficiency	1497.1	663.7	833.4	1328.5	593.1	735.5	21.4	19.1	23.7	25.2	22.7	27.5	22.6	20.6	24.4	23.3	21.5	25.1
	Dietary iron deficiency	27297.7	16498.2	10799.4	25080.1	15251.6	9828.5	390.7	474.7	307.5	440.8	540.1	342.9	400.1	484.6	316.5	442.5	545.3	340.4
	Other nutritional deficiencies	2556.3	1339.0	1217.4	2075.8	1102.8	973.0	36.6	38.5	34.7	36.5	39.1	33.9	37.7	39.4	36.4	38.6	40.6	36.8
2011	Protein-energy malnutrition	20796.9	10343.0	10453.9	12226.6	6081.3	6145.3	294.1	293.9	294.2	212.0	212.4	211.6	313.5	319.2	309.5	229.3	232.6	227.5
	Iodine deficiency	2526.5	1512.6	1013.9	2446.5	1462.4	984.1	35.7	43.0	28.5	42.4	51.1	33.9	34.9	41.9	27.9	41.9	50.4	33.6
	Vitamin A deficiency	1458.3	647.6	810.7	1295.4	579.2	716.3	20.6	18.4	22.8	24.2	21.9	26.5	21.8	20.0	23.6	22.6	20.8	24.3
	Dietary iron deficiency	27489.9	16641.5	10848.4	25272.5	15394.7	9877.8	388.7	472.9	305.3	438.2	537.7	340.2	398.9	483.5	315.1	440.2	542.8	338.2
	Other nutritional deficiencies	2524.5	1323.2	1201.3	2055.4	1090.5	964.9	35.7	37.6	33.8	35.6	38.1	33.2	36.8	38.4	35.4	37.6	39.5	35.9
2012	Protein-energy malnutrition	19348.3	9637.9	9710.4	11596.1	5749.6	5846.4	270.3	270.6	270.1	198.4	198.1	198.8	289.4	295.0	285.3	215.4	217.8	214.3
	Iodine deficiency	2503.9	1511.4	992.5	2423.7	1461.1	962.6	35.0	42.4	27.6	41.5	50.3	32.7	34.1	41.4	26.9	41.0	49.6	32.4
	Vitamin A deficiency	1412.4	627.9	784.5	1255.8	562.0	693.8	19.7	17.6	21.8	23.1	20.9	25.3	21.0	19.2	22.7	21.8	20.0	23.4
	Dietary iron deficiency	27695.2	16794.0	10901.2	25477.5	15546.8	9930.6	387.0	471.5	303.2	435.9	535.6	337.6	397.7	482.5	313.7	438.1	540.6	336.1
	Other nutritional deficiencies	2493.7	1309.9	1183.8	2037.1	1078.2	959.0	34.8	36.8	32.9	34.9	37.1	32.6	35.8	37.5	34.3	36.6	38.5	35.0
2013	Protein-energy malnutrition	18672.3	9309.3	9362.9	11272.1	5592.5	5679.6	257.8	258.2	257.4	190.3	190.0	190.6	277.4	283.0	273.2	207.3	209.7	206.2
	Iodine deficiency	2470.9	1506.3	964.7	2390.6	1455.8	934.8	34.1	41.8	26.5	40.4	49.5	31.4	33.3	40.7	25.9	39.9	48.8	31.1
	Vitamin A deficiency	1364.2	607.1	757.2	1214.3	543.8	670.4	18.8	16.8	20.8	22.1	20.0	24.1	20.2	18.5	21.8	20.9	19.2	22.4
	Dietary iron deficiency	27905.7	16952.1	10953.6	25689.7	15705.5	9984.2	385.3	470.2	301.2	433.8	533.7	335.1	396.7	481.7	312.3	436.2	538.7	334.1
	Other nutritional deficiencies	2414.2	1268.6	1145.5	1970.2	1041.8	928.5	33.3	35.2	31.5	33.3	35.4	31.2	34.2	35.8	32.8	34.9	36.6	33.5
2014	Protein-energy malnutrition	17960.6	8966.9	8993.8	10960.5	5443.8	5516.7	245.2	245.8	244.5	182.7	182.5	182.8	265.4	271.1	261.1	199.6	202.1	198.3
	Iodine deficiency	2444.6	1503.8	940.8	2364.1	1453.2	911.0	33.4	41.2	25.6	39.4	48.7	30.2	32.5	40.1	24.9	38.9	48.0	29.9
	Vitamin A deficiency	1317.7	586.9	730.8	1174.4	526.3	648.0	18.0	16.1	19.9	21.0	19.0	23.0	19.4	17.8	20.9	20.0	18.5	21.5
	Dietary iron deficiency	28112.6	17109.3	11003.2	25902.2	15865.1	10037.1	383.7	469.0	299.2	431.7	531.9	332.7	395.7	481.1	311.1	434.3	536.8	332.2
	Other nutritional deficiencies	2361.1	1248.0	1113.1	1930.5	1025.9	904.6	32.2	34.2	30.3	32.2	34.4	30.0	33.0	34.8	31.5	33.6	35.4	32.1

2015	Protein-energy malnutrition	17332.8	8658.6	8674.2	10708.0	5325.0	5382.9	233.9	234.6	233.2	176.2	176.2	176.2	254.3	259.8	250.1	193.1	195.8	191.6
	Iodine deficiency	2436.2	1506.9	929.3	2355.7	1456.2	899.5	32.9	40.8	25.0	38.8	48.2	29.4	32.0	39.8	24.4	38.3	47.5	29.1
	Vitamin A deficiency	1278.1	569.8	708.3	1140.7	511.6	629.0	17.2	15.4	19.0	20.2	18.2	22.0	18.7	17.1	20.1	19.3	17.8	20.8
	Dietary iron deficiency	28312.4	17261.7	11050.7	26108.4	16020.1	10088.2	382.1	467.7	297.1	429.6	530.1	330.2	394.6	480.3	309.8	432.6	535.1	330.4
	Other nutritional deficiencies	2308.8	1218.8	1090.0	1888.0	1000.9	887.1	31.2	33.0	29.3	31.1	33.1	29.0	31.8	33.5	30.4	32.4	34.0	31.0
2016	Protein-energy malnutrition	16795.2	8418.4	8376.8	10562.7	5293.2	5269.5	224.1	225.5	222.7	171.6	172.9	170.4	244.0	250.1	239.2	188.4	192.5	185.7
	Iodine deficiency	2426.3	1503.2	923.1	2345.7	1452.5	893.1	32.4	40.3	24.5	38.1	47.4	28.9	31.5	39.2	23.9	37.6	46.8	28.6
	Vitamin A deficiency	1244.3	555.4	688.9	1112.4	499.5	612.9	16.6	14.9	18.3	19.4	17.6	21.2	18.1	16.6	19.5	18.7	17.3	20.1
	Dietary iron deficiency	28498.4	17412.4	11086.0	26303.1	16174.3	10128.7	380.3	466.4	294.7	427.4	528.3	327.5	393.2	479.3	308.0	430.6	533.3	328.3
	Other nutritional deficiencies	2289.7	1220.8	1068.9	1880.4	1008.6	871.7	30.6	32.7	28.4	30.6	32.9	28.2	31.1	33.0	29.3	31.7	33.6	29.9
2017	Protein-energy malnutrition	16284.8	8204.5	8080.2	10416.6	5270.9	5145.7	214.9	217.3	212.5	167.1	169.9	164.3	235.2	242.2	229.5	183.6	189.2	179.2
	Iodine deficiency	2422.2	1502.4	919.8	2341.6	1451.7	889.8	32.0	39.8	24.2	37.6	46.8	28.4	31.1	38.8	23.6	37.1	46.1	28.1
	Vitamin A deficiency	1215.8	543.3	672.5	1089.3	489.6	599.6	16.0	14.4	17.7	18.7	17.0	20.5	17.6	16.1	18.9	18.2	16.8	19.5
	Dietary iron deficiency	28626.7	17530.5	11096.2	26448.1	16300.2	10147.9	377.8	464.3	291.8	424.3	525.5	324.1	391.2	477.5	305.7	427.8	530.5	325.4
	Other nutritional deficiencies	2290.7	1235.4	1055.3	1893.5	1029.4	864.1	30.2	32.7	27.8	30.4	33.2	27.6	30.6	33.0	28.5	31.3	33.7	29.1
2018	Protein-energy malnutrition	15612.9	7835.5	7777.3	10100.2	5084.3	5015.9	203.9	205.3	202.4	160.1	161.9	158.3	224.8	230.5	220.3	175.7	180.1	172.5
	Iodine deficiency	2434.6	1513.1	921.5	2353.9	1462.4	891.5	31.8	39.6	24.0	37.3	46.6	28.1	31.0	38.6	23.4	36.8	45.9	27.8
	Vitamin A deficiency	1193.7	534.0	659.7	1071.5	482.1	589.4	15.6	14.0	17.2	18.2	16.5	19.9	17.2	15.8	18.5	17.8	16.5	19.1
	Dietary iron deficiency	28633.6	17569.0	11064.7	26476.2	16349.2	10127.0	373.9	460.3	288.0	419.6	520.5	319.5	387.9	473.9	302.7	423.3	525.5	321.5
	Other nutritional deficiencies	2304.9	1237.8	1067.1	1919.1	1038.7	880.4	30.1	32.4	27.8	30.4	33.1	27.8	30.4	32.6	28.5	31.2	33.4	29.1
2019	Protein-energy malnutrition	15256.5	7612.3	7644.3	9988.4	4988.4	5000.1	197.2	197.4	197.0	156.4	156.9	155.9	218.3	222.5	215.1	171.7	174.7	169.8
	Iodine deficiency	2438.6	1519.9	918.7	2357.9	1469.2	888.7	31.5	39.4	23.7	36.9	46.2	27.7	30.7	38.4	23.1	36.4	45.5	27.4
	Vitamin A deficiency	1177.5	527.0	650.5	1057.8	476.2	581.6	15.2	13.7	16.8	17.8	16.1	19.4	16.9	15.5	18.2	17.5	16.2	18.8
	Dietary iron deficiency	28534.7	17540.0	10994.7	26390.6	16327.7	10063.0	368.8	454.8	283.3	413.3	513.5	313.9	383.4	468.7	298.8	417.4	518.6	316.5
	Other nutritional deficiencies	2367.8	1257.3	1110.5	1983.1	1059.9	923.2	30.6	32.6	28.6	31.1	33.3	28.8	30.8	32.7	29.2	31.6	33.5	29.9

Table S4. Age- and Sex-specific incidence rate and disability-adjusted life-years (DALYs) rate of overall nutritional deficiencies and high-risk subcategories in low-and middle-income countries (LMICs) by calendar year, 1990-2019.

year	Age group	Nutritional deficiencies						High-risk subcategories								
		ASIR			ASDR			ASIR for protein energy malnutrition			ASIR for vitamin A deficiency			ASDR for dietary iron deficiency		
		Both	Female	Male	Both	Female	Male	Both	Female	Male	Both	Female	Male	Both	Female	Male

1990	1 to 4	5798.6	5570.3	6013.7	6567.1	7080	6083.9	5671.4	5433.8	5895.3	38281.3	31552.4	44621.9	975.9	963.9	987.3
	5 to 14	1126.6	1016.8	1230.9	1220.8	1291.2	1153.8	793.5	652.7	927.3	21295.0	16218.7	26118.5	735.9	786.2	687.9
	15 to 44	1118.9	1016	1214.3	560.5	718.3	407.3	950.7	814.1	1099.2	17142.8	12741.0	21115.9	329.7	456.5	206.8
	45 to 59	1164.8	1002.1	1320.6	886.1	1119.3	663	1147.2	985	1302.6	12541.7	9051.6	15881.4	555	787.8	332.2
	60 to 74	1227.4	1052.4	1417.3	1212.6	1295.3	1126.4	1217.8	1043.1	1407.5	10457.1	7595.5	13558.1	588.9	695.7	474.7
	75+	1240.8	1061.7	1511.5	1963.6	1831.2	2184.3	1236.2	1057.1	1506.9	8579.2	6160.9	12184.4	661.3	674.6	644.5
1991	1 to 4	5755.2	5474.7	6019.1	6274.2	6757.4	5819.5	5639.8	5346.9	5915.5	37662.9	31095.9	43842.2	973.3	960.7	985.1
	5 to 14	1111	1000.4	1215.8	1207.7	1284.9	1134.3	801.8	653.2	942.7	21016.1	16085.2	25692.1	734.1	783.3	687.3
	15 to 44	1133.8	1024.6	1235	551.4	708.1	399.4	957.6	814.6	1112.7	16845.9	12664.9	20651.0	327.4	454.7	204.2
	45 to 59	1175.8	1007.1	1337.5	872.8	1102.4	653	1158.2	990	1319.4	12376.5	8955.7	15653.2	550.4	781.7	328.8
	60 to 74	1233.8	1054.4	1427.6	1189.9	1272.8	1103.8	1224.2	1045.1	1417.8	10303.9	7488.3	13341.1	585.5	692.6	471.6
	75+	1252	1069.9	1525.8	1920.5	1794.1	2131	1247.3	1065.2	1521.1	8440.3	6050.6	11986.7	659.4	674.6	640.1
1992	1 to 4	5743.5	5424.6	6043.1	6030.3	6482.4	5605.5	5638.6	5304.3	5952.7	37212.4	30767.9	43267.7	972.2	958.9	984.7
	5 to 14	1097.5	985.8	1203.1	1184.3	1260.3	1112.4	810.9	655	958.3	20784.2	15964.8	25344.4	731.3	779.3	685.7
	15 to 44	1150	1033.8	1257	544	700.1	392.5	967	817.3	1128.7	16655.8	12622.3	20330.8	325.1	452.5	201.5
	45 to 59	1185.7	1011.2	1353.1	857.3	1086.4	637.9	1168.1	994.1	1335	12216.3	8844.3	15447.0	544.7	774.4	324.6
	60 to 74	1243.4	1058.9	1441.9	1162.7	1249	1073.5	1233.8	1049.5	1432	10176.7	7388.0	13171.7	581.4	688.7	467.8
	75+	1268.4	1082.6	1545.5	1880	1762.7	2074.9	1263.7	1077.9	1540.8	8331.3	5955.7	11832.3	657.2	674.7	634.8
1993	1 to 4	5758.4	5413.4	6082.2	5827.1	6244.5	5435.5	5662.5	3669.9	6003	36162.0	29618.4	42022.1	972.7	958.7	985.9
	5 to 14	1086.6	973.3	1193.5	1157.1	1230	1088.2	820.4	486.5	973.9	19952.0	15725.2	24219.3	728.2	775	683.9
	15 to 44	1166.5	1043	1279	534.5	688.3	385.2	977.9	720	1145.8	15994.2	14155.1	19355.6	322.5	450	198.8
	45 to 59	1197	1016.2	1370.7	840.7	1066.2	624.4	1179.5	957.7	1352.7	11629.8	9815.2	14643.4	538.4	766	320.1
	60 to 74	1254.7	1064.7	1458.8	1131.7	1216.4	1044.5	1245.1	1044.8	1449	9701.8	7696.9	12496.0	576.9	684.5	463.3
	75+	1287.5	1077.2	1568.6	1845.6	1733.3	2031.4	1282.9	1063.7	1563.9	8026.1	6073.0	11319.6	654.4	674.6	628.6
1994	1 to 4	5791.5	3755.8	6128.9	5641.7	6024.2	5283.3	5702.5	3644	6058.4	35255.5	28689.6	40952.3	974.5	959.7	988.3
	5 to 14	1078.9	668.5	1187.9	1125.7	1192.7	1062.5	830	482.9	988.8	19228.8	15300.9	23249.0	725.1	770.7	682
	15 to 44	1182.3	830.7	1300.3	526.4	678	379	989.6	720.5	1163.7	15451.2	13841.8	18541.5	319.9	447.3	196.2
	45 to 59	1208.2	975.9	1388.4	822.8	1045.7	609.1	1190.7	959.2	1370.3	11071.5	9547.3	13878.9	530.7	755.8	314.8
	60 to 74	1269.6	1061.8	1479.2	1100	1185.9	1012	1259.9	1052.1	1469.3	9282.2	7580.1	11889.5	572.5	681.2	458.2
	75+	1303.5	1080.7	1590.9	1797.4	1687.9	1977.1	1298.8	1072.6	1586.2	7736.8	5934.6	10852.6	649.1	670.7	621.3
1995	1 to 4	5833.7	3729.9	6175.2	5594.7	5952	5260.3	5749.5	3620.6	6110.4	34626.1	27744.6	40141.2	976.4	960.8	991
	5 to 14	1075	663.1	1187.1	1110.9	1173.2	1052.2	839.3	480.7	1002.8	18696.5	14844.6	22511.0	722.7	767.2	680.7
	15 to 44	1196.5	832.2	1319.8	539.6	685.4	397.8	1001.4	721.7	1181.5	15064.2	13544.9	17928.6	317.2	444.5	193.5

	45 to 59	1219.3	976.7	1406.3	903.5	1103.1	711.8	1201.7	959.9	1388.3	10706.9	9292.2	13389.6	523.2	745.3	309.7
	60 to 74	1283.3	1066.8	1499.1	1251.9	1335.3	1165.7	1273.6	1057	1489.1	9028.3	7538.6	11519.7	567.7	677.4	452.4
	75+	1322.3	1094	1615.8	2129	2082.3	2209.7	1317.6	1085.7	1611.1	7584.4	5882.5	10579.8	643.5	667	613.4
1996	1 to 4	5897.5	3715.2	6216.8	5496	5832.8	5181.1	5816.7	3609.1	6155.8	33987.7	26822.4	39329.7	977	960.5	992.4
	5 to 14	1077.4	659.5	1193.1	1095	1155.6	1038.1	852.9	480.1	1020.3	18173.1	14404.8	21821.1	721.6	765	680.8
	15 to 44	1212.6	833.2	1342.1	519.5	666.8	376.2	1017.9	723.3	1204.1	14635.0	13220.6	17332.1	314.1	441	190.7
	45 to 59	1236.4	979.3	1430.8	818.4	1030.5	614.4	1218.8	962.6	1412.8	10353.3	9050.3	12960.1	515.6	735.1	304.3
	60 to 74	1301.2	1071.3	1524.8	1098.3	1188.3	1005.8	1291.5	1061.5	1514.9	8760.2	7490.9	11175.5	561.8	672.8	445.2
	75+	1343	1104.8	1645.5	1908.3	1843.4	2013.5	1338.3	1096.5	1640.7	7408.6	5832.5	10313.3	634.3	659.1	602.6
1997	1 to 4	5989.9	3713.7	6255.6	5277.1	5590.7	4984.3	5912.1	3612.3	6198.1	33266.7	26190.8	38283.2	975.7	958.1	992.3
	5 to 14	1086.5	655.3	1205.3	1078.7	1134.2	1026.7	872.8	479.7	1043.2	17577.9	13974.0	20998.1	721.8	764	682.3
	15 to 44	1232.7	832.9	1369.4	511.8	657.2	370.1	1040.8	724.6	1233.7	14214.3	12818.0	16634.4	310.9	437.4	187.7
	45 to 59	1260.6	981.6	1463.3	799	1007.5	598	1243.1	964.9	1445.2	9749.3	8744.6	12403.6	506.8	723.5	298
	60 to 74	1324.1	1074.9	1556.6	1074.4	1166.6	979.1	1314.4	1065	1546.7	8354.7	7378.5	10720.1	554.3	666.7	436.1
	75+	1369.2	1116.8	1682.6	1845.6	1788.9	1937.8	1364.4	1108.5	1677.8	6822.0	5753.1	9942.1	621.1	647.1	587.8
1998	1 to 4	3821.9	3715.7	6292.6	5055.8	5339.3	4791.1	3749.2	3619.6	6238.1	30128.6	25971.9	37568.3	973.1	954.1	990.8
	5 to 14	821.2	650	1220.8	1064.5	1113.3	1018.9	648.3	478.4	1067.4	15442.3	13642.7	20425.5	723.1	764	684.9
	15 to 44	1128.6	831.1	1396.8	503.5	646.7	363.7	1013.8	724.8	1264.3	14156.4	12438.3	16133.7	307.5	433.7	184.6
	45 to 59	1373.3	983.5	1497.5	775	978.8	578.3	1353.7	966.8	1479.5	9420.8	8450.3	11985.6	496.9	710.1	291
	60 to 74	1421.5	1078.1	1588.6	1043.7	1139.4	944.2	1410.9	1068.2	1578.6	8149.8	7249.8	10413.1	546.5	660.5	426.2
	75+	1389.8	1124.3	1718.1	1774.5	1726.7	1852.7	1381.2	1116.1	1713.4	6642.8	5665.7	9689.4	604.9	631.2	571.1
1999	1 to 4	3816.7	3713.3	6329.9	4841.3	4747.9	4609.9	3748.1	3622.2	6277.3	29844.8	27084.1	37398.2	969.7	897.1	988.2
	5 to 14	817.9	644.3	1236.3	1048.8	1080.8	1004.5	646.2	476	1088.8	15003.4	13816.3	20221.0	725.3	758.6	688
	15 to 44	1131.6	828.8	1419.7	496.2	649.6	357.2	1016.6	867.7	1290.3	13603.7	10836.2	15888.4	304.2	438.5	181.5
	45 to 59	1385.2	985.1	1526.6	751.3	980.1	559	1365.6	1144.6	1508.6	9063.8	7468.5	11783.2	486.1	712.6	283.6
	60 to 74	1430.8	1081.1	1614.7	1007.2	1175.8	904.1	1420.2	1216.1	1604.8	7948.4	6482.6	10343.8	539.3	689	416.8
	75+	1394.1	1127.8	1743.5	1702.7	1872.5	1761.9	1385.5	1792.6	1738.8	6482.4	6079.1	9598.9	588.2	666.8	554.7
2000	1 to 4	3804.2	6124.4	6368.2	4457.8	4668.4	4261.5	3738.4	6028	6316.2	28841.6	29067.4	36858.8	966.3	946	985.3
	5 to 14	814.8	989.8	1249.1	1033.8	1078.2	992.4	643.7	736.5	1103.3	14420.1	13874.9	19789.0	727.7	766.9	691.2
	15 to 44	1132.6	1116.6	1433.5	494.4	632.7	359.2	1017.3	904.8	1306	12949.3	10525.4	15472.7	300.7	425.9	178.4
	45 to 59	1391.3	1090.1	1542.9	765.2	952.5	583.8	1371.7	1073	1524.9	8610.1	6726.2	11429.6	475.9	681.6	276.6
	60 to 74	1436.5	1147.1	1628.3	1063.8	1157.9	964.3	1425.9	1137.5	1618.4	7661.1	5895.5	10137.2	533.1	650.7	408.5
	75+	1394.6	1179.9	1752.4	1691.9	1676.1	1725.5	1385.7	1175.2	1747.7	6277.9	4899.0	9365.5	572.3	597.8	539.6

2001	1 to 4	3779.6	6168.7	6413.4	4236.1	4418.8	4065.8	3714.8	6070.6	6360.5	27746.2	28876.9	35881.1	960.2	940	979.1
	5 to 14	811.7	998.3	1259	1025.6	1066.3	987.7	638.5	741	1110.8	14072.9	13789.0	19158.6	729.4	768.3	693.1
	15 to 44	1124.7	1118.4	1436	485.6	622.3	351.6	1010	906.9	1308.7	12369.6	10216.9	14804.8	296.8	421.6	174.7
	45 to 59	1382.2	1087.7	1540	742.2	926.6	563.5	1362.8	1070.6	1522.1	8016.3	6475.1	10832.2	465.6	668	269.2
	60 to 74	1431.5	1146.8	1622.9	1041.5	1137.4	939.7	1421	1137.2	1613	7254.6	5734.2	9640.1	526.5	645.4	400.3
	75+	1387	1173.4	1736.3	1632.6	1622.8	1656.9	1377.8	1168.7	1731.6	6001.3	4758.0	8892.5	556.7	581.5	524.9
2002	1 to 4	3741.7	6202.4	6464.8	4047.4	4221.7	3884.9	3676.7	6101.2	6409.8	16171.9	28631.5	35142.1	950.2	930.3	968.8
	5 to 14	804.6	1008.3	1268	1009.1	1049.5	971.4	627.8	743.5	1114.6	8066.5	13758.7	18763.2	730	768.9	693.6
	15 to 44	1102.8	1118	1431.2	474.9	610.2	342.1	990.4	905.3	1302.9	6280.9	9992.6	14306.5	292.4	417.3	170.1
	45 to 59	1353.1	1079.2	1522.9	718.9	900.7	542.3	1334.3	1062.2	1505	3378.9	6291.3	10364.1	454.9	654.4	261
	60 to 74	1414.6	1139.2	1602.1	1016.3	1114.2	912.1	1404.2	1129.6	1592.1	2810.9	5633.2	9271.3	518.4	638.6	390.7
	75+	1372.3	1160.1	1701.9	1573.5	1569.5	1589.5	1362.6	1155.4	1697.2	2826.7	4639.1	8505.9	540.3	564.3	509.6
2003	1 to 4	4990.4	6226.1	6514.6	3745.5	3903.1	3598.6	4887	6121.4	6457.2	15609.8	28239.2	34367.9	938.4	919	956.4
	5 to 14	815.1	1018.4	1276.3	987.5	1028.1	949.7	561.4	744.7	1116.5	7860.1	13658.1	18389.2	730.2	769.5	693.5
	15 to 44	1009.4	1116.4	1424.3	449.8	586.7	315.4	826.4	902.1	1294.8	4801.8	9760.1	13881.9	287.6	412.6	165.1
	45 to 59	1141.2	1067.2	1500.1	642.2	832.7	456.7	1119.6	1050.3	1482.3	3275.6	6128.5	9976.6	444.3	640.9	252.7
	60 to 74	1267	1130	1578.6	841.4	950.6	725.8	1255.2	1120.4	1568.6	2737.8	5549.3	8997.8	510	631.5	380.7
	75+	1381	1145.4	1663.4	1294.2	1242.8	1378.6	1362.6	1140.8	1658.7	2725.5	4546.2	8199.0	525	548.9	494.9
2004	1 to 4	4851.4	6239.8	6554.3	3557.1	3709.1	3415.4	4755.7	6132.1	6494.7	14982.7	27730.6	33514.7	926.6	907.7	944.3
	5 to 14	812.5	1027.2	1284	983.8	1025.3	945.2	562	745.3	1118.1	7585.9	13447.3	17932.8	731.1	771	694
	15 to 44	1000.4	1114.8	1420.1	440.8	576.4	307.4	824.7	899.5	1289.9	5921.7	9480.6	13422.7	282.9	407.7	160.3
	45 to 59	1137	1056.3	1482.1	623	811.6	438.8	1115.3	1039.4	1464.4	3150.7	5935.2	9586.1	434.1	628.1	244.6
	60 to 74	1260.7	1122.6	1561.8	814.3	925.4	696.7	1249	1113.1	1551.8	2647.3	5437.5	8735.3	501.9	624.7	371.5
	75+	1370.8	1131.6	1631.9	1238.8	1193	1315.2	1352.3	1126.9	1627.2	2610.5	4430.4	7898.9	511.8	535.8	482
2005	1 to 4	4742.3	6242.9	6574.9	3412.1	3557.8	3276.4	4652.3	6133.4	6513.8	14349.6	27019.6	32536.1	917.2	898.6	934.5
	5 to 14	809.4	1033.6	1291.7	983.1	1027.9	941.3	561.9	746	1121.6	7284.3	13111.4	17372.0	733.4	774	695.5
	15 to 44	994.4	1114	1423	433.1	567.1	301.3	824.4	899.7	1293.2	5700.4	9126.7	12909.4	278.6	402.7	156.6
	45 to 59	1135.1	1050	1478.6	607.6	793.3	425.7	1113.5	1033.2	1461	3026.5	5707.5	9166.2	425.5	617	238
	60 to 74	1258.7	1120.2	1562.7	794.5	907.6	674.7	1246.9	1110.6	1552.7	2555.2	5269.8	8413.3	494.2	617.4	363.4
	75+	1364.7	1126	1622.6	1199.3	1160.5	1265.6	1346	1121.4	1617.9	2497.2	4287.1	7567.5	501.5	526.2	471.1
2006	1 to 4	4719.5	6222.7	6573.2	3281.2	3430.6	3141.9	4634.2	6113.6	6511.5	13644.0	26209.1	31531.3	908.1	889.7	925.2
	5 to 14	803.5	1037	1302.5	978.8	1025.4	935.4	561.8	748	1130.2	6950.4	12819.8	16841.3	735.6	777.1	697.1
	15 to 44	993.1	1117.1	1436.5	427	559.4	296.7	824.7	905.1	1308.6	5445.5	8803.6	12437.4	275	398.5	153.6

	45 to 59	1134	1054.2	1496	594.8	777.4	415.5	1112.5	1037.4	1478.5	2884.7	5503.2	8790.5	417.5	606.3	232.1
	60 to 74	1260	1127.8	1588.7	778.3	890.6	659.4	1248.2	1118.2	1578.7	2440.8	5093.0	8076.6	485	607.4	355
	75+	1362	1133.9	1643.1	1164.8	1131.3	1223.1	1343.4	1129.3	1638.4	2373.8	4157.3	7256.6	491.5	517.1	460.2
2007	1 to 4	4709	6173.3	6553.9	3137.8	3287.4	2998.4	4630.2	6066.4	6491.8	13047.6	25406.2	30500.4	896.9	878.7	913.8
	5 to 14	792.2	1037.2	1316.6	971.5	1017.9	928.4	561	750.1	1142.9	6680.2	12553.0	16347.7	736.8	779.1	697.5
	15 to 44	994	1123.9	1457	421.3	552.4	292.2	823.6	914.1	1332	5223.6	8508.8	12018.4	272.1	395.5	150.8
	45 to 59	1131.5	1068.2	1529.6	581.7	760.5	405.6	1110.3	1051.4	1512.2	2756.7	5308.3	8447.1	408.6	594	225.9
	60 to 74	1258.5	1142.1	1628.4	759.2	869.2	643	1246.8	1132.5	1618.4	2337.1	4914.6	7750.1	474.6	595.8	345.9
	75+	1361	1156.7	1687.8	1128	1096.9	1182.6	1342.5	1152.1	1683.1	2259.9	4031.8	6961.6	481.1	507.8	448.5
2008	1 to 4	4697.7	6104.6	6521.1	3004.6	3155.5	2863.9	4625.6	6000.7	6458.7	12488.8	24610.4	29486.4	885.1	867	902
	5 to 14	777.4	1033.7	1330.4	966.4	1013.9	922.3	558.8	750.2	1155.6	6451.5	12255.3	15867.2	737.5	780.7	697.4
	15 to 44	993.7	1130.7	1479.3	417.5	548.2	288.8	820.4	922.6	1357.1	5034.8	8240.5	11649.1	270.1	393.8	148.4
	45 to 59	1128	1085.4	1569.6	568.8	743.8	395.7	1107.2	1068.7	1552.3	2632.4	5114.7	8106.4	399.4	581.3	219.5
	60 to 74	1255	1160.3	1676	741.6	848.5	628.8	1243.4	1150.7	1666.1	2229.5	4742.6	7448.6	464.3	583.9	337.4
	75+	1359.7	1185.1	1743.6	1101.9	1074.3	1151.5	1341.3	1180.4	1738.9	2147.0	3907.1	6673.4	470.9	498.9	437.2
2009	1 to 4	4678.1	6027.2	6478.9	2872.7	3016.8	2738.2	4611.7	5926.2	6416.5	11940.2	23706.8	28360.1	874.1	856	891
	5 to 14	762.2	1026.6	1339.7	958.8	1006.4	914.5	554.6	746.6	1164.4	6223.5	11880.4	15307.7	738.4	782.3	697.4
	15 to 44	990.5	1133.9	1497.6	413.7	544.1	285.3	815.3	926.6	1377.9	4861.4	7951.5	11228.3	268.7	393	146.6
	45 to 59	1123.2	1099.9	1605.9	557.4	729.1	387.1	1102.7	1083.2	1588.8	2518.8	4907.2	7728.9	391.4	570.3	213.9
	60 to 74	1252.5	1175.9	1722.7	722.8	827.1	612.8	1241	1166.2	1712.8	2123.9	4528.4	7044.8	453.4	571	328.6
	75+	1357.8	1212.6	1798.8	1074.6	1050.8	1119.3	1339.5	1207.9	1794.1	2038.8	3771.3	6342.6	463.2	492.8	427.4
2010	1 to 4	4646.9	5952.3	6431.5	2799.1	2933.2	2674	4583.7	5852.9	6369.2	11427.6	22790.1	27295.2	865.4	847.4	882.2
	5 to 14	750.6	1015.7	1340.2	953.5	1000.6	909.6	548.6	737.5	1164.7	5987.7	11475.2	14760.1	739.5	784.2	697.9
	15 to 44	984.2	1129.7	1506	411.2	541.5	283	819.8	922.4	1387.7	4688.0	7680.1	10840.7	267.7	392.3	145.2
	45 to 59	1120.6	1106.1	1631.8	548.8	718	380.6	1100.4	1089.5	1614.8	2415.6	4713.5	7385.6	384.4	560.9	208.8
	60 to 74	1249.8	1182.5	1753.5	711.4	813.2	603.6	1238.3	1172.8	1743.7	2028.4	4339.2	6714.4	445.3	560.9	322.4
	75+	1355.8	1228.9	1838.5	1059	1039.2	1098.3	1337.4	1224.2	1833.8	1945.3	3651.7	6066.1	459.1	490.6	421.2
2011	1 to 4	4578.6	5856	6361.6	2705.7	2833.9	2586	4516.9	5758.6	6300.4	11135.2	21906.7	26256.2	858.9	840.9	875.7
	5 to 14	728.4	982.7	1319.1	947.2	993.8	903.8	529.5	706.9	1145.6	5846.8	11126.0	14238.4	741.1	786.5	698.8
	15 to 44	966	1106.3	1499	408.1	538.6	279.8	791.4	897	1380.9	4571.7	7427.0	10483.4	267	391.9	144.2
	45 to 59	1110.7	1093.4	1645	539.5	706.4	373.1	1090.4	1076.8	1628.2	2329.6	4529.8	7068.1	378.1	552.2	204.4
	60 to 74	1241.8	1174.2	1775.6	701.1	802.7	593.4	1230.3	1164.5	1765.8	1957.7	4165.5	6417.2	439	553.5	317.2
	75+	1348.5	1227.4	1867.7	1042.6	1028.3	1073.9	1330.2	1222.7	1863	1867.5	3531.9	5801.3	457	489.9	417.1

2012	1 to 4	4465.3	5719.4	6257.1	2573.8	2693.3	2462.3	4405.3	5625.6	6198.5	10784.4	20884.6	25069.8	852.6	834.7	869.4
	5 to 14	687	921.8	1275.2	936.9	982.8	894.1	493.2	651.5	1106.9	5677.7	10697.6	13651.9	742.6	788.6	699.7
	15 to 44	933.7	1064.1	1479.7	403.7	534.4	275.3	757.5	850.4	1361.2	4444.9	7160.5	10092.1	266.5	391.9	143.2
	45 to 59	1088.2	1064.2	1654	530.3	695.2	365.3	1067.8	1047.7	1637.3	2234.6	4342.0	6731.7	372.5	544.5	200.5
	60 to 74	1224.2	1153	1798	688.7	791.3	579.7	1212.5	1143.4	1788.3	1876.0	3976.6	6076.6	432.5	545.8	311.8
	75+	1333.3	1213.2	1897.3	1023	1014.2	1046.1	1314.9	1208.5	1892.6	1783.7	3393.9	5509.8	454.4	487.7	413.7
2013	1 to 4	4327.7	5560.5	6130.8	2497.1	2616.8	2385.2	4269.5	5471	6075.4	10325.0	19857.3	23849.9	846	828.2	862.7
	5 to 14	638.8	852.7	1221.7	926.6	973.4	882.8	450.7	589.4	1060.1	5450.3	10232.3	13057.9	743.6	790.1	700.3
	15 to 44	896.8	1017	1455.4	399.5	530.6	270.6	717.8	798.3	1336	4275.6	6900.4	9706.2	266.2	392.3	142.4
	45 to 59	1060.8	1030.5	1660.5	522.2	685.8	358.1	1040.2	1014.1	1644	2125.8	4167.7	6415.9	367.9	538.2	197.2
	60 to 74	1203.9	1129.1	1821.5	670.7	771.9	563	1192.1	1119.5	1811.9	1777.1	3788.4	5740.2	425.6	537.3	306.4
	75+	1315.5	1195.6	1926.4	1012.8	1007	1031.6	1297.2	1190.9	1921.8	1685.8	3257.1	5224.9	451.5	485.3	410.1
2014	1 to 4	4194.2	5403.1	6004.4	2420	2541	2306.9	4137.6	5317.8	5952.1	10003.0	18862.9	22643.9	839.5	821.8	856
	5 to 14	597.2	794.3	1171.8	918	966	873.1	414.1	537.9	1016.7	5276.4	9761.8	12475.0	743.9	790.6	700.2
	15 to 44	865.5	979.3	1433.2	396.1	527.8	266.7	683.6	756.1	1313.1	4155.7	6643.5	9318.9	266.2	393	141.7
	45 to 59	1037.2	1004.4	1666.6	516.3	679.8	352.2	1016.4	988.1	1650.3	2041.1	4000.4	6108.2	364.2	533.2	194.6
	60 to 74	1188.4	1111.6	1845	653.6	752.8	547.9	1176.5	1102	1835.4	1693.2	3597.9	5399.1	418.5	528.5	301
	75+	1301.9	1183.3	1954.8	1002.4	1002	1013.5	1283.6	1178.7	1950.2	1598.7	3124.9	4937.2	448.5	482.9	406
2015	1 to 4	4108.1	5270.6	5900	2349.8	2470.7	2236.9	4052.3	5188.1	5849.8	9749.3	17908.5	21472.9	833.8	816.2	850.2
	5 to 14	577.2	765.4	1138.7	910.3	958.4	865.4	396.7	514	988.3	5120.0	9277.9	11912.2	743.1	789.9	699.4
	15 to 44	850.6	964.3	1420.2	393.7	526	263.6	667.3	739.1	1299.7	4060.4	6399.0	8946.4	266.2	393.6	141.1
	45 to 59	1027.9	998.3	1674.7	511.8	674.6	348.2	1006.9	982.1	1658.4	1958.1	3843.9	5812.7	361.1	529	192.4
	60 to 74	1184.7	1110	1865.8	642.6	740.1	538.4	1172.7	1100.4	1856.2	1607.4	3413.9	5064.8	412.4	520.6	296.6
	75+	1299.4	1186.4	1979.2	987.6	990.6	993.4	1281	1181.8	1974.6	1514.2	2993.2	4655.1	445.8	480.5	402.8
2016	1 to 4	4033.1	5060.4	5661.6	2290.9	2416.9	2173.1	3978.1	4980.1	5613	9472.1	17082.2	20457.3	827.8	810.4	844
	5 to 14	575.7	790.1	1097.4	902.2	951.9	855.7	395.7	542.6	950.2	4987.1	8954.7	11473.5	740.2	786.7	696.7
	15 to 44	853.8	1012.1	1405.7	393.4	528	261.1	668	787.9	1285.4	3947.6	6205.1	8665.0	266.6	395	140.4
	45 to 59	1031.6	1067.9	1691.7	508.1	671	344.4	1010.5	1051.6	1675.6	1879.7	3723.5	5593.6	358	524.9	190.2
	60 to 74	1187.6	1196	1900.8	633.4	730.4	529.6	1175.4	1186.4	1891.4	1525.2	3271.8	4813.0	406.9	513.7	292.4
	75+	1303.8	1279.6	2029.1	976	981	977.9	1285.1	1274.9	2024.5	1444.7	2893.1	4451.2	444.4	479.1	401.4
2017	1 to 4	3986.2	4902	5453.3	2220.6	2351.4	2098.4	3931.5	4823.3	5405.7	9181.4	16309.7	19513.4	819.9	802.8	835.8
	5 to 14	581.7	847.1	1069.7	893	944.4	844.9	400.7	602.6	924.9	4838.8	8649.6	11066.6	734.6	780.6	691.6
	15 to 44	864.4	1095.6	1400.5	394	531.3	259.1	673.7	872.5	1280.5	3823.4	6017.0	8403.0	266.4	395.8	139.4

	45 to 59	1037.4	1168.1	1719.3	504.3	667.4	340.3	1015.9	1151.6	1703.2	1820.4	3604.2	5396.0	354.1	519.7	187.5
	60 to 74	1188.6	1307.2	1942.2	625	722.1	521	1176.2	1297.6	1933	1453.7	3152.1	4615.7	401.8	507.4	288.5
	75+	1307.9	1394.8	2082.8	967	974.5	964.7	1288.9	1390.1	2078.2	1386.3	2811.9	4301.1	442.8	477.1	400.3
2018	1 to 4	4145.8	5039	5531.6	2110.1	2226.4	2001.4	4088.8	4961.3	5484.7	8901.3	15782.2	18863.2	810.7	793.5	826.9
	5 to 14	637.3	948.2	1168.2	882.8	932.4	836.4	450.9	705.4	1024.9	4686.5	8332.6	10636.7	726.2	771.2	684
	15 to 44	915.6	1197.9	1488.1	395.3	532.7	260.3	725.9	974.4	1368.8	3693.4	5813.6	8109.2	264.8	394.2	137.6
	45 to 59	1084.6	1257.3	1797.3	499	660.3	336.6	1063.6	1240.9	1781.2	1758.1	3471.2	5170.6	348.8	512.6	183.8
	60 to 74	1227.2	1382.2	2000.4	614.6	710.2	512	1215.2	1372.7	1991.1	1379.9	3025.8	4401.4	396.1	500.2	284.1
	75+	1346.3	1464.7	2133.3	952.5	961.8	947.5	1326.8	1460.1	2128.6	1324.8	2722.5	4124.8	439.6	473.4	397.8
2019	1 to 4	4432.3	5417.8	5812.6	2026.3	2133.4	1926.2	4370.1	5340.7	5766	8673.3	15391.4	18384.5	801.9	784.1	818.6
	5 to 14	745	1108.3	1391.3	874.1	920.6	830.5	545.6	866.5	1249.2	4568.4	8083.4	10298.3	715.4	759	674.6
	15 to 44	1011.9	1338.2	1674.5	397.4	533.3	264	825.6	1113.9	1556.3	3590.1	5657.0	7882.9	261.7	390.8	134.9
	45 to 59	1173.6	1365.8	1934.6	494.1	652.5	334.5	1153.5	1349.3	1918.4	1707.1	3369.2	4995.4	342.3	504	179.4
	60 to 74	1305.2	1463.9	2090.8	604.6	698.5	503.5	1293.6	1454.4	2081.4	1317.3	2925.8	4227.0	389.1	491.8	278.4
	75+	1415.4	1538	2199.2	936.5	945.7	931.3	1395.2	1533.4	2194.5	1272.2	2650.2	3979.5	434	466.5	394.2

Table S5. Sex-specific incidence counts, crude rate, age-standardized rate in 2019 and the estimated annual percentage change (EAPC) for age-standardized rate of nutritional deficiencies and its main subcategories from 1990 to 2019 at national level in low-and middle-income countries (LMICs).

Country	Cause	Both				Female				Male			
		Number	Crude rate	ASIR	EAPC	Number	Crude rate	ASIR	EAPC	Number	Crude rate	ASIR	EAPC
China	Overall nutritional deficiencies	28569879.2	2008.6	2058.9	0.6(0.4,0.8)	10531820.2	1509.9	1625.5	-0.3(-0.5,0.0)	18038059.0	2488.6	2482.3	1.3(1.1,1.4)
	Protein-energy malnutrition	27834269.9	1956.9	1996.5	0.6(0.4,0.8)	10028640.8	1437.7	1625.5	-0.3(-0.6,-0.1)	17805629.1	2456.6	2444.7	1.3(1.2,1.4)
	Iodine deficiency	735609.3	51.7	62.4	0.2(-0.3,0.6)	503179.4	72.1	89.5	0.7(0.3,1.1)	232429.9	32.1	37.6	-0.7(-1.3,-0.1)
	Vitamin A deficiency	25263603.3	1776.2	2113.3	-5.7(-5.9,-5.5)	11412862.4	1636.2	1951.8	-4.8(-5.1,-4.4)	13850740.9	1910.9	2264.7	-6.3(-6.4,-6.2)
Democratic People's Republic of Korea	Overall nutritional deficiencies	558021.2	2127.2	2413.6	-1.7(-2.3,-1.1)	192517.1	1460.5	1691.5	-0.8(-1.1,-0.5)	365504.2	2800.6	3132.7	-2.2(-3.0,-1.4)
	Protein-energy malnutrition	556980.0	2123.2	2409.7	-1.7(-2.3,-1.1)	191863.6	1455.5	1691.5	-0.8(-1.1,-0.5)	365116.4	2797.6	3129.8	-2.2(-3.0,-1.4)
	Iodine deficiency	1041.3	4.0	3.9	-1.7(-1.9,-1.5)	653.5	5.0	5.0	-1.4(-1.6,-1.2)	387.8	3.0	2.9	-2.1(-2.3,-1.8)
	Vitamin A deficiency	1577940.1	6015.1	7105.4	-3.3(-3.6,-3.1)	630626.5	4784.1	5691.1	-2.9(-3.3,-2.6)	947313.5	7258.5	8454.5	-3.6(-3.9,-3.4)
Cambodia	Overall nutritional deficiencies	307888.6	1854.4	1866.1	-1.5(-1.7,-1.3)	134272.2	1588.8	1603.3	-1.5(-1.7,-1.3)	173616.4	2129.8	2154.9	-1.5(-1.7,-1.3)
	Protein-energy malnutrition	301705.6	1817.2	1832.0	-1.3(-1.5,-1.2)	130040.0	1538.7	1603.3	-1.3(-1.5,-1.0)	171665.6	2105.9	2133.0	-1.4(-1.6,-1.3)
	Iodine deficiency	6182.9	37.2	34.1	-5.8(-6.3,-5.3)	4232.2	50.1	46.7	-5.8(-6.3,-5.3)	1950.8	23.9	21.8	-5.7(-6.2,-5.2)

	Vitamin A deficiency	1163972.8	7010.6	7755.0	-5.4(-5.5,-5.2)	575638.8	6811.2	7575.3	-4.9(-5.0,-4.8)	588334.0	7217.2	7935.3	-5.8(-5.9,-5.7)
Indonesia	Overall nutritional deficiencies	8559739.2	3299.0	3574.8	0.2(0.0,0.3)	3927875.4	3055.1	3325.8	0.5(0.4,0.7)	4631863.8	3538.5	3828.8	-0.1(-0.3,0.1)
	Protein-energy malnutrition	8507822.1	3279.0	3555.7	0.2(0.1,0.4)	3893579.7	3028.5	3325.8	0.6(0.4,0.7)	4614242.4	3525.0	3816.0	-0.1(-0.2,0.1)
	Iodine deficiency	51917.1	20.0	19.1	-2.3(-2.4,-2.2)	34295.7	26.7	25.6	-2.3(-2.4,-2.2)	17621.4	13.5	12.9	-2.3(-2.4,-2.2)
	Vitamin A deficiency	13341449.4	5141.9	5770.6	-5.1(-5.2,-5.0)	7324694.2	5697.2	6237.3	-4.2(-4.4,-4.1)	6016755.2	4596.5	5317.6	-5.8(-6.0,-5.7)
Lao People's Democratic Republic	Overall nutritional deficiencies	147132.0	2055.4	2036.0	-1.4(-1.6,-1.3)	69563.8	1953.7	1929.4	-1.0(-1.2,-0.8)	77568.2	2156.1	2146.7	-1.8(-1.9,-1.6)
	Protein-energy malnutrition	145524.2	2033.0	2015.7	-1.4(-1.6,-1.3)	68478.6	1923.2	1929.4	-1.0(-1.2,-0.8)	77045.6	2141.6	2133.3	-1.8(-1.9,-1.6)
	Iodine deficiency	1607.8	22.5	20.3	-2.2(-2.3,-2.1)	1085.2	30.5	27.3	-2.1(-2.2,-2.0)	522.6	14.5	13.4	-2.2(-2.3,-2.0)
	Vitamin A deficiency	792528.2	11071.5	11596.3	-4.0(-4.3,-3.7)	360215.1	10116.6	10615.7	-3.7(-4.0,-3.5)	432313.1	12016.6	12560.0	-4.2(-4.5,-3.9)
Malaysia	Overall nutritional deficiencies	739835.1	2363.6	2529.4	-0.5(-0.7,-0.3)	282764.2	1871.8	2016.0	-0.5(-0.6,-0.3)	457070.9	2822.3	3013.2	-0.5(-0.7,-0.2)
	Protein-energy malnutrition	725825.6	2318.8	2488.5	-0.2(-0.4,0.0)	272970.5	1807.0	2016.0	-0.1(-0.2,0.1)	452855.1	2796.3	2989.3	-0.3(-0.6,-0.1)
	Iodine deficiency	14009.5	44.8	40.9	-6.0(-6.4,-5.7)	9793.7	64.8	59.2	-5.8(-6.1,-5.5)	4215.8	26.0	24.0	-6.5(-6.9,-6.0)
	Vitamin A deficiency	156266.9	499.2	546.7	-6.2(-6.8,-5.7)	93126.3	616.5	675.0	-5.5(-6.1,-5.0)	63140.6	389.9	427.9	-7.0(-7.6,-6.5)
Maldives	Overall nutritional deficiencies	23937.1	4802.7	5012.4	-0.2(-0.4,0.0)	6355.2	3205.0	3234.6	-0.9(-1.2,-0.7)	17581.9	5858.3	6344.0	0.1(-0.1,0.3)
	Protein-energy malnutrition	23872.3	4789.6	5000.1	-0.2(-0.4,0.0)	6319.3	3186.9	3234.6	-0.9(-1.2,-0.7)	17553.0	5848.6	6335.1	0.1(-0.1,0.3)
	Iodine deficiency	64.8	13.0	12.2	-4.7(-4.8,-4.6)	35.8	18.1	17.2	-4.6(-4.7,-4.4)	29.0	9.7	8.9	-4.2(-4.3,-4.1)
	Vitamin A deficiency	13220.9	2652.6	2948.4	-8.1(-8.5,-7.6)	6222.3	3137.9	3468.8	-6.9(-7.4,-6.4)	6998.6	2331.9	2604.9	-9.0(-9.5,-8.6)
Myanmar	Overall nutritional deficiencies	919257.8	1681.3	1758.5	-0.6(-0.8,-0.4)	495004.8	1744.4	1841.3	-0.7(-0.9,-0.6)	424253.0	1613.1	1675.1	-0.5(-0.8,-0.3)
	Protein-energy malnutrition	905713.3	1656.5	1734.9	-0.5(-0.7,-0.4)	485697.8	1711.6	1841.3	-0.6(-0.8,-0.5)	420015.5	1597.0	1659.7	-0.5(-0.7,-0.2)
	Iodine deficiency	13544.5	24.8	23.7	-3.8(-4.0,-3.5)	9307.0	32.8	31.7	-3.8(-4.0,-3.6)	4237.5	16.1	15.3	-3.7(-4.0,-3.5)
	Vitamin A deficiency	2536980.6	4639.9	5309.2	-6.7(-6.9,-6.5)	1316105.7	4637.9	5325.3	-6.0(-6.2,-5.9)	1220874.9	4642.1	5292.3	-7.3(-7.6,-7.1)
Philippines	Overall nutritional deficiencies	2139530.5	1907.9	1875.6	-1.0(-1.2,-0.7)	1154928.5	2089.1	2069.3	-1.1(-1.4,-0.7)	984602.0	1731.7	1678.0	-0.9(-1.1,-0.8)
	Protein-energy malnutrition	2007938.8	1790.5	1769.2	-1.1(-1.3,-0.9)	1075189.6	1944.8	2069.3	-1.2(-1.5,-0.9)	932749.2	1640.5	1595.6	-1.0(-1.2,-0.9)
	Iodine deficiency	131591.7	117.3	106.4	1.3(0.3,2.3)	79738.9	144.2	131.5	1.6(0.5,2.7)	51852.7	91.2	82.4	1.0(0.1,1.9)
	Vitamin A deficiency	9410610.2	8391.6	9309.2	-1.8(-2.2,-1.4)	4004719.3	7243.8	8034.0	-1.9(-2.3,-1.5)	5405890.9	9507.7	10529.4	-1.7(-2.2,-1.3)
Sri Lanka	Overall nutritional deficiencies	1022679.9	4679.5	5129.6	0.0(-0.3,0.4)	239251.3	2118.7	2495.4	-0.1(-0.3,0.0)	783428.6	7417.4	7909.0	0.2(-0.3,0.7)
	Protein-energy malnutrition	1013464.8	4637.3	5085.8	0.1(-0.3,0.5)	232615.0	2059.9	2495.4	0.0(-0.2,0.1)	780849.8	7392.9	7884.3	0.2(-0.3,0.7)
	Iodine deficiency	9215.1	42.2	43.8	-3.0(-3.1,-2.9)	6636.3	58.8	62.4	-2.9(-3.0,-2.8)	2578.8	24.4	24.7	-3.3(-3.4,-3.1)
	Vitamin A deficiency	655627.9	3000.0	3499.5	-5.7(-5.9,-5.5)	420741.8	3725.9	4260.1	-4.8(-5.1,-4.6)	234886.1	2223.9	2709.0	-6.7(-7.0,-6.5)
Thailand	Overall nutritional deficiencies	966960.3	1379.2	1579.9	-0.2(-0.3,-0.2)	459265.2	1279.3	1506.9	-0.2(-0.3,0.0)	507695.2	1484.0	1660.1	-0.3(-0.4,-0.3)
	Protein-energy malnutrition	950906.8	1356.3	1554.8	-0.2(-0.3,-0.1)	448584.3	1249.5	1506.9	-0.1(-0.2,0.0)	502322.5	1468.3	1643.6	-0.3(-0.3,-0.2)
	Iodine deficiency	16053.6	22.9	25.2	-2.4(-2.8,-2.1)	10680.9	29.8	33.9	-2.5(-2.9,-2.2)	5372.7	15.7	16.5	-2.2(-2.4,-1.9)
	Vitamin A deficiency	1040549.9	1484.1	1594.7	-7.4(-7.9,-7.0)	526832.6	1467.5	1583.7	-6.5(-7.0,-6.0)	513717.2	1501.6	1605.9	-8.2(-8.6,-7.7)

Timor-Leste	Overall nutritional deficiencies	40171.7	3009.5	2736.8	-1.3(-1.5,-1.2)	18037.8	2735.0	2413.1	-1.5(-1.6,-1.3)	22133.9	3277.6	3056.8	-1.2(-1.3,-1.1)
	Protein-energy malnutrition	39857.5	2986.0	2715.6	-1.3(-1.4,-1.2)	17824.3	2702.7	2413.1	-1.4(-1.6,-1.3)	22033.2	3262.7	3042.9	-1.2(-1.3,-1.1)
	Iodine deficiency	314.2	23.5	21.2	-3.1(-3.3,-2.8)	213.5	32.4	28.6	-3.1(-3.4,-2.9)	100.7	14.9	13.9	-3.1(-3.4,-2.9)
	Vitamin A deficiency	100363.6	7518.9	8083.8	-6.0(-6.4,-5.6)	47542.8	7208.8	7725.8	-5.8(-6.2,-5.4)	52820.8	7821.6	8432.1	-6.2(-6.6,-5.7)
Viet Nam	Overall nutritional deficiencies	1900367.6	1971.9	2253.9	-0.3(-0.4,-0.1)	973368.1	2001.8	2364.8	-0.2(-0.4,0.0)	926999.5	1941.4	2176.4	-0.3(-0.5,-0.2)
	Protein-energy malnutrition	1865484.8	1935.7	2218.0	-0.1(-0.3,0.1)	949799.7	1953.3	2364.8	0.0(-0.3,0.2)	915685.1	1917.7	2153.4	-0.3(-0.4,-0.1)
	Iodine deficiency	34882.8	36.2	35.9	-4.1(-4.2,-4.1)	23568.4	48.5	49.4	-4.1(-4.2,-4.0)	11314.4	23.7	23.0	-4.1(-4.2,-4.0)
	Vitamin A deficiency	1319984.9	1369.7	1674.9	-5.3(-5.6,-5.0)	673596.5	1385.3	1733.3	-5.3(-5.6,-5.1)	646388.4	1353.7	1617.4	-5.2(-5.6,-4.8)
Fiji	Overall nutritional deficiencies	11548.4	1267.3	1311.5	-0.4(-0.6,-0.2)	5643.7	1256.0	1298.4	-0.4(-0.5,-0.2)	5904.7	1278.4	1319.6	-0.4(-0.6,-0.2)
	Protein-energy malnutrition	11517.5	1263.9	1308.1	-0.4(-0.5,-0.2)	5625.2	1251.8	1298.4	-0.4(-0.5,-0.2)	5892.4	1275.7	1316.9	-0.4(-0.6,-0.2)
	Iodine deficiency	30.9	3.4	3.3	-1.8(-2.0,-1.6)	18.5	4.1	4.1	-1.6(-1.8,-1.4)	12.4	2.7	2.6	-2.1(-2.3,-1.9)
	Vitamin A deficiency	62843.8	6896.4	7759.1	-2.6(-2.8,-2.4)	15769.3	3509.3	3759.7	-3.4(-3.7,-3.1)	47074.4	10191.6	11598.6	-2.4(-2.5,-2.2)
Kiribati	Overall nutritional deficiencies	1819.8	1534.1	1463.1	-1.0(-1.1,-0.9)	927.8	1534.6	1503.1	-1.1(-1.2,-1.0)	892.0	1533.6	1414.6	-1.0(-1.1,-0.9)
	Protein-energy malnutrition	1814.1	1529.3	1458.4	-1.0(-1.1,-0.9)	924.3	1528.7	1503.1	-1.1(-1.2,-1.0)	889.8	1529.9	1410.9	-1.0(-1.1,-0.9)
	Iodine deficiency	5.7	4.8	4.7	-2.0(-2.4,-1.6)	3.6	5.9	5.7	-1.8(-2.2,-1.5)	2.2	3.7	3.7	-2.3(-2.7,-1.9)
	Vitamin A deficiency	28648.2	24151.1	26227.1	-0.1(-0.3,0.0)	10875.5	17987.4	19564.9	-0.3(-0.5,-0.2)	17772.7	30558.7	33008.9	0.0(-0.2,0.2)
Marshall Islands	Overall nutritional deficiencies	630.0	1108.3	1145.8	-0.6(-0.8,-0.5)	292.5	1054.0	1099.4	-0.8(-1.0,-0.7)	337.5	1160.2	1189.7	-0.4(-0.6,-0.3)
	Protein-energy malnutrition	627.7	1104.3	1141.9	-0.6(-0.7,-0.5)	291.1	1049.1	1099.4	-0.8(-0.9,-0.7)	336.6	1157.1	1186.7	-0.4(-0.6,-0.3)
	Iodine deficiency	2.3	4.0	3.9	-2.1(-2.5,-1.8)	1.4	4.9	4.8	-1.9(-2.2,-1.6)	0.9	3.1	3.1	-2.4(-2.8,-2.1)
	Vitamin A deficiency	12090.5	21270.3	22663.7	-2.4(-2.5,-2.4)	4508.1	16244.2	17382.7	-2.7(-2.8,-2.7)	7582.4	26065.3	27705.0	-2.3(-2.3,-2.2)
Micronesia (Federated States of)	Overall nutritional deficiencies	1088.7	1066.1	1149.3	-0.6(-0.7,-0.4)	519.1	1035.6	1119.5	-0.6(-0.7,-0.5)	569.6	1095.5	1171.0	-0.5(-0.8,-0.3)
	Protein-energy malnutrition	1084.4	1062.0	1145.3	-0.5(-0.7,-0.4)	516.5	1030.5	1119.5	-0.6(-0.7,-0.5)	567.9	1092.3	1167.9	-0.5(-0.8,-0.3)
	Iodine deficiency	4.2	4.1	4.0	-2.1(-2.4,-1.9)	2.6	5.1	4.9	-1.9(-2.2,-1.7)	1.7	3.2	3.1	-2.4(-2.7,-2.1)
	Vitamin A deficiency	34699.7	33980.8	34768.5	-1.3(-1.3,-1.2)	13500.0	26935.3	27763.6	-1.4(-1.5,-1.2)	21199.7	40772.1	41438.9	-1.2(-1.3,-1.1)
Papua New Guinea	Overall nutritional deficiencies	219204.4	2221.7	1743.5	-0.6(-0.9,-0.3)	99995.1	2100.1	1665.8	-1.3(-1.5,-1.1)	119209.3	2335.1	1815.5	0.2(-0.2,0.6)
	Protein-energy malnutrition	218813.0	2217.7	1739.5	-0.6(-0.9,-0.3)	99760.8	2095.2	1665.8	-1.3(-1.5,-1.1)	119052.2	2332.0	1812.4	0.2(-0.2,0.6)
	Iodine deficiency	391.3	4.0	3.9	-2.3(-2.6,-1.9)	234.3	4.9	4.8	-2.1(-2.4,-1.7)	157.0	3.1	3.1	-2.6(-2.9,-2.2)
	Vitamin A deficiency	1116848.0	11319.5	11735.8	-0.7(-1.1,-0.2)	482068.4	10124.5	10440.8	-0.7(-1.1,-0.3)	634779.6	12434.0	12943.6	-0.6(-1.1,-0.2)
Samoa	Overall nutritional deficiencies	2017.6	954.6	1064.3	-0.1(-0.2,0.1)	1012.0	986.4	1106.3	-0.2(-0.4,0.0)	1005.6	924.7	1021.5	0.0(-0.1,0.1)
	Protein-energy malnutrition	2011.9	951.9	1061.7	-0.1(-0.2,0.1)	1008.6	983.1	1106.3	-0.2(-0.4,0.0)	1003.2	922.5	1019.3	0.0(-0.1,0.1)
	Iodine deficiency	5.8	2.7	2.7	-1.1(-1.1,-1.1)	3.4	3.3	3.2	-0.9(-0.9,-0.9)	2.4	2.2	2.1	-1.4(-1.5,-1.3)
	Vitamin A deficiency	34085.8	16127.4	17957.8	-0.7(-0.9,-0.5)	11726.5	11429.2	12705.2	-0.7(-0.8,-0.5)	22359.4	20559.9	22880.3	-0.7(-0.8,-0.5)
Solomon Islands	Overall nutritional deficiencies	9240.9	1409.5	1267.1	-0.8(-1.0,-0.7)	4279.5	1331.8	1232.2	-1.0(-1.2,-0.9)	4961.4	1484.1	1298.4	-0.6(-0.7,-0.5)

	Protein-energy malnutrition	9216.0	1405.7	1263.3	-0.8(-1.0,-0.7)	4264.4	1327.1	1232.2	-1.0(-1.2,-0.9)	4951.6	1481.2	1295.5	-0.6(-0.7,-0.5)
	Iodine deficiency	24.9	3.8	3.8	-0.8(-1.0,-0.7)	15.1	4.7	4.6	-0.6(-0.7,-0.5)	9.8	2.9	3.0	-1.2(-1.4,-1.0)
	Vitamin A deficiency	171562.2	26167.5	27989.2	-1.2(-1.4,-1.0)	67365.8	20964.5	22321.0	-1.3(-1.5,-1.1)	104196.4	31168.6	33421.1	-1.0(-1.2,-0.8)
Tonga	Overall nutritional deficiencies	1308.3	1278.3	1279.5	-0.3(-0.5,-0.2)	674.5	1311.5	1330.2	-0.4(-0.5,-0.2)	633.8	1244.8	1220.1	-0.3(-0.4,-0.1)
	Protein-energy malnutrition	1304.6	1274.7	1275.9	-0.3(-0.5,-0.2)	672.2	1307.1	1330.2	-0.4(-0.5,-0.2)	632.4	1241.9	1217.3	-0.3(-0.4,-0.1)
	Iodine deficiency	3.7	3.6	3.6	-2.0(-2.2,-1.8)	2.3	4.4	4.4	-1.8(-2.0,-1.6)	1.4	2.8	2.8	-2.3(-2.6,-2.0)
	Vitamin A deficiency	11396.1	11134.4	12528.0	-1.7(-2.0,-1.5)	4169.7	8107.6	9102.3	-1.9(-2.1,-1.7)	7226.3	14191.4	15920.2	-1.6(-1.9,-1.4)
Vanuatu	Overall nutritional deficiencies	3657.3	1241.6	1217.1	-0.3(-0.4,-0.2)	1848.3	1272.5	1261.5	-0.5(-0.6,-0.4)	1809.0	1211.6	1174.9	-0.1(-0.2,0.0)
	Protein-energy malnutrition	3577.9	1214.7	1193.2	-0.3(-0.4,-0.2)	1798.5	1238.2	1261.5	-0.5(-0.6,-0.4)	1779.4	1191.8	1157.3	-0.1(-0.2,0.1)
	Iodine deficiency	79.4	26.9	23.9	-1.0(-1.1,-0.9)	49.8	34.3	30.0	-0.8(-0.9,-0.7)	29.6	19.8	17.6	-1.4(-1.6,-1.2)
	Vitamin A deficiency	76217.5	25875.9	28080.4	-1.2(-1.3,-1.0)	26679.0	18367.6	19812.6	-1.5(-1.6,-1.3)	49538.5	33180.4	36154.2	-1.0(-1.2,-0.8)
Armenia	Overall nutritional deficiencies	18443.6	610.8	766.5	0.0(-0.1,0.1)	10293.3	658.9	877.2	0.0(-0.1,0.1)	8150.3	559.2	666.0	0.1(-0.1,0.3)
	Protein-energy malnutrition	17490.5	579.2	725.7	0.2(0.1,0.2)	9385.8	600.8	877.2	0.3(0.2,0.4)	8104.7	556.1	662.7	0.1(-0.1,0.3)
	Iodine deficiency	953.1	31.6	40.8	-2.3(-2.8,-1.7)	907.5	58.1	82.6	-2.2(-2.7,-1.6)	45.5	3.1	3.3	-0.6(-0.7,-0.5)
	Vitamin A deficiency	15055.7	498.6	494.6	-2.8(-3.2,-2.3)	9152.7	585.9	580.8	-2.1(-2.5,-1.7)	5903.0	405.0	407.1	-3.5(-4.0,-3.1)
Azerbaijan	Overall nutritional deficiencies	65579.2	638.0	777.1	-0.9(-1.1,-0.7)	31917.6	621.4	783.2	-0.6(-0.8,-0.4)	33661.6	654.7	768.0	-1.2(-1.4,-0.9)
	Protein-energy malnutrition	64465.3	627.2	765.5	-0.9(-1.1,-0.7)	31001.0	603.5	783.2	-0.6(-0.7,-0.4)	33464.4	650.8	764.1	-1.2(-1.4,-0.9)
	Iodine deficiency	1113.9	10.8	11.6	-1.7(-2.2,-1.2)	916.6	17.8	20.1	-1.6(-2.2,-1.1)	197.2	3.8	3.9	-1.4(-1.9,-0.8)
	Vitamin A deficiency	259776.1	2527.3	2574.2	-2.2(-2.9,-1.6)	118413.1	2305.2	2325.4	-1.7(-2.3,-1.2)	141363.0	2749.3	2814.9	-2.6(-3.4,-1.8)
Georgia	Overall nutritional deficiencies	17635.9	481.2	606.2	-0.3(-0.5,0.0)	9075.6	475.3	610.3	0.0(-0.2,0.2)	8560.3	487.7	599.1	-0.6(-0.9,-0.2)
	Protein-energy malnutrition	17174.4	468.6	590.7	-0.2(-0.5,0.0)	8668.2	453.9	610.3	0.1(-0.1,0.2)	8506.2	484.6	595.8	-0.6(-0.9,-0.2)
	Iodine deficiency	461.5	12.6	15.5	-0.7(-0.8,-0.6)	407.4	21.3	28.6	-0.6(-0.7,-0.5)	54.1	3.1	3.3	-0.4(-0.5,-0.3)
	Vitamin A deficiency	82673.2	2255.9	2335.2	-0.2(-0.8,0.5)	40222.1	2106.3	2162.0	-0.1(-0.6,0.5)	42451.1	2418.6	2509.1	-0.3(-1.0,0.4)
Kazakhstan	Overall nutritional deficiencies	145794.5	792.7	808.7	0.5(0.4,0.6)	79556.7	838.9	874.1	0.6(0.5,0.7)	66237.8	743.5	744.2	0.4(0.3,0.5)
	Protein-energy malnutrition	142464.4	774.6	789.9	0.6(0.5,0.7)	76562.7	807.3	874.1	0.8(0.7,1.0)	65901.7	739.8	740.4	0.4(0.3,0.5)
	Iodine deficiency	3330.1	18.1	18.8	-2.5(-2.9,-2.2)	2994.0	31.6	34.2	-2.6(-2.9,-2.2)	336.1	3.8	3.8	-2.1(-2.4,-1.8)
	Vitamin A deficiency	1351551.7	7348.6	7612.0	-2.5(-2.6,-2.4)	383224.4	4040.9	4272.9	-2.1(-2.1,-2.0)	968327.3	10869.8	11018.8	-2.7(-2.8,-2.6)
Kyrgyzstan	Overall nutritional deficiencies	48866.2	747.7	711.0	0.0(-0.2,0.3)	24595.6	744.8	723.5	0.6(0.4,0.8)	24270.6	750.7	704.1	-0.5(-0.9,-0.1)
	Protein-energy malnutrition	47721.7	730.2	694.5	0.1(-0.2,0.3)	23568.5	713.7	723.5	0.6(0.4,0.8)	24153.2	747.1	700.6	-0.5(-0.9,-0.1)
	Iodine deficiency	1144.5	17.5	16.5	-0.3(-0.3,-0.3)	1027.1	31.1	29.8	-0.2(-0.3,-0.2)	117.4	3.6	3.5	-0.6(-0.6,-0.5)
	Vitamin A deficiency	285560.8	4369.4	4423.5	-0.6(-0.9,-0.3)	129771.9	3929.6	3958.2	-0.3(-0.6,0.0)	155788.9	4818.6	4886.6	-0.8(-1.1,-0.5)
Mongolia	Overall nutritional deficiencies	21474.9	633.9	585.4	0.0(-0.2,0.2)	12956.0	754.8	711.4	0.3(0.1,0.4)	8518.9	509.8	463.3	-0.4(-0.7,0.0)
	Protein-energy malnutrition	20908.8	617.2	568.7	0.2(0.0,0.4)	12467.8	726.3	711.4	0.6(0.4,0.7)	8441.1	505.1	458.6	-0.3(-0.7,0.0)

	Iodine deficiency	566.1	16.7	16.8	-3.5(-3.8,-3.2)	488.3	28.4	29.1	-3.6(-3.9,-3.3)	77.8	4.7	4.7	-2.6(-3.0,-2.3)
	Vitamin A deficiency	41896.2	1236.8	1242.1	-4.8(-5.0,-4.6)	19566.7	1139.9	1145.4	-3.8(-3.9,-3.7)	22329.6	1336.3	1339.2	-5.5(-5.8,-5.3)
Tajikistan	Overall nutritional deficiencies	109204.2	1150.4	966.5	-0.1(-0.2,0.0)	57087.4	1216.3	1034.1	-0.2(-0.4,-0.1)	52116.8	1086.0	901.7	-0.1(-0.2,0.0)
	Protein-energy malnutrition	106154.1	1118.3	938.0	-0.1(-0.2,0.0)	54323.8	1157.4	1034.1	-0.1(-0.3,0.0)	51830.3	1080.0	896.1	-0.1(-0.1,0.0)
	Iodine deficiency	3050.1	32.1	28.5	-1.3(-2.1,-0.5)	2763.6	58.9	52.6	-1.3(-2.1,-0.5)	286.5	6.0	5.6	-1.0(-1.8,-0.2)
	Vitamin A deficiency	766523.5	8075.1	8084.8	-1.7(-2.2,-1.1)	344661.6	7343.3	7381.8	-1.1(-1.6,-0.6)	421861.8	8790.8	8765.7	-2.1(-2.7,-1.5)
Turkmenistan	Overall nutritional deficiencies	45313.3	891.5	849.4	-0.2(-0.3,-0.1)	22585.4	908.1	871.3	-0.2(-0.3,0.0)	22727.8	875.5	830.4	-0.2(-0.3,-0.1)
	Protein-energy malnutrition	44509.8	875.6	833.9	-0.2(-0.3,-0.1)	21867.9	879.2	871.3	-0.2(-0.3,0.0)	22641.9	872.2	827.1	-0.2(-0.3,-0.1)
	Iodine deficiency	803.5	15.8	15.5	-0.8(-0.9,-0.7)	717.5	28.8	28.7	-0.7(-0.8,-0.6)	86.0	3.3	3.3	-0.5(-0.6,-0.4)
	Vitamin A deficiency	113808.9	2239.0	2273.9	-3.4(-3.8,-3.1)	51579.6	2073.8	2092.9	-2.9(-3.2,-2.6)	62229.3	2397.2	2442.2	-3.9(-4.2,-3.5)
Uzbekistan	Overall nutritional deficiencies	329885.7	979.6	939.2	-0.4(-0.5,-0.2)	167405.9	992.4	978.6	-0.5(-0.6,-0.3)	162479.8	966.7	903.9	-0.2(-0.3,-0.1)
	Protein-energy malnutrition	320752.4	952.4	913.6	-0.3(-0.4,-0.1)	159155.2	943.5	978.6	-0.3(-0.4,-0.2)	161597.2	961.4	898.8	-0.2(-0.3,-0.1)
	Iodine deficiency	9133.3	27.1	25.6	-2.7(-3.1,-2.3)	8250.6	48.9	47.1	-2.7(-3.1,-2.3)	882.7	5.3	5.0	-2.3(-2.7,-1.9)
	Vitamin A deficiency	992648.4	2947.5	2979.1	-3.4(-3.6,-3.1)	478616.0	2837.3	2857.4	-2.6(-2.8,-2.4)	514032.4	3058.2	3098.9	-4.0(-4.2,-3.7)
Albania	Overall nutritional deficiencies	45929.0	1688.3	2096.0	0.2(0.1,0.4)	27463.1	2022.4	2573.4	-0.4(-0.7,0.0)	18466.0	1355.4	1648.3	1.1(0.8,1.4)
	Protein-energy malnutrition	45674.3	1679.0	2085.2	0.2(0.1,0.4)	27303.9	2010.7	2573.4	-0.3(-0.7,0.0)	18370.4	1348.4	1640.3	1.1(0.8,1.4)
	Iodine deficiency	254.7	9.4	10.8	-1.9(-2.0,-1.7)	159.1	11.7	13.7	-1.8(-2.0,-1.7)	95.6	7.0	8.0	-2.0(-2.1,-1.8)
	Vitamin A deficiency	304737.7	11202.1	11731.9	-3.5(-3.7,-3.2)	203278.5	14969.6	15056.4	-2.8(-3.0,-2.6)	101459.2	7447.1	8519.1	-4.5(-4.8,-4.2)
Bosnia and Herzegovina	Overall nutritional deficiencies	25549.6	774.2	1015.8	0.4(0.3,0.5)	12811.3	758.0	1041.2	0.3(0.2,0.4)	12738.3	791.3	994.8	0.6(0.5,0.6)
	Protein-energy malnutrition	25056.1	759.3	996.2	0.5(0.4,0.6)	12482.8	738.5	1041.2	0.4(0.3,0.5)	12573.3	781.1	982.0	0.6(0.5,0.7)
	Iodine deficiency	493.5	15.0	19.6	-2.6(-3.1,-2.2)	328.4	19.4	26.7	-2.5(-3.0,-2.1)	165.0	10.3	12.8	-2.8(-3.2,-2.4)
	Vitamin A deficiency	281442.4	8528.6	8900.1	-3.8(-4.1,-3.5)	202166.4	11961.0	12077.6	-3.2(-3.4,-3.0)	79276.0	4924.7	5716.3	-4.8(-5.1,-4.5)
Bulgaria	Overall nutritional deficiencies	48088.3	693.5	960.3	0.6(0.4,0.7)	23551.2	660.0	958.7	0.4(0.3,0.5)	24537.1	728.9	964.5	0.7(0.6,0.9)
	Protein-energy malnutrition	47614.3	686.6	951.9	0.6(0.5,0.7)	23206.1	650.4	958.7	0.4(0.3,0.5)	24408.2	725.0	960.2	0.7(0.6,0.9)
	Iodine deficiency	474.0	6.8	8.4	-1.4(-1.5,-1.4)	345.1	9.7	12.7	-1.4(-1.5,-1.3)	128.9	3.8	4.3	-1.5(-1.6,-1.4)
	Vitamin A deficiency	549143.1	7918.9	8299.1	-1.8(-2.0,-1.5)	387384.5	10856.8	10981.9	-1.5(-1.7,-1.3)	161758.6	4805.0	5652.2	-2.3(-2.6,-1.9)
North Macedonia	Overall nutritional deficiencies	20181.4	937.5	1208.0	0.8(0.6,0.9)	11614.6	1096.2	1442.8	0.6(0.4,0.8)	8566.9	783.7	988.3	1.1(1.0,1.2)
	Protein-energy malnutrition	19981.5	928.2	1197.1	0.8(0.6,0.9)	11490.0	1084.4	1442.8	0.6(0.4,0.8)	8491.5	776.8	980.2	1.1(1.0,1.2)
	Iodine deficiency	199.9	9.3	10.9	-1.2(-1.3,-1.1)	124.5	11.8	13.9	-1.2(-1.3,-1.1)	75.4	6.9	8.1	-1.3(-1.4,-1.2)
	Vitamin A deficiency	265633.8	12339.4	12794.1	-2.7(-2.9,-2.6)	130680.6	12333.6	12575.0	-2.3(-2.5,-2.2)	134953.3	12345.0	13001.2	-3.1(-3.3,-2.9)
Montenegro	Overall nutritional deficiencies	6412.1	1033.6	1264.6	1.0(0.9,1.2)	3323.4	1059.3	1399.0	0.9(0.7,1.1)	3088.7	1007.3	1148.7	1.2(1.0,1.3)
	Protein-energy malnutrition	6354.7	1024.4	1254.0	1.0(0.9,1.2)	3287.5	1047.9	1399.0	0.9(0.8,1.1)	3067.2	1000.4	1140.8	1.2(1.1,1.3)
	Iodine deficiency	57.4	9.2	10.6	-1.2(-1.3,-1.1)	35.9	11.4	13.6	-1.1(-1.2,-1.0)	21.5	7.0	7.8	-1.2(-1.3,-1.1)

	Vitamin A deficiency	37826.7	6097.7	6333.4	-2.0(-2.5,-1.5)	27075.9	8630.5	8694.5	-1.6(-2.0,-1.2)	10750.8	3506.3	4021.2	-2.7(-3.3,-2.1)
Serbia	Overall nutritional deficiencies	76114.8	870.2	1101.2	0.5(0.4,0.6)	35050.3	795.0	1001.0	0.5(0.4,0.6)	41064.5	946.6	1194.3	0.4(0.3,0.5)
	Protein-energy malnutrition	75491.7	863.1	1092.9	0.5(0.4,0.6)	34735.5	787.9	1001.0	0.5(0.4,0.6)	40756.2	939.5	1186.2	0.4(0.3,0.5)
	Iodine deficiency	623.1	7.1	8.3	-1.4(-1.5,-1.3)	314.7	7.1	8.4	-1.4(-1.6,-1.3)	308.3	7.1	8.1	-1.4(-1.5,-1.3)
	Vitamin A deficiency	1333724.2	15248.2	15871.8	-2.4(-2.6,-2.2)	621905.4	14106.3	14466.6	-2.3(-2.5,-2.1)	711818.8	16408.6	17233.2	-2.4(-2.7,-2.2)
Belarus	Overall nutritional deficiencies	53576.8	563.9	772.8	-0.2(-0.3,-0.1)	23120.2	455.8	677.1	-0.6(-0.7,-0.5)	30456.6	687.8	872.1	0.1(0.0,0.2)
	Protein-energy malnutrition	53003.3	557.9	765.5	-0.2(-0.3,-0.1)	22722.4	447.9	677.1	-0.6(-0.7,-0.5)	30280.9	683.8	867.8	0.1(0.0,0.2)
	Iodine deficiency	573.4	6.0	7.3	-1.0(-1.3,-0.7)	397.8	7.8	10.2	-1.0(-1.3,-0.7)	175.7	4.0	4.4	-1.0(-1.3,-0.6)
	Vitamin A deficiency	121017.0	1273.8	1287.0	-3.6(-3.8,-3.3)	85107.7	1677.8	1689.9	-2.9(-3.1,-2.6)	35909.3	810.9	866.7	-4.7(-4.9,-4.5)
Republic of Moldova	Overall nutritional deficiencies	20763.2	563.0	858.4	0.6(0.5,0.7)	9798.0	507.9	851.0	0.6(0.5,0.7)	10965.2	623.3	871.1	0.6(0.5,0.7)
	Protein-energy malnutrition	20604.8	558.7	853.4	0.6(0.5,0.7)	9696.3	502.6	851.0	0.6(0.5,0.7)	10908.5	620.1	867.5	0.6(0.5,0.7)
	Iodine deficiency	158.4	4.3	5.0	0.4(-0.2,1.0)	101.7	5.3	6.5	0.4(-0.1,1.0)	56.7	3.2	3.6	0.4(-0.2,1.0)
	Vitamin A deficiency	67366.8	1826.6	1858.0	-2.4(-2.8,-2.0)	45437.9	2355.4	2373.6	-1.8(-2.2,-1.4)	21928.9	1246.6	1330.2	-3.3(-3.6,-2.9)
Russian Federation	Overall nutritional deficiencies	1106361.6	754.1	997.5	0.2(0.0,0.3)	528306.1	673.8	959.8	0.2(0.0,0.3)	578055.5	846.2	1041.4	0.2(0.0,0.3)
	Protein-energy malnutrition	1095257.8	746.5	988.4	0.2(0.0,0.3)	519916.2	663.1	959.8	0.2(0.0,0.3)	575341.6	842.2	1037.1	0.2(0.0,0.3)
	Iodine deficiency	11103.8	7.6	9.1	-0.3(-0.7,0.1)	8389.9	10.7	13.9	-0.3(-0.7,0.1)	2713.9	4.0	4.3	-0.3(-0.6,0.1)
	Vitamin A deficiency	304598.5	207.6	199.5	-2.5(-2.6,-2.4)	190571.5	243.1	229.6	-2.2(-2.3,-2.1)	114027.0	166.9	167.8	-2.8(-2.9,-2.7)
Ukraine	Overall nutritional deficiencies	325783.2	739.7	1159.1	0.3(0.2,0.5)	146347.7	616.1	1062.6	0.4(0.2,0.6)	179435.5	884.4	1258.7	0.3(0.1,0.4)
	Protein-energy malnutrition	317673.7	721.3	1131.7	0.3(0.2,0.5)	140646.6	592.1	1062.6	0.4(0.2,0.6)	177027.1	872.5	1243.2	0.3(0.1,0.4)
	Iodine deficiency	8109.5	18.4	27.4	-0.1(-0.6,0.4)	5701.2	24.0	39.9	0.0(-0.5,0.5)	2408.3	11.9	15.5	-0.2(-0.7,0.4)
	Vitamin A deficiency	589962.8	1339.5	1357.4	-1.7(-1.9,-1.5)	420498.3	1770.3	1792.4	-1.2(-1.4,-1.0)	169464.6	835.2	894.3	-2.5(-2.7,-2.4)
Argentina	Overall nutritional deficiencies	454896.5	1008.3	1020.4	0.1(-0.1,0.3)	201462.0	871.2	890.4	0.2(-0.1,0.4)	253434.5	1152.5	1164.1	0.1(-0.1,0.2)
	Protein-energy malnutrition	451318.1	1000.4	1012.1	0.1(-0.1,0.3)	199511.0	862.7	890.4	0.2(-0.1,0.4)	251807.1	1145.1	1156.4	0.1(-0.1,0.3)
	Iodine deficiency	3578.4	7.9	8.3	-0.6(-0.6,-0.5)	1951.0	8.4	9.0	-0.5(-0.6,-0.5)	1627.4	7.4	7.6	-0.6(-0.6,-0.6)
	Vitamin A deficiency	3372876.5	7476.1	7788.9	-1.2(-1.4,-1.0)	1440201.8	6227.9	6513.5	-1.1(-1.4,-0.9)	1932674.8	8788.8	9085.5	-1.2(-1.5,-1.0)
Belize	Overall nutritional deficiencies	2654.6	647.3	698.2	-1.6(-1.7,-1.4)	1527.3	741.4	802.1	-1.6(-1.7,-1.4)	1127.4	552.4	594.6	-1.5(-1.7,-1.4)
	Protein-energy malnutrition	2628.6	641.0	692.2	-1.6(-1.7,-1.4)	1512.7	734.3	802.1	-1.6(-1.7,-1.4)	1115.9	546.7	589.3	-1.5(-1.7,-1.4)
	Iodine deficiency	26.0	6.3	6.0	-1.5(-1.6,-1.5)	14.6	7.1	6.6	-1.6(-1.6,-1.5)	11.5	5.6	5.3	-1.5(-1.6,-1.4)
	Vitamin A deficiency	18856.4	4598.1	4673.4	-3.3(-3.4,-3.1)	9575.2	4648.3	4681.5	-2.9(-3.1,-2.8)	9281.2	4547.4	4665.2	-3.6(-3.8,-3.4)
Cuba	Overall nutritional deficiencies	88689.0	780.8	918.2	0.3(0.0,0.5)	44408.2	778.2	960.3	0.4(0.2,0.7)	44280.8	783.5	880.9	0.1(-0.1,0.4)
	Protein-energy malnutrition	87060.7	766.5	901.1	0.4(0.1,0.6)	43513.4	762.5	960.3	0.5(0.3,0.8)	43547.4	770.5	865.9	0.2(-0.1,0.5)
	Iodine deficiency	1628.3	14.3	17.1	-2.2(-2.7,-1.6)	894.9	15.7	19.4	-2.2(-2.7,-1.6)	733.4	13.0	15.0	-2.1(-2.6,-1.6)
	Vitamin A deficiency	252109.4	2219.6	2264.9	-2.1(-2.3,-1.9)	130466.0	2286.2	2305.7	-1.8(-2.0,-1.7)	121643.5	2152.3	2225.0	-2.3(-2.6,-2.1)

Dominica	Overall nutritional deficiencies	667.5	971.9	1067.3	-0.5(-0.6,-0.3)	324.2	964.1	1063.4	-0.4(-0.6,-0.3)	343.3	979.4	1071.3	-0.5(-0.7,-0.4)
	Protein-energy malnutrition	657.7	957.7	1052.3	-0.4(-0.6,-0.3)	318.9	948.2	1063.4	-0.4(-0.5,-0.2)	338.9	966.8	1058.1	-0.5(-0.6,-0.4)
	Iodine deficiency	9.8	14.2	15.0	-2.5(-2.7,-2.3)	5.4	15.9	17.0	-2.5(-2.7,-2.4)	4.4	12.6	13.2	-2.5(-2.7,-2.3)
	Vitamin A deficiency	1392.1	2026.8	2129.1	-3.6(-3.9,-3.3)	775.1	2304.8	2394.8	-3.1(-3.4,-2.9)	617.0	1760.1	1877.0	-4.0(-4.4,-3.7)
Dominican Republic	Overall nutritional deficiencies	63031.2	579.2	585.5	-1.6(-1.8,-1.5)	28803.7	532.4	539.6	-1.7(-1.8,-1.5)	34227.5	625.6	631.0	-1.6(-1.7,-1.5)
	Protein-energy malnutrition	61011.6	560.7	567.2	-1.6(-1.7,-1.5)	27678.8	511.6	539.6	-1.7(-1.8,-1.5)	33332.8	609.2	615.0	-1.6(-1.7,-1.4)
	Iodine deficiency	2019.6	18.6	18.2	-2.4(-2.5,-2.3)	1124.9	20.8	20.5	-2.4(-2.6,-2.3)	894.7	16.4	16.0	-2.4(-2.5,-2.3)
	Vitamin A deficiency	517319.8	4754.0	4835.1	-4.4(-4.7,-4.0)	264747.7	4893.3	4971.3	-3.8(-4.2,-3.5)	252572.1	4616.2	4701.8	-4.9(-5.2,-4.5)
Grenada	Overall nutritional deficiencies	1036.1	1003.8	1076.6	-0.3(-0.4,-0.2)	410.9	815.5	898.6	-0.3(-0.5,-0.2)	625.2	1183.4	1258.2	-0.3(-0.5,-0.2)
	Protein-energy malnutrition	1023.4	991.5	1064.0	-0.3(-0.4,-0.1)	404.0	801.9	898.6	-0.2(-0.4,-0.1)	619.4	1172.4	1247.1	-0.3(-0.4,-0.2)
	Iodine deficiency	12.7	12.3	12.6	-3.2(-3.3,-3.1)	6.9	13.6	14.2	-3.2(-3.4,-3.1)	5.8	11.0	11.1	-3.1(-3.3,-3.0)
	Vitamin A deficiency	3584.4	3472.8	3614.9	-3.6(-4.0,-3.3)	1817.6	3607.6	3722.0	-3.2(-3.5,-2.9)	1766.8	3344.2	3514.7	-4.1(-4.5,-3.7)
Guyana	Overall nutritional deficiencies	8209.9	1065.2	1131.3	-1.9(-2.1,-1.8)	3872.7	994.7	1068.2	-2.1(-2.3,-1.9)	4337.2	1137.3	1198.7	-1.8(-1.9,-1.6)
	Protein-energy malnutrition	8023.9	1041.1	1108.0	-1.9(-2.1,-1.8)	3768.1	967.8	1068.2	-2.1(-2.3,-1.9)	4255.8	1116.0	1178.3	-1.8(-1.9,-1.6)
	Iodine deficiency	186.0	24.1	23.3	-2.1(-2.3,-2.0)	104.6	26.9	26.3	-2.1(-2.3,-2.0)	81.4	21.3	20.4	-2.1(-2.2,-1.9)
	Vitamin A deficiency	37096.3	4813.3	4953.9	-3.3(-3.4,-3.1)	18668.7	4794.9	4897.4	-2.8(-2.9,-2.7)	18427.6	4832.1	5010.9	-3.7(-3.8,-3.5)
Haiti	Overall nutritional deficiencies	148567.1	1197.9	1083.5	-1.2(-1.3,-1.0)	75230.4	1177.8	1084.8	-1.0(-1.2,-0.9)	73336.7	1219.3	1085.6	-1.3(-1.5,-1.2)
	Protein-energy malnutrition	141523.5	1141.1	1034.1	-1.2(-1.3,-1.1)	71201.9	1114.7	1084.8	-1.0(-1.2,-0.9)	70321.6	1169.2	1042.5	-1.4(-1.5,-1.2)
	Iodine deficiency	7043.6	56.8	49.4	-0.9(-1.2,-0.7)	4028.5	63.1	55.8	-1.0(-1.3,-0.7)	3015.1	50.1	43.0	-0.9(-1.2,-0.6)
	Vitamin A deficiency	1668645.6	13454.5	13512.7	-2.4(-2.5,-2.4)	797415.4	12483.9	12558.7	-2.1(-2.2,-2.1)	871230.2	14485.4	14516.8	-2.6(-2.7,-2.6)
Jamaica	Overall nutritional deficiencies	20921.8	744.3	837.1	-1.9(-2.1,-1.7)	9747.7	688.9	793.7	-2.1(-2.3,-1.9)	11174.1	800.6	883.6	-1.7(-1.9,-1.6)
	Protein-energy malnutrition	20441.6	727.3	819.7	-1.9(-2.1,-1.7)	9478.9	669.9	793.7	-2.1(-2.3,-1.9)	10962.7	785.5	868.4	-1.7(-1.9,-1.6)
	Iodine deficiency	480.2	17.1	17.3	-1.8(-2.0,-1.7)	268.8	19.0	19.5	-1.9(-2.0,-1.7)	211.4	15.1	15.2	-1.8(-1.9,-1.7)
	Vitamin A deficiency	67629.4	2406.1	2504.5	-3.3(-3.4,-3.1)	38672.5	2732.9	2822.8	-2.8(-3.0,-2.7)	28957.0	2074.7	2185.9	-3.7(-3.9,-3.5)
Saint Lucia	Overall nutritional deficiencies	1532.1	877.3	967.4	-0.7(-0.8,-0.5)	709.8	808.6	886.7	-0.7(-0.9,-0.6)	822.2	946.8	1049.2	-0.6(-0.8,-0.5)
	Protein-energy malnutrition	1519.6	870.2	959.9	-0.7(-0.8,-0.5)	702.9	800.7	886.7	-0.7(-0.9,-0.6)	816.7	940.4	1042.6	-0.6(-0.8,-0.5)
	Iodine deficiency	12.5	7.2	7.5	-1.4(-1.4,-1.3)	6.9	7.9	8.4	-1.4(-1.4,-1.3)	5.6	6.4	6.6	-1.3(-1.4,-1.3)
	Vitamin A deficiency	5022.3	2876.1	3007.2	-2.8(-3.0,-2.6)	2760.3	3144.4	3257.6	-2.6(-2.8,-2.4)	2262.0	2604.8	2758.7	-3.1(-3.3,-2.9)
Saint Vincent and the Grenadines	Overall nutritional deficiencies	1160.2	1025.4	1095.8	-1.0(-1.1,-0.9)	515.6	932.5	1021.1	-0.7(-0.8,-0.5)	644.6	1114.2	1167.1	-1.3(-1.5,-1.2)
	Protein-energy malnutrition	1144.9	1011.9	1081.6	-1.0(-1.1,-0.8)	507.2	917.3	1021.1	-0.6(-0.8,-0.5)	637.7	1102.2	1154.7	-1.3(-1.4,-1.2)
	Iodine deficiency	15.3	13.5	14.2	-2.9(-3.1,-2.8)	8.4	15.2	16.0	-2.9(-3.1,-2.8)	6.9	11.9	12.5	-2.9(-3.0,-2.7)
	Vitamin A deficiency	4313.9	3812.8	3982.8	-3.7(-3.9,-3.5)	2228.0	4030.1	4157.2	-3.1(-3.2,-2.9)	2085.9	3605.1	3815.7	-4.2(-4.5,-4.0)
Suriname	Overall nutritional deficiencies	5583.0	969.5	1062.0	-0.7(-0.8,-0.5)	2907.4	998.9	1111.2	-0.5(-0.6,-0.3)	2675.6	939.4	1016.4	-0.9(-1.0,-0.8)

	Protein-energy malnutrition	5510.2	956.8	1049.2	-0.6(-0.8,-0.5)	2866.6	984.9	1111.2	-0.4(-0.6,-0.2)	2643.6	928.2	1005.2	-0.9(-1.0,-0.7)
	Iodine deficiency	72.8	12.6	12.8	-2.8(-3.2,-2.5)	40.8	14.0	14.4	-2.8(-3.2,-2.5)	32.1	11.3	11.2	-2.8(-3.2,-2.4)
	Vitamin A deficiency	23893.4	4149.0	4317.3	-3.2(-3.3,-3.0)	12109.4	4160.4	4288.5	-2.8(-2.9,-2.6)	11784.0	4137.3	4346.0	-3.5(-3.7,-3.3)
Bolivia (Plurinational State of)	Overall nutritional deficiencies	94958.5	790.5	783.2	-0.7(-0.8,-0.5)	37881.3	631.4	619.6	-1.5(-1.6,-1.3)	57077.3	949.3	949.8	0.0(-0.2,0.2)
	Protein-energy malnutrition	94322.4	785.3	777.9	-0.7(-0.8,-0.5)	37536.0	625.7	619.6	-1.5(-1.6,-1.3)	56786.4	944.5	945.1	0.0(-0.2,0.2)
	Iodine deficiency	636.1	5.3	5.2	-0.9(-0.9,-0.8)	345.3	5.8	5.7	-0.9(-0.9,-0.8)	290.9	4.8	4.8	-0.9(-0.9,-0.8)
	Vitamin A deficiency	960652.6	7997.6	8475.2	-1.6(-1.8,-1.4)	499854.1	8332.1	8613.3	-1.6(-1.8,-1.5)	460798.4	7663.9	8338.8	-1.6(-1.9,-1.3)
Ecuador	Overall nutritional deficiencies	124255.3	706.5	731.9	-1.1(-1.3,-0.9)	63952.1	724.1	748.1	-1.2(-1.4,-1.0)	60303.1	688.7	714.3	-0.9(-1.2,-0.7)
	Protein-energy malnutrition	123406.1	701.6	727.2	-1.1(-1.3,-0.9)	63490.2	718.9	748.1	-1.2(-1.4,-1.0)	59915.9	684.3	709.9	-0.9(-1.2,-0.7)
	Iodine deficiency	849.1	4.8	4.7	-0.5(-0.5,-0.4)	461.9	5.2	5.2	-0.5(-0.5,-0.4)	387.2	4.4	4.3	-0.5(-0.5,-0.4)
	Vitamin A deficiency	583168.8	3315.6	3770.8	-2.7(-3.0,-2.4)	326682.0	3698.8	4138.7	-2.3(-2.6,-2.0)	256486.8	2929.2	3404.0	-3.2(-3.5,-2.8)
Peru	Overall nutritional deficiencies	158411.3	466.0	473.2	-1.6(-1.7,-1.4)	90104.2	531.2	532.8	-1.7(-2.0,-1.5)	68307.1	401.0	411.7	-1.3(-1.5,-1.2)
	Protein-energy malnutrition	156820.9	461.3	468.5	-1.6(-1.8,-1.4)	89246.8	526.2	532.8	-1.7(-2.0,-1.5)	67574.0	396.7	407.4	-1.4(-1.5,-1.2)
	Iodine deficiency	1590.5	4.7	4.6	-0.6(-0.7,-0.5)	857.4	5.1	5.1	-0.6(-0.7,-0.5)	733.1	4.3	4.3	-0.6(-0.6,-0.5)
	Vitamin A deficiency	1996094.7	5871.7	6087.7	-3.2(-3.3,-3.0)	935389.5	5514.6	5698.4	-2.8(-2.9,-2.7)	1060705.3	6227.2	6469.0	-3.5(-3.7,-3.3)
Colombia	Overall nutritional deficiencies	324037.7	678.2	707.8	-0.7(-0.9,-0.5)	182771.6	748.2	795.3	-0.7(-0.9,-0.5)	141266.1	605.0	623.5	-0.7(-0.9,-0.5)
	Protein-energy malnutrition	313165.8	655.5	683.3	-0.7(-1.0,-0.5)	176392.0	722.1	795.3	-0.7(-1.0,-0.5)	136773.7	585.8	603.4	-0.7(-0.9,-0.5)
	Iodine deficiency	10871.9	22.8	24.6	-0.4(-0.5,-0.3)	6379.5	26.1	29.2	-0.4(-0.5,-0.3)	4492.4	19.2	20.1	-0.3(-0.4,-0.2)
	Vitamin A deficiency	1391264.6	2912.0	2925.5	-4.0(-4.1,-3.9)	696931.5	2853.2	2860.0	-3.7(-3.8,-3.6)	694333.0	2973.6	2992.2	-4.2(-4.3,-4.1)
Costa Rica	Overall nutritional deficiencies	44960.9	953.2	997.0	0.0(-0.3,0.2)	20630.6	848.3	922.6	0.0(-0.2,0.2)	24330.3	1064.9	1085.6	0.0(-0.3,0.4)
	Protein-energy malnutrition	43960.3	932.0	974.1	0.0(-0.3,0.3)	20037.4	823.9	922.6	0.0(-0.2,0.2)	23922.9	1047.1	1066.8	0.1(-0.3,0.4)
	Iodine deficiency	1000.6	21.2	22.9	-0.4(-0.5,-0.3)	593.2	24.4	27.1	-0.4(-0.5,-0.4)	407.4	17.8	18.7	-0.4(-0.5,-0.3)
	Vitamin A deficiency	131698.9	2792.2	2864.1	-3.3(-3.4,-3.2)	66484.4	2733.7	2800.8	-3.0(-3.1,-2.9)	65214.5	2854.4	2930.2	-3.5(-3.6,-3.4)
El Salvador	Overall nutritional deficiencies	52395.2	837.5	869.7	-0.2(-0.3,0.0)	30849.2	927.8	957.2	-0.1(-0.2,0.1)	21545.9	735.1	773.5	-0.3(-0.5,-0.2)
	Protein-energy malnutrition	50703.5	810.5	842.9	-0.2(-0.3,0.0)	29833.1	897.2	957.2	-0.1(-0.2,0.1)	20870.5	712.0	751.6	-0.3(-0.5,-0.1)
	Iodine deficiency	1691.6	27.0	26.8	-0.8(-0.9,-0.7)	1016.2	30.6	31.7	-0.8(-0.9,-0.7)	675.4	23.0	21.8	-0.7(-0.8,-0.7)
	Vitamin A deficiency	355173.3	5677.2	5828.6	-4.7(-5.0,-4.3)	173752.4	5225.7	5375.0	-4.2(-4.5,-3.8)	181420.9	6189.4	6324.2	-5.0(-5.4,-4.7)
Guatemala	Overall nutritional deficiencies	150314.7	845.6	909.4	-2.5(-2.6,-2.3)	83530.9	914.4	966.9	-2.9(-3.1,-2.7)	66783.8	772.8	852.3	-1.9(-2.0,-1.7)
	Protein-energy malnutrition	145103.9	816.3	883.2	-2.5(-2.7,-2.4)	80427.3	880.4	966.9	-3.0(-3.2,-2.8)	64676.6	748.5	830.8	-1.9(-2.1,-1.7)
	Iodine deficiency	5210.8	29.3	26.2	-0.1(-0.2,0.0)	3103.6	34.0	31.0	-0.1(-0.2,0.0)	2107.2	24.4	21.4	-0.1(-0.1,0.0)
	Vitamin A deficiency	1051749.7	5916.5	5880.1	-3.9(-4.1,-3.8)	529418.3	5795.4	5772.2	-3.7(-3.8,-3.5)	522331.5	6044.6	5991.7	-4.2(-4.4,-4.0)
Honduras	Overall nutritional deficiencies	75702.6	771.3	825.0	-1.3(-1.5,-1.2)	43825.6	870.2	932.4	-1.4(-1.6,-1.2)	31877.0	667.2	710.4	-1.3(-1.5,-1.2)
	Protein-energy malnutrition	72116.4	734.8	792.9	-1.4(-1.6,-1.2)	41683.4	827.6	932.4	-1.4(-1.6,-1.2)	30433.0	636.9	684.2	-1.4(-1.5,-1.2)

	Iodine deficiency	3586.2	36.5	32.1	-0.4(-0.6,-0.2)	2142.2	42.5	38.1	-0.4(-0.6,-0.3)	1443.9	30.2	26.2	-0.4(-0.5,-0.2)
	Vitamin A deficiency	585316.0	5963.9	5901.6	-3.1(-3.3,-2.8)	297399.0	5905.0	5836.6	-2.7(-2.9,-2.5)	287917.0	6025.9	5969.3	-3.4(-3.6,-3.1)
Mexico	Overall nutritional deficiencies	1971471.9	1577.9	1625.4	-1.0(-1.3,-0.7)	932606.3	1461.1	1510.9	-1.2(-1.3,-1.1)	1038865.6	1699.9	1752.8	-0.7(-1.3,-0.1)
	Protein-energy malnutrition	1923660.3	1539.7	1586.2	-1.0(-1.3,-0.7)	898023.0	1406.9	1510.9	-1.3(-1.4,-1.1)	1025637.3	1678.3	1731.3	-0.7(-1.3,-0.1)
	Iodine deficiency	47811.6	38.3	39.2	0.0(0.0,0.1)	34583.3	54.2	57.2	0.1(0.0,0.1)	13228.3	21.6	21.5	0.1(0.0,0.1)
	Vitamin A deficiency	8293913.1	6638.3	7181.9	-3.2(-3.3,-3.2)	3196430.4	5007.9	5405.8	-3.1(-3.2,-3.0)	5097482.7	8341.2	9004.3	-3.3(-3.4,-3.2)
Nicaragua	Overall nutritional deficiencies	38886.4	597.3	617.7	-1.5(-1.8,-1.3)	18034.5	546.9	568.4	-1.6(-1.9,-1.4)	20851.9	649.0	672.9	-1.5(-1.7,-1.2)
	Protein-energy malnutrition	36663.9	563.2	586.2	-1.6(-1.9,-1.3)	16724.0	507.2	568.4	-1.7(-2.0,-1.5)	19940.0	620.6	647.1	-1.5(-1.8,-1.3)
	Iodine deficiency	2222.4	34.1	31.5	-0.4(-0.7,-0.2)	1310.5	39.7	37.4	-0.4(-0.7,-0.2)	911.9	28.4	25.7	-0.4(-0.7,-0.2)
	Vitamin A deficiency	119161.6	1830.3	1750.3	-6.7(-7.3,-6.2)	61953.6	1878.9	1769.8	-6.0(-6.5,-5.6)	57208.1	1780.5	1730.7	-7.3(-7.9,-6.8)
Venezuela (Bolivarian Republic of)	Overall nutritional deficiencies	288944.8	1029.4	1087.3	-0.4(-0.7,-0.1)	120073.8	840.0	918.2	-0.3(-0.5,0.0)	168871.0	1226.0	1268.9	-0.5(-0.8,-0.2)
	Protein-energy malnutrition	282326.9	1005.8	1062.7	-0.4(-0.7,-0.1)	116179.6	812.8	918.2	-0.3(-0.5,0.0)	166147.4	1206.2	1248.7	-0.5(-0.8,-0.2)
	Iodine deficiency	6617.9	23.6	24.6	0.0(-0.1,0.2)	3894.2	27.2	29.1	0.0(-0.1,0.2)	2723.6	19.8	20.2	0.1(0.0,0.2)
	Vitamin A deficiency	1500266.1	5344.9	5461.0	-1.3(-1.9,-0.8)	659594.5	4614.4	4710.6	-1.1(-1.6,-0.6)	840671.5	6103.1	6227.3	-1.5(-2.0,-0.9)
Brazil	Overall nutritional deficiencies	1379151.0	636.5	693.1	-1.4(-1.6,-1.1)	689623.9	622.0	690.5	-1.3(-1.5,-1.0)	689527.1	651.7	699.8	-1.4(-1.7,-1.1)
	Protein-energy malnutrition	1365700.0	630.3	686.7	-1.4(-1.6,-1.1)	681954.9	615.1	690.5	-1.3(-1.6,-1.1)	683745.1	646.3	694.3	-1.4(-1.7,-1.2)
	Iodine deficiency	13451.0	6.2	6.3	-0.4(-0.5,-0.4)	7669.0	6.9	7.2	-0.3(-0.4,-0.3)	5782.0	5.5	5.5	-0.5(-0.6,-0.5)
	Vitamin A deficiency	21512083.7	9928.7	10128.6	-3.2(-3.3,-3.1)	9934895.1	8961.2	9187.5	-3.0(-3.1,-2.9)	11577188.5	10942.6	11088.7	-3.3(-3.4,-3.1)
Paraguay	Overall nutritional deficiencies	60787.3	877.1	937.1	1.0(0.8,1.1)	32397.1	946.4	1002.8	1.1(0.9,1.2)	28390.2	809.5	871.9	0.9(0.8,1.1)
	Protein-energy malnutrition	60294.7	870.0	930.2	1.0(0.9,1.2)	32124.2	938.4	1002.8	1.1(0.9,1.2)	28170.5	803.2	865.8	0.9(0.8,1.1)
	Iodine deficiency	492.6	7.1	6.9	-0.9(-1.0,-0.8)	272.9	8.0	7.7	-0.9(-0.9,-0.8)	219.8	6.3	6.1	-1.0(-1.0,-0.9)
	Vitamin A deficiency	421006.3	6074.7	6379.2	-2.8(-2.9,-2.6)	192604.3	5626.5	5903.5	-2.7(-2.9,-2.6)	228402.0	6512.2	6837.7	-2.9(-3.0,-2.7)
Algeria	Overall nutritional deficiencies	616516.7	1473.3	1464.2	-0.2(-0.4,0.0)	299072.1	1448.6	1451.4	-0.1(-0.4,0.1)	317444.6	1497.3	1476.9	-0.2(-0.4,0.0)
	Protein-energy malnutrition	603887.9	1443.1	1434.3	-0.1(-0.4,0.1)	291482.6	1411.8	1451.4	-0.1(-0.3,0.1)	312405.3	1473.5	1453.3	-0.2(-0.4,0.0)
	Iodine deficiency	12628.8	30.2	29.9	-1.2(-1.4,-1.1)	7589.5	36.8	36.6	-1.1(-1.2,-0.9)	5039.3	23.8	23.5	-1.4(-1.6,-1.3)
	Vitamin A deficiency	1104506.3	2639.4	2690.4	-4.6(-4.8,-4.4)	456375.0	2210.5	2268.5	-4.2(-4.4,-4.0)	648131.2	3057.0	3100.8	-4.8(-5.0,-4.7)
Egypt	Overall nutritional deficiencies	1328339.4	1340.8	1308.5	-0.5(-0.7,-0.3)	608689.1	1275.9	1250.6	-0.4(-0.6,-0.3)	719650.3	1401.1	1365.2	-0.6(-0.8,-0.3)
	Protein-energy malnutrition	1290196.3	1302.3	1273.2	-0.4(-0.6,-0.2)	580898.6	1217.6	1250.6	-0.3(-0.5,-0.1)	709297.7	1381.0	1346.3	-0.5(-0.8,-0.3)
	Iodine deficiency	38143.1	38.5	35.3	-3.0(-3.2,-2.7)	27790.5	58.3	52.9	-2.9(-3.2,-2.7)	10352.6	20.2	18.9	-3.0(-3.3,-2.8)
	Vitamin A deficiency	1909494.4	1927.4	1925.2	-3.8(-4.2,-3.5)	960882.6	2014.1	2006.9	-3.3(-3.7,-2.9)	948611.9	1846.9	1848.7	-4.3(-4.7,-4.0)
Iran (Islamic Republic of)	Overall nutritional deficiencies	1146864.9	1360.5	1467.3	-0.2(-0.4,0.0)	560391.0	1351.0	1460.5	0.0(-0.3,0.2)	586473.9	1369.7	1473.3	-0.3(-0.6,-0.1)
	Protein-energy malnutrition	1132989.6	1344.0	1450.8	-0.2(-0.4,0.0)	551699.3	1330.0	1460.5	0.0(-0.2,0.2)	581290.3	1357.6	1461.1	-0.3(-0.5,-0.1)
	Iodine deficiency	13875.3	16.5	16.6	-1.6(-1.8,-1.4)	8691.7	21.0	21.2	-1.5(-1.7,-1.3)	5183.6	12.1	12.1	-1.8(-2.0,-1.6)

	Vitamin A deficiency	1105546.9	1311.5	1344.7	-7.0(-7.6,-6.5)	456080.0	1099.5	1143.3	-6.5(-7.0,-6.0)	649466.8	1516.8	1538.9	-7.4(-7.9,-6.8)
Iraq	Overall nutritional deficiencies	683577.7	1622.9	1512.7	0.1(-0.1,0.3)	319866.2	1556.5	1454.2	0.3(0.0,0.5)	363711.5	1686.3	1568.3	-0.1(-0.2,0.1)
	Protein-energy malnutrition	658962.3	1564.5	1459.7	0.2(0.0,0.4)	305086.0	1484.6	1454.2	0.4(0.2,0.6)	353876.3	1640.7	1526.5	0.0(-0.1,0.2)
	Iodine deficiency	24615.4	58.4	53.1	-1.8(-2.3,-1.2)	14780.3	71.9	65.0	-1.6(-2.1,-1.1)	9835.1	45.6	41.7	-1.9(-2.5,-1.4)
	Vitamin A deficiency	1337629.4	3175.8	3209.2	-5.2(-5.5,-4.8)	568486.6	2766.3	2796.5	-4.9(-5.2,-4.7)	769142.8	3565.9	3600.2	-5.3(-5.7,-4.9)
Jordan	Overall nutritional deficiencies	150631.3	1294.4	1305.7	0.1(0.0,0.2)	68674.4	1266.6	1272.8	-0.1(-0.2,0.0)	81956.9	1318.8	1337.9	0.3(0.1,0.4)
	Protein-energy malnutrition	147017.4	1263.4	1277.1	0.1(0.0,0.2)	66689.4	1230.0	1272.8	-0.1(-0.2,0.0)	80328.0	1292.6	1313.3	0.3(0.2,0.4)
	Iodine deficiency	3613.9	31.1	28.7	-1.2(-1.3,-1.1)	1985.0	36.6	33.3	-1.1(-1.1,-1.0)	1628.9	26.2	24.6	-1.4(-1.6,-1.2)
	Vitamin A deficiency	575229.2	4943.2	5045.5	-3.7(-4.1,-3.3)	235641.3	4346.0	4476.8	-3.3(-3.7,-2.9)	339587.9	5464.3	5543.8	-4.0(-4.4,-3.5)
Lebanon	Overall nutritional deficiencies	70643.8	1364.6	1370.2	0.1(0.0,0.3)	35055.9	1333.2	1351.8	0.2(0.0,0.3)	35587.9	1396.9	1383.1	0.1(-0.1,0.2)
	Protein-energy malnutrition	67662.4	1307.0	1310.2	0.3(0.1,0.4)	33264.0	1265.1	1351.8	0.3(0.2,0.5)	34398.4	1350.2	1336.0	0.2(0.0,0.4)
	Iodine deficiency	2981.4	57.6	59.9	-2.3(-2.8,-1.9)	1791.9	68.1	73.6	-2.2(-2.6,-1.8)	1189.5	46.7	47.1	-2.6(-3.0,-2.2)
	Vitamin A deficiency	85319.9	1648.0	1682.7	-4.8(-5.0,-4.5)	44295.1	1684.6	1727.9	-4.5(-4.7,-4.3)	41024.8	1610.3	1637.5	-5.1(-5.3,-4.8)
Libya	Overall nutritional deficiencies	80346.9	1192.9	1431.6	0.2(0.1,0.4)	37479.4	1150.8	1382.8	0.3(0.1,0.4)	42867.5	1232.2	1477.7	0.2(0.0,0.4)
	Protein-energy malnutrition	76968.6	1142.7	1379.7	0.3(0.1,0.4)	35484.7	1089.6	1382.8	0.3(0.1,0.5)	41484.0	1192.5	1436.8	0.2(0.1,0.4)
	Iodine deficiency	3378.3	50.2	51.9	-0.1(-0.4,0.3)	1994.7	61.3	63.6	0.1(-0.2,0.5)	1383.6	39.8	40.9	-0.3(-0.6,0.1)
	Vitamin A deficiency	121052.8	1797.2	1829.2	-4.6(-5.2,-4.0)	55442.9	1702.4	1733.6	-4.4(-4.9,-3.9)	65609.9	1886.0	1918.5	-4.8(-5.4,-4.2)
Morocco	Overall nutritional deficiencies	439666.0	1222.9	1315.8	-0.1(-0.3,0.1)	213799.9	1196.1	1301.3	-0.1(-0.3,0.1)	225866.1	1249.4	1331.2	-0.1(-0.3,0.1)
	Protein-energy malnutrition	416095.0	1157.4	1249.5	0.1(-0.1,0.3)	199616.7	1116.8	1301.3	0.1(-0.1,0.4)	216478.3	1197.5	1279.3	0.1(-0.2,0.3)
	Iodine deficiency	23571.0	65.6	66.3	-2.4(-2.6,-2.2)	14183.3	79.3	81.2	-2.3(-2.5,-2.1)	9387.8	51.9	51.9	-2.6(-2.8,-2.4)
	Vitamin A deficiency	2140589.7	5954.0	6117.3	-4.0(-4.2,-3.7)	796522.7	4456.2	4673.6	-3.7(-3.9,-3.5)	1344067.0	7434.9	7536.4	-4.1(-4.3,-3.9)
Palestine	Overall nutritional deficiencies	58086.6	1171.9	1123.2	0.1(-0.1,0.3)	34012.3	1398.9	1345.0	0.2(0.0,0.4)	24074.3	953.4	907.6	0.0(-0.2,0.2)
	Protein-energy malnutrition	56807.3	1146.1	1100.0	0.1(0.0,0.3)	33240.6	1367.1	1345.0	0.2(0.1,0.4)	23566.7	933.3	889.4	0.0(-0.1,0.2)
	Iodine deficiency	1279.3	25.8	23.2	-1.2(-1.2,-1.1)	771.7	31.7	28.3	-1.0(-1.1,-0.9)	507.6	20.1	18.2	-1.4(-1.4,-1.3)
	Vitamin A deficiency	266715.6	5381.0	5435.6	-6.8(-7.2,-6.4)	106901.2	4396.7	4450.4	-6.0(-6.4,-5.6)	159814.4	6328.8	6379.0	-7.3(-7.8,-6.8)
Syrian Arab Republic	Overall nutritional deficiencies	218141.9	1505.3	1689.2	0.0(-0.2,0.2)	109136.9	1468.2	1694.8	0.1(-0.1,0.3)	109005.1	1544.4	1685.8	0.0(-0.2,0.1)
	Protein-energy malnutrition	209376.8	1444.9	1632.1	0.1(-0.1,0.2)	103742.1	1395.6	1694.8	0.1(-0.1,0.3)	105634.8	1496.7	1641.4	0.0(-0.2,0.2)
	Iodine deficiency	8765.1	60.5	57.1	-1.3(-1.6,-1.1)	5394.8	72.6	69.6	-1.2(-1.5,-1.0)	3370.3	47.8	44.4	-1.6(-1.8,-1.3)
	Vitamin A deficiency	529820.8	3656.1	3722.4	-4.6(-4.8,-4.4)	240134.1	3230.5	3291.4	-4.4(-4.7,-4.2)	289686.7	4104.4	4179.6	-4.7(-5.0,-4.5)
Tunisia	Overall nutritional deficiencies	136143.5	1176.5	1310.6	0.0(-0.1,0.1)	68028.9	1171.1	1325.9	0.0(-0.1,0.2)	68114.6	1182.0	1296.2	0.0(-0.1,0.1)
	Protein-energy malnutrition	134075.5	1158.7	1292.1	0.0(-0.1,0.2)	66926.9	1152.1	1325.9	0.1(-0.1,0.2)	67148.6	1165.2	1279.0	0.0(-0.1,0.1)
	Iodine deficiency	2068.0	17.9	18.5	-1.6(-1.7,-1.5)	1102.0	19.0	19.8	-1.4(-1.5,-1.3)	966.0	16.8	17.1	-1.8(-1.9,-1.7)
	Vitamin A deficiency	202042.0	1746.0	1755.0	-5.2(-5.5,-5.0)	95043.7	1636.2	1638.9	-4.9(-5.2,-4.6)	106998.2	1856.7	1870.6	-5.5(-5.7,-5.2)

Turkey	Overall nutritional deficiencies	896321.1	1101.7	1191.1	1.5(1.2,1.7)	518184.9	1287.2	1405.8	1.4(1.2,1.6)	378136.2	920.0	981.4	1.5(1.2,1.8)
	Protein-energy malnutrition	860176.7	1057.3	1141.3	1.8(1.6,2.1)	497342.0	1235.4	1405.8	1.8(1.5,2.0)	362834.7	882.7	940.3	1.9(1.6,2.2)
	Iodine deficiency	36144.4	44.4	49.8	-2.9(-3.2,-2.7)	20842.9	51.8	59.0	-2.9(-3.1,-2.6)	15301.5	37.2	41.1	-3.0(-3.3,-2.7)
	Vitamin A deficiency	2574869.7	3164.8	3184.3	-5.2(-5.3,-5.1)	1564612.5	3886.6	3905.7	-4.7(-4.9,-4.6)	1010257.2	2457.8	2492.7	-5.8(-5.9,-5.7)
Yemen	Overall nutritional deficiencies	987923.5	3136.0	2613.6	-0.9(-1.0,-0.8)	392196.8	2518.2	2025.2	-0.4(-0.6,-0.3)	595726.7	3740.0	3195.6	-1.2(-1.4,-1.1)
	Protein-energy malnutrition	941519.6	2988.7	2502.5	-1.0(-1.1,-0.8)	364067.6	2337.6	2025.2	-0.4(-0.6,-0.2)	577452.0	3625.3	3109.0	-1.2(-1.4,-1.1)
	Iodine deficiency	46403.9	147.3	111.1	-0.6(-1.0,-0.1)	28129.2	180.6	136.8	-0.4(-0.9,0.1)	18274.7	114.7	86.6	-0.8(-1.2,-0.3)
	Vitamin A deficiency	4727578.6	15006.8	15059.3	-4.5(-4.8,-4.2)	1799488.1	11554.2	11609.7	-4.6(-4.9,-4.3)	2928090.5	18382.6	18416.6	-4.4(-4.7,-4.1)
Afghanistan	Overall nutritional deficiencies	678527.2	1772.7	1215.5	-0.3(-0.5,-0.1)	287870.0	1544.4	1046.1	-1.0(-1.2,-0.7)	390657.2	1989.3	1377.5	0.3(0.1,0.5)
	Protein-energy malnutrition	644967.5	1685.0	1147.6	-0.3(-0.4,-0.1)	267584.4	1435.5	1046.1	-1.0(-1.2,-0.8)	377383.1	1921.7	1324.2	0.4(0.2,0.6)
	Iodine deficiency	33559.6	87.7	67.9	-1.0(-1.8,-0.1)	20285.6	108.8	83.3	-0.8(-1.7,0.0)	13274.0	67.6	53.3	-1.2(-2.0,-0.3)
	Vitamin A deficiency	9976079.4	26062.5	26013.8	-1.6(-2.1,-1.2)	3986939.5	21389.3	21419.0	-1.6(-2.1,-1.1)	5989139.9	30498.3	30358.7	-1.7(-2.1,-1.2)
Bangladesh	Overall nutritional deficiencies	2893820.4	1817.0	1972.2	-1.7(-2.1,-1.4)	1318542.0	1634.1	1805.6	-2.0(-2.4,-1.6)	1575278.4	2004.9	2136.8	-1.5(-1.8,-1.2)
	Protein-energy malnutrition	2675906.6	1680.2	1847.5	-1.7(-2.1,-1.3)	1177056.6	1458.7	1805.6	-2.0(-2.4,-1.5)	1498850.0	1907.7	2045.9	-1.5(-1.8,-1.1)
	Iodine deficiency	217913.8	136.8	124.8	-1.9(-2.5,-1.2)	141485.4	175.3	156.4	-1.6(-2.3,-0.9)	76428.3	97.3	90.9	-2.3(-2.9,-1.7)
	Vitamin A deficiency	7439736.9	4671.4	4761.9	-4.3(-4.6,-4.0)	4156954.5	5151.8	5244.4	-3.6(-4.0,-3.3)	3282782.4	4178.2	4262.1	-4.9(-5.3,-4.6)
Bhutan	Overall nutritional deficiencies	23007.3	3050.4	3138.2	1.1(0.8,1.4)	5790.2	1601.8	1684.2	-1.1(-1.3,-0.9)	17217.1	4383.4	4489.4	2.4(2.1,2.8)
	Protein-energy malnutrition	22754.4	3016.8	3107.5	1.1(0.8,1.5)	5613.4	1552.9	1684.2	-1.1(-1.3,-0.9)	17141.0	4364.1	4471.3	2.5(2.1,2.8)
	Iodine deficiency	252.9	33.5	30.7	-0.9(-1.4,-0.5)	176.9	48.9	44.3	-1.1(-1.6,-0.6)	76.1	19.4	18.1	-0.7(-0.9,-0.4)
	Vitamin A deficiency	43653.2	5787.6	5858.6	-5.0(-5.1,-5.0)	20845.0	5766.7	5878.5	-4.4(-4.5,-4.3)	22808.1	5806.9	5840.3	-5.5(-5.5,-5.4)
India	Overall nutritional deficiencies	56050385.4	4030.4	4321.4	-0.1(-0.3,0.0)	26111294.4	3853.9	4200.5	-0.2(-0.4,0.0)	29939090.9	4197.9	4430.3	-0.1(-0.2,0.0)
	Protein-energy malnutrition	52699182.9	3789.4	4102.7	0.0(-0.2,0.1)	24137477.2	3562.6	4200.5	-0.1(-0.3,0.2)	28561705.7	4004.8	4255.8	0.0(-0.1,0.1)
	Iodine deficiency	3351202.4	241.0	218.7	-1.6(-2.1,-1.2)	1973817.2	291.3	266.2	-1.4(-1.8,-1.0)	1377385.2	193.1	174.5	-1.9(-2.4,-1.4)
	Vitamin A deficiency	110835606.0	7969.7	8129.9	-4.4(-4.7,-4.1)	42234239.8	6233.6	6468.8	-4.0(-4.3,-3.7)	68601366.2	9619.0	9687.5	-4.6(-4.9,-4.3)
Nepal	Overall nutritional deficiencies	746818.3	2455.3	2564.5	-0.4(-0.5,-0.3)	347192.3	2181.1	2311.7	-0.5(-0.7,-0.3)	399626.0	2756.5	2850.6	-0.3(-0.4,-0.2)
	Protein-energy malnutrition	726085.5	2387.2	2503.9	-0.4(-0.5,-0.3)	334131.9	2099.0	2311.7	-0.5(-0.7,-0.4)	391953.6	2703.5	2802.8	-0.3(-0.4,-0.2)
	Iodine deficiency	20732.7	68.2	60.6	0.5(-0.1,1.1)	13060.4	82.0	72.2	1.1(0.3,1.9)	7672.4	52.9	47.8	-0.4(-0.7,-0.1)
	Vitamin A deficiency	1703360.8	5600.1	5693.3	-4.8(-5.0,-4.7)	1087113.4	6829.2	6989.2	-4.2(-4.4,-4.0)	616247.5	4250.6	4282.2	-5.8(-5.9,-5.6)
Pakistan	Overall nutritional deficiencies	6667345.9	2975.7	2438.5	-0.9(-1.0,-0.7)	3361403.9	3077.7	2575.8	-0.8(-0.9,-0.6)	3305942.0	2878.6	2304.7	-1.0(-1.2,-0.7)
	Protein-energy malnutrition	6001951.0	2678.7	2190.4	-1.0(-1.2,-0.8)	2787588.3	2552.3	2575.8	-1.1(-1.3,-0.9)	3214362.6	2798.8	2235.7	-1.0(-1.2,-0.7)
	Iodine deficiency	665395.0	297.0	248.0	0.6(0.6,0.7)	573815.6	525.4	435.4	0.8(0.8,0.9)	91579.4	79.7	69.0	-0.8(-0.9,-0.7)
	Vitamin A deficiency	7910724.9	3530.6	3521.7	-6.1(-6.5,-5.8)	4235296.3	3877.9	3903.4	-5.0(-5.2,-4.8)	3675428.6	3200.3	3158.8	-7.1(-7.6,-6.6)
Angola	Overall nutritional deficiencies	457581.1	1518.3	1133.2	-2.4(-2.7,-2.2)	259414.0	1671.9	1255.5	-2.3(-2.6,-2.0)	198167.1	1355.2	1011.4	-2.6(-2.8,-2.4)

	Protein-energy malnutrition	344902.1	1144.4	855.9	-2.3(-2.4,-2.1)	182898.8	1178.8	1255.5	-1.8(-2.0,-1.6)	162003.3	1107.9	828.9	-2.7(-2.8,-2.5)
	Iodine deficiency	112679.0	373.9	277.3	-3.0(-3.5,-2.5)	76515.2	493.1	368.0	-3.3(-3.8,-2.8)	36163.8	247.3	182.5	-2.3(-2.8,-1.9)
	Vitamin A deficiency	4301084.5	14271.1	14328.4	-4.0(-4.4,-3.7)	1717259.7	11067.8	11230.9	-3.8(-4.2,-3.4)	2583824.7	17670.0	17590.1	-4.2(-4.5,-3.8)
Central African Republic	Overall nutritional deficiencies	90909.1	1715.3	1266.2	-0.6(-0.7,-0.6)	51005.0	1885.6	1377.9	-0.7(-0.8,-0.6)	39904.2	1537.8	1161.1	-0.5(-0.7,-0.4)
	Protein-energy malnutrition	73585.5	1388.4	1013.0	-0.2(-0.4,-0.1)	39573.6	1463.0	1377.9	-0.3(-0.4,-0.2)	34011.9	1310.7	985.7	-0.1(-0.4,0.2)
	Iodine deficiency	17323.6	326.9	253.2	-2.0(-2.4,-1.6)	11431.3	422.6	328.4	-1.8(-2.1,-1.5)	5892.3	227.1	175.5	-2.4(-3.1,-1.8)
	Vitamin A deficiency	1759901.8	33206.6	33496.1	-1.1(-1.2,-1.0)	525625.0	19432.2	19728.5	-1.2(-1.4,-1.0)	1234276.8	47564.7	47754.7	-1.0(-1.1,-1.0)
Congo	Overall nutritional deficiencies	69202.9	1314.2	1161.3	-0.8(-0.9,-0.6)	38010.9	1431.2	1250.2	-0.9(-1.0,-0.8)	31192.0	1195.1	1071.0	-0.6(-0.7,-0.4)
	Protein-energy malnutrition	43736.5	830.6	765.2	-0.7(-0.9,-0.5)	20277.8	763.5	1250.2	-0.6(-0.8,-0.4)	23458.7	898.8	827.0	-0.8(-1.0,-0.6)
	Iodine deficiency	25466.3	483.6	396.0	-0.9(-1.0,-0.8)	17733.1	667.7	544.9	-1.3(-1.5,-1.2)	7733.2	296.3	244.0	0.2(0.0,0.4)
	Vitamin A deficiency	1376526.9	26140.7	27043.5	-1.8(-2.2,-1.5)	408996.0	15399.2	16138.7	-2.1(-2.6,-1.7)	967530.9	37071.7	38140.2	-1.7(-2.0,-1.4)
Democratic Republic of the Congo	Overall nutritional deficiencies	2099785.0	2395.1	1820.9	-0.9(-1.2,-0.5)	1302615.5	2974.8	2269.3	-0.9(-1.2,-0.6)	797169.5	1816.6	1371.7	-0.8(-1.1,-0.5)
	Protein-energy malnutrition	1439571.2	1642.0	1271.7	-1.0(-1.3,-0.6)	876926.1	2002.7	2269.3	-0.9(-1.3,-0.6)	562645.1	1282.2	983.4	-1.0(-1.3,-0.7)
	Iodine deficiency	660213.7	753.1	549.2	-0.6(-0.9,-0.3)	425689.4	972.2	712.2	-0.8(-1.1,-0.6)	234524.4	534.4	388.3	-0.2(-0.6,0.1)
	Vitamin A deficiency	26160531.1	29839.6	30146.1	-0.6(-1.2,-0.1)	9355206.9	21364.9	21849.1	-0.6(-1.2,0.1)	16805324.1	38295.9	38353.8	-0.7(-1.2,-0.2)
Equatorial Guinea	Overall nutritional deficiencies	14804.7	1042.7	878.8	-4.2(-4.8,-3.6)	7879.3	1206.9	990.4	-4.4(-5.0,-3.7)	6925.4	903.0	788.6	-3.9(-4.4,-3.3)
	Protein-energy malnutrition	10811.5	761.5	670.6	-2.5(-2.9,-2.1)	5428.6	831.5	990.4	-2.6(-3.1,-2.0)	5382.8	701.8	644.2	-2.4(-2.8,-2.0)
	Iodine deficiency	3993.3	281.2	208.2	-7.2(-8.0,-6.3)	2450.7	375.4	291.0	-7.0(-7.8,-6.2)	1542.6	201.1	144.4	-7.1(-8.0,-6.2)
	Vitamin A deficiency	63631.6	4481.6	4574.2	-9.9(-10.4,-9.4)	21089.9	3230.3	3319.0	-9.9(-10.3,-9.4)	42541.8	5546.7	5605.1	-10.1(-10.6,-9.6)
Gabon	Overall nutritional deficiencies	14216.7	812.4	783.6	-1.7(-1.9,-1.5)	7912.9	874.6	851.5	-2.2(-2.4,-1.9)	6303.8	745.7	713.0	-1.1(-1.2,-1.0)
	Protein-energy malnutrition	11863.6	677.9	666.1	-0.8(-1.0,-0.7)	6320.5	698.6	851.5	-1.1(-1.3,-0.9)	5543.1	655.7	633.7	-0.6(-0.7,-0.4)
	Iodine deficiency	2353.0	134.5	117.5	-4.5(-5.1,-3.9)	1592.3	176.0	153.3	-4.9(-5.5,-4.2)	760.7	90.0	79.3	-3.8(-4.3,-3.3)
	Vitamin A deficiency	78191.4	4468.0	4611.7	-5.0(-5.1,-4.8)	29398.8	3249.5	3383.7	-4.4(-4.6,-4.3)	48792.7	5772.2	5922.1	-5.2(-5.4,-5.1)
Burundi	Overall nutritional deficiencies	217048.0	1818.7	1320.9	-1.6(-1.7,-1.5)	105738.6	1760.7	1268.9	-1.1(-1.2,-1.0)	111309.4	1877.4	1369.2	-2.1(-2.3,-1.8)
	Protein-energy malnutrition	201264.2	1686.4	1208.3	-1.5(-1.6,-1.4)	96040.7	1599.2	1268.9	-1.0(-1.1,-0.8)	105223.5	1774.7	1279.3	-2.1(-2.3,-1.8)
	Iodine deficiency	15783.7	132.3	112.6	-2.1(-2.2,-1.9)	9697.9	161.5	133.8	-2.0(-2.2,-1.9)	6085.8	102.6	89.9	-2.1(-2.3,-2.0)
	Vitamin A deficiency	2725352.6	22836.2	22039.3	-2.0(-2.2,-1.8)	1150115.2	19151.4	18726.9	-1.8(-2.1,-1.6)	1575237.4	26568.4	25430.4	-2.1(-2.3,-1.9)
Comoros	Overall nutritional deficiencies	8425.0	1179.4	1153.0	-1.2(-1.3,-1.1)	4549.9	1272.4	1249.2	-0.9(-1.0,-0.8)	3875.0	1086.1	1057.7	-1.5(-1.6,-1.4)
	Protein-energy malnutrition	8256.4	1155.8	1130.8	-1.2(-1.3,-1.1)	4449.7	1244.4	1249.2	-0.9(-1.1,-0.8)	3806.7	1067.0	1039.7	-1.5(-1.6,-1.4)
	Iodine deficiency	168.6	23.6	22.2	-0.1(-0.2,-0.1)	100.3	28.0	26.3	-0.1(-0.2,-0.1)	68.3	19.1	18.1	-0.1(-0.2,0.0)
	Vitamin A deficiency	141702.9	19836.6	19533.7	-2.6(-2.7,-2.4)	50507.2	14124.6	14124.3	-2.8(-2.9,-2.6)	91195.7	25561.6	24875.9	-2.5(-2.7,-2.4)
Djibouti	Overall nutritional deficiencies	30164.2	2507.8	2156.5	-0.8(-1.0,-0.6)	15201.4	2698.2	2271.1	-1.0(-1.2,-0.9)	14962.8	2340.1	2054.5	-0.6(-0.8,-0.3)
	Protein-energy malnutrition	23836.0	1981.7	1679.9	-0.9(-1.1,-0.8)	11813.4	2096.8	2271.1	-1.2(-1.3,-1.1)	12022.6	1880.3	1642.4	-0.7(-0.9,-0.4)

	Iodine deficiency	6328.2	526.1	476.6	-0.3(-0.7,0.1)	3388.1	601.4	552.7	-0.4(-0.7,0.0)	2940.2	459.8	412.0	-0.3(-0.7,0.1)
	Vitamin A deficiency	202048.2	16798.2	16576.5	-3.5(-3.8,-3.3)	73644.7	13071.6	13146.5	-3.2(-3.4,-3.0)	128403.5	20081.8	19591.5	-3.7(-4.0,-3.5)
Eritrea	Overall nutritional deficiencies	116179.4	1731.1	1436.3	-2.0(-2.2,-1.8)	52276.9	1566.9	1292.4	-1.9(-2.0,-1.8)	63902.5	1893.5	1587.7	-2.1(-2.5,-1.8)
	Protein-energy malnutrition	114088.6	1700.0	1408.4	-2.0(-2.2,-1.8)	51055.0	1530.3	1292.4	-1.9(-2.0,-1.8)	63033.6	1867.7	1564.4	-2.2(-2.5,-1.8)
	Iodine deficiency	2090.8	31.2	27.8	-1.1(-1.3,-0.9)	1221.9	36.6	32.6	-1.2(-1.4,-1.0)	868.9	25.7	23.3	-1.0(-1.2,-0.8)
	Vitamin A deficiency	1505043.1	22425.8	22128.4	-2.9(-3.0,-2.8)	577529.2	17310.3	17269.6	-2.9(-3.0,-2.8)	927513.9	27482.9	26862.8	-2.9(-3.0,-2.8)
Ethiopia	Overall nutritional deficiencies	2428189.5	2256.9	1791.7	-1.1(-1.3,-0.8)	1202274.1	2256.2	1779.9	-1.1(-1.3,-0.9)	1225915.4	2257.5	1802.9	-1.0(-1.4,-0.7)
	Protein-energy malnutrition	1881004.1	1748.3	1387.3	-1.2(-1.4,-0.9)	898196.9	1685.6	1779.9	-1.2(-1.3,-1.0)	982807.2	1809.8	1445.1	-1.2(-1.5,-0.8)
	Iodine deficiency	547185.4	508.6	404.5	-0.8(-1.3,-0.3)	304077.2	570.6	451.6	-1.0(-1.5,-0.6)	243108.3	447.7	357.8	-0.5(-1.1,0.1)
	Vitamin A deficiency	26813284.7	24921.5	24329.1	-2.9(-3.3,-2.5)	11200491.3	21019.3	20767.1	-3.0(-3.4,-2.6)	15612793.4	28750.4	27823.1	-2.8(-3.2,-2.5)
Kenya	Overall nutritional deficiencies	920318.9	1832.3	1729.0	-0.4(-0.6,-0.2)	387671.1	1540.6	1440.1	-0.3(-0.5,-0.2)	532647.9	2125.1	2026.1	-0.5(-0.7,-0.2)
	Protein-energy malnutrition	886329.2	1764.6	1670.6	-0.4(-0.6,-0.2)	369466.0	1468.3	1440.1	-0.4(-0.5,-0.2)	516863.1	2062.1	1971.4	-0.5(-0.7,-0.3)
	Iodine deficiency	33989.8	67.7	58.4	0.1(-0.1,0.4)	18205.0	72.3	62.0	0.0(-0.3,0.3)	15784.8	63.0	54.7	0.4(0.1,0.6)
	Vitamin A deficiency	14836608.9	29538.7	29712.6	-2.0(-2.2,-1.9)	7464609.1	29665.0	29768.6	-1.9(-2.1,-1.7)	7371999.7	29411.8	29656.8	-2.1(-2.3,-2.0)
Madagascar	Overall nutritional deficiencies	514379.4	1927.2	1629.3	-1.4(-1.5,-1.3)	259661.5	1946.2	1570.5	-1.4(-1.5,-1.3)	254717.8	1908.2	1699.1	-1.4(-1.5,-1.2)
	Protein-energy malnutrition	484344.3	1814.7	1534.0	-1.5(-1.6,-1.4)	241348.1	1809.0	1570.5	-1.5(-1.6,-1.5)	242996.2	1820.4	1623.9	-1.4(-1.6,-1.3)
	Iodine deficiency	30035.0	112.5	95.2	0.3(0.1,0.5)	18313.4	137.3	115.3	0.3(0.1,0.5)	11721.6	87.8	75.2	0.4(0.2,0.5)
	Vitamin A deficiency	5945036.2	22274.1	21864.7	-2.0(-2.2,-1.7)	2404822.4	18024.7	17943.7	-1.7(-2.0,-1.5)	3540213.8	26521.3	25767.6	-2.1(-2.3,-1.9)
Malawi	Overall nutritional deficiencies	331564.8	1797.9	1655.6	-0.9(-1.1,-0.7)	219360.4	2315.5	2135.5	-0.4(-0.7,-0.2)	112204.4	1251.1	1149.2	-1.7(-1.9,-1.5)
	Protein-energy malnutrition	306284.6	1660.8	1544.8	-0.9(-1.1,-0.6)	204783.5	2161.6	2135.5	-0.4(-0.6,-0.1)	101501.1	1131.7	1052.1	-1.7(-1.9,-1.5)
	Iodine deficiency	25280.2	137.1	110.7	-1.4(-1.7,-1.0)	14576.9	153.9	123.6	-1.3(-1.5,-1.0)	10703.3	119.3	97.1	-1.5(-1.8,-1.1)
	Vitamin A deficiency	4866233.1	26386.3	25987.3	-2.6(-2.9,-2.4)	2633954.7	27802.8	27349.2	-2.1(-2.4,-1.8)	2232278.4	24890.1	24560.3	-3.1(-3.3,-3.0)
Mozambique	Overall nutritional deficiencies	296468.1	1004.0	791.3	-2.6(-2.7,-2.4)	130620.4	852.2	668.0	-2.6(-2.8,-2.4)	165847.7	1167.9	930.4	-2.5(-2.7,-2.3)
	Protein-energy malnutrition	278961.5	944.7	739.5	-2.6(-2.8,-2.3)	120208.0	784.3	668.0	-2.6(-2.8,-2.3)	158753.5	1118.0	885.9	-2.5(-2.7,-2.3)
	Iodine deficiency	17506.6	59.3	51.8	-2.5(-2.9,-2.2)	10412.3	67.9	58.6	-2.6(-2.9,-2.2)	7094.2	50.0	44.5	-2.5(-2.8,-2.1)
	Vitamin A deficiency	8598573.4	29120.0	28865.4	-2.9(-3.0,-2.8)	3387894.3	22103.1	22189.9	-3.0(-3.1,-2.8)	5210679.1	36694.0	36007.4	-2.9(-3.0,-2.7)
Rwanda	Overall nutritional deficiencies	145526.6	1147.0	1071.4	-1.8(-2.0,-1.6)	78060.7	1197.7	1123.7	-1.5(-1.7,-1.2)	67465.9	1093.3	1018.7	-2.1(-2.4,-1.9)
	Protein-energy malnutrition	127065.4	1001.5	949.5	-1.8(-2.0,-1.5)	66798.8	1024.9	1123.7	-1.4(-1.7,-1.1)	60266.5	976.7	921.4	-2.2(-2.4,-1.9)
	Iodine deficiency	18461.2	145.5	121.9	-1.8(-2.2,-1.5)	11261.8	172.8	146.1	-1.8(-2.2,-1.5)	7199.4	116.7	97.3	-1.8(-2.2,-1.5)
	Vitamin A deficiency	1872246.2	14755.9	14592.4	-3.1(-3.5,-2.8)	826466.6	12680.7	12717.5	-2.9(-3.3,-2.5)	1045779.6	16947.8	16536.4	-3.3(-3.6,-2.9)
Somalia	Overall nutritional deficiencies	665125.1	3269.5	2276.6	-1.3(-1.5,-1.1)	351573.4	3527.0	2410.7	-1.4(-1.6,-1.3)	313551.7	3022.2	2163.0	-1.1(-1.4,-0.9)
	Protein-energy malnutrition	482901.2	2373.8	1551.4	-1.9(-2.1,-1.6)	252406.2	2532.1	2410.7	-2.1(-2.4,-1.9)	230494.9	2221.7	1521.2	-1.7(-2.0,-1.4)
	Iodine deficiency	182224.0	895.8	725.1	0.3(0.2,0.4)	99167.2	994.8	814.9	0.2(0.1,0.3)	83056.8	800.6	641.8	0.4(0.3,0.5)

	Vitamin A deficiency	13022650.7	64015.0	63640.1	-0.3(-0.3,-0.3)	5427045.5	54443.6	54280.6	-0.3(-0.3,-0.3)	7595605.1	73211.2	72502.1	-0.3(-0.3,-0.3)
United Republic of Tanzania	Overall nutritional deficiencies	586036.4	1032.9	787.0	-1.7(-1.9,-1.6)	301403.4	1034.0	805.8	-1.4(-1.5,-1.2)	284633.0	1031.8	767.5	-2.1(-2.3,-2.0)
	Protein-energy malnutrition	571151.4	1006.7	762.8	-1.7(-1.9,-1.6)	292383.4	1003.1	805.8	-1.4(-1.5,-1.2)	278768.0	1010.5	747.4	-2.1(-2.3,-2.0)
	Iodine deficiency	14885.0	26.2	24.2	-1.3(-1.5,-1.1)	9020.0	30.9	28.1	-1.4(-1.6,-1.2)	5865.0	21.3	20.1	-1.3(-1.4,-1.1)
	Vitamin A deficiency	8066024.7	14216.7	13929.6	-2.4(-2.6,-2.1)	3913047.8	13424.1	13332.9	-1.8(-2.1,-1.6)	4152976.9	15054.3	14557.8	-2.8(-3.0,-2.6)
Uganda	Overall nutritional deficiencies	430970.7	1048.1	821.4	-1.5(-1.6,-1.4)	234081.0	1120.3	888.0	-1.0(-1.2,-0.9)	196889.8	973.6	751.8	-1.9(-2.0,-1.8)
	Protein-energy malnutrition	408989.5	994.7	775.0	-1.5(-1.6,-1.4)	220964.2	1057.5	888.0	-1.0(-1.2,-0.8)	188025.4	929.8	713.2	-1.9(-2.1,-1.8)
	Iodine deficiency	21981.2	53.5	46.3	-1.6(-1.7,-1.5)	13116.8	62.8	53.8	-1.6(-1.7,-1.5)	8864.4	43.8	38.6	-1.5(-1.6,-1.4)
	Vitamin A deficiency	4903505.6	11925.5	11785.6	-3.6(-3.8,-3.4)	2327214.5	11137.6	11228.0	-3.2(-3.4,-3.0)	2576291.1	12739.6	12353.7	-3.9(-4.1,-3.7)
Zambia	Overall nutritional deficiencies	229415.0	1257.9	1078.4	-0.7(-0.8,-0.6)	110151.2	1194.6	1011.5	-0.5(-0.7,-0.4)	119263.8	1322.7	1148.1	-0.8(-1.0,-0.7)
	Protein-energy malnutrition	218288.5	1196.9	1025.9	-0.6(-0.8,-0.5)	103467.0	1122.1	1011.5	-0.5(-0.6,-0.3)	114821.5	1273.4	1104.6	-0.8(-0.9,-0.6)
	Iodine deficiency	11126.5	61.0	52.6	-1.6(-1.9,-1.2)	6684.2	72.5	61.2	-1.6(-1.9,-1.3)	4442.3	49.3	43.5	-1.5(-1.8,-1.2)
	Vitamin A deficiency	2543623.8	13947.1	13680.4	-3.8(-4.2,-3.3)	933157.8	10120.0	10138.6	-3.7(-4.1,-3.2)	1610465.9	17860.8	17302.4	-3.8(-4.3,-3.4)
Botswana	Overall nutritional deficiencies	22196.3	949.1	972.1	0.1(-0.1,0.3)	11118.5	935.9	970.4	0.3(0.1,0.6)	11077.7	962.7	977.1	-0.2(-0.4,0.0)
	Protein-energy malnutrition	21759.1	930.4	954.6	0.1(-0.1,0.3)	10863.3	914.4	970.4	0.4(0.1,0.6)	10895.8	946.9	962.3	-0.2(-0.4,0.0)
	Iodine deficiency	437.2	18.7	17.5	-0.4(-0.5,-0.4)	255.2	21.5	20.2	-0.3(-0.4,-0.3)	182.0	15.8	14.8	-0.5(-0.5,-0.4)
	Vitamin A deficiency	210163.6	8986.3	9632.5	-3.5(-3.6,-3.3)	96744.5	8143.3	8741.8	-3.2(-3.3,-3.1)	113419.0	9856.5	10534.6	-3.7(-3.8,-3.6)
Lesotho	Overall nutritional deficiencies	26324.6	1258.6	1242.8	-1.9(-2.2,-1.5)	10181.8	961.9	943.1	-2.4(-2.9,-1.8)	16142.8	1562.6	1573.5	-1.5(-1.7,-1.2)
	Protein-energy malnutrition	22683.0	1084.5	1093.8	-1.5(-1.7,-1.2)	8037.4	759.3	943.1	-1.9(-2.4,-1.5)	14645.6	1417.7	1451.4	-1.1(-1.3,-1.0)
	Iodine deficiency	3641.6	174.1	149.0	-4.0(-4.8,-3.2)	2144.4	202.6	176.4	-3.9(-4.7,-3.1)	1497.2	144.9	122.1	-4.0(-4.8,-3.1)
	Vitamin A deficiency	303756.4	14522.8	15405.1	-3.0(-3.1,-2.8)	126745.7	11973.6	12646.0	-2.9(-3.0,-2.7)	177010.7	17134.8	18157.1	-3.1(-3.2,-2.9)
Namibia	Overall nutritional deficiencies	33171.6	1380.4	1261.5	-0.6(-0.9,-0.3)	16875.3	1361.4	1257.6	-0.5(-0.8,-0.1)	16296.3	1400.6	1268.9	-0.8(-1.0,-0.5)
	Protein-energy malnutrition	32383.6	1347.6	1231.2	-0.6(-0.9,-0.3)	16378.1	1321.3	1257.6	-0.4(-0.7,-0.1)	16005.6	1375.6	1245.7	-0.8(-1.0,-0.5)
	Iodine deficiency	787.9	32.8	30.3	-1.3(-1.3,-1.2)	497.2	40.1	37.1	-1.3(-1.4,-1.2)	290.7	25.0	23.3	-1.3(-1.3,-1.3)
	Vitamin A deficiency	148577.6	6182.7	6569.4	-3.0(-3.2,-2.8)	71790.0	5791.4	6125.3	-2.7(-3.0,-2.4)	76787.7	6599.5	7032.9	-3.3(-3.5,-3.1)
South Africa	Overall nutritional deficiencies	530237.9	953.9	1005.5	-1.0(-1.2,-0.8)	210745.1	743.1	798.9	-1.4(-1.6,-1.1)	319492.8	1173.4	1232.6	-0.8(-1.1,-0.6)
	Protein-energy malnutrition	511895.5	920.9	973.6	-1.0(-1.3,-0.8)	199906.9	704.9	798.9	-1.4(-1.6,-1.1)	311988.6	1145.9	1206.3	-0.8(-1.1,-0.6)
	Iodine deficiency	18342.4	33.0	31.9	-0.8(-0.9,-0.7)	10838.3	38.2	37.7	-0.8(-0.9,-0.7)	7504.2	27.6	26.2	-0.8(-0.9,-0.7)
	Vitamin A deficiency	2013626.3	3622.4	4066.7	-4.3(-4.4,-4.2)	1030525.2	3633.6	4073.2	-3.6(-3.7,-3.5)	983101.1	3610.7	4060.2	-4.9(-5.1,-4.8)
Eswatini	Overall nutritional deficiencies	9321.6	816.2	780.4	-0.7(-0.8,-0.6)	3904.5	668.3	641.2	-0.9(-1.0,-0.8)	5417.1	971.1	938.7	-0.6(-0.7,-0.4)
	Protein-energy malnutrition	8744.4	765.6	735.6	-0.7(-0.8,-0.6)	3558.2	609.0	641.2	-0.9(-1.0,-0.8)	5186.2	929.7	901.9	-0.6(-0.7,-0.4)
	Iodine deficiency	577.2	50.5	44.8	-0.8(-0.8,-0.7)	346.3	59.3	52.9	-0.7(-0.8,-0.7)	230.9	41.4	36.8	-0.8(-0.8,-0.7)
	Vitamin A deficiency	94457.3	8270.4	8688.4	-3.7(-3.8,-3.6)	43231.7	7399.3	7770.5	-3.3(-3.4,-3.1)	51225.6	9182.8	9623.1	-4.1(-4.2,-4.0)

Zimbabwe	Overall nutritional deficiencies	221176.2	1473.4	1407.6	0.0(-0.2,0.2)	103228.3	1322.8	1243.0	0.2(0.0,0.5)	117947.9	1636.5	1609.2	-0.1(-0.3,0.1)
	Protein-energy malnutrition	199517.5	1329.2	1286.7	0.0(-0.1,0.2)	90712.1	1162.4	1243.0	0.3(0.0,0.5)	108805.4	1509.7	1502.9	-0.1(-0.3,0.1)
	Iodine deficiency	21658.7	144.3	120.9	-0.3(-0.6,0.1)	12516.1	160.4	134.9	-0.2(-0.5,0.1)	9142.5	126.9	106.3	-0.3(-0.7,0.0)
	Vitamin A deficiency	2882549.7	19203.1	19669.6	-0.6(-1.0,-0.3)	1494677.6	19153.5	19498.9	-0.1(-0.5,0.2)	1387872.1	19256.9	19851.2	-1.0(-1.4,-0.7)
Benin	Overall nutritional deficiencies	217401.5	1716.5	1259.3	-1.4(-1.6,-1.3)	106013.7	1646.4	1206.5	-1.0(-1.1,-0.9)	111387.8	1788.9	1318.2	-1.8(-2.0,-1.7)
	Protein-energy malnutrition	210555.3	1662.4	1211.2	-1.5(-1.6,-1.3)	101626.5	1578.3	1206.5	-1.0(-1.1,-0.9)	108928.8	1749.4	1282.5	-1.8(-2.0,-1.7)
	Iodine deficiency	6846.2	54.1	48.0	-0.9(-1.0,-0.8)	4387.2	68.1	59.8	-0.8(-0.9,-0.7)	2459.0	39.5	35.7	-0.9(-1.0,-0.8)
	Vitamin A deficiency	3371316.3	26617.6	27262.3	-2.1(-2.2,-2.0)	1103784.1	17142.0	17753.4	-1.9(-2.1,-1.6)	2267532.3	36416.3	37035.9	-2.2(-2.3,-2.2)
Burkina Faso	Overall nutritional deficiencies	563775.8	2484.5	1659.1	-0.6(-1.0,-0.2)	233036.2	1999.1	1390.0	-0.9(-1.2,-0.5)	330739.7	2997.3	1934.1	-0.3(-0.8,0.1)
	Protein-energy malnutrition	551217.7	2429.2	1609.4	-0.5(-0.9,-0.1)	225349.3	1933.1	1390.0	-0.8(-1.2,-0.4)	325868.4	2953.1	1894.3	-0.3(-0.7,0.1)
	Iodine deficiency	12558.1	55.3	49.7	-1.8(-2.0,-1.7)	7686.8	65.9	58.9	-1.8(-1.9,-1.7)	4871.3	44.1	39.8	-1.9(-2.0,-1.7)
	Vitamin A deficiency	6155171.1	27125.1	27594.1	-2.8(-2.9,-2.7)	2339591.2	20070.0	20743.0	-2.4(-2.5,-2.2)	3815579.9	34578.3	34743.6	-3.1(-3.2,-3.0)
Cameroon	Overall nutritional deficiencies	353144.2	1213.5	1048.2	-0.7(-0.8,-0.5)	152737.7	1045.1	901.0	-0.7(-0.8,-0.5)	200406.5	1383.3	1197.3	-0.7(-0.9,-0.4)
	Protein-energy malnutrition	340708.0	1170.7	1010.4	-0.7(-0.8,-0.5)	144875.5	991.3	901.0	-0.7(-0.8,-0.5)	195832.4	1351.7	1168.8	-0.7(-0.9,-0.5)
	Iodine deficiency	12436.2	42.7	37.8	-0.3(-0.4,-0.2)	7862.1	53.8	47.0	-0.2(-0.4,-0.1)	4574.1	31.6	28.5	-0.4(-0.5,-0.2)
	Vitamin A deficiency	7937384.2	27274.5	27949.8	-3.1(-3.3,-2.9)	1929207.8	13201.0	13878.3	-4.0(-4.2,-3.9)	6008176.4	41470.8	42090.7	-2.7(-2.9,-2.5)
Cabo Verde	Overall nutritional deficiencies	5211.3	924.7	964.7	-0.4(-0.6,-0.2)	2604.1	930.9	966.3	-0.6(-0.9,-0.2)	2607.3	918.6	965.3	-0.2(-0.3,-0.1)
	Protein-energy malnutrition	4776.5	847.6	892.9	-0.3(-0.5,0.0)	2336.2	835.1	966.3	-0.5(-0.8,-0.1)	2440.3	859.8	910.8	-0.1(-0.3,0.0)
	Iodine deficiency	434.8	77.2	71.7	-1.4(-1.6,-1.3)	267.9	95.8	89.8	-1.3(-1.5,-1.2)	167.0	58.8	54.5	-1.4(-1.6,-1.3)
	Vitamin A deficiency	28178.3	5000.0	5248.9	-6.1(-6.3,-5.8)	9976.8	3566.5	3804.5	-4.9(-5.0,-4.8)	18201.4	6412.9	6638.4	-6.7(-7.1,-6.4)
Chad	Overall nutritional deficiencies	425299.2	2593.5	1619.2	-1.0(-1.1,-0.9)	200556.5	2427.5	1499.7	-0.8(-1.0,-0.7)	224742.7	2762.0	1734.1	-1.1(-1.2,-1.0)
	Protein-energy malnutrition	414720.9	2529.0	1562.2	-0.9(-1.0,-0.8)	194173.0	2350.2	1499.7	-0.8(-1.0,-0.6)	220547.9	2710.4	1688.0	-1.1(-1.2,-1.0)
	Iodine deficiency	10578.3	64.5	57.0	-2.4(-2.6,-2.3)	6383.5	77.3	67.2	-2.4(-2.5,-2.2)	4194.8	51.6	46.1	-2.4(-2.6,-2.3)
	Vitamin A deficiency	5550082.8	33844.3	34260.5	-1.9(-2.0,-1.8)	1802340.3	21815.3	22163.1	-2.1(-2.3,-1.8)	3747742.4	46057.7	46629.5	-1.8(-1.9,-1.8)
C 么 te d'Ivoire	Overall nutritional deficiencies	366321.1	1399.7	1122.0	-0.9(-1.1,-0.8)	161810.2	1278.0	998.5	-0.9(-1.0,-0.8)	204510.9	1513.7	1237.3	-1.0(-1.1,-0.8)
	Protein-energy malnutrition	351620.1	1343.5	1072.2	-0.9(-1.0,-0.7)	150615.8	1189.6	998.5	-0.8(-0.8,-0.7)	201004.2	1487.8	1213.3	-1.0(-1.1,-0.8)
	Iodine deficiency	14701.0	56.2	49.8	-1.9(-2.1,-1.7)	11194.4	88.4	76.7	-2.0(-2.2,-1.8)	3506.7	26.0	24.1	-1.3(-1.5,-1.1)
	Vitamin A deficiency	3608170.5	13786.6	14240.0	-3.2(-3.3,-3.0)	1714405.7	13540.7	13920.6	-2.8(-3.0,-2.6)	1893764.8	14017.1	14540.5	-3.5(-3.6,-3.4)
Gambia	Overall nutritional deficiencies	43121.5	1920.0	1620.0	-0.9(-1.1,-0.7)	16604.5	1455.2	1225.4	-1.2(-1.4,-1.1)	26517.0	2400.1	2017.7	-0.6(-0.9,-0.4)
	Protein-energy malnutrition	39220.8	1746.4	1478.4	-0.8(-1.1,-0.6)	14055.6	1231.8	1225.4	-1.2(-1.4,-1.0)	25165.2	2277.8	1916.7	-0.6(-0.9,-0.3)
	Iodine deficiency	3900.7	173.7	141.6	-1.4(-1.8,-1.1)	2548.9	223.4	179.8	-1.4(-1.7,-1.1)	1351.8	122.4	101.0	-1.5(-1.9,-1.2)
	Vitamin A deficiency	499975.6	22262.0	22716.8	-2.9(-3.0,-2.9)	177918.6	15592.6	16201.8	-2.7(-2.8,-2.7)	322057.0	29150.1	29437.9	-3.0(-3.1,-3.0)
Ghana	Overall nutritional deficiencies	428405.7	1358.5	1236.8	-1.4(-1.6,-1.3)	200691.5	1236.8	1129.1	-1.7(-2.0,-1.5)	227714.1	1487.3	1362.2	-1.1(-1.3,-1.0)

	Protein-energy malnutrition	394624.8	1251.3	1144.3	-1.3(-1.5,-1.1)	178734.0	1101.5	1129.1	-1.6(-1.8,-1.3)	215890.8	1410.1	1295.4	-1.0(-1.2,-0.9)
	Iodine deficiency	33780.8	107.1	92.5	-2.8(-3.0,-2.6)	21957.6	135.3	117.0	-2.8(-3.0,-2.6)	11823.3	77.2	66.8	-2.9(-3.1,-2.7)
	Vitamin A deficiency	4824126.0	15297.1	15915.7	-3.7(-3.9,-3.5)	1500803.0	9249.3	9832.0	-3.8(-4.1,-3.6)	3323323.0	21706.6	22256.1	-3.6(-3.8,-3.5)
Guinea	Overall nutritional deficiencies	200936.6	1589.3	1172.6	-1.0(-1.2,-0.9)	96423.5	1479.8	1109.7	-1.3(-1.4,-1.2)	104513.1	1705.7	1236.9	-0.8(-1.1,-0.5)
	Protein-energy malnutrition	184076.8	1455.9	1059.2	-0.7(-0.8,-0.5)	85924.6	1318.7	1109.7	-0.9(-1.0,-0.8)	98152.2	1601.9	1148.4	-0.4(-0.7,-0.1)
	Iodine deficiency	16859.8	133.4	113.4	-3.4(-3.7,-3.2)	10498.8	161.1	135.4	-3.3(-3.6,-3.0)	6361.0	103.8	88.5	-3.6(-3.9,-3.4)
	Vitamin A deficiency	3036286.2	24015.3	24602.8	-2.5(-2.5,-2.4)	1014619.5	15571.4	16071.0	-2.2(-2.3,-2.1)	2021666.7	32994.8	33671.4	-2.6(-2.7,-2.4)
Guinea-Bissau	Overall nutritional deficiencies	27929.6	1469.1	1194.1	-1.3(-1.5,-1.2)	12770.0	1307.8	1073.5	-1.3(-1.4,-1.1)	15159.6	1639.4	1323.2	-1.4(-1.5,-1.3)
	Protein-energy malnutrition	24633.8	1295.7	1052.3	-1.3(-1.4,-1.2)	10566.7	1082.1	1073.5	-1.1(-1.3,-1.0)	14067.1	1521.2	1226.4	-1.4(-1.5,-1.2)
	Iodine deficiency	3295.8	173.4	141.8	-1.7(-1.8,-1.5)	2203.3	225.6	184.2	-1.7(-1.9,-1.5)	1092.4	118.1	96.7	-1.6(-1.7,-1.4)
	Vitamin A deficiency	510801.6	26867.4	27544.1	-2.4(-2.5,-2.4)	163893.5	16784.3	17406.2	-2.3(-2.4,-2.2)	346908.2	37514.7	38143.9	-2.5(-2.6,-2.4)
Liberia	Overall nutritional deficiencies	51081.6	1066.4	967.7	-1.5(-1.7,-1.4)	21758.5	915.1	845.6	-1.6(-1.8,-1.5)	29323.0	1215.6	1084.9	-1.5(-1.7,-1.3)
	Protein-energy malnutrition	49107.1	1025.2	931.1	-1.6(-1.7,-1.4)	20517.8	862.9	845.6	-1.7(-1.8,-1.5)	28589.3	1185.2	1057.2	-1.5(-1.7,-1.3)
	Iodine deficiency	1974.5	41.2	36.7	-1.1(-1.5,-0.7)	1240.8	52.2	45.6	-1.0(-1.4,-0.6)	733.7	30.4	27.7	-1.1(-1.5,-0.7)
	Vitamin A deficiency	640225.3	13366.1	13461.1	-3.7(-4.0,-3.4)	132195.2	5559.8	5679.7	-3.4(-4.0,-2.8)	508030.1	21060.9	21171.2	-3.8(-4.1,-3.6)
Mali	Overall nutritional deficiencies	626808.2	2859.9	1869.5	-0.8(-0.9,-0.6)	282821.6	2564.4	1609.1	0.0(-0.2,0.2)	343986.6	3159.1	2128.8	-1.3(-1.5,-1.0)
	Protein-energy malnutrition	616320.4	2812.0	1826.4	-0.8(-0.9,-0.6)	276129.4	2503.7	1609.1	0.0(-0.2,0.2)	340190.9	3124.2	2096.7	-1.3(-1.5,-1.0)
	Iodine deficiency	10487.9	47.9	43.0	-1.6(-1.7,-1.5)	6692.2	60.7	53.4	-1.5(-1.6,-1.4)	3795.7	34.9	32.1	-1.6(-1.7,-1.5)
	Vitamin A deficiency	6550941.6	29889.1	30561.2	-2.3(-2.4,-2.3)	2399097.2	21753.2	22468.3	-2.0(-2.1,-1.9)	4151844.4	38129.7	38781.3	-2.5(-2.6,-2.5)
Mauritania	Overall nutritional deficiencies	67829.6	1689.7	1510.9	-1.2(-1.4,-1.1)	31900.1	1563.2	1436.6	-1.1(-1.5,-0.8)	35929.4	1820.5	1580.3	-1.4(-1.5,-1.2)
	Protein-energy malnutrition	61159.4	1523.5	1372.0	-1.2(-1.4,-1.0)	27615.6	1353.2	1436.6	-1.0(-1.4,-0.7)	33543.8	1699.6	1478.6	-1.4(-1.5,-1.2)
	Iodine deficiency	6670.2	166.2	138.9	-1.6(-1.9,-1.4)	4284.5	210.0	173.9	-1.6(-1.8,-1.3)	2385.6	120.9	101.7	-1.7(-2.0,-1.5)
	Vitamin A deficiency	404040.8	10065.1	10418.3	-3.8(-3.9,-3.7)	146452.9	7176.6	7516.9	-3.2(-3.4,-3.1)	257587.9	13051.8	13417.4	-4.1(-4.2,-4.0)
Niger	Overall nutritional deficiencies	654823.3	2811.0	1637.2	-1.0(-1.2,-0.9)	313827.4	2675.5	1556.6	-1.0(-1.2,-0.9)	340995.9	2948.3	1720.8	-1.0(-1.2,-0.9)
	Protein-energy malnutrition	619409.1	2658.9	1509.4	-1.1(-1.2,-1.0)	293069.3	2498.5	1556.6	-1.1(-1.3,-0.9)	326339.8	2821.6	1615.2	-1.1(-1.2,-0.9)
	Iodine deficiency	35414.3	152.0	127.8	-0.4(-0.6,-0.3)	20758.2	177.0	149.1	-0.3(-0.5,-0.1)	14656.1	126.7	105.6	-0.5(-0.7,-0.4)
	Vitamin A deficiency	10054063.4	43159.1	43501.5	-1.2(-1.3,-1.1)	3669299.8	31282.2	31676.0	-1.0(-1.1,-0.9)	6384763.6	55204.3	55451.4	-1.3(-1.4,-1.2)
Nigeria	Overall nutritional deficiencies	4935329.1	2297.4	1795.0	-0.4(-0.7,-0.2)	1859842.4	1667.2	1295.8	-0.7(-0.9,-0.4)	3075486.7	2978.2	2325.6	-0.2(-0.5,0.1)
	Protein-energy malnutrition	4795462.0	2232.3	1738.0	-0.4(-0.6,-0.1)	1760978.9	1578.5	1295.8	-0.6(-0.9,-0.3)	3034483.1	2938.5	2289.8	-0.2(-0.5,0.1)
	Iodine deficiency	139867.1	65.1	57.0	-1.6(-2.0,-1.1)	98863.5	88.6	76.2	-2.0(-2.6,-1.4)	41003.6	39.7	35.9	-0.6(-0.6,-0.5)
	Vitamin A deficiency	11240747.1	5232.5	5128.4	-4.4(-4.9,-3.8)	6416728.1	5751.9	5665.1	-2.9(-3.8,-2.1)	4824018.9	4671.5	4550.9	-5.7(-6.1,-5.3)
Sao Tome and Principe	Overall nutritional deficiencies	2300.3	1120.0	1092.4	-1.3(-1.5,-1.1)	1030.5	1006.6	982.0	-1.4(-1.6,-1.2)	1269.9	1232.7	1202.4	-1.3(-1.5,-1.1)
	Protein-energy malnutrition	2204.8	1073.5	1051.2	-1.3(-1.5,-1.1)	970.4	947.9	982.0	-1.4(-1.6,-1.2)	1234.5	1198.3	1171.4	-1.3(-1.5,-1.1)

	Iodine deficiency	95.5	46.5	41.3	-0.9(-1.1,-0.7)	60.1	58.7	51.6	-0.9(-1.1,-0.7)	35.4	34.4	31.0	-0.9(-1.1,-0.7)
	Vitamin A deficiency	26275.2	12793.1	13261.5	-4.6(-4.7,-4.5)	7593.3	7417.4	7787.0	-4.3(-4.5,-4.1)	18681.9	18135.4	18688.4	-4.7(-4.8,-4.6)
Senegal	Overall nutritional deficiencies	196672.3	1299.5	1106.0	-0.9(-1.0,-0.8)	97546.9	1292.5	1095.8	-1.1(-1.2,-0.9)	99125.4	1306.5	1118.6	-0.8(-0.9,-0.7)
	Protein-energy malnutrition	183171.1	1210.3	1029.1	-0.8(-0.9,-0.8)	89650.0	1187.9	1095.8	-1.0(-1.2,-0.8)	93521.1	1232.6	1055.2	-0.7(-0.8,-0.6)
	Iodine deficiency	13501.2	89.2	76.9	-1.9(-2.1,-1.7)	7896.9	104.6	90.6	-1.8(-2.0,-1.6)	5604.3	73.9	63.4	-2.0(-2.3,-1.8)
	Vitamin A deficiency	2127531.2	14057.9	14427.5	-4.3(-4.8,-3.9)	574845.9	7616.8	7814.3	-3.7(-4.0,-3.4)	1552685.3	20465.0	20931.3	-4.6(-5.2,-4.1)
Sierra Leone	Overall nutritional deficiencies	129774.9	1566.4	1252.7	-1.2(-1.3,-1.1)	58722.2	1403.9	1136.1	-1.3(-1.4,-1.2)	71052.7	1732.1	1367.5	-1.1(-1.3,-1.0)
	Protein-energy malnutrition	123886.8	1495.4	1191.3	-1.2(-1.3,-1.1)	54916.9	1313.0	1136.1	-1.3(-1.4,-1.2)	68969.9	1681.3	1322.5	-1.1(-1.3,-1.0)
	Iodine deficiency	5888.1	71.1	61.4	-0.9(-1.3,-0.5)	3805.3	91.0	77.1	-0.8(-1.2,-0.4)	2082.8	50.8	45.0	-0.9(-1.3,-0.5)
	Vitamin A deficiency	1884541.2	22747.1	23318.3	-2.6(-2.8,-2.5)	622039.7	14871.9	15365.4	-2.3(-2.5,-2.0)	1262501.5	30776.9	31458.2	-2.9(-3.0,-2.8)
Togo	Overall nutritional deficiencies	116131.0	1466.0	1281.1	-1.0(-1.2,-0.9)	51521.4	1274.1	1125.0	-1.1(-1.3,-1.0)	64609.6	1666.2	1450.9	-0.9(-1.1,-0.7)
	Protein-energy malnutrition	107751.3	1360.2	1189.3	-0.9(-1.1,-0.8)	46363.7	1146.6	1125.0	-1.0(-1.1,-0.9)	61387.5	1583.1	1379.7	-0.8(-1.0,-0.6)
	Iodine deficiency	8379.7	105.8	91.8	-2.0(-2.4,-1.7)	5157.7	127.5	112.1	-1.9(-2.2,-1.6)	3222.1	83.1	71.2	-2.2(-2.5,-1.9)
	Vitamin A deficiency	1443362.6	18220.8	18885.7	-2.8(-3.0,-2.7)	431855.5	10679.6	11191.0	-2.4(-2.6,-2.2)	1011507.1	26084.8	26750.8	-3.0(-3.2,-2.9)
American Samoa	Overall nutritional deficiencies	753.9	1358.3	1431.7	-0.3(-0.4,-0.1)	403.0	1462.1	1538.3	-0.3(-0.4,-0.3)	350.9	1255.8	1313.3	-0.2(-0.4,0.0)
	Protein-energy malnutrition	752.3	1355.4	1428.9	-0.3(-0.4,-0.1)	402.1	1458.6	1538.3	-0.3(-0.4,-0.3)	350.2	1253.6	1311.1	-0.2(-0.4,0.0)
	Iodine deficiency	1.6	2.8	2.8	-1.2(-1.3,-1.1)	0.9	3.4	3.4	-1.0(-1.1,-0.9)	0.6	2.3	2.2	-1.5(-1.6,-1.3)
	Vitamin A deficiency	3182.5	5733.8	6562.3	-1.9(-2.1,-1.6)	1243.1	4509.5	5138.5	-2.0(-2.2,-1.8)	1939.4	6941.8	7948.5	-1.8(-2.0,-1.5)
Tuvalu	Overall nutritional deficiencies	140.6	1192.0	1265.0	-0.7(-0.8,-0.6)	69.9	1231.8	1288.8	-0.6(-0.7,-0.5)	70.7	1155.1	1236.7	-0.7(-0.8,-0.6)
	Protein-energy malnutrition	140.2	1188.0	1261.2	-0.7(-0.8,-0.6)	69.6	1227.0	1288.8	-0.6(-0.7,-0.5)	70.6	1152.0	1233.7	-0.7(-0.8,-0.6)
	Iodine deficiency	0.5	4.0	3.9	-2.6(-2.7,-2.4)	0.3	4.8	4.7	-2.3(-2.5,-2.2)	0.2	3.1	3.1	-2.8(-3.0,-2.6)
	Vitamin A deficiency	1556.6	13193.7	14979.9	-1.9(-2.0,-1.7)	527.6	9300.5	10575.8	-2.0(-2.2,-1.9)	1028.9	16799.7	18956.0	-1.9(-2.1,-1.7)
South Sudan	Overall nutritional deficiencies	217076.1	2338.4	1711.3	-1.4(-1.6,-1.3)	108540.1	2347.7	1689.3	-1.1(-1.2,-1.0)	108536.0	2329.3	1734.5	-1.7(-1.9,-1.5)
	Protein-energy malnutrition	208824.0	2249.5	1634.2	-1.5(-1.7,-1.3)	103650.5	2241.9	1689.3	-1.2(-1.3,-1.0)	105173.4	2257.1	1671.3	-1.8(-2.0,-1.5)
	Iodine deficiency	8252.1	88.9	77.1	0.4(0.2,0.5)	4889.6	105.8	90.5	0.3(0.2,0.5)	3362.6	72.2	63.2	0.4(0.2,0.5)
	Vitamin A deficiency	1917038.2	20651.1	20173.6	-2.3(-2.5,-2.2)	786254.9	17006.4	16805.0	-2.1(-2.2,-2.0)	1130783.3	24267.5	23506.3	-2.5(-2.6,-2.3)
Sudan	Overall nutritional deficiencies	1142091.9	2798.7	2338.0	-0.8(-1.2,-0.4)	492538.2	2447.1	2064.1	-1.2(-1.6,-0.8)	649553.7	3140.8	2601.0	-0.4(-0.9,0.0)
	Protein-energy malnutrition	1112322.4	2725.7	2277.1	-0.7(-1.2,-0.3)	475601.6	2363.0	2064.1	-1.2(-1.6,-0.8)	636720.8	3078.8	2549.2	-0.4(-0.8,0.1)
	Iodine deficiency	29769.5	72.9	60.8	-2.6(-3.0,-2.1)	16936.6	84.1	70.2	-2.5(-2.9,-2.1)	12832.9	62.1	51.8	-2.7(-3.1,-2.2)
	Vitamin A deficiency	3681594.6	9021.7	9067.5	-4.5(-4.8,-4.2)	1519548.6	7549.7	7602.5	-4.3(-4.6,-4.0)	2162045.9	10454.2	10498.3	-4.7(-5.0,-4.4)

Table S6. Sex-specific disability-adjusted life-years (DALYs) counts, crude rate, age-standardized rate in 2019 and the estimated annual percentage change (EAPC) for age-standardized rate of nutritional deficiencies and its main subcategories from 1990 to 2019 at national level in low-and middle-income countries (LMICs).

Country	Cause	Both				Female				Male			
		Number	Crude rate	ASDR	EAPC	Number	Crude rate	ASDR	EAPC	Number	Crude rate	ASDR	EAPC
China	Overall nutritional deficiencies	2606925.9	183.3	174.8	-4.9(-5.3,-4.6)	1350572.2	193.6	177.8	-5.6(-5.9,-5.2)	1256353.7	173.3	173.5	-4.2(-4.5,-3.8)
	Protein-energy malnutrition	790443.6	55.6	54.6	-6.5(-7.3,-5.8)	261299.5	37.5	36.9	-8.6(-9.4,-7.8)	529144.1	73.0	72.9	-4.8(-5.5,-4.2)
	Iodine deficiency	264709.4	18.6	15.6	-0.5(-0.9,-0.2)	187078.1	26.8	22.2	0.1(-0.2,0.4)	77631.3	10.7	9.1	-1.7(-2.1,-1.2)
	Vitamin A deficiency	40319.2	2.8	3.7	-4.1(-4.4,-3.8)	15298.8	2.2	2.9	-3.8(-4.1,-3.6)	25020.5	3.5	4.4	-4.3(-4.7,-3.8)
	Dietary iron deficiency	1206621.3	84.8	80.7	-5.0(-5.3,-4.8)	790042.8	113.3	102.6	-5.1(-5.4,-4.9)	416578.5	57.5	59.6	-4.9(-5.2,-4.7)
	Other nutritional deficiencies	304832.3	21.4	20.3	-0.2(-0.6,0.3)	96853.1	13.9	13.2	-1.5(-1.9,-1.0)	207979.2	28.7	27.4	0.7(0.2,1.1)
Democratic People's Republic of Korea	Overall nutritional deficiencies	95062.3	362.4	380.9	-9.3(-14.4,-3.9)	62263.4	472.3	478.1	-8.3(-13.1,-3.3)	32799.0	251.3	295.3	-10.4(-15.8,-4.6)
	Protein-energy malnutrition	16658.8	63.5	76.1	-14.2(-20.5,-7.5)	7089.6	53.8	62.3	-14.6(-20.8,-7.9)	9569.2	73.3	92.9	-13.9(-20.2,-7.1)
	Iodine deficiency	571.7	2.2	1.9	-1.8(-3.8,0.2)	344.9	2.6	2.3	-1.4(-3.3,0.5)	226.7	1.7	1.6	-2.3(-4.4,0.0)
	Vitamin A deficiency	1531.1	5.8	8.8	-3.6(-4.0,-3.1)	666.0	5.1	7.8	-2.9(-3.5,-2.3)	865.2	6.6	9.8	-4.0(-4.4,-3.6)
	Dietary iron deficiency	72709.1	277.2	280.1	-0.9(-1.2,-0.6)	52773.8	400.4	395.6	-0.6(-0.9,-0.3)	19935.3	152.7	172.9	-1.2(-1.5,-0.9)
	Other nutritional deficiencies	3591.5	13.7	13.9	-4.0(-4.6,-3.3)	1389.0	10.5	10.3	-3.1(-3.3,-2.8)	2202.5	16.9	18.1	-4.4(-5.4,-3.3)
Cambodia	Overall nutritional deficiencies	108765.2	655.1	687.4	-5.3(-5.6,-5.0)	64356.7	761.5	792.5	-4.4(-4.6,-4.2)	44408.6	544.8	574.1	-6.3(-6.7,-6.0)
	Protein-energy malnutrition	23119.5	139.2	160.6	-9.4(-9.8,-9.0)	10927.9	129.3	146.0	-8.9(-9.3,-8.6)	12191.6	149.6	177.9	-9.7(-10.1,-9.3)
	Iodine deficiency	2352.8	14.2	14.4	-7.2(-8.5,-5.8)	1627.9	19.3	19.2	-6.9(-8.2,-5.5)	724.9	8.9	9.2	-7.7(-9.2,-6.1)
	Vitamin A deficiency	3391.0	20.4	19.3	-4.2(-4.2,-4.1)	1600.2	18.9	18.6	-3.6(-3.7,-3.5)	1790.8	22.0	19.9	-4.6(-4.6,-4.6)
	Dietary iron deficiency	76581.7	461.2	468.1	-1.3(-1.4,-1.2)	48734.9	576.7	588.2	-1.1(-1.3,-1.0)	27846.8	341.6	336.2	-1.5(-1.5,-1.4)
	Other nutritional deficiencies	3320.2	20.0	25.1	-5.2(-5.5,-4.9)	1465.7	17.3	20.6	-3.9(-4.2,-3.6)	1854.5	22.7	30.9	-6.0(-6.3,-5.8)
Indonesia	Overall nutritional deficiencies	1618167.2	623.7	732.3	-2.5(-2.7,-2.4)	955968.5	743.6	831.8	-2.3(-2.4,-2.1)	662198.7	505.9	634.1	-2.9(-3.1,-2.7)
	Protein-energy malnutrition	692134.7	266.8	356.8	-2.7(-3.0,-2.3)	355636.8	276.6	356.5	-2.6(-2.9,-2.2)	336497.8	257.1	358.2	-2.9(-3.2,-2.5)
	Iodine deficiency	10742.2	4.1	3.9	-6.8(-7.8,-5.8)	7249.8	5.6	5.3	-6.8(-7.9,-5.8)	3492.4	2.7	2.5	-6.7(-7.6,-5.8)
	Vitamin A deficiency	42527.2	16.4	17.6	-4.0(-4.1,-3.9)	21610.8	16.8	18.1	-3.3(-3.5,-3.2)	20916.4	16.0	17.1	-4.6(-4.7,-4.5)
	Dietary iron deficiency	746652.1	287.8	299.5	-2.4(-2.5,-2.2)	512207.4	398.4	401.2	-2.0(-2.1,-1.9)	234444.7	179.1	198.0	-3.0(-3.2,-2.9)
	Other nutritional deficiencies	126111.1	48.6	54.4	-0.9(-1.3,-0.5)	59263.6	46.1	50.8	-0.4(-0.8,-0.1)	66847.4	51.1	58.2	-1.2(-1.7,-0.7)
Lao People's Democratic Republic	Overall nutritional deficiencies	40048.2	559.5	600.1	-4.6(-4.8,-4.5)	23613.9	663.2	708.4	-4.0(-4.1,-3.8)	16434.3	456.8	490.4	-5.4(-5.6,-5.3)
	Protein-energy malnutrition	11381.5	159.0	176.1	-7.4(-7.5,-7.2)	5641.1	158.4	175.0	-7.1(-7.2,-7.0)	5740.4	159.6	176.7	-7.7(-7.9,-7.5)

	Iodine deficiency	562.1	7.9	8.1	-5.6(-7.2,-4.0)	380.9	10.7	10.9	-5.5(-7.1,-3.9)	181.2	5.0	5.3	-5.7(-7.3,-4.1)
	Vitamin A deficiency	1215.6	17.0	15.4	-4.0(-4.2,-3.7)	566.9	15.9	14.7	-3.4(-3.7,-3.1)	648.7	18.0	16.2	-4.4(-4.6,-4.1)
	Dietary iron deficiency	25317.7	353.7	373.3	-1.7(-1.7,-1.6)	16387.9	460.3	485.2	-1.3(-1.3,-1.2)	8929.8	248.2	260.2	-2.3(-2.4,-2.2)
	Other nutritional deficiencies	1571.3	22.0	27.3	-3.7(-3.9,-3.4)	637.1	17.9	22.6	-2.7(-2.9,-2.6)	934.2	26.0	32.0	-4.3(-4.6,-4.0)
Malaysia	Overall nutritional deficiencies	119843.9	382.9	401.2	-2.1(-2.2,-2.0)	64683.4	428.2	441.1	-2.3(-2.4,-2.2)	55160.5	340.6	364.7	-1.8(-1.9,-1.7)
	Protein-energy malnutrition	21906.9	70.0	79.8	-1.1(-1.3,-1.0)	9140.3	60.5	70.0	-1.1(-1.2,-1.0)	12766.6	78.8	88.9	-1.2(-1.4,-1.0)
	Iodine deficiency	4578.0	14.6	13.7	-6.2(-6.8,-5.5)	3099.6	20.5	19.2	-5.9(-6.4,-5.4)	1478.4	9.1	8.5	-6.5(-7.4,-5.7)
	Vitamin A deficiency	125.5	0.4	0.5	-6.3(-6.9,-5.8)	69.0	0.5	0.5	-5.3(-5.9,-4.8)	56.6	0.3	0.4	-7.3(-7.8,-6.8)
	Dietary iron deficiency	88538.4	282.9	292.4	-2.0(-2.0,-1.9)	50772.9	336.1	340.7	-2.2(-2.2,-2.1)	37765.5	233.2	248.0	-1.7(-1.7,-1.6)
	Other nutritional deficiencies	4695.1	15.0	14.9	-1.5(-2.1,-0.9)	1601.6	10.6	10.6	-1.1(-1.7,-0.6)	3093.4	19.1	18.9	-1.8(-2.4,-1.2)
Maldives	Overall nutritional deficiencies	2268.0	455.0	484.2	-4.9(-5.3,-4.4)	1006.2	507.4	506.0	-5.4(-5.8,-4.9)	1261.8	420.4	478.6	-4.2(-4.7,-3.7)
	Protein-energy malnutrition	860.3	172.6	185.9	-5.4(-6.4,-4.4)	204.1	102.9	112.0	-7.9(-9.0,-6.7)	656.2	218.6	239.0	-3.8(-4.5,-3.0)
	Iodine deficiency	25.3	5.1	4.5	-6.0(-7.3,-4.6)	13.5	6.8	6.4	-5.9(-7.3,-4.5)	11.8	3.9	3.3	-5.5(-6.9,-4.1)
	Vitamin A deficiency	28.5	5.7	7.0	-7.8(-8.4,-7.3)	14.0	7.0	7.3	-6.8(-7.2,-6.3)	14.5	4.8	6.7	-8.7(-9.3,-8.1)
	Dietary iron deficiency	1060.8	212.8	227.6	-4.3(-4.5,-4.1)	717.7	362.0	347.7	-3.8(-4.0,-3.5)	343.1	114.3	152.8	-4.6(-4.8,-4.3)
	Other nutritional deficiencies	293.1	58.8	59.3	-4.6(-5.8,-3.4)	56.9	28.7	32.7	-6.9(-8.0,-5.7)	236.3	78.7	76.8	-3.5(-4.6,-2.4)
Myanmar	Overall nutritional deficiencies	369394.7	675.6	708.4	-2.9(-3.0,-2.8)	216461.6	762.8	780.6	-1.9(-2.0,-1.8)	152933.1	581.5	628.0	-4.0(-4.1,-3.9)
	Protein-energy malnutrition	53726.0	98.3	113.7	-6.7(-6.9,-6.6)	20329.7	71.6	80.4	-5.6(-5.7,-5.4)	33396.3	127.0	155.2	-7.1(-7.3,-7.0)
	Iodine deficiency	5380.3	9.8	9.7	-6.8(-8.4,-5.2)	3774.1	13.3	12.8	-6.5(-8.0,-5.0)	1606.2	6.1	6.1	-7.5(-9.1,-5.8)
	Vitamin A deficiency	7410.3	13.6	14.4	-5.2(-5.4,-5.0)	3407.3	12.0	13.5	-4.4(-4.6,-4.2)	4003.0	15.2	15.3	-5.8(-6.1,-5.6)
	Dietary iron deficiency	293742.9	537.2	551.4	-0.8(-0.8,-0.8)	185177.8	652.6	659.9	-0.8(-0.8,-0.7)	108565.1	412.8	425.0	-0.9(-1.0,-0.9)
	Other nutritional deficiencies	9135.2	16.7	19.2	-3.2(-3.4,-3.0)	3772.7	13.3	14.1	-2.1(-2.4,-1.8)	5362.5	20.4	26.3	-3.7(-3.8,-3.5)
Philippines	Overall nutritional deficiencies	431583.2	384.9	411.3	-2.9(-3.0,-2.8)	233464.8	422.3	451.9	-2.8(-2.9,-2.7)	198118.4	348.4	369.0	-3.0(-3.2,-2.9)
	Protein-energy malnutrition	144255.9	128.6	144.1	-3.8(-4.0,-3.6)	66190.0	119.7	136.2	-3.7(-3.9,-3.5)	78065.9	137.3	151.2	-4.0(-4.2,-3.8)
	Iodine deficiency	36246.7	32.3	33.3	-0.5(-1.2,0.1)	21529.0	38.9	39.9	-0.4(-1.0,0.3)	14717.7	25.9	26.7	-0.7(-1.3,-0.1)
	Vitamin A deficiency	16143.8	14.4	13.2	-1.5(-1.8,-1.3)	7031.1	12.7	11.8	-1.6(-1.8,-1.3)	9112.7	16.0	14.4	-1.5(-1.8,-1.3)
	Dietary iron deficiency	213747.2	190.6	196.0	-2.5(-2.7,-2.4)	129438.9	234.1	241.6	-2.6(-2.8,-2.5)	84308.3	148.3	149.5	-2.4(-2.5,-2.2)
	Other nutritional deficiencies	21189.6	18.9	24.8	-1.7(-1.9,-1.4)	9275.8	16.8	22.4	-1.4(-1.7,-1.1)	11913.8	21.0	27.2	-1.8(-2.1,-1.5)
Sri Lanka	Overall nutritional deficiencies	89970.5	411.7	417.8	-3.0(-3.2,-2.8)	49122.6	435.0	435.5	-3.2(-3.3,-3.0)	40847.8	386.7	398.3	-2.8(-3.1,-2.5)
	Protein-energy malnutrition	29221.8	133.7	142.9	-2.4(-2.7,-2.0)	8767.7	77.6	89.1	-2.6(-2.8,-2.4)	20454.1	193.7	201.1	-2.2(-2.6,-1.7)
	Iodine deficiency	2259.9	10.3	9.6	-5.5(-5.9,-5.1)	1690.1	15.0	13.8	-5.2(-5.6,-4.9)	569.8	5.4	5.1	-6.5(-7.0,-5.9)
	Vitamin A deficiency	874.8	4.0	4.7	-6.1(-6.2,-5.9)	415.3	3.7	4.5	-4.9(-5.1,-4.7)	459.5	4.4	4.9	-6.9(-7.1,-6.7)
	Dietary iron deficiency	49170.9	225.0	223.4	-3.2(-3.3,-3.1)	36311.8	321.6	311.5	-3.2(-3.3,-3.1)	12859.2	121.7	127.6	-3.4(-3.5,-3.2)

	Other nutritional deficiencies	8443.1	38.6	37.3	-1.5(-2.0,-1.1)	1937.7	17.2	16.6	-1.2(-1.7,-0.8)	6505.3	61.6	59.6	-1.5(-2.0,-1.0)
Thailand	Overall nutritional deficiencies	132743.2	189.3	191.9	-3.4(-3.6,-3.1)	75139.6	209.3	208.3	-3.0(-3.3,-2.8)	57603.6	168.4	174.5	-3.8(-4.1,-3.4)
	Protein-energy malnutrition	34615.3	49.4	48.7	-4.2(-4.7,-3.8)	16609.1	46.3	44.1	-3.5(-3.8,-3.3)	18006.2	52.6	53.4	-4.9(-5.6,-4.3)
	Iodine deficiency	7950.1	11.3	9.7	-4.0(-5.1,-3.0)	5279.1	14.7	12.6	-3.3(-4.3,-2.4)	2671.0	7.8	6.7	-5.1(-6.4,-3.8)
	Vitamin A deficiency	2503.9	3.6	5.2	-4.4(-4.7,-4.1)	1215.6	3.4	5.1	-4.2(-4.6,-3.8)	1288.3	3.8	5.3	-4.5(-4.7,-4.3)
	Dietary iron deficiency	79406.6	113.3	118.1	-2.9(-3.0,-2.7)	48639.5	135.5	138.3	-2.8(-2.9,-2.6)	30767.1	89.9	96.8	-2.9(-3.1,-2.8)
	Other nutritional deficiencies	8267.2	11.8	10.2	-3.3(-3.8,-2.8)	3396.3	9.5	8.2	-3.6(-4.0,-3.2)	4871.0	14.2	12.4	-3.0(-3.6,-2.4)
Timor-Leste	Overall nutritional deficiencies	9468.7	709.4	690.2	-6.5(-6.9,-6.1)	4821.9	731.1	726.8	-5.7(-6.1,-5.3)	4646.8	688.1	655.5	-7.3(-7.7,-6.8)
	Protein-energy malnutrition	3902.5	292.4	294.6	-8.9(-9.4,-8.4)	1835.2	278.3	279.1	-8.3(-8.7,-7.8)	2067.2	306.1	310.1	-9.4(-9.9,-8.9)
	Iodine deficiency	96.9	7.3	8.5	-6.0(-7.5,-4.4)	65.8	10.0	11.5	-5.9(-7.4,-4.3)	31.1	4.6	5.5	-6.1(-7.7,-4.6)
	Vitamin A deficiency	322.9	24.2	18.8	-4.9(-5.3,-4.5)	140.9	21.4	16.8	-4.7(-5.1,-4.3)	182.0	27.0	20.7	-5.1(-5.5,-4.7)
	Dietary iron deficiency	4783.1	358.3	332.3	-1.4(-1.6,-1.2)	2645.1	401.1	392.7	-1.3(-1.5,-1.1)	2138.0	316.6	273.7	-1.5(-1.7,-1.3)
	Other nutritional deficiencies	363.4	27.2	36.1	-5.1(-5.3,-4.8)	134.9	20.5	26.8	-4.4(-4.6,-4.2)	228.5	33.8	45.5	-5.5(-5.7,-5.2)
Viet Nam	Overall nutritional deficiencies	232349.8	241.1	259.4	-3.7(-3.8,-3.6)	133186.0	273.9	286.7	-3.3(-3.4,-3.1)	99163.7	207.7	241.9	-4.2(-4.3,-4.0)
	Protein-energy malnutrition	46731.1	48.5	54.3	-2.4(-2.6,-2.2)	21730.8	44.7	51.2	-0.8(-1.0,-0.7)	25000.3	52.4	58.9	-3.4(-3.7,-3.1)
	Iodine deficiency	14949.7	15.5	13.9	-5.1(-6.0,-4.1)	10678.3	22.0	19.6	-5.1(-6.1,-4.1)	4271.4	8.9	8.1	-4.9(-5.7,-4.0)
	Vitamin A deficiency	2777.4	2.9	3.6	-4.4(-4.8,-4.1)	1468.1	3.0	3.9	-4.4(-4.7,-4.1)	1309.2	2.7	3.3	-4.5(-4.8,-4.1)
	Dietary iron deficiency	136991.6	142.1	152.5	-3.6(-3.7,-3.4)	92202.8	189.6	197.4	-3.6(-3.7,-3.4)	44788.8	93.8	105.5	-3.4(-3.6,-3.3)
	Other nutritional deficiencies	30899.9	32.1	35.1	-5.0(-5.3,-4.7)	7106.0	14.6	14.5	-1.3(-1.8,-0.8)	23793.9	49.8	66.1	-5.4(-5.6,-5.2)
Fiji	Overall nutritional deficiencies	5800.9	636.6	662.2	-0.3(-0.5,-0.2)	3221.5	716.9	741.5	-0.5(-0.7,-0.3)	2579.4	558.4	574.6	-0.1(-0.2,0.1)
	Protein-energy malnutrition	1081.6	118.7	138.0	-1.8(-2.1,-1.5)	498.0	110.8	128.0	-2.3(-2.6,-1.9)	583.6	126.4	145.6	-1.3(-1.5,-1.1)
	Iodine deficiency	5.0	0.6	0.6	-4.9(-6.1,-3.8)	3.0	0.7	0.7	-4.7(-5.8,-3.6)	2.0	0.4	0.4	-5.2(-6.4,-4.1)
	Vitamin A deficiency	118.3	13.0	12.8	-1.6(-1.8,-1.5)	34.9	7.8	7.7	-2.2(-2.3,-2.1)	83.4	18.1	17.6	-1.4(-1.5,-1.2)
	Dietary iron deficiency	4534.0	497.6	503.8	0.2(0.0,0.4)	2652.1	590.2	597.4	0.0(-0.2,0.2)	1881.9	407.4	404.2	0.6(0.3,0.8)
	Other nutritional deficiencies	62.0	6.8	7.1	-1.0(-1.4,-0.6)	33.5	7.4	7.7	-0.9(-1.4,-0.4)	28.5	6.2	6.7	-1.2(-1.5,-0.9)
Kiribati	Overall nutritional deficiencies	1543.9	1301.5	1386.8	-2.4(-2.5,-2.3)	935.1	1546.7	1686.5	-2.4(-2.4,-2.3)	608.7	1046.7	1013.3	-2.7(-2.7,-2.6)
	Protein-energy malnutrition	734.9	619.5	692.4	-3.7(-3.8,-3.5)	421.5	697.2	804.3	-3.7(-3.9,-3.5)	313.4	538.8	540.0	-3.9(-4.0,-3.7)
	Iodine deficiency	2.8	2.4	2.7	-2.6(-3.1,-2.1)	1.8	2.9	3.2	-2.4(-2.9,-1.9)	1.0	1.8	2.1	-2.9(-3.4,-2.3)
	Vitamin A deficiency	50.7	42.7	34.8	-0.1(-0.3,0.0)	20.9	34.6	29.7	-0.1(-0.3,0.1)	29.8	51.2	39.6	-0.1(-0.3,0.0)
	Dietary iron deficiency	735.4	620.0	632.1	-0.3(-0.4,-0.3)	480.0	793.9	824.5	-0.3(-0.4,-0.3)	255.4	439.2	406.1	-0.4(-0.5,-0.4)
	Other nutritional deficiencies	20.0	16.9	24.8	-1.7(-1.9,-1.6)	10.9	18.0	24.8	-1.6(-1.8,-1.5)	9.1	15.7	25.6	-1.8(-1.9,-1.7)
Marshall Islands	Overall nutritional deficiencies	364.7	641.5	682.2	-1.3(-1.5,-1.2)	202.7	730.3	816.4	-1.1(-1.3,-0.9)	162.0	556.8	557.6	-1.5(-1.6,-1.4)
	Protein-energy malnutrition	75.0	132.0	166.2	-2.8(-3.2,-2.5)	39.9	143.8	198.1	-2.3(-2.7,-1.9)	35.1	120.7	137.7	-3.2(-3.5,-2.9)

	Iodine deficiency	0.9	1.6	1.8	-2.7(-3.2,-2.2)	0.5	2.0	2.2	-2.5(-3.0,-1.9)	0.4	1.2	1.4	-2.9(-3.4,-2.5)
	Vitamin A deficiency	18.2	32.0	28.9	-2.2(-2.3,-2.2)	7.1	25.6	23.2	-2.5(-2.5,-2.4)	11.1	38.0	34.3	-2.0(-2.1,-2.0)
	Dietary iron deficiency	266.3	468.5	476.3	-0.5(-0.6,-0.5)	153.0	551.4	583.6	-0.5(-0.6,-0.4)	113.3	389.5	375.5	-0.5(-0.6,-0.5)
	Other nutritional deficiencies	4.3	7.5	9.0	-1.4(-1.9,-0.9)	2.1	7.5	9.3	-1.5(-1.9,-1.0)	2.2	7.4	8.7	-1.3(-1.9,-0.8)
Micronesia (Federated States of)	Overall nutritional deficiencies	578.9	566.9	631.5	-1.7(-1.8,-1.6)	350.1	698.5	780.3	-1.5(-1.5,-1.4)	228.8	440.1	467.0	-2.1(-2.2,-2.0)
	Protein-energy malnutrition	107.5	105.3	148.9	-3.7(-3.9,-3.4)	58.5	116.7	167.7	-3.5(-3.7,-3.2)	49.0	94.3	119.7	-4.1(-4.4,-3.8)
	Iodine deficiency	1.8	1.7	1.9	-2.7(-3.0,-2.4)	1.1	2.1	2.3	-2.5(-2.8,-2.2)	0.7	1.4	1.5	-2.9(-3.3,-2.6)
	Vitamin A deficiency	35.0	34.3	33.5	-1.7(-1.7,-1.6)	14.4	28.8	28.5	-1.6(-1.8,-1.5)	20.5	39.5	38.2	-1.7(-1.7,-1.6)
	Dietary iron deficiency	426.6	417.7	438.0	-0.7(-0.8,-0.7)	271.8	542.3	571.9	-0.6(-0.7,-0.5)	154.8	297.6	299.2	-1.0(-1.1,-1.0)
	Other nutritional deficiencies	8.0	7.9	9.2	-1.0(-1.5,-0.5)	4.3	8.5	9.9	-0.9(-1.4,-0.3)	3.8	7.3	8.5	-1.2(-1.7,-0.8)
Papua New Guinea	Overall nutritional deficiencies	73746.8	747.4	711.9	-0.8(-0.9,-0.7)	41025.7	861.6	877.0	-0.8(-0.9,-0.7)	32721.2	640.9	561.2	-0.8(-0.9,-0.7)
	Protein-energy malnutrition	17586.4	178.2	167.4	-1.6(-1.9,-1.3)	8054.1	169.2	159.4	-1.5(-1.8,-1.3)	9532.4	186.7	175.8	-1.6(-1.9,-1.3)
	Iodine deficiency	170.0	1.7	2.1	-2.9(-3.5,-2.4)	104.0	2.2	2.6	-2.7(-3.2,-2.2)	66.0	1.3	1.6	-3.3(-3.9,-2.7)
	Vitamin A deficiency	2231.0	22.6	17.0	-0.6(-0.9,-0.3)	987.6	20.7	15.8	-0.6(-0.9,-0.3)	1243.3	24.4	18.1	-0.6(-1.0,-0.3)
	Dietary iron deficiency	53044.5	537.6	517.4	-0.5(-0.5,-0.4)	31528.6	662.2	690.8	-0.6(-0.6,-0.5)	21515.8	421.4	358.0	-0.2(-0.3,-0.2)
	Other nutritional deficiencies	714.9	7.2	8.0	-1.4(-1.8,-1.0)	351.3	7.4	8.4	-1.7(-2.2,-1.2)	363.6	7.1	7.8	-1.0(-1.4,-0.7)
Samoa	Overall nutritional deficiencies	877.4	415.1	439.2	-1.0(-1.1,-0.8)	494.0	481.4	522.1	-0.8(-1.0,-0.7)	383.5	352.6	351.3	-1.2(-1.4,-1.0)
	Protein-energy malnutrition	186.3	88.2	110.7	-2.1(-2.3,-1.9)	103.5	100.9	126.9	-1.6(-1.7,-1.5)	82.8	76.1	90.7	-2.7(-2.9,-2.4)
	Iodine deficiency	0.8	0.4	0.4	-1.1(-1.2,-1.1)	0.5	0.5	0.5	-0.9(-0.9,-0.9)	0.3	0.3	0.3	-1.4(-1.5,-1.3)
	Vitamin A deficiency	46.7	22.1	19.3	-0.5(-0.6,-0.3)	17.7	17.2	15.2	-0.5(-0.7,-0.3)	29.0	26.7	23.1	-0.5(-0.6,-0.3)
	Dietary iron deficiency	606.1	286.8	288.6	-0.5(-0.7,-0.4)	353.0	344.1	358.1	-0.6(-0.7,-0.4)	253.1	232.7	218.2	-0.5(-0.6,-0.4)
	Other nutritional deficiencies	37.4	17.7	20.2	-0.8(-1.4,-0.2)	19.2	18.7	21.4	-0.8(-1.4,-0.1)	18.2	16.8	18.9	-0.8(-1.5,-0.2)
Solomon Islands	Overall nutritional deficiencies	5286.6	806.3	786.8	-1.4(-1.5,-1.3)	2769.6	861.9	921.4	-1.2(-1.3,-1.2)	2517.0	752.9	656.3	-1.6(-1.7,-1.6)
	Protein-energy malnutrition	1647.7	251.3	256.2	-2.9(-3.0,-2.7)	854.5	265.9	295.8	-2.6(-2.8,-2.4)	793.2	237.3	220.6	-3.2(-3.4,-3.0)
	Iodine deficiency	11.8	1.8	2.2	-1.0(-1.3,-0.8)	7.3	2.3	2.7	-0.8(-1.0,-0.6)	4.5	1.3	1.7	-1.4(-1.7,-1.1)
	Vitamin A deficiency	342.8	52.3	37.7	-0.9(-1.1,-0.7)	133.0	41.4	30.4	-1.0(-1.2,-0.7)	209.8	62.8	44.4	-0.9(-1.1,-0.7)
	Dietary iron deficiency	3234.1	493.3	480.3	-0.2(-0.3,-0.1)	1752.7	545.4	582.6	-0.3(-0.4,-0.2)	1481.5	443.2	378.7	-0.4(-0.5,-0.3)
	Other nutritional deficiencies	50.3	7.7	10.4	-1.2(-1.6,-0.8)	22.2	6.9	9.8	-1.0(-1.4,-0.7)	28.1	8.4	11.0	-1.4(-1.8,-1.0)
Tonga	Overall nutritional deficiencies	516.6	504.7	517.5	-0.9(-0.9,-0.8)	314.4	611.4	637.0	-0.8(-0.9,-0.8)	202.2	397.0	376.9	-1.0(-1.0,-0.9)
	Protein-energy malnutrition	119.7	117.0	121.5	-2.0(-2.2,-1.8)	69.1	134.4	135.4	-2.2(-2.4,-2.0)	50.6	99.4	101.1	-1.9(-2.1,-1.7)
	Iodine deficiency	0.6	0.6	0.6	-2.9(-3.6,-2.2)	0.4	0.7	0.7	-2.7(-3.4,-2.0)	0.2	0.4	0.5	-3.2(-3.9,-2.4)
	Vitamin A deficiency	14.5	14.2	11.9	-1.6(-1.8,-1.3)	5.0	9.6	8.5	-1.6(-1.8,-1.4)	9.5	18.7	15.1	-1.5(-1.8,-1.3)
	Dietary iron deficiency	370.7	362.2	371.4	-0.3(-0.4,-0.3)	234.0	455.1	479.6	-0.3(-0.4,-0.2)	136.7	268.4	248.9	-0.5(-0.5,-0.4)

	Other nutritional deficiencies	11.1	10.8	12.1	-0.9(-1.4,-0.3)	6.0	11.6	12.7	-0.8(-1.4,-0.3)	5.1	10.0	11.4	-0.9(-1.3,-0.4)
Vanuatu	Overall nutritional deficiencies	2306.1	782.9	815.6	-0.5(-0.6,-0.4)	1403.1	966.0	1062.5	-0.3(-0.4,-0.2)	903.0	604.8	574.7	-0.9(-1.0,-0.8)
	Protein-energy malnutrition	627.2	212.9	232.5	-1.9(-2.2,-1.7)	332.0	228.6	270.8	-1.7(-2.0,-1.4)	295.2	197.7	196.8	-2.2(-2.4,-2.0)
	Iodine deficiency	32.5	11.0	12.5	-1.1(-1.3,-1.0)	19.3	13.3	14.9	-0.9(-1.1,-0.8)	13.1	8.8	10.0	-1.4(-1.6,-1.2)
	Vitamin A deficiency	108.0	36.7	28.3	-0.9(-1.0,-0.7)	38.6	26.6	21.1	-1.1(-1.3,-0.9)	69.4	46.5	35.0	-0.8(-0.9,-0.6)
	Dietary iron deficiency	1515.2	514.4	532.5	0.5(0.3,0.6)	1001.2	689.3	745.3	0.5(0.4,0.6)	513.9	344.2	323.5	0.3(0.1,0.4)
	Other nutritional deficiencies	23.2	7.9	9.9	-1.0(-1.4,-0.5)	12.0	8.2	10.4	-1.0(-1.5,-0.5)	11.3	7.5	9.4	-0.9(-1.3,-0.5)
Armenia	Overall nutritional deficiencies	7991.9	264.7	277.9	-1.9(-2.0,-1.8)	4339.8	277.8	290.3	-1.7(-1.8,-1.5)	3652.1	250.6	264.3	-2.2(-2.4,-2.0)
	Protein-energy malnutrition	360.7	11.9	12.9	-6.3(-7.2,-5.5)	209.7	13.4	15.3	-4.6(-5.1,-4.0)	151.0	10.4	10.7	-8.0(-9.2,-6.8)
	Iodine deficiency	335.8	11.1	11.0	-2.7(-3.4,-2.0)	327.1	20.9	21.1	-2.7(-3.4,-2.0)	8.7	0.6	0.6	-0.6(-0.7,-0.5)
	Vitamin A deficiency	6.8	0.2	0.3	-2.2(-2.7,-1.7)	3.7	0.2	0.4	-1.2(-1.8,-0.6)	3.1	0.2	0.3	-3.2(-3.6,-2.7)
	Dietary iron deficiency	7110.9	235.5	248.3	-0.9(-1.0,-0.8)	3701.0	236.9	247.8	-0.8(-0.9,-0.7)	3409.9	234.0	247.6	-1.1(-1.2,-1.0)
	Other nutritional deficiencies	177.7	5.9	5.4	-10.3(-11.6,-9.1)	98.3	6.3	5.7	-9.6(-10.7,-8.5)	79.4	5.4	5.1	-11.1(-12.6,-9.6)
Azerbaijan	Overall nutritional deficiencies	37275.0	362.6	362.7	-2.2(-2.3,-2.1)	25955.6	505.3	482.3	-1.9(-2.1,-1.8)	11319.4	220.1	240.4	-2.7(-2.8,-2.6)
	Protein-energy malnutrition	2641.0	25.7	30.0	-6.3(-6.8,-5.9)	1396.6	27.2	31.9	-6.2(-6.7,-5.7)	1244.4	24.2	27.8	-6.5(-7.0,-6.0)
	Iodine deficiency	240.0	2.3	2.2	-5.7(-7.1,-4.4)	202.4	3.9	3.8	-5.6(-6.9,-4.3)	37.5	0.7	0.7	-5.8(-7.2,-4.3)
	Vitamin A deficiency	143.9	1.4	1.8	-3.1(-4.0,-2.3)	61.4	1.2	1.6	-2.3(-3.0,-1.6)	82.5	1.6	1.9	-3.7(-4.6,-2.8)
	Dietary iron deficiency	33305.4	324.0	319.1	-1.4(-1.6,-1.2)	23789.2	463.1	434.3	-1.2(-1.4,-1.0)	9516.2	185.1	201.3	-1.8(-2.0,-1.5)
	Other nutritional deficiencies	944.7	9.2	9.5	-4.2(-4.5,-3.9)	505.9	9.8	10.7	-4.9(-5.3,-4.6)	438.7	8.5	8.7	-3.0(-3.3,-2.7)
Georgia	Overall nutritional deficiencies	13244.8	361.4	352.7	-0.5(-0.6,-0.5)	8811.8	461.5	444.3	-0.5(-0.5,-0.4)	4433.0	252.6	258.3	-0.6(-0.7,-0.5)
	Protein-energy malnutrition	633.3	17.3	19.0	-0.1(-0.3,0.1)	334.1	17.5	17.7	0.2(-0.1,0.4)	299.2	17.0	19.8	-0.4(-0.6,-0.3)
	Iodine deficiency	114.7	3.1	3.2	-0.7(-0.8,-0.6)	104.3	5.5	5.8	-0.6(-0.7,-0.6)	10.4	0.6	0.6	-0.4(-0.5,-0.3)
	Vitamin A deficiency	46.3	1.3	1.8	0.0(-0.6,0.7)	22.2	1.2	1.8	0.7(0.1,1.3)	24.1	1.4	1.8	-0.5(-1.1,0.1)
	Dietary iron deficiency	12143.1	331.3	321.1	-0.5(-0.6,-0.5)	8183.1	428.5	411.0	-0.5(-0.5,-0.4)	3960.0	225.6	228.7	-0.5(-0.6,-0.5)
	Other nutritional deficiencies	307.4	8.4	7.7	-1.9(-3.1,-0.7)	168.1	8.8	8.0	-0.3(-1.3,0.8)	139.3	7.9	7.4	-3.3(-4.7,-1.9)
Kazakhstan	Overall nutritional deficiencies	81482.7	443.0	437.8	-1.7(-2.0,-1.5)	54127.8	570.7	558.3	-1.7(-2.0,-1.5)	27354.9	307.1	306.9	-1.7(-2.0,-1.5)
	Protein-energy malnutrition	6865.1	37.3	38.2	-0.7(-0.8,-0.5)	3774.8	39.8	40.3	0.1(-0.2,0.5)	3090.3	34.7	35.9	-1.5(-1.8,-1.1)
	Iodine deficiency	728.7	4.0	4.0	-5.5(-6.4,-4.6)	669.5	7.1	7.1	-5.4(-6.2,-4.5)	59.2	0.7	0.7	-6.4(-7.5,-5.3)
	Vitamin A deficiency	1233.0	6.7	6.8	-3.3(-3.6,-3.1)	445.8	4.7	5.0	-2.7(-3.0,-2.4)	787.2	8.8	8.4	-3.7(-3.9,-3.5)
	Dietary iron deficiency	70382.2	382.7	376.7	-1.8(-2.0,-1.5)	48015.6	506.3	493.6	-1.8(-2.1,-1.5)	22366.6	251.1	249.9	-1.7(-1.9,-1.4)
	Other nutritional deficiencies	2273.7	12.4	12.2	-0.2(-0.6,0.1)	1222.1	12.9	12.3	0.6(0.0,1.2)	1051.6	11.8	11.9	-1.0(-1.5,-0.4)
Kyrgyzstan	Overall nutritional deficiencies	29086.8	445.1	440.0	-1.4(-1.6,-1.2)	19020.6	576.0	571.9	-1.3(-1.4,-1.1)	10066.3	311.4	304.2	-1.7(-1.8,-1.5)
	Protein-energy malnutrition	1010.9	15.5	15.4	-4.2(-4.6,-3.8)	461.6	14.0	13.8	-3.8(-4.1,-3.5)	549.3	17.0	17.3	-4.5(-5.0,-3.9)

	Iodine deficiency	220.1	3.4	3.4	-0.2(-0.3,-0.2)	200.8	6.1	6.0	-0.2(-0.2,-0.1)	19.3	0.6	0.6	-0.6(-0.6,-0.5)
	Vitamin A deficiency	661.2	10.1	9.3	-0.5(-0.7,-0.3)	325.1	9.8	9.2	0.1(-0.2,0.3)	336.2	10.4	9.3	-0.9(-1.1,-0.8)
	Dietary iron deficiency	26916.7	411.9	407.7	-1.3(-1.4,-1.1)	17928.1	542.9	539.7	-1.2(-1.4,-1.0)	8988.6	278.0	271.5	-1.5(-1.6,-1.3)
	Other nutritional deficiencies	277.9	4.3	4.2	-3.8(-4.2,-3.4)	105.0	3.2	3.2	-6.0(-6.4,-5.5)	172.9	5.3	5.4	-1.7(-2.2,-1.2)
Mongolia	Overall nutritional deficiencies	13411.1	395.9	397.1	-2.8(-3.0,-2.5)	6542.0	381.1	382.1	-2.7(-3.1,-2.4)	6869.0	411.1	415.5	-2.8(-3.0,-2.6)
	Protein-energy malnutrition	403.1	11.9	11.5	-2.7(-3.0,-2.5)	252.4	14.7	14.3	-0.6(-0.8,-0.5)	150.8	9.0	8.7	-4.8(-5.2,-4.4)
	Iodine deficiency	232.8	6.9	6.9	-4.3(-4.7,-3.9)	202.4	11.8	11.8	-4.3(-4.7,-4.0)	30.4	1.8	1.9	-4.3(-4.7,-3.9)
	Vitamin A deficiency	211.4	6.2	6.0	-2.7(-2.8,-2.5)	98.5	5.7	5.6	-2.1(-2.2,-2.0)	112.9	6.8	6.5	-3.1(-3.3,-2.9)
	Dietary iron deficiency	11761.2	347.2	349.7	-2.5(-2.7,-2.2)	5910.3	344.3	345.9	-2.8(-3.1,-2.4)	5850.9	350.1	355.4	-2.1(-2.3,-1.9)
	Other nutritional deficiencies	802.6	23.7	22.9	-5.5(-5.8,-5.3)	78.5	4.6	4.5	-0.8(-1.3,-0.3)	724.1	43.3	43.0	-5.7(-5.9,-5.4)
Tajikistan	Overall nutritional deficiencies	41615.0	438.4	438.3	-1.5(-2.1,-1.0)	23277.1	495.9	498.8	-1.4(-1.9,-0.9)	18337.9	382.1	378.7	-1.7(-2.3,-1.1)
	Protein-energy malnutrition	3303.6	34.8	29.2	-1.8(-3.1,-0.4)	1873.3	39.9	33.9	-2.3(-3.9,-0.6)	1430.3	29.8	24.7	-0.9(-1.6,-0.3)
	Iodine deficiency	1249.5	13.2	13.3	-1.7(-2.7,-0.6)	1139.7	24.3	24.2	-1.5(-2.6,-0.5)	109.8	2.3	2.5	-2.2(-3.5,-0.9)
	Vitamin A deficiency	1346.2	14.2	12.2	-1.5(-1.8,-1.1)	646.0	13.8	12.0	-0.9(-1.2,-0.5)	700.1	14.6	12.4	-1.9(-2.3,-1.5)
	Dietary iron deficiency	33817.7	356.3	365.0	-0.9(-1.0,-0.8)	19227.3	409.7	420.2	-0.8(-0.9,-0.7)	14590.4	304.0	310.7	-1.0(-1.1,-0.9)
	Other nutritional deficiencies	1898.1	20.0	18.6	-6.1(-8.4,-3.8)	390.7	8.3	8.5	-8.5(-11.0,-6.0)	1507.3	31.4	28.4	-5.1(-7.4,-2.8)
Turkmenistan	Overall nutritional deficiencies	18543.3	364.8	362.0	-1.7(-1.8,-1.6)	10855.3	436.5	430.2	-1.6(-1.7,-1.5)	7688.0	296.2	300.0	-1.8(-1.9,-1.7)
	Protein-energy malnutrition	1273.9	25.1	24.0	-2.7(-2.8,-2.6)	599.0	24.1	23.1	-2.9(-3.1,-2.7)	674.9	26.0	24.9	-2.5(-2.6,-2.4)
	Iodine deficiency	157.1	3.1	3.1	-0.9(-1.0,-0.9)	142.4	5.7	5.7	-0.8(-0.9,-0.7)	14.7	0.6	0.6	-0.5(-0.6,-0.4)
	Vitamin A deficiency	413.7	8.1	7.8	-1.4(-1.5,-1.3)	199.5	8.0	7.8	-1.0(-1.1,-0.9)	214.2	8.3	7.8	-1.7(-1.9,-1.6)
	Dietary iron deficiency	16299.2	320.7	319.3	-1.4(-1.6,-1.3)	9731.1	391.3	386.3	-1.4(-1.5,-1.2)	6568.1	253.0	258.2	-1.4(-1.6,-1.3)
	Other nutritional deficiencies	399.4	7.9	7.8	-6.3(-6.6,-5.9)	183.3	7.4	7.3	-5.7(-6.2,-5.3)	216.1	8.3	8.4	-6.7(-7.0,-6.3)
Uzbekistan	Overall nutritional deficiencies	224062.3	665.3	649.1	-1.3(-1.4,-1.2)	151110.0	895.8	870.8	-1.2(-1.3,-1.1)	72952.3	434.0	425.3	-1.4(-1.4,-1.3)
	Protein-energy malnutrition	13804.5	41.0	39.1	-1.7(-1.9,-1.5)	7193.7	42.6	41.8	-1.6(-1.8,-1.4)	6610.8	39.3	36.7	-1.8(-2.0,-1.5)
	Iodine deficiency	3772.4	11.2	11.1	-3.0(-3.4,-2.5)	3432.7	20.3	19.9	-2.9(-3.3,-2.4)	339.8	2.0	2.1	-3.6(-4.2,-3.0)
	Vitamin A deficiency	3178.3	9.4	8.9	-2.3(-2.4,-2.2)	1584.0	9.4	9.1	-1.4(-1.6,-1.3)	1594.3	9.5	8.8	-2.9(-3.0,-2.9)
	Dietary iron deficiency	199840.9	593.4	579.7	-1.0(-1.1,-0.8)	137170.3	813.2	789.8	-1.0(-1.1,-0.8)	62670.6	372.9	367.3	-0.9(-1.0,-0.8)
	Other nutritional deficiencies	3466.3	10.3	10.3	-6.8(-7.2,-6.4)	1729.4	10.3	10.2	-6.6(-6.9,-6.2)	1736.9	10.3	10.3	-7.0(-7.5,-6.5)
Albania	Overall nutritional deficiencies	6697.2	246.2	270.2	-2.4(-2.6,-2.1)	4390.6	323.3	342.4	-2.1(-2.4,-1.8)	2306.6	169.3	196.3	-3.0(-3.2,-2.7)
	Protein-energy malnutrition	1220.6	44.9	50.1	-4.1(-4.8,-3.3)	716.0	52.7	59.6	-3.4(-4.2,-2.7)	504.5	37.0	41.0	-4.8(-5.6,-4.0)
	Iodine deficiency	57.7	2.1	2.0	-5.8(-6.7,-4.9)	36.8	2.7	2.6	-5.6(-6.5,-4.8)	20.9	1.5	1.5	-6.1(-7.0,-5.2)
	Vitamin A deficiency	129.7	4.8	7.9	-4.4(-4.7,-4.0)	67.1	4.9	8.5	-3.8(-4.2,-3.5)	62.6	4.6	7.2	-4.9(-5.2,-4.5)
	Dietary iron deficiency	4861.4	178.7	194.8	-1.8(-2.0,-1.6)	3309.7	243.7	252.5	-1.7(-1.9,-1.5)	1551.6	113.9	134.7	-2.2(-2.3,-2.0)

	Other nutritional deficiencies	427.9	15.7	15.4	0.3(-0.3,0.9)	261.0	19.2	19.1	0.8(0.2,1.5)	166.9	12.2	11.9	-0.5(-1.0,0.1)
Bosnia and Herzegovina	Overall nutritional deficiencies	7158.8	216.9	234.3	-1.8(-2.1,-1.5)	4308.1	254.9	266.2	-1.8(-2.1,-1.5)	2850.7	177.1	202.7	-1.9(-2.1,-1.6)
	Protein-energy malnutrition	1125.2	34.1	33.2	0.8(0.7,0.9)	516.0	30.5	30.3	0.5(0.4,0.6)	609.2	37.8	36.3	1.1(1.0,1.2)
	Iodine deficiency	145.1	4.4	4.2	-6.6(-7.6,-5.5)	102.1	6.0	5.7	-6.3(-7.3,-5.3)	43.0	2.7	2.6	-7.0(-8.1,-6.0)
	Vitamin A deficiency	73.5	2.2	4.6	-5.1(-5.6,-4.7)	40.8	2.4	5.2	-4.5(-5.0,-4.1)	32.7	2.0	4.0	-5.8(-6.2,-5.3)
	Dietary iron deficiency	5336.9	161.7	179.2	-1.9(-2.2,-1.7)	3429.1	202.9	213.0	-1.8(-2.1,-1.5)	1907.9	118.5	145.6	-2.2(-2.4,-1.9)
	Other nutritional deficiencies	478.2	14.5	13.1	0.5(-0.1,1.2)	220.1	13.0	11.9	0.1(-0.5,0.7)	258.1	16.0	14.2	0.9(0.2,1.7)
Bulgaria	Overall nutritional deficiencies	12961.9	186.9	215.0	-0.8(-0.8,-0.7)	7880.7	220.9	246.8	-0.6(-0.7,-0.6)	5081.2	150.9	184.7	-0.9(-1.0,-0.8)
	Protein-energy malnutrition	1529.5	22.1	25.5	0.6(0.4,0.9)	725.8	20.3	24.1	0.3(0.1,0.6)	803.7	23.9	27.0	0.9(0.7,1.2)
	Iodine deficiency	113.3	1.6	1.5	-4.4(-5.4,-3.3)	86.7	2.4	2.3	-4.3(-5.3,-3.2)	26.5	0.8	0.7	-4.5(-5.7,-3.3)
	Vitamin A deficiency	148.9	2.1	4.4	-2.3(-2.5,-2.0)	79.2	2.2	4.8	-1.8(-2.0,-1.7)	69.6	2.1	4.1	-2.7(-3.0,-2.4)
	Dietary iron deficiency	10488.5	151.2	173.8	-0.9(-0.9,-0.8)	6654.9	186.5	206.2	-0.7(-0.7,-0.6)	3833.6	113.9	142.7	-1.1(-1.2,-1.0)
	Other nutritional deficiencies	681.8	9.8	9.7	0.5(0.0,0.9)	334.0	9.4	9.3	0.2(-0.3,0.7)	347.8	10.3	10.2	0.7(0.3,1.1)
North Macedonia	Overall nutritional deficiencies	4094.8	190.2	218.8	-1.3(-1.4,-1.2)	2112.0	199.3	223.2	-1.2(-1.3,-1.1)	1982.9	181.4	215.1	-1.4(-1.5,-1.3)
	Protein-energy malnutrition	693.3	32.2	33.3	0.3(0.1,0.4)	319.6	30.2	30.7	-0.2(-0.5,0.1)	373.7	34.2	35.9	0.8(0.7,0.9)
	Iodine deficiency	46.7	2.2	2.0	-1.4(-1.5,-1.3)	29.6	2.8	2.6	-1.3(-1.4,-1.2)	17.1	1.6	1.5	-1.4(-1.6,-1.3)
	Vitamin A deficiency	75.6	3.5	6.4	-3.6(-3.8,-3.4)	32.4	3.1	5.7	-3.4(-3.5,-3.2)	43.2	3.9	7.1	-3.8(-4.0,-3.6)
	Dietary iron deficiency	2998.8	139.3	164.5	-1.5(-1.6,-1.4)	1596.7	150.7	171.9	-1.3(-1.4,-1.2)	1402.1	128.3	158.0	-1.7(-1.8,-1.6)
	Other nutritional deficiencies	280.4	13.0	12.5	0.1(-0.4,0.7)	133.7	12.6	12.4	-0.1(-0.7,0.4)	146.7	13.4	12.7	0.4(-0.1,0.9)
Montenegro	Overall nutritional deficiencies	1116.2	179.9	193.1	-0.7(-0.9,-0.6)	640.3	204.1	215.5	-0.6(-0.7,-0.5)	475.9	155.2	171.4	-0.9(-1.0,-0.8)
	Protein-energy malnutrition	215.4	34.7	36.3	0.7(0.6,0.8)	97.5	31.1	34.6	0.5(0.3,0.6)	117.9	38.5	38.4	0.9(0.8,1.0)
	Iodine deficiency	12.9	2.1	2.0	-1.3(-1.4,-1.2)	8.3	2.7	2.5	-1.2(-1.3,-1.1)	4.6	1.5	1.4	-1.4(-1.5,-1.3)
	Vitamin A deficiency	10.1	1.6	2.7	-2.8(-3.3,-2.2)	5.6	1.8	3.2	-2.2(-2.6,-1.7)	4.5	1.5	2.4	-3.4(-3.9,-2.8)
	Dietary iron deficiency	799.6	128.9	140.4	-1.0(-1.2,-0.9)	493.6	157.3	164.2	-0.8(-0.9,-0.7)	306.0	99.8	116.5	-1.3(-1.5,-1.2)
	Other nutritional deficiencies	78.1	12.6	11.7	0.4(-0.1,0.9)	35.3	11.2	10.9	0.1(-0.4,0.7)	42.9	14.0	12.7	0.7(0.2,1.2)
Serbia	Overall nutritional deficiencies	15451.4	176.7	196.5	-1.7(-1.8,-1.6)	8932.3	202.6	218.5	-1.6(-1.8,-1.5)	6519.1	150.3	174.4	-1.8(-1.9,-1.7)
	Protein-energy malnutrition	2153.3	24.6	26.2	-0.1(-0.2,0.0)	917.5	20.8	21.8	-0.4(-0.6,-0.3)	1235.8	28.5	30.4	0.1(0.0,0.2)
	Iodine deficiency	135.2	1.5	1.5	-1.5(-1.7,-1.4)	68.2	1.5	1.5	-1.5(-1.7,-1.4)	67.0	1.5	1.5	-1.6(-1.7,-1.4)
	Vitamin A deficiency	376.1	4.3	7.7	-3.5(-3.7,-3.3)	168.8	3.8	7.2	-3.3(-3.4,-3.1)	207.2	4.8	8.1	-3.7(-4.0,-3.5)
	Dietary iron deficiency	11954.2	136.7	152.5	-1.9(-2.0,-1.8)	7418.8	168.3	181.0	-1.7(-1.8,-1.6)	4535.4	104.5	124.2	-2.1(-2.2,-2.0)
	Other nutritional deficiencies	832.7	9.5	8.6	-0.6(-1.0,-0.2)	359.0	8.1	7.0	-0.7(-1.0,-0.3)	473.7	10.9	10.2	-0.6(-1.1,-0.2)
Belarus	Overall nutritional deficiencies	16283.4	171.4	158.8	-2.1(-2.4,-1.9)	9856.6	194.3	173.4	-2.1(-2.3,-1.9)	6426.7	145.1	143.3	-2.2(-2.4,-2.0)
	Protein-energy malnutrition	2541.5	26.8	28.3	-1.7(-1.9,-1.5)	1167.1	23.0	24.7	-1.8(-2.1,-1.6)	1374.4	31.0	32.1	-1.6(-1.9,-1.3)

	Iodine deficiency	299.4	3.2	3.0	-1.3(-1.6,-0.9)	217.1	4.3	4.2	-1.2(-1.5,-0.8)	82.3	1.9	1.8	-1.5(-2.0,-1.0)
	Vitamin A deficiency	16.0	0.2	0.3	-4.9(-5.3,-4.6)	8.7	0.2	0.3	-3.9(-4.3,-3.6)	7.4	0.2	0.2	-5.9(-6.2,-5.6)
	Dietary iron deficiency	12238.7	128.8	115.5	-2.2(-2.5,-2.0)	7852.1	154.8	132.8	-2.2(-2.4,-1.9)	4386.6	99.1	96.9	-2.4(-2.7,-2.2)
	Other nutritional deficiencies	1187.8	12.5	11.8	-2.2(-2.4,-2.0)	611.8	12.1	11.4	-2.6(-3.0,-2.2)	576.0	13.0	12.3	-1.8(-2.0,-1.6)
Republic of Moldova	Overall nutritional deficiencies	9582.0	259.8	245.8	-1.8(-1.9,-1.6)	6772.4	351.1	325.5	-1.6(-1.7,-1.4)	2809.6	159.7	162.8	-2.1(-2.2,-2.0)
	Protein-energy malnutrition	525.7	14.3	16.4	-1.8(-2.1,-1.5)	291.9	15.1	19.2	-0.9(-1.1,-0.6)	233.9	13.3	13.8	-2.8(-3.3,-2.4)
	Iodine deficiency	77.1	2.1	1.9	0.3(-0.4,0.9)	50.7	2.6	2.5	0.4(-0.2,1.0)	26.4	1.5	1.4	0.1(-0.6,0.9)
	Vitamin A deficiency	14.0	0.4	0.7	-3.9(-4.3,-3.4)	8.0	0.4	0.8	-3.2(-3.6,-2.7)	6.0	0.3	0.6	-4.6(-5.1,-4.1)
	Dietary iron deficiency	8762.4	237.6	221.2	-1.8(-1.9,-1.6)	6315.2	327.4	297.1	-1.6(-1.8,-1.4)	2447.1	139.1	141.6	-2.1(-2.2,-1.9)
	Other nutritional deficiencies	202.8	5.5	5.6	-1.7(-2.2,-1.1)	106.6	5.5	5.9	-1.9(-2.5,-1.2)	96.2	5.5	5.4	-1.5(-1.9,-1.0)
Russian Federation	Overall nutritional deficiencies	276280.8	188.3	166.2	-1.9(-2.1,-1.6)	172215.5	219.6	186.9	-1.8(-2.1,-1.5)	104065.2	152.3	144.2	-2.0(-2.3,-1.8)
	Protein-energy malnutrition	37927.4	25.9	27.3	-0.8(-1.1,-0.5)	17888.8	22.8	25.3	-0.5(-0.7,-0.2)	20038.6	29.3	29.8	-1.1(-1.4,-0.8)
	Iodine deficiency	5641.2	3.8	3.7	-0.4(-0.8,0.0)	4439.8	5.7	5.6	-0.3(-0.7,0.1)	1201.4	1.8	1.7	-0.5(-1.1,0.0)
	Vitamin A deficiency	36.9	0.0	0.0	-4.1(-4.4,-3.8)	15.8	0.0	0.0	-3.2(-3.5,-2.9)	21.0	0.0	0.0	-4.6(-5.0,-4.3)
	Dietary iron deficiency	216608.0	147.6	124.8	-2.1(-2.4,-1.8)	142067.3	181.2	146.5	-2.0(-2.3,-1.7)	74540.8	109.1	101.3	-2.3(-2.5,-2.0)
	Other nutritional deficiencies	16067.2	11.0	10.3	-1.6(-1.9,-1.3)	7803.7	10.0	9.4	-1.4(-1.6,-1.1)	8263.5	12.1	11.4	-1.8(-2.1,-1.5)
Ukraine	Overall nutritional deficiencies	67444.8	153.1	154.4	-1.9(-2.1,-1.6)	32692.2	137.6	137.7	-1.8(-2.2,-1.5)	34752.6	171.3	173.4	-1.9(-2.1,-1.7)
	Protein-energy malnutrition	12514.9	28.4	38.9	-1.3(-1.6,-0.9)	5224.1	22.0	33.6	-1.5(-2.0,-1.0)	7290.8	35.9	44.5	-1.1(-1.3,-0.9)
	Iodine deficiency	5570.5	12.6	12.6	-0.1(-0.7,0.5)	4034.0	17.0	17.7	0.0(-0.6,0.5)	1536.5	7.6	7.3	-0.3(-1.0,0.4)
	Vitamin A deficiency	62.2	0.1	0.3	-3.1(-3.3,-2.9)	32.5	0.1	0.3	-2.4(-2.6,-2.2)	29.7	0.1	0.3	-3.8(-4.0,-3.5)
	Dietary iron deficiency	43971.8	99.8	89.8	-1.9(-2.1,-1.7)	21155.2	89.1	76.0	-1.9(-2.2,-1.6)	22816.6	112.5	105.6	-2.0(-2.2,-1.7)
	Other nutritional deficiencies	5325.4	12.1	12.9	-4.1(-4.6,-3.6)	2246.4	9.5	10.1	-4.3(-4.8,-3.8)	3078.9	15.2	15.7	-3.9(-4.4,-3.3)
Argentina	Overall nutritional deficiencies	101738.8	225.5	232.9	-3.0(-3.2,-2.8)	50752.8	219.5	223.2	-3.0(-3.1,-2.8)	50986.0	231.9	245.0	-3.1(-3.3,-2.9)
	Protein-energy malnutrition	26883.4	59.6	56.3	-5.4(-5.8,-5.0)	12120.6	52.4	45.7	-5.6(-5.9,-5.3)	14762.8	67.1	68.8	-5.2(-5.6,-4.8)
	Iodine deficiency	691.6	1.5	1.5	-0.6(-0.6,-0.6)	382.5	1.7	1.6	-0.5(-0.6,-0.5)	309.1	1.4	1.4	-0.6(-0.7,-0.6)
	Vitamin A deficiency	1982.5	4.4	5.5	-3.1(-3.2,-2.9)	822.8	3.6	4.7	-2.9(-3.2,-2.7)	1159.7	5.3	6.4	-3.2(-3.3,-3.0)
	Dietary iron deficiency	69220.4	153.4	163.5	-1.9(-2.1,-1.7)	36188.2	156.5	166.7	-1.9(-2.1,-1.7)	33032.2	150.2	161.0	-1.8(-2.1,-1.6)
	Other nutritional deficiencies	2961.0	6.6	6.0	-1.0(-1.3,-0.8)	1238.7	5.4	4.6	-1.1(-1.4,-0.8)	1722.3	7.8	7.6	-1.1(-1.3,-0.8)
Belize	Overall nutritional deficiencies	2155.6	525.6	552.3	-2.1(-2.2,-1.9)	1219.8	592.2	611.9	-1.7(-1.9,-1.6)	935.8	458.5	488.7	-2.5(-2.7,-2.3)
	Protein-energy malnutrition	524.6	127.9	149.3	-4.3(-4.6,-3.9)	179.6	87.2	101.4	-4.8(-5.1,-4.4)	345.0	169.0	196.1	-4.0(-4.3,-3.6)
	Iodine deficiency	9.0	2.2	2.2	-1.7(-1.8,-1.6)	5.1	2.5	2.5	-1.7(-1.8,-1.7)	3.9	1.9	1.9	-1.7(-1.7,-1.6)
	Vitamin A deficiency	34.8	8.5	8.3	-2.4(-2.6,-2.2)	17.3	8.4	8.3	-2.1(-2.2,-1.9)	17.4	8.5	8.3	-2.7(-2.9,-2.5)
	Dietary iron deficiency	1541.6	375.9	379.7	-0.6(-0.7,-0.5)	993.2	482.2	486.2	-0.5(-0.6,-0.4)	548.3	268.7	270.2	-0.9(-1.0,-0.8)

	Other nutritional deficiencies	45.7	11.1	12.9	-3.7(-4.0,-3.5)	24.5	11.9	13.6	-4.0(-4.3,-3.6)	21.1	10.4	12.1	-3.4(-3.8,-3.1)
Cuba	Overall nutritional deficiencies	28912.4	254.5	268.8	-1.4(-1.5,-1.4)	18495.8	324.1	328.3	-1.4(-1.5,-1.3)	10416.6	184.3	210.2	-1.5(-1.6,-1.4)
	Protein-energy malnutrition	4478.7	39.4	36.8	-1.3(-1.5,-1.1)	2108.8	37.0	35.3	-1.2(-1.4,-1.0)	2369.9	41.9	38.6	-1.4(-1.6,-1.2)
	Iodine deficiency	851.4	7.5	6.9	-2.2(-2.8,-1.6)	479.4	8.4	7.8	-2.1(-2.7,-1.5)	372.0	6.6	6.0	-2.2(-2.8,-1.6)
	Vitamin A deficiency	89.2	0.8	1.4	-2.7(-2.8,-2.6)	39.8	0.7	1.3	-2.4(-2.5,-2.3)	49.4	0.9	1.5	-2.9(-3.0,-2.7)
	Dietary iron deficiency	21932.3	193.1	211.2	-1.4(-1.5,-1.3)	15099.1	264.6	271.5	-1.4(-1.5,-1.3)	6833.2	120.9	151.6	-1.4(-1.5,-1.3)
	Other nutritional deficiencies	1560.8	13.7	12.4	-1.5(-2.0,-0.9)	768.7	13.5	12.3	-1.6(-2.2,-1.0)	792.1	14.0	12.5	-1.3(-1.9,-0.8)
Dominica	Overall nutritional deficiencies	284.2	413.8	430.8	-1.0(-1.3,-0.8)	176.6	525.0	533.8	-0.6(-0.9,-0.4)	107.6	307.0	335.2	-1.5(-1.8,-1.2)
	Protein-energy malnutrition	65.2	94.9	109.4	-1.9(-2.3,-1.5)	29.4	87.3	97.2	-1.8(-2.1,-1.4)	35.8	102.2	121.4	-2.1(-2.5,-1.7)
	Iodine deficiency	4.2	6.2	5.8	-2.7(-2.8,-2.5)	2.3	6.9	6.5	-2.6(-2.8,-2.5)	1.9	5.5	5.1	-2.7(-2.9,-2.5)
	Vitamin A deficiency	2.9	4.3	5.1	-1.3(-1.5,-1.1)	1.5	4.6	5.5	-1.2(-1.5,-1.0)	1.4	4.0	4.7	-1.5(-1.7,-1.2)
	Dietary iron deficiency	192.2	279.9	283.7	-0.5(-0.7,-0.3)	136.7	406.4	406.3	-0.2(-0.4,0.0)	55.5	158.5	168.2	-1.0(-1.1,-0.8)
	Other nutritional deficiencies	19.6	28.6	26.8	-1.4(-1.8,-1.0)	6.7	19.8	18.2	-1.3(-1.8,-0.9)	13.0	37.0	35.9	-1.7(-2.0,-1.3)
Dominican Republic	Overall nutritional deficiencies	47508.1	436.6	446.3	-3.7(-4.2,-3.1)	26134.5	483.0	491.5	-3.3(-3.8,-2.7)	21373.5	390.6	401.6	-4.1(-4.7,-3.5)
	Protein-energy malnutrition	19048.0	175.0	181.4	-5.6(-6.4,-4.8)	7992.2	147.7	152.8	-5.8(-6.8,-4.9)	11055.8	202.1	209.1	-5.5(-6.2,-4.8)
	Iodine deficiency	741.2	6.8	6.7	-2.7(-2.8,-2.6)	419.5	7.8	7.7	-2.6(-2.8,-2.5)	321.7	5.9	5.8	-2.8(-2.9,-2.7)
	Vitamin A deficiency	735.0	6.8	6.8	-3.2(-3.4,-3.1)	377.5	7.0	7.1	-2.8(-3.0,-2.7)	357.5	6.5	6.5	-3.6(-3.8,-3.4)
	Dietary iron deficiency	25338.3	232.8	235.7	-1.4(-1.6,-1.3)	16875.6	311.9	315.0	-1.1(-1.3,-1.0)	8462.8	154.7	157.6	-1.8(-2.0,-1.7)
	Other nutritional deficiencies	1645.6	15.1	15.8	-0.5(-1.4,0.5)	469.7	8.7	9.0	-0.5(-1.5,0.4)	1175.8	21.5	22.6	-0.5(-1.4,0.5)
Grenada	Overall nutritional deficiencies	405.2	392.6	433.2	-1.6(-1.8,-1.4)	235.4	467.3	494.3	-1.2(-1.4,-1.0)	169.7	321.3	380.5	-2.1(-2.3,-1.9)
	Protein-energy malnutrition	87.5	84.8	94.0	-3.3(-3.6,-3.0)	30.8	61.2	67.1	-3.0(-3.4,-2.7)	56.7	107.3	123.9	-3.5(-3.7,-3.3)
	Iodine deficiency	5.2	5.1	4.8	-3.5(-3.6,-3.3)	2.9	5.8	5.4	-3.4(-3.5,-3.3)	2.3	4.4	4.1	-3.5(-3.6,-3.4)
	Vitamin A deficiency	6.1	5.9	7.2	-2.6(-2.9,-2.2)	3.0	5.9	7.2	-2.2(-2.5,-1.8)	3.1	5.9	7.2	-2.9(-3.3,-2.5)
	Dietary iron deficiency	292.3	283.2	314.1	-0.8(-0.9,-0.7)	191.9	380.8	401.5	-0.6(-0.7,-0.5)	100.4	190.1	232.4	-1.0(-1.1,-0.8)
	Other nutritional deficiencies	14.0	13.6	13.1	-3.0(-3.5,-2.3)	6.9	13.6	13.0	-3.1(-3.8,-2.4)	7.1	13.5	13.0	-2.8(-3.3,-2.3)
Guyana	Overall nutritional deficiencies	6236.1	809.1	856.5	-2.5(-2.7,-2.4)	3506.5	900.6	938.1	-2.2(-2.4,-2.1)	2729.5	715.7	771.6	-2.9(-3.1,-2.7)
	Protein-energy malnutrition	2007.6	260.5	293.0	-3.9(-4.1,-3.7)	837.7	215.2	241.4	-4.1(-4.3,-3.8)	1169.9	306.8	348.3	-3.7(-4.0,-3.5)
	Iodine deficiency	76.1	9.9	9.6	-2.3(-2.4,-2.1)	43.3	11.1	10.8	-2.2(-2.4,-2.0)	32.8	8.6	8.4	-2.4(-2.5,-2.2)
	Vitamin A deficiency	69.2	9.0	9.3	-2.6(-2.8,-2.5)	33.2	8.5	8.9	-2.2(-2.3,-2.1)	36.0	9.5	9.6	-3.0(-3.1,-2.8)
	Dietary iron deficiency	3841.7	498.5	510.2	-1.2(-1.2,-1.1)	2488.9	639.2	648.2	-1.0(-1.0,-1.0)	1352.8	354.7	364.3	-1.5(-1.5,-1.4)
	Other nutritional deficiencies	241.5	31.3	34.5	-4.4(-5.1,-3.8)	103.5	26.6	28.7	-4.5(-5.2,-3.9)	138.0	36.2	40.9	-4.3(-5.0,-3.6)
Haiti	Overall nutritional deficiencies	165240.0	1332.4	1235.8	-2.7(-3.0,-2.4)	87947.8	1376.9	1332.4	-2.6(-2.9,-2.3)	77292.2	1285.1	1120.2	-2.8(-3.1,-2.4)
	Protein-energy malnutrition	69770.4	562.6	491.2	-4.6(-5.0,-4.2)	30373.7	475.5	434.3	-5.0(-5.4,-4.6)	39396.7	655.0	546.2	-4.2(-4.7,-3.8)

	Iodine deficiency	2796.1	22.5	23.0	-1.0(-1.2,-0.8)	1607.7	25.2	25.4	-1.0(-1.2,-0.8)	1188.4	19.8	20.5	-1.0(-1.2,-0.8)
	Vitamin A deficiency	3869.3	31.2	26.1	-1.4(-1.5,-1.4)	1832.5	28.7	24.8	-1.1(-1.2,-1.1)	2036.8	33.9	27.4	-1.7(-1.7,-1.6)
	Dietary iron deficiency	86510.5	697.5	674.8	-0.1(-0.2,-0.1)	52795.9	826.5	824.8	-0.2(-0.3,-0.2)	33714.5	560.6	508.1	0.0(-0.1,0.0)
	Other nutritional deficiencies	2293.7	18.5	20.6	-3.3(-3.9,-2.8)	1338.0	20.9	23.1	-2.9(-3.3,-2.4)	955.7	15.9	17.9	-3.9(-4.5,-3.3)
Jamaica	Overall nutritional deficiencies	10785.4	383.7	409.4	-2.4(-2.8,-2.0)	6778.3	479.0	493.2	-1.8(-2.2,-1.4)	4007.1	287.1	323.9	-3.1(-3.6,-2.7)
	Protein-energy malnutrition	1937.6	68.9	74.9	-6.0(-6.7,-5.3)	825.0	58.3	62.4	-6.1(-6.8,-5.4)	1112.6	79.7	87.8	-6.0(-6.7,-5.2)
	Iodine deficiency	207.5	7.4	6.9	-1.9(-2.0,-1.7)	118.1	8.3	7.8	-1.8(-2.0,-1.7)	89.4	6.4	6.0	-1.9(-2.0,-1.7)
	Vitamin A deficiency	137.4	4.9	5.8	-1.8(-2.0,-1.6)	71.8	5.1	6.1	-1.6(-1.7,-1.4)	65.6	4.7	5.5	-2.1(-2.3,-1.9)
	Dietary iron deficiency	8156.0	290.2	310.0	-0.6(-0.8,-0.4)	5606.7	396.2	406.5	-0.4(-0.6,-0.2)	2549.3	182.7	211.3	-1.0(-1.2,-0.9)
	Other nutritional deficiencies	346.8	12.3	11.8	-2.8(-3.3,-2.3)	156.7	11.1	10.4	-2.4(-2.7,-2.1)	190.1	13.6	13.3	-3.0(-3.7,-2.3)
Saint Lucia	Overall nutritional deficiencies	753.6	431.5	460.5	-1.9(-2.1,-1.7)	482.2	549.3	559.9	-1.5(-1.6,-1.3)	271.4	312.5	362.5	-2.6(-2.9,-2.3)
	Protein-energy malnutrition	164.9	94.4	102.5	-3.8(-4.2,-3.5)	70.2	80.0	86.7	-3.5(-3.8,-3.2)	94.6	109.0	120.3	-4.1(-4.6,-3.7)
	Iodine deficiency	5.3	3.1	2.8	-1.5(-1.6,-1.5)	3.0	3.5	3.1	-1.5(-1.6,-1.5)	2.3	2.7	2.4	-1.5(-1.5,-1.4)
	Vitamin A deficiency	8.3	4.8	6.3	-1.7(-1.9,-1.5)	4.2	4.8	6.6	-1.5(-1.7,-1.3)	4.1	4.7	6.1	-1.9(-2.2,-1.7)
	Dietary iron deficiency	542.2	310.5	331.5	-1.0(-1.1,-0.9)	383.6	437.0	441.7	-0.7(-0.8,-0.6)	158.6	182.6	221.2	-1.4(-1.5,-1.3)
	Other nutritional deficiencies	32.9	18.8	17.3	-3.9(-4.7,-3.1)	21.1	24.0	21.8	-4.0(-4.8,-3.2)	11.8	13.6	12.5	-3.5(-4.3,-2.8)
Saint Vincent and the Grenadines	Overall nutritional deficiencies	651.4	575.8	604.7	-1.9(-2.1,-1.7)	362.0	654.7	669.6	-1.6(-1.7,-1.4)	289.5	500.3	544.6	-2.3(-2.5,-2.1)
	Protein-energy malnutrition	209.0	184.7	200.2	-3.5(-3.9,-3.2)	80.7	146.0	160.0	-3.7(-4.1,-3.3)	128.3	221.8	239.7	-3.5(-3.9,-3.1)
	Iodine deficiency	6.6	5.8	5.5	-3.1(-3.3,-3.0)	3.6	6.5	6.2	-3.1(-3.2,-2.9)	2.9	5.1	4.8	-3.2(-3.3,-3.0)
	Vitamin A deficiency	7.1	6.2	7.6	-2.4(-2.7,-2.2)	3.5	6.4	7.7	-2.0(-2.2,-1.8)	3.5	6.1	7.4	-2.8(-3.1,-2.5)
	Dietary iron deficiency	399.6	353.2	367.5	-0.5(-0.6,-0.4)	265.3	479.8	480.6	-0.4(-0.5,-0.3)	134.3	232.2	259.8	-0.7(-0.8,-0.6)
	Other nutritional deficiencies	29.2	25.8	24.0	-2.3(-2.6,-2.0)	8.8	16.0	15.0	-2.1(-2.5,-1.7)	20.4	35.2	33.0	-2.6(-2.9,-2.3)
Suriname	Overall nutritional deficiencies	2791.6	484.8	502.8	-2.0(-2.1,-2.0)	1684.8	578.8	588.8	-1.6(-1.7,-1.6)	1106.9	388.6	415.1	-2.5(-2.6,-2.4)
	Protein-energy malnutrition	575.3	99.9	112.5	-4.5(-4.7,-4.3)	262.5	90.2	101.3	-4.2(-4.5,-4.0)	312.8	109.8	124.7	-4.7(-4.9,-4.5)
	Iodine deficiency	28.9	5.0	4.9	-3.0(-3.4,-2.6)	16.5	5.7	5.5	-3.0(-3.4,-2.6)	12.4	4.4	4.2	-3.0(-3.4,-2.6)
	Vitamin A deficiency	49.5	8.6	9.3	-2.1(-2.1,-2.0)	24.8	8.5	9.5	-1.7(-1.7,-1.6)	24.7	8.7	9.2	-2.4(-2.5,-2.3)
	Dietary iron deficiency	2081.8	361.5	366.3	-0.7(-0.8,-0.7)	1349.9	463.8	461.9	-0.7(-0.7,-0.6)	731.9	257.0	267.9	-0.9(-0.9,-0.8)
	Other nutritional deficiencies	56.2	9.8	9.8	-3.1(-3.7,-2.5)	31.0	10.6	10.6	-3.2(-3.8,-2.5)	25.2	8.8	9.0	-3.0(-3.5,-2.4)
Bolivia (Plurinational State of)	Overall nutritional deficiencies	89860.6	748.1	729.9	-4.0(-4.2,-3.9)	40841.7	680.8	674.8	-4.3(-4.4,-4.2)	49018.9	815.3	781.8	-3.8(-4.0,-3.7)
	Protein-energy malnutrition	32959.0	274.4	279.2	-6.2(-6.3,-6.0)	16138.8	269.0	275.2	-6.2(-6.4,-6.1)	16820.3	279.8	282.1	-6.1(-6.3,-6.0)
	Iodine deficiency	227.0	1.9	2.0	-2.3(-2.4,-2.2)	125.0	2.1	2.2	-2.3(-2.4,-2.2)	102.0	1.7	1.8	-2.2(-2.3,-2.1)
	Vitamin A deficiency	1719.1	14.3	12.5	-0.9(-1.1,-0.8)	818.7	13.6	12.0	-1.0(-1.2,-0.9)	900.4	15.0	13.0	-0.9(-1.1,-0.7)
	Dietary iron deficiency	51876.9	431.9	406.6	-1.4(-1.5,-1.4)	22229.6	370.5	356.7	-1.8(-1.9,-1.7)	29647.3	493.1	454.4	-1.2(-1.3,-1.1)

	Other nutritional deficiencies	3078.5	25.6	29.5	-3.6(-3.8,-3.4)	1529.6	25.5	28.7	-3.7(-3.8,-3.6)	1548.9	25.8	30.5	-3.5(-3.7,-3.2)
Ecuador	Overall nutritional deficiencies	38343.6	218.0	238.1	-5.2(-5.5,-4.9)	21241.2	240.5	258.2	-5.1(-5.3,-4.9)	17102.4	195.3	216.8	-5.3(-5.7,-4.8)
	Protein-energy malnutrition	17455.5	99.2	113.3	-6.3(-6.8,-5.7)	8119.6	91.9	104.7	-6.5(-7.0,-6.1)	9335.9	106.6	121.6	-6.0(-6.7,-5.4)
	Iodine deficiency	142.5	0.8	0.8	-0.5(-0.6,-0.4)	78.2	0.9	0.9	-0.5(-0.6,-0.4)	64.2	0.7	0.7	-0.5(-0.6,-0.5)
	Vitamin A deficiency	937.0	5.3	5.3	-3.6(-3.8,-3.4)	489.5	5.5	5.6	-3.2(-3.4,-3.0)	447.5	5.1	5.0	-4.0(-4.2,-3.8)
	Dietary iron deficiency	16435.1	93.4	97.0	-4.2(-4.3,-4.0)	10867.5	123.0	126.0	-4.0(-4.1,-3.8)	5567.6	63.6	67.1	-4.5(-4.7,-4.4)
	Other nutritional deficiencies	3373.6	19.2	21.7	-2.0(-2.4,-1.7)	1686.4	19.1	21.1	-2.3(-2.7,-2.0)	1687.2	19.3	22.4	-1.8(-2.2,-1.4)
Peru	Overall nutritional deficiencies	112730.7	331.6	342.7	-5.4(-5.6,-5.2)	58296.3	343.7	349.0	-5.9(-6.1,-5.6)	54434.4	319.6	332.6	-4.9(-5.1,-4.8)
	Protein-energy malnutrition	33562.0	98.7	103.4	-7.8(-8.2,-7.5)	16830.5	99.2	102.4	-8.0(-8.4,-7.7)	16731.6	98.2	104.1	-7.7(-8.0,-7.3)
	Iodine deficiency	275.6	0.8	0.8	-2.8(-3.7,-1.9)	150.9	0.9	0.9	-2.8(-3.7,-1.9)	124.7	0.7	0.7	-2.8(-3.7,-1.9)
	Vitamin A deficiency	3174.5	9.3	9.7	-3.2(-3.4,-3.0)	1420.4	8.4	8.9	-3.0(-3.2,-2.8)	1754.1	10.3	10.5	-3.4(-3.6,-3.1)
	Dietary iron deficiency	71368.6	209.9	215.7	-3.5(-3.6,-3.3)	37558.0	221.4	223.0	-4.4(-4.5,-4.2)	33810.6	198.5	204.9	-2.2(-2.3,-2.2)
	Other nutritional deficiencies	4349.9	12.8	13.1	-5.0(-5.2,-4.8)	2336.5	13.8	13.8	-4.9(-5.2,-4.6)	2013.4	11.8	12.3	-5.2(-5.3,-5.0)
Colombia	Overall nutritional deficiencies	107646.1	225.3	242.1	-3.1(-3.3,-2.8)	56439.7	231.1	246.9	-3.1(-3.3,-2.8)	51206.4	219.3	236.8	-3.1(-3.3,-2.8)
	Protein-energy malnutrition	56771.2	118.8	133.0	-2.8(-3.2,-2.4)	27908.6	114.3	131.5	-2.6(-3.0,-2.2)	28862.6	123.6	135.7	-3.0(-3.4,-2.5)
	Iodine deficiency	4581.9	9.6	9.3	-1.2(-2.2,-0.3)	2776.4	11.4	11.0	-1.2(-2.1,-0.2)	1805.4	7.7	7.5	-1.3(-2.3,-0.3)
	Vitamin A deficiency	1882.4	3.9	4.3	-2.8(-3.0,-2.6)	1019.3	4.2	4.7	-2.2(-2.4,-2.0)	863.2	3.7	4.0	-3.3(-3.5,-3.1)
	Dietary iron deficiency	41846.0	87.6	90.2	-3.4(-3.6,-3.3)	23306.6	95.4	94.0	-3.7(-3.9,-3.5)	18539.4	79.4	84.8	-3.1(-3.2,-3.0)
	Other nutritional deficiencies	2564.6	5.4	5.3	-4.9(-5.4,-4.5)	1428.8	5.8	5.7	-4.8(-5.3,-4.2)	1135.8	4.9	4.9	-5.1(-5.6,-4.7)
Costa Rica	Overall nutritional deficiencies	7494.3	158.9	165.2	-2.0(-2.1,-1.9)	4150.9	170.7	174.9	-2.0(-2.1,-1.9)	3343.4	146.3	154.5	-2.0(-2.2,-1.8)
	Protein-energy malnutrition	1573.3	33.4	33.9	-2.5(-3.0,-2.0)	578.5	23.8	24.7	-2.6(-3.1,-2.1)	994.8	43.5	44.1	-2.4(-2.9,-1.9)
	Iodine deficiency	240.5	5.1	4.9	-3.4(-4.0,-2.8)	149.3	6.1	5.8	-3.4(-3.9,-2.8)	91.3	4.0	3.8	-3.6(-4.2,-3.0)
	Vitamin A deficiency	170.5	3.6	4.1	-2.0(-2.2,-1.8)	92.1	3.8	4.4	-1.8(-2.0,-1.6)	78.5	3.4	3.8	-2.2(-2.4,-2.0)
	Dietary iron deficiency	5006.7	106.1	112.1	-1.8(-1.9,-1.7)	3159.2	129.9	133.2	-1.9(-1.9,-1.8)	1847.5	80.9	89.0	-1.8(-1.8,-1.7)
	Other nutritional deficiencies	503.3	10.7	10.1	-1.5(-2.2,-0.9)	171.9	7.1	6.7	-2.3(-2.9,-1.7)	331.4	14.5	13.9	-1.0(-1.7,-0.4)
El Salvador	Overall nutritional deficiencies	18162.9	290.3	299.6	-2.8(-2.9,-2.6)	8996.1	270.6	280.0	-2.9(-3.0,-2.7)	9166.7	312.7	324.0	-2.7(-2.8,-2.6)
	Protein-energy malnutrition	7508.7	120.0	124.0	-4.1(-4.3,-3.9)	3407.7	102.5	103.0	-4.5(-4.7,-4.3)	4101.0	139.9	151.7	-3.6(-3.9,-3.4)
	Iodine deficiency	681.3	10.9	10.7	-1.8(-2.9,-0.8)	429.1	12.9	12.5	-1.8(-2.8,-0.7)	252.2	8.6	8.6	-2.0(-3.2,-0.8)
	Vitamin A deficiency	471.6	7.5	7.8	-3.0(-3.3,-2.7)	236.7	7.1	7.7	-2.6(-2.9,-2.3)	234.9	8.0	7.8	-3.4(-3.7,-3.0)
	Dietary iron deficiency	9131.8	146.0	151.2	-1.4(-1.6,-1.2)	4715.5	141.8	150.6	-1.3(-1.5,-1.1)	4416.3	150.7	150.0	-1.5(-1.7,-1.3)
	Other nutritional deficiencies	369.4	5.9	5.9	-0.6(-1.3,0.1)	207.1	6.2	6.1	-0.5(-1.3,0.2)	162.3	5.5	5.8	-0.7(-1.4,0.1)
Guatemala	Overall nutritional deficiencies	106587.9	599.6	683.6	-6.4(-6.6,-6.2)	57188.9	626.0	701.1	-6.5(-6.7,-6.2)	49399.0	571.7	674.2	-6.3(-6.5,-6.1)
	Protein-energy malnutrition	55348.0	311.4	391.5	-7.7(-8.0,-7.4)	27795.9	304.3	372.0	-8.0(-8.3,-7.7)	27552.1	318.8	421.9	-7.3(-7.7,-7.0)

	Iodine deficiency	1900.7	10.7	10.7	-1.1(-2.3,0.1)	1162.3	12.7	12.5	-1.0(-2.2,0.1)	738.4	8.5	8.7	-1.2(-2.4,0.0)
	Vitamin A deficiency	1822.7	10.3	9.3	-2.4(-2.7,-2.1)	998.5	10.9	10.1	-2.1(-2.3,-1.8)	824.2	9.5	8.4	-2.7(-3.0,-2.3)
	Dietary iron deficiency	43368.7	244.0	243.0	-1.3(-1.6,-1.1)	24855.6	272.1	275.3	-1.3(-1.6,-1.0)	18513.0	214.2	207.7	-1.4(-1.7,-1.2)
	Other nutritional deficiencies	4147.8	23.3	29.2	-8.1(-9.1,-7.2)	2376.5	26.0	31.2	-8.3(-9.3,-7.3)	1771.3	20.5	27.6	-7.9(-8.7,-7.1)
Honduras	Overall nutritional deficiencies	32741.9	333.6	356.8	-2.9(-3.0,-2.7)	18488.1	367.1	398.3	-2.7(-2.9,-2.6)	14253.8	298.3	310.9	-3.1(-3.3,-2.9)
	Protein-energy malnutrition	8097.3	82.5	100.2	-5.0(-5.3,-4.7)	4502.5	89.4	109.3	-5.0(-5.3,-4.8)	3594.8	75.2	89.5	-5.0(-5.3,-4.7)
	Iodine deficiency	1350.8	13.8	13.7	-1.4(-2.5,-0.3)	819.4	16.3	16.0	-1.3(-2.3,-0.3)	531.4	11.1	11.3	-1.5(-2.7,-0.3)
	Vitamin A deficiency	773.1	7.9	7.0	-2.7(-2.8,-2.5)	404.0	8.0	7.3	-2.2(-2.4,-2.1)	369.1	7.7	6.7	-3.0(-3.3,-2.8)
	Dietary iron deficiency	21568.7	219.8	223.5	-1.3(-1.4,-1.2)	12137.4	241.0	250.2	-1.0(-1.1,-0.9)	9431.3	197.4	194.3	-1.7(-1.7,-1.6)
	Other nutritional deficiencies	952.0	9.7	12.4	-4.2(-4.5,-3.8)	624.9	12.4	15.4	-4.1(-4.4,-3.8)	327.1	6.8	9.0	-4.3(-4.8,-3.8)
Mexico	Overall nutritional deficiencies	403500.5	323.0	345.9	-4.0(-4.3,-3.7)	218933.3	343.0	361.4	-3.9(-4.2,-3.6)	184567.2	302.0	329.7	-4.2(-4.5,-3.8)
	Protein-energy malnutrition	205018.8	164.1	180.3	-5.0(-5.3,-4.7)	96985.9	151.9	166.5	-5.1(-5.4,-4.9)	108032.9	176.8	195.9	-4.8(-5.1,-4.5)
	Iodine deficiency	12941.4	10.4	10.0	-0.6(-0.9,-0.2)	9744.5	15.3	14.7	-0.4(-0.7,-0.1)	3196.9	5.2	5.1	-1.0(-1.6,-0.5)
	Vitamin A deficiency	11070.4	8.9	9.5	-2.2(-2.4,-2.1)	5601.5	8.8	9.5	-1.8(-1.9,-1.7)	5468.9	8.9	9.4	-2.6(-2.8,-2.5)
	Dietary iron deficiency	140594.2	112.5	118.1	-1.3(-1.5,-1.1)	89282.7	139.9	143.1	-1.1(-1.3,-0.9)	51311.5	84.0	90.6	-1.6(-1.8,-1.4)
	Other nutritional deficiencies	33875.7	27.1	28.0	-6.0(-6.6,-5.4)	17318.7	27.1	27.5	-6.1(-6.7,-5.5)	16557.0	27.1	28.7	-5.9(-6.6,-5.2)
Nicaragua	Overall nutritional deficiencies	15706.4	241.3	256.7	-4.9(-5.1,-4.7)	7561.6	229.3	243.5	-5.2(-5.5,-4.9)	8144.9	253.5	270.8	-4.6(-4.8,-4.4)
	Protein-energy malnutrition	8629.5	132.5	141.6	-5.8(-6.1,-5.5)	3798.1	115.2	123.1	-6.2(-6.6,-5.9)	4831.4	150.4	161.8	-5.4(-5.7,-5.1)
	Iodine deficiency	888.5	13.6	13.4	-1.3(-2.4,-0.3)	529.9	16.1	15.7	-1.3(-2.3,-0.3)	358.5	11.2	11.0	-1.4(-2.6,-0.3)
	Vitamin A deficiency	254.6	3.9	3.8	-3.3(-3.7,-3.0)	145.8	4.4	4.3	-2.5(-2.8,-2.2)	108.8	3.4	3.3	-4.1(-4.5,-3.6)
	Dietary iron deficiency	5634.1	86.5	93.1	-3.5(-3.6,-3.3)	2946.7	89.4	95.9	-3.8(-4.0,-3.6)	2687.4	83.6	89.4	-3.0(-3.1,-3.0)
	Other nutritional deficiencies	299.9	4.6	4.9	-1.4(-2.0,-0.9)	141.0	4.3	4.5	-0.9(-1.4,-0.4)	158.8	4.9	5.2	-1.9(-2.5,-1.2)
Venezuela (Bolivarian Republic of)	Overall nutritional deficiencies	61827.9	220.3	234.7	-3.4(-4.0,-2.9)	31735.7	222.0	236.6	-3.4(-3.9,-2.9)	30092.2	218.5	233.3	-3.5(-4.1,-2.9)
	Protein-energy malnutrition	26412.8	94.1	105.5	-4.5(-5.5,-3.6)	11548.2	80.8	93.9	-4.6(-5.5,-3.6)	14864.6	107.9	118.3	-4.4(-5.4,-3.5)
	Iodine deficiency	2751.0	9.8	9.5	-1.6(-2.6,-0.5)	1660.8	11.6	11.3	-1.5(-2.5,-0.5)	1090.2	7.9	7.7	-1.7(-2.8,-0.6)
	Vitamin A deficiency	1393.3	5.0	5.4	-1.3(-1.6,-1.0)	720.6	5.0	5.6	-1.0(-1.2,-0.7)	672.7	4.9	5.2	-1.6(-2.0,-1.3)
	Dietary iron deficiency	29798.2	106.2	108.9	-2.1(-2.3,-1.9)	17179.0	120.2	121.5	-2.2(-2.4,-1.9)	12619.2	91.6	95.8	-2.0(-2.2,-1.9)
	Other nutritional deficiencies	1472.6	5.2	5.2	-6.2(-7.0,-5.4)	627.0	4.4	4.4	-6.6(-7.4,-5.7)	845.6	6.1	6.2	-5.9(-6.7,-5.1)
Brazil	Overall nutritional deficiencies	942659.1	435.1	455.7	-3.8(-3.9,-3.6)	629795.9	568.1	576.0	-3.1(-3.2,-3.0)	312863.2	295.7	332.0	-4.7(-4.9,-4.5)
	Protein-energy malnutrition	234582.1	108.3	121.3	-6.7(-7.0,-6.4)	100678.2	90.8	101.7	-6.8(-7.1,-6.5)	133903.8	126.6	143.6	-6.6(-6.9,-6.3)
	Iodine deficiency	2583.8	1.2	1.1	-0.8(-1.0,-0.7)	1502.4	1.4	1.3	-0.7(-0.9,-0.6)	1081.4	1.0	1.0	-1.0(-1.1,-0.9)
	Vitamin A deficiency	18072.3	8.3	10.2	-2.8(-2.9,-2.7)	8978.2	8.1	10.2	-2.6(-2.7,-2.5)	9094.0	8.6	10.2	-2.9(-3.0,-2.8)
	Dietary iron deficiency	659743.0	304.5	310.3	-1.4(-1.5,-1.4)	505940.6	456.4	451.6	-1.3(-1.4,-1.2)	153802.5	145.4	162.8	-1.8(-1.9,-1.7)

	Other nutritional deficiencies	27677.9	12.8	12.7	-4.9(-5.2,-4.6)	12696.4	11.5	11.3	-4.9(-5.2,-4.6)	14981.5	14.2	14.4	-4.8(-5.1,-4.5)
Paraguay	Overall nutritional deficiencies	29248.8	422.0	448.9	-0.4(-0.7,-0.1)	17654.3	515.7	535.6	-0.5(-0.8,-0.2)	11594.5	330.6	366.6	-0.2(-0.5,0.2)
	Protein-energy malnutrition	10610.4	153.1	176.0	1.2(0.5,1.9)	4972.9	145.3	163.3	0.9(0.2,1.5)	5637.5	160.7	190.6	1.6(0.8,2.3)
	Iodine deficiency	85.8	1.2	1.2	-4.6(-5.4,-3.8)	47.9	1.4	1.4	-4.6(-5.4,-3.8)	37.9	1.1	1.1	-4.6(-5.4,-3.8)
	Vitamin A deficiency	625.6	9.0	9.0	-2.0(-2.1,-1.9)	322.0	9.4	9.5	-1.9(-2.0,-1.8)	303.6	8.7	8.6	-2.2(-2.3,-2.1)
	Dietary iron deficiency	17432.9	251.5	255.0	-1.2(-1.3,-1.1)	12045.5	351.9	353.3	-1.0(-1.1,-0.8)	5387.3	153.6	159.1	-1.6(-1.7,-1.5)
	Other nutritional deficiencies	494.1	7.1	7.7	0.8(0.3,1.3)	266.0	7.8	8.2	0.5(0.2,0.9)	228.2	6.5	7.2	1.1(0.4,1.7)
Algeria	Overall nutritional deficiencies	111700.0	266.9	268.7	-2.5(-2.6,-2.5)	68728.4	332.9	338.1	-2.3(-2.3,-2.3)	42971.6	202.7	201.3	-2.8(-2.9,-2.7)
	Protein-energy malnutrition	21190.3	50.6	51.4	-4.3(-4.5,-4.0)	11559.2	56.0	57.8	-3.8(-4.0,-3.6)	9631.1	45.4	45.6	-4.8(-5.1,-4.4)
	Iodine deficiency	5230.0	12.5	12.5	-2.5(-2.7,-2.2)	3201.0	15.5	15.4	-2.1(-2.3,-1.8)	2029.0	9.6	9.6	-3.0(-3.4,-2.7)
	Vitamin A deficiency	3077.8	7.4	7.3	-2.8(-3.0,-2.7)	1455.7	7.1	7.1	-2.3(-2.4,-2.2)	1622.0	7.7	7.6	-3.3(-3.4,-3.1)
	Dietary iron deficiency	77744.7	185.8	186.5	-1.9(-2.0,-1.9)	50009.0	242.2	245.4	-1.9(-2.0,-1.9)	27735.6	130.8	129.1	-1.9(-2.0,-1.9)
	Other nutritional deficiencies	4457.3	10.7	10.9	-2.0(-2.3,-1.7)	2503.5	12.1	12.4	-1.1(-1.5,-0.6)	1953.8	9.2	9.4	-3.0(-3.2,-2.7)
Egypt	Overall nutritional deficiencies	306185.5	309.1	309.2	-2.3(-2.3,-2.2)	158383.5	332.0	349.8	-2.4(-2.4,-2.3)	147802.0	287.8	276.3	-2.0(-2.1,-1.9)
	Protein-energy malnutrition	54320.2	54.8	63.2	-3.3(-3.5,-3.1)	24170.0	50.7	63.1	-3.6(-3.8,-3.4)	30150.2	58.7	64.9	-2.8(-3.1,-2.6)
	Iodine deficiency	15441.4	15.6	16.1	-4.3(-4.6,-4.0)	11399.2	23.9	24.5	-4.0(-4.3,-3.7)	4042.1	7.9	8.3	-4.9(-5.2,-4.6)
	Vitamin A deficiency	4196.0	4.2	3.8	-2.8(-3.1,-2.5)	2147.1	4.5	4.0	-2.4(-2.7,-2.1)	2048.9	4.0	3.6	-3.2(-3.5,-2.9)
	Dietary iron deficiency	219598.3	221.7	210.5	-1.8(-1.9,-1.7)	115567.2	242.2	244.0	-1.9(-2.0,-1.8)	104031.1	202.5	182.3	-1.6(-1.7,-1.5)
	Other nutritional deficiencies	12629.7	12.7	15.6	-0.7(-1.1,-0.4)	5099.9	10.7	14.1	-0.6(-0.9,-0.3)	7529.8	14.7	17.2	-0.8(-1.1,-0.4)
Iran (Islamic Republic of)	Overall nutritional deficiencies	123513.9	146.5	156.7	-3.4(-3.6,-3.1)	66278.7	159.8	171.1	-3.4(-3.6,-3.2)	57235.2	133.7	142.6	-3.3(-3.5,-3.1)
	Protein-energy malnutrition	27885.7	33.1	35.7	-5.2(-5.5,-4.9)	13303.7	32.1	34.9	-5.5(-5.9,-5.2)	14581.9	34.1	36.6	-4.9(-5.2,-4.6)
	Iodine deficiency	5158.0	6.1	5.9	-3.7(-4.0,-3.4)	3221.2	7.8	7.5	-3.6(-3.8,-3.3)	1936.8	4.5	4.4	-3.8(-4.2,-3.4)
	Vitamin A deficiency	540.2	0.6	0.8	-8.7(-9.2,-8.3)	234.6	0.6	0.7	-8.1(-8.5,-7.7)	305.6	0.7	0.8	-9.2(-9.7,-8.7)
	Dietary iron deficiency	81576.3	96.8	104.3	-2.6(-2.8,-2.5)	45528.2	109.8	118.2	-2.7(-2.9,-2.5)	36048.1	84.2	90.5	-2.5(-2.7,-2.4)
	Other nutritional deficiencies	8353.8	9.9	10.0	-0.7(-1.2,-0.1)	3991.0	9.6	9.8	-0.3(-0.8,0.2)	4362.8	10.2	10.3	-1.0(-1.6,-0.3)
Iraq	Overall nutritional deficiencies	113150.6	268.6	265.4	-2.7(-2.9,-2.6)	66247.1	322.4	326.8	-2.4(-2.6,-2.3)	46903.5	217.5	205.7	-3.1(-3.4,-2.9)
	Protein-energy malnutrition	23197.9	55.1	52.0	-4.7(-5.0,-4.5)	11150.5	54.3	51.2	-4.7(-5.0,-4.5)	12047.4	55.9	52.9	-4.7(-5.0,-4.4)
	Iodine deficiency	9856.3	23.4	23.7	-2.2(-2.9,-1.6)	5847.5	28.5	28.5	-1.9(-2.4,-1.3)	4008.8	18.6	19.0	-2.7(-3.4,-1.9)
	Vitamin A deficiency	3353.4	8.0	7.3	-3.3(-3.5,-3.2)	1540.9	7.5	6.9	-2.9(-3.1,-2.8)	1812.6	8.4	7.7	-3.6(-3.8,-3.4)
	Dietary iron deficiency	72632.5	172.4	172.9	-1.9(-2.0,-1.8)	45891.9	223.3	231.6	-1.7(-1.9,-1.6)	26740.6	124.0	115.6	-2.2(-2.4,-2.1)
	Other nutritional deficiencies	4110.4	9.8	9.5	-2.9(-3.2,-2.6)	1816.4	8.8	8.6	-2.0(-2.3,-1.7)	2294.0	10.6	10.4	-3.5(-3.9,-3.2)
Jordan	Overall nutritional deficiencies	27907.2	239.8	238.9	-2.1(-2.2,-2.0)	18588.4	342.8	347.0	-1.9(-2.0,-1.8)	9318.8	149.9	148.4	-2.3(-2.4,-2.3)
	Protein-energy malnutrition	2562.5	22.0	24.2	-2.1(-2.3,-1.9)	1296.5	23.9	26.2	-2.5(-2.8,-2.2)	1266.0	20.4	22.5	-1.4(-1.6,-1.3)

	Iodine deficiency	1353.5	11.6	11.8	-1.8(-1.9,-1.6)	734.4	13.5	13.8	-1.5(-1.6,-1.3)	619.1	10.0	10.1	-2.1(-2.3,-1.9)
	Vitamin A deficiency	881.9	7.6	7.1	-2.9(-3.2,-2.6)	404.2	7.5	6.8	-2.7(-2.9,-2.5)	477.7	7.7	7.4	-3.1(-3.4,-2.8)
	Dietary iron deficiency	22543.2	193.7	190.7	-2.1(-2.2,-2.0)	15879.4	292.9	294.8	-1.9(-2.0,-1.7)	6663.8	107.2	103.6	-2.5(-2.5,-2.4)
	Other nutritional deficiencies	566.2	4.9	5.0	-2.0(-2.3,-1.7)	273.9	5.1	5.3	-2.2(-2.5,-1.9)	292.2	4.7	4.8	-1.8(-2.0,-1.5)
Lebanon	Overall nutritional deficiencies	7463.0	144.2	145.4	-3.1(-3.2,-2.9)	4288.9	163.1	163.7	-3.4(-3.6,-3.2)	3174.1	124.6	124.1	-2.6(-2.7,-2.5)
	Protein-energy malnutrition	1994.5	38.5	38.6	-0.9(-0.9,-0.8)	1121.0	42.6	42.5	-1.0(-1.1,-0.9)	873.5	34.3	34.2	-0.7(-0.7,-0.6)
	Iodine deficiency	730.1	14.1	14.0	-2.5(-3.0,-2.1)	465.7	17.7	17.5	-2.4(-2.8,-1.9)	264.4	10.4	10.4	-2.8(-3.3,-2.3)
	Vitamin A deficiency	52.2	1.0	1.0	-6.1(-6.4,-5.9)	26.1	1.0	1.1	-5.7(-6.0,-5.4)	26.2	1.0	1.0	-6.5(-6.8,-6.2)
	Dietary iron deficiency	4142.1	80.0	81.3	-4.0(-4.1,-3.8)	2358.6	89.7	90.9	-4.4(-4.7,-4.2)	1783.5	70.0	69.5	-3.3(-3.5,-3.2)
	Other nutritional deficiencies	544.1	10.5	10.4	0.0(-0.6,0.6)	317.5	12.1	11.7	-0.1(-0.7,0.5)	226.6	8.9	8.9	0.0(-0.5,0.6)
Libya	Overall nutritional deficiencies	16845.5	250.1	270.9	-1.2(-1.4,-1.0)	11076.2	340.1	352.0	-1.0(-1.2,-0.9)	5769.3	165.8	194.8	-1.5(-1.6,-1.3)
	Protein-energy malnutrition	2017.9	30.0	35.2	-1.3(-1.5,-1.2)	934.4	28.7	33.6	-1.7(-1.9,-1.5)	1083.5	31.1	36.7	-1.0(-1.1,-0.8)
	Iodine deficiency	1669.7	24.8	23.3	0.1(-0.3,0.5)	979.1	30.1	28.1	0.3(-0.1,0.7)	690.6	19.9	18.7	-0.1(-0.6,0.3)
	Vitamin A deficiency	418.1	6.2	7.0	-1.9(-2.2,-1.6)	194.7	6.0	6.8	-1.7(-2.0,-1.5)	223.4	6.4	7.3	-2.1(-2.4,-1.8)
	Dietary iron deficiency	12130.7	180.1	196.3	-1.3(-1.4,-1.1)	8688.4	266.8	274.9	-1.0(-1.2,-0.9)	3442.2	98.9	122.6	-1.8(-2.0,-1.6)
	Other nutritional deficiencies	609.0	9.0	9.1	-0.3(-0.8,0.2)	279.6	8.6	8.6	-0.5(-1.0,0.0)	329.5	9.5	9.5	-0.2(-0.7,0.4)
Morocco	Overall nutritional deficiencies	124134.0	345.3	359.0	-2.0(-2.1,-2.0)	78710.8	440.4	452.6	-1.8(-1.9,-1.8)	45423.2	251.3	264.9	-2.3(-2.3,-2.2)
	Protein-energy malnutrition	16867.9	46.9	52.0	-3.3(-3.4,-3.2)	8584.8	48.0	53.6	-3.5(-3.7,-3.4)	8283.0	45.8	50.5	-3.1(-3.2,-2.9)
	Iodine deficiency	11355.2	31.6	30.8	-3.0(-3.3,-2.8)	6761.2	37.8	36.7	-2.8(-2.9,-2.6)	4594.0	25.4	24.9	-3.4(-3.7,-3.1)
	Vitamin A deficiency	3352.9	9.3	10.0	-3.5(-3.6,-3.3)	1529.2	8.6	9.3	-3.1(-3.2,-2.9)	1823.7	10.1	10.7	-3.8(-4.0,-3.7)
	Dietary iron deficiency	88183.1	245.3	253.6	-1.5(-1.6,-1.5)	59706.6	334.0	340.8	-1.4(-1.4,-1.3)	28476.5	157.5	165.8	-1.7(-1.8,-1.7)
	Other nutritional deficiencies	4374.9	12.2	12.6	-0.8(-1.1,-0.5)	2129.0	11.9	12.3	-0.7(-1.1,-0.4)	2245.9	12.4	12.9	-0.9(-1.2,-0.6)
Palestine	Overall nutritional deficiencies	11115.1	224.2	219.8	-2.1(-2.2,-2.0)	6691.4	275.2	281.7	-1.9(-2.0,-1.8)	4423.7	175.2	158.5	-2.2(-2.3,-2.2)
	Protein-energy malnutrition	1388.6	28.0	31.8	-2.1(-2.4,-1.9)	718.2	29.5	32.7	-2.2(-2.4,-1.9)	670.4	26.5	31.6	-2.0(-2.3,-1.7)
	Iodine deficiency	201.9	4.1	4.3	-7.3(-8.4,-6.2)	124.0	5.1	5.4	-7.0(-8.1,-5.9)	77.9	3.1	3.3	-7.7(-8.8,-6.5)
	Vitamin A deficiency	582.2	11.7	9.7	-4.4(-4.7,-4.1)	270.1	11.1	9.3	-3.6(-3.9,-3.4)	312.1	12.4	10.2	-4.9(-5.3,-4.6)
	Dietary iron deficiency	8620.4	173.9	166.9	-1.7(-1.8,-1.5)	5395.6	221.9	226.2	-1.6(-1.8,-1.4)	3224.8	127.7	107.4	-1.6(-1.7,-1.4)
	Other nutritional deficiencies	322.0	6.5	7.1	-1.1(-1.5,-0.7)	183.5	7.5	8.2	-0.9(-1.3,-0.5)	138.5	5.5	6.1	-1.3(-1.7,-0.9)
Syrian Arab Republic	Overall nutritional deficiencies	46851.1	323.3	337.8	-2.3(-2.7,-1.9)	29139.7	392.0	404.6	-2.2(-2.5,-1.9)	17711.4	250.9	265.9	-2.5(-2.9,-2.2)
	Protein-energy malnutrition	10224.4	70.6	80.7	-3.1(-3.7,-2.6)	4839.2	65.1	77.0	-3.2(-3.7,-2.6)	5385.2	76.3	84.4	-3.1(-3.7,-2.5)
	Iodine deficiency	4919.8	34.0	32.9	-1.8(-2.2,-1.4)	2908.2	39.1	37.5	-1.7(-2.1,-1.3)	2011.6	28.5	27.8	-2.0(-2.5,-1.6)
	Vitamin A deficiency	460.4	3.2	3.6	-4.8(-5.0,-4.5)	199.7	2.7	3.2	-4.6(-4.9,-4.4)	260.7	3.7	4.1	-4.9(-5.2,-4.6)
	Dietary iron deficiency	28676.6	197.9	201.9	-2.0(-2.3,-1.7)	19900.9	267.7	268.3	-1.9(-2.2,-1.6)	8775.7	124.3	130.7	-2.2(-2.4,-2.0)

	Other nutritional deficiencies	2569.9	17.7	18.7	-1.8(-2.0,-1.5)	1291.8	17.4	18.6	-1.8(-2.0,-1.6)	1278.2	18.1	18.9	-1.7(-2.0,-1.3)
Tunisia	Overall nutritional deficiencies	17071.9	147.5	156.9	-2.2(-2.4,-2.1)	10364.9	178.4	186.2	-2.3(-2.4,-2.1)	6707.1	116.4	126.7	-2.2(-2.3,-2.1)
	Protein-energy malnutrition	3590.2	31.0	33.7	-2.6(-3.0,-2.2)	1820.5	31.3	34.4	-3.2(-3.7,-2.7)	1769.7	30.7	33.1	-2.0(-2.2,-1.7)
	Iodine deficiency	868.7	7.5	7.2	-3.0(-3.2,-2.8)	467.2	8.0	7.6	-2.6(-2.9,-2.4)	401.5	7.0	6.7	-3.5(-3.7,-3.3)
	Vitamin A deficiency	490.0	4.2	4.7	-2.5(-2.7,-2.3)	236.2	4.1	4.6	-2.2(-2.4,-2.0)	253.8	4.4	4.8	-2.7(-3.0,-2.5)
	Dietary iron deficiency	11057.6	95.6	102.3	-2.1(-2.2,-2.1)	7308.7	125.8	130.8	-2.1(-2.1,-2.0)	3748.8	65.1	73.0	-2.3(-2.4,-2.3)
	Other nutritional deficiencies	1065.5	9.2	9.1	-0.5(-1.0,0.1)	532.3	9.2	8.9	-0.5(-1.1,0.1)	533.3	9.3	9.2	-0.4(-1.0,0.1)
Turkey	Overall nutritional deficiencies	180408.7	221.7	240.3	-3.3(-3.6,-3.1)	125519.3	311.8	322.1	-3.2(-3.4,-3.0)	54889.4	133.5	160.8	-3.6(-3.9,-3.3)
	Protein-energy malnutrition	27571.8	33.9	38.1	-4.4(-4.9,-3.8)	15424.3	38.3	42.6	-4.3(-4.9,-3.6)	12147.6	29.6	34.0	-4.5(-4.9,-4.0)
	Iodine deficiency	18395.1	22.6	21.1	-3.1(-3.3,-2.9)	10486.6	26.0	24.4	-2.9(-3.0,-2.7)	7908.5	19.2	18.0	-3.4(-3.6,-3.1)
	Vitamin A deficiency	1060.2	1.3	2.0	-6.7(-6.8,-6.5)	602.6	1.5	2.3	-5.8(-5.9,-5.6)	457.6	1.1	1.7	-7.6(-7.8,-7.4)
	Dietary iron deficiency	123440.0	151.7	166.8	-3.2(-3.4,-3.0)	93654.0	232.6	240.3	-3.1(-3.3,-2.9)	29786.0	72.5	95.0	-3.4(-3.6,-3.2)
	Other nutritional deficiencies	9941.5	12.2	12.2	0.6(-0.2,1.4)	5351.8	13.3	12.5	1.5(0.7,2.4)	4589.7	11.2	12.0	-0.2(-0.9,0.6)
Yemen	Overall nutritional deficiencies	418986.4	1330.0	1162.8	-2.2(-2.5,-2.0)	247970.8	1592.2	1467.7	-1.8(-2.0,-1.5)	171015.6	1073.6	858.6	-3.0(-3.1,-2.8)
	Protein-energy malnutrition	85810.0	272.4	235.0	-6.4(-6.5,-6.2)	41713.6	267.8	225.5	-6.6(-6.8,-6.5)	44096.3	276.8	245.4	-6.1(-6.3,-5.9)
	Iodine deficiency	20671.1	65.6	68.2	-1.5(-2.0,-0.9)	11775.1	75.6	77.4	-1.3(-1.7,-0.8)	8896.1	55.8	59.1	-1.7(-2.3,-1.2)
	Vitamin A deficiency	15568.5	49.4	36.3	-2.6(-2.9,-2.3)	6223.6	40.0	30.0	-2.7(-3.1,-2.4)	9344.9	58.7	42.3	-2.5(-2.8,-2.2)
	Dietary iron deficiency	291052.9	923.9	801.6	1.2(1.1,1.4)	186112.6	1195.0	1119.5	1.4(1.2,1.5)	104940.3	658.8	483.5	0.9(0.7,1.0)
	Other nutritional deficiencies	5883.9	18.7	21.7	-3.0(-3.3,-2.8)	2145.9	13.8	15.3	-3.0(-3.2,-2.8)	3738.0	23.5	28.3	-3.1(-3.3,-2.8)
Afghanistan	Overall nutritional deficiencies	264468.4	690.9	546.8	-3.2(-3.6,-2.8)	161226.2	865.0	722.2	-3.2(-3.6,-2.9)	103242.3	525.7	372.8	-3.2(-3.7,-2.7)
	Protein-energy malnutrition	116434.0	304.2	202.0	-5.1(-5.7,-4.5)	74737.0	401.0	271.0	-5.1(-5.6,-4.5)	41697.0	212.3	137.1	-5.2(-5.9,-4.4)
	Iodine deficiency	18407.7	48.1	52.3	-1.0(-1.7,-0.2)	10680.7	57.3	61.8	-0.9(-1.6,-0.1)	7727.0	39.3	43.2	-1.1(-1.9,-0.3)
	Vitamin A deficiency	17488.5	45.7	28.6	-1.4(-1.8,-1.0)	8196.8	44.0	27.8	-1.1(-1.6,-0.7)	9291.7	47.3	29.4	-1.6(-2.0,-1.3)
	Dietary iron deficiency	106812.2	279.0	250.1	-1.1(-1.2,-1.1)	65023.6	348.8	348.2	-1.3(-1.3,-1.2)	41788.6	212.8	149.2	-1.0(-1.1,-0.9)
	Other nutritional deficiencies	5326.0	13.9	13.8	-1.6(-1.9,-1.3)	2588.0	13.9	13.4	-1.8(-2.1,-1.4)	2738.0	13.9	14.0	-1.4(-1.8,-1.1)
Bangladesh	Overall nutritional deficiencies	1107557.0	695.4	744.4	-6.0(-6.2,-5.8)	692193.6	857.8	927.3	-5.8(-6.0,-5.6)	415363.5	528.7	560.8	-6.4(-6.5,-6.2)
	Protein-energy malnutrition	194737.3	122.3	140.0	-10.4(-10.7,-10.1)	117336.4	145.4	173.5	-10.2(-10.5,-9.9)	77400.9	98.5	108.4	-10.7(-11.1,-10.3)
	Iodine deficiency	64323.0	40.4	39.4	-2.9(-3.4,-2.4)	39529.2	49.0	47.4	-2.6(-3.1,-2.0)	24793.8	31.6	31.1	-3.5(-3.8,-3.1)
	Vitamin A deficiency	14216.0	8.9	9.4	-4.3(-4.5,-4.1)	7747.5	9.6	10.4	-3.2(-3.5,-2.9)	6468.5	8.2	8.5	-5.2(-5.4,-5.0)
	Dietary iron deficiency	732424.9	459.9	488.7	-1.8(-1.9,-1.8)	444746.6	551.2	588.1	-1.7(-1.7,-1.6)	287678.3	366.1	386.5	-2.2(-2.2,-2.1)
	Other nutritional deficiencies	101855.9	64.0	66.8	-5.1(-5.4,-4.8)	82833.9	102.7	107.9	-3.8(-4.1,-3.5)	19022.0	24.2	26.3	-7.9(-8.2,-7.5)
Bhutan	Overall nutritional deficiencies	8300.7	1100.5	1193.2	-2.0(-2.1,-1.9)	5027.6	1390.9	1463.5	-2.1(-2.2,-2.0)	3273.1	833.3	944.8	-1.7(-1.7,-1.6)
	Protein-energy malnutrition	590.9	78.3	83.6	-7.7(-8.1,-7.3)	307.1	85.0	91.8	-8.4(-8.7,-8.1)	283.8	72.3	75.6	-6.3(-6.8,-5.8)

	Iodine deficiency	47.2	6.3	5.9	-5.7(-6.1,-5.2)	33.8	9.4	8.9	-5.5(-5.9,-5.1)	13.4	3.4	3.2	-5.9(-6.4,-5.4)
	Vitamin A deficiency	111.2	14.7	16.4	-4.7(-4.9,-4.6)	54.8	15.1	16.4	-4.1(-4.3,-3.9)	56.5	14.4	16.4	-5.3(-5.4,-5.1)
	Dietary iron deficiency	7292.3	966.8	1050.0	-0.5(-0.6,-0.4)	4479.4	1239.2	1299.4	-0.4(-0.5,-0.3)	2812.9	716.2	821.7	-0.6(-0.8,-0.5)
	Other nutritional deficiencies	259.1	34.3	37.2	-4.5(-4.8,-4.3)	152.5	42.2	47.0	-5.2(-5.4,-5.1)	106.6	27.1	27.9	-2.4(-2.9,-2.0)
India	Overall nutritional deficiencies	16416561.2	1180.4	1242.0	-3.3(-3.5,-3.2)	10209716.6	1506.9	1568.8	-3.0(-3.2,-2.9)	6206844.6	870.3	928.4	-3.9(-4.1,-3.7)
	Protein-energy malnutrition	2570193.1	184.8	208.0	-7.0(-7.2,-6.8)	1374337.9	202.8	234.2	-6.9(-7.0,-6.8)	1195855.1	167.7	183.8	-7.2(-7.4,-6.9)
	Iodine deficiency	1100369.1	79.1	76.4	-2.4(-2.6,-2.3)	641103.6	94.6	91.0	-2.2(-2.4,-2.1)	459265.5	64.4	62.3	-2.7(-3.0,-2.5)
	Vitamin A deficiency	297500.8	21.4	22.9	-3.4(-3.8,-3.1)	135244.6	20.0	21.6	-3.0(-3.3,-2.6)	162256.2	22.8	24.1	-3.8(-4.1,-3.5)
	Dietary iron deficiency	11659376.9	838.4	873.3	-0.6(-0.7,-0.6)	7607542.1	1122.8	1150.3	-0.4(-0.5,-0.3)	4051834.8	568.1	606.9	-1.1(-1.2,-1.0)
	Other nutritional deficiencies	789121.3	56.7	61.4	-7.2(-7.6,-6.7)	451488.3	66.6	71.7	-7.3(-7.7,-6.9)	337633.0	47.3	51.2	-7.1(-7.6,-6.6)
Nepal	Overall nutritional deficiencies	248399.3	816.7	882.3	-4.3(-4.6,-4.0)	150192.0	943.5	1027.1	-4.1(-4.4,-3.8)	98207.3	677.4	720.6	-4.5(-4.8,-4.3)
	Protein-energy malnutrition	56101.9	184.4	208.0	-7.8(-8.1,-7.6)	31825.6	199.9	231.8	-7.7(-8.0,-7.4)	24276.3	167.4	183.4	-8.0(-8.2,-7.7)
	Iodine deficiency	4070.5	13.4	13.6	0.5(-0.1,1.2)	2637.2	16.6	16.3	1.2(0.3,2.0)	1433.3	9.9	10.4	-0.4(-0.8,-0.1)
	Vitamin A deficiency	3320.8	10.9	10.7	-4.5(-4.7,-4.3)	2034.0	12.8	13.2	-3.7(-3.9,-3.5)	1286.8	8.9	8.3	-5.5(-5.7,-5.2)
	Dietary iron deficiency	171756.8	564.7	598.6	-0.7(-0.8,-0.7)	106202.1	667.2	707.7	-0.8(-0.9,-0.8)	65554.7	452.2	474.0	-0.7(-0.8,-0.6)
	Other nutritional deficiencies	13149.3	43.2	51.3	-5.2(-5.6,-4.7)	7493.1	47.1	58.1	-4.5(-4.9,-4.1)	5656.2	39.0	44.4	-5.9(-6.4,-5.4)
Pakistan	Overall nutritional deficiencies	3165218.6	1412.6	1341.4	-1.0(-1.1,-0.9)	1763533.5	1614.7	1588.9	-1.0(-1.1,-0.9)	1401685.1	1220.5	1102.1	-1.1(-1.2,-1.0)
	Protein-energy malnutrition	823837.8	367.7	287.4	-2.3(-2.7,-1.8)	414277.4	379.3	304.1	-2.5(-2.9,-2.1)	409560.4	356.6	271.6	-2.1(-2.6,-1.5)
	Iodine deficiency	169197.1	75.5	85.0	0.0(-0.2,0.1)	134812.2	123.4	138.2	0.3(0.2,0.5)	34384.9	29.9	33.8	-1.5(-1.7,-1.3)
	Vitamin A deficiency	42649.0	19.0	16.1	-3.8(-4.2,-3.5)	17813.2	16.3	13.7	-3.1(-3.3,-2.9)	24835.8	21.6	18.2	-4.3(-4.7,-3.9)
	Dietary iron deficiency	1833961.4	818.5	796.4	-0.4(-0.6,-0.3)	997097.9	913.0	915.7	-0.4(-0.5,-0.2)	836863.4	728.7	680.2	-0.6(-0.8,-0.4)
	Other nutritional deficiencies	295573.3	131.9	156.5	-0.9(-1.1,-0.7)	199532.7	182.7	217.1	-1.2(-1.4,-0.9)	96040.5	83.6	98.3	-0.5(-0.7,-0.3)
Angola	Overall nutritional deficiencies	376306.8	1248.6	1064.6	-6.3(-6.5,-6.2)	188748.6	1216.5	1053.2	-5.7(-5.8,-5.5)	187558.3	1282.7	1080.9	-6.9(-7.0,-6.8)
	Protein-energy malnutrition	206858.0	686.4	562.6	-7.9(-8.2,-7.6)	95648.0	616.5	502.0	-7.4(-7.7,-7.1)	111210.0	760.5	633.7	-8.3(-8.5,-8.0)
	Iodine deficiency	23125.6	76.7	87.7	-3.2(-3.8,-2.7)	15702.9	101.2	114.9	-3.6(-4.1,-3.0)	7422.7	50.8	57.3	-2.7(-3.2,-2.2)
	Vitamin A deficiency	12919.3	42.9	29.2	-2.1(-2.4,-1.8)	5638.1	36.3	25.6	-2.2(-2.5,-1.8)	7281.2	49.8	32.9	-2.0(-2.3,-1.7)
	Dietary iron deficiency	128596.6	426.7	358.1	-0.4(-0.5,-0.3)	68928.7	444.2	380.6	-0.3(-0.4,-0.3)	59667.9	408.1	334.1	-0.4(-0.5,-0.4)
	Other nutritional deficiencies	4807.4	16.0	26.9	-7.0(-7.3,-6.7)	2830.9	18.2	30.1	-5.8(-5.9,-5.7)	1976.5	13.5	22.9	-8.3(-8.8,-7.8)
Central African Republic	Overall nutritional deficiencies	131346.8	2478.3	2067.3	-0.2(-0.4,0.1)	62691.5	2317.7	1940.3	-0.2(-0.4,0.0)	68655.3	2645.7	2213.8	-0.1(-0.4,0.2)
	Protein-energy malnutrition	88302.4	1666.1	1328.8	0.0(-0.5,0.4)	40396.9	1493.5	1169.6	-0.1(-0.5,0.3)	47905.5	1846.1	1510.8	0.0(-0.5,0.5)
	Iodine deficiency	4691.2	88.5	95.7	-1.9(-2.2,-1.5)	2904.4	107.4	116.0	-1.7(-2.0,-1.5)	1786.8	68.9	73.5	-2.1(-2.6,-1.6)
	Vitamin A deficiency	5630.6	106.2	72.2	-1.1(-1.2,-0.9)	2101.7	77.7	54.4	-1.1(-1.3,-0.8)	3528.9	136.0	89.8	-1.0(-1.1,-1.0)
	Dietary iron deficiency	31111.8	587.0	524.2	0.3(0.1,0.4)	16406.0	606.5	552.0	0.3(0.1,0.4)	14705.8	566.7	496.2	0.2(0.1,0.3)

	Other nutritional deficiencies	1610.9	30.4	46.5	-2.2(-2.4,-2.1)	882.5	32.6	48.3	-1.9(-2.0,-1.7)	728.3	28.1	43.5	-2.8(-2.9,-2.6)
Congo	Overall nutritional deficiencies	50680.0	962.4	976.1	-2.2(-2.4,-2.0)	29677.3	1117.4	1148.4	-1.8(-2.0,-1.6)	21002.7	804.7	803.6	-2.7(-2.9,-2.5)
	Protein-energy malnutrition	12876.6	244.5	288.7	-4.1(-4.5,-3.7)	6136.5	231.0	278.6	-3.8(-4.2,-3.3)	6740.1	258.3	298.7	-4.4(-4.8,-4.0)
	Iodine deficiency	5957.4	113.1	119.2	-1.2(-1.4,-1.0)	4225.6	159.1	167.5	-1.5(-1.6,-1.3)	1731.8	66.4	69.7	-0.1(-0.5,0.2)
	Vitamin A deficiency	3673.5	69.8	55.1	-1.4(-1.7,-1.1)	1454.5	54.8	43.8	-1.5(-1.9,-1.0)	2219.0	85.0	66.3	-1.3(-1.6,-1.1)
	Dietary iron deficiency	27495.4	522.1	493.4	-0.4(-0.5,-0.3)	17452.9	657.1	635.0	-0.2(-0.3,-0.1)	10042.5	384.8	353.2	-0.7(-0.8,-0.6)
	Other nutritional deficiencies	677.0	12.9	19.8	-5.1(-5.4,-4.8)	407.7	15.4	23.5	-4.6(-4.9,-4.3)	269.3	10.3	15.7	-5.8(-6.2,-5.3)
Democratic Republic of the Congo	Overall nutritional deficiencies	1158522.3	1321.5	1215.9	-2.7(-3.2,-2.3)	590095.4	1347.6	1270.2	-2.5(-2.9,-2.2)	568426.8	1295.3	1165.8	-2.9(-3.5,-2.4)
	Protein-energy malnutrition	425770.4	485.6	438.3	-4.3(-5.1,-3.6)	190736.2	435.6	387.4	-4.3(-5.0,-3.6)	235034.2	535.6	496.8	-4.3(-5.1,-3.5)
	Iodine deficiency	154919.6	176.7	193.1	-0.6(-1.1,-0.2)	97838.4	223.4	245.5	-0.8(-1.2,-0.4)	57081.3	130.1	139.4	-0.2(-0.7,0.3)
	Vitamin A deficiency	71779.8	81.9	55.7	-0.6(-1.2,-0.1)	30057.0	68.6	47.2	-0.4(-1.0,0.2)	41722.7	95.1	64.1	-0.8(-1.3,-0.3)
	Dietary iron deficiency	490091.7	559.0	500.3	-0.8(-1.0,-0.7)	261678.0	597.6	557.3	-0.8(-1.0,-0.6)	228413.8	520.5	441.7	-0.8(-1.0,-0.7)
	Other nutritional deficiencies	15960.7	18.2	28.5	-3.9(-4.1,-3.6)	9785.9	22.3	32.8	-3.7(-3.9,-3.6)	6174.9	14.1	23.8	-4.1(-4.4,-3.7)
Equatorial Guinea	Overall nutritional deficiencies	8466.5	596.3	599.2	-6.8(-7.4,-6.2)	4319.1	661.6	661.9	-6.2(-6.6,-5.7)	4147.4	540.8	531.1	-7.6(-8.3,-6.9)
	Protein-energy malnutrition	1800.3	126.8	154.8	-10.0(-10.6,-9.4)	891.9	136.6	155.3	-9.2(-9.6,-8.7)	908.4	118.4	153.8	-10.9(-11.6,-10.2)
	Iodine deficiency	822.2	57.9	62.4	-8.1(-9.0,-7.1)	522.2	80.0	85.1	-7.9(-8.8,-6.9)	300.0	39.1	40.5	-8.2(-9.3,-7.1)
	Vitamin A deficiency	334.2	23.5	20.5	-7.5(-7.9,-7.1)	136.5	20.9	18.6	-7.3(-7.7,-6.9)	197.7	25.8	22.2	-7.6(-8.1,-7.2)
	Dietary iron deficiency	5402.0	380.5	348.3	-2.3(-2.5,-2.1)	2706.1	414.5	387.5	-2.3(-2.5,-2.1)	2696.0	351.5	304.7	-2.5(-2.7,-2.3)
	Other nutritional deficiencies	107.8	7.6	13.2	-8.0(-8.7,-7.2)	62.4	9.6	15.5	-7.0(-7.5,-6.5)	45.4	5.9	10.0	-9.6(-10.8,-8.5)
Gabon	Overall nutritional deficiencies	14530.9	830.3	855.1	-1.7(-2.0,-1.5)	8362.7	924.3	974.0	-1.4(-1.6,-1.2)	6168.1	729.7	729.0	-2.2(-2.4,-1.9)
	Protein-energy malnutrition	2604.5	148.8	180.5	-4.0(-4.4,-3.7)	1079.8	119.3	147.2	-4.3(-4.7,-3.8)	1524.7	180.4	216.2	-3.8(-4.1,-3.6)
	Iodine deficiency	565.6	32.3	32.4	-4.8(-5.4,-4.2)	380.5	42.1	42.1	-5.2(-5.9,-4.5)	185.2	21.9	21.9	-4.0(-4.5,-3.5)
	Vitamin A deficiency	303.2	17.3	16.0	-3.6(-3.8,-3.5)	136.1	15.0	14.2	-3.0(-3.1,-2.9)	167.1	19.8	17.9	-4.1(-4.2,-4.0)
	Dietary iron deficiency	10861.1	620.6	610.4	-0.1(-0.3,0.1)	6654.7	735.5	753.7	0.2(-0.1,0.4)	4206.4	497.6	458.8	-0.5(-0.8,-0.3)
	Other nutritional deficiencies	196.4	11.2	15.8	-4.1(-4.2,-4.0)	111.7	12.3	16.8	-4.2(-4.4,-4.0)	84.8	10.0	14.2	-3.9(-4.1,-3.8)
Burundi	Overall nutritional deficiencies	206925.3	1733.9	1438.6	-3.6(-3.9,-3.3)	96353.8	1604.5	1363.9	-3.5(-3.7,-3.2)	110571.6	1864.9	1516.8	-3.7(-4.0,-3.3)
	Protein-energy malnutrition	138579.1	1161.2	933.3	-4.4(-4.8,-3.9)	62877.2	1047.0	822.4	-4.4(-4.8,-4.1)	75701.8	1276.8	1042.7	-4.4(-4.8,-3.9)
	Iodine deficiency	4450.8	37.3	45.0	-2.3(-2.5,-2.1)	2526.9	42.1	50.8	-2.3(-2.5,-2.0)	1923.9	32.4	39.2	-2.3(-2.5,-2.0)
	Vitamin A deficiency	5736.7	48.1	30.3	-2.0(-2.3,-1.8)	2628.4	43.8	27.8	-1.8(-2.1,-1.5)	3108.3	52.4	32.8	-2.2(-2.5,-1.9)
	Dietary iron deficiency	55246.8	462.9	390.8	0.0(-0.2,0.1)	26705.2	444.7	419.4	-0.1(-0.2,0.1)	28541.6	481.4	367.3	0.1(0.0,0.3)
	Other nutritional deficiencies	2911.9	24.4	39.2	-5.6(-6.0,-5.2)	1616.0	26.9	43.6	-5.0(-5.4,-4.6)	1295.9	21.9	34.7	-6.3(-6.7,-5.8)
Comoros	Overall nutritional deficiencies	7844.1	1098.1	1123.1	-3.3(-3.5,-3.2)	4497.4	1257.7	1287.2	-3.0(-3.2,-2.9)	3346.8	938.1	954.3	-3.7(-3.8,-3.5)
	Protein-energy malnutrition	3044.4	426.2	452.6	-4.8(-5.0,-4.7)	1718.2	480.5	499.1	-4.5(-4.7,-4.3)	1326.2	371.7	407.3	-5.2(-5.3,-5.0)

	Iodine deficiency	60.0	8.4	8.8	-0.5(-0.5,-0.4)	36.4	10.2	10.6	-0.4(-0.4,-0.3)	23.7	6.6	7.0	-0.6(-0.6,-0.5)
	Vitamin A deficiency	211.8	29.7	26.7	-2.9(-3.1,-2.7)	85.7	24.0	21.9	-2.9(-3.0,-2.7)	126.2	35.4	31.4	-3.0(-3.1,-2.8)
	Dietary iron deficiency	4381.4	613.3	609.5	-1.2(-1.3,-1.0)	2561.4	716.3	723.5	-1.1(-1.3,-0.9)	1820.0	510.1	490.3	-1.3(-1.4,-1.2)
	Other nutritional deficiencies	146.4	20.5	25.5	-5.3(-5.7,-4.9)	95.8	26.8	32.0	-5.0(-5.3,-4.6)	50.7	14.2	18.2	-5.9(-6.3,-5.4)
Djibouti	Overall nutritional deficiencies	22535.2	1873.6	1809.2	-3.8(-4.1,-3.5)	10634.4	1887.6	1809.0	-3.6(-3.9,-3.3)	11900.8	1861.2	1817.8	-4.1(-4.4,-3.8)
	Protein-energy malnutrition	13646.4	1134.6	1074.8	-4.7(-5.1,-4.3)	6036.3	1071.4	970.0	-4.7(-5.1,-4.2)	7610.1	1190.2	1171.4	-4.8(-5.2,-4.4)
	Iodine deficiency	1729.5	143.8	151.8	-0.4(-0.8,0.0)	941.7	167.2	174.6	-0.4(-0.8,0.0)	787.7	123.2	131.5	-0.3(-0.8,0.1)
	Vitamin A deficiency	336.8	28.0	23.0	-3.5(-3.7,-3.3)	145.1	25.8	21.2	-2.8(-3.0,-2.6)	191.7	30.0	24.6	-4.0(-4.2,-3.7)
	Dietary iron deficiency	6475.6	538.4	520.6	-1.3(-1.4,-1.1)	3356.6	595.8	606.4	-1.2(-1.4,-1.1)	3118.9	487.8	449.2	-1.3(-1.5,-1.1)
	Other nutritional deficiencies	346.9	28.8	39.0	-5.0(-5.2,-4.8)	154.6	27.4	36.8	-4.5(-4.7,-4.2)	192.3	30.1	41.1	-5.5(-5.8,-5.1)
Eritrea	Overall nutritional deficiencies	114884.4	1711.8	1698.7	-5.2(-5.6,-4.8)	57548.7	1724.9	1712.5	-4.7(-5.1,-4.2)	57335.8	1698.9	1680.6	-5.7(-6.1,-5.3)
	Protein-energy malnutrition	66065.2	984.4	989.6	-6.4(-6.8,-6.0)	31601.2	947.2	924.5	-6.0(-6.4,-5.5)	34464.0	1021.2	1066.9	-6.7(-7.1,-6.4)
	Iodine deficiency	669.7	10.0	11.5	-2.5(-2.8,-2.2)	395.7	11.9	13.5	-2.4(-2.7,-2.1)	274.0	8.1	9.5	-2.7(-3.0,-2.3)
	Vitamin A deficiency	2830.4	42.2	31.8	-3.0(-3.1,-2.9)	1204.2	36.1	27.5	-2.8(-2.9,-2.8)	1626.2	48.2	35.9	-3.1(-3.2,-3.0)
	Dietary iron deficiency	43352.5	646.0	617.0	-1.1(-1.1,-1.0)	23184.5	694.9	692.3	-1.0(-1.0,-0.9)	20168.0	597.6	527.9	-1.2(-1.3,-1.2)
	Other nutritional deficiencies	1966.6	29.3	48.8	-6.4(-6.8,-5.9)	1163.0	34.9	54.7	-5.5(-6.0,-5.0)	803.5	23.8	40.3	-7.5(-7.8,-7.1)
Ethiopia	Overall nutritional deficiencies	1320435.1	1227.3	1083.1	-5.8(-6.1,-5.6)	684657.8	1284.9	1154.8	-5.3(-5.6,-5.1)	635777.3	1170.8	1014.4	-6.3(-6.7,-6.0)
	Protein-energy malnutrition	605438.6	562.7	492.1	-7.8(-8.2,-7.3)	321600.9	603.5	518.6	-7.2(-7.6,-6.8)	283837.7	522.7	467.2	-8.3(-8.8,-7.7)
	Iodine deficiency	113899.2	105.9	127.0	-1.4(-2.0,-0.9)	62621.9	117.5	139.9	-1.6(-2.1,-1.1)	51277.2	94.4	114.1	-1.2(-1.9,-0.5)
	Vitamin A deficiency	62932.8	58.5	39.8	-2.5(-2.8,-2.2)	29234.7	54.9	37.9	-2.3(-2.6,-2.1)	33698.2	62.1	41.7	-2.7(-3.0,-2.4)
	Dietary iron deficiency	517902.8	481.4	396.0	-1.0(-1.1,-0.9)	258805.4	485.7	422.7	-1.0(-1.1,-0.9)	259097.4	477.1	370.5	-1.0(-1.1,-1.0)
	Other nutritional deficiencies	20261.7	18.8	28.1	-8.9(-9.4,-8.5)	12395.0	23.3	35.7	-8.2(-8.7,-7.7)	7866.8	14.5	20.8	-9.8(-10.2,-9.4)
Kenya	Overall nutritional deficiencies	481088.8	957.8	991.0	-2.4(-2.5,-2.2)	238551.8	948.0	994.4	-2.1(-2.3,-1.9)	242536.9	967.6	986.2	-2.7(-2.8,-2.6)
	Protein-energy malnutrition	290070.8	577.5	583.8	-3.2(-3.4,-3.0)	134884.3	536.0	538.1	-3.1(-3.4,-2.8)	155186.5	619.1	630.4	-3.3(-3.4,-3.2)
	Iodine deficiency	5715.3	11.4	12.8	0.2(-0.1,0.5)	3091.7	12.3	13.7	0.0(-0.4,0.3)	2623.6	10.5	12.0	0.4(0.2,0.7)
	Vitamin A deficiency	22486.0	44.8	34.9	-2.2(-2.4,-2.0)	9934.0	39.5	31.3	-2.2(-2.4,-1.9)	12552.1	50.1	38.5	-2.2(-2.4,-2.1)
	Dietary iron deficiency	153654.8	305.9	332.9	0.0(-0.1,0.0)	86209.5	342.6	386.2	0.3(0.3,0.4)	67445.2	269.1	277.7	-0.5(-0.6,-0.4)
	Other nutritional deficiencies	9161.9	18.2	26.5	-3.8(-4.0,-3.7)	4432.3	17.6	25.2	-3.8(-4.1,-3.6)	4729.5	18.9	27.6	-3.8(-3.9,-3.7)
Madagascar	Overall nutritional deficiencies	526964.4	1974.4	1813.5	-3.8(-4.0,-3.7)	274090.3	2054.4	1937.0	-3.7(-3.9,-3.5)	252874.1	1894.4	1685.9	-4.0(-4.2,-3.9)
	Protein-energy malnutrition	361867.2	1355.8	1194.3	-4.6(-4.7,-4.4)	189155.1	1417.8	1261.2	-4.4(-4.6,-4.2)	172712.1	1293.9	1127.1	-4.7(-4.9,-4.6)
	Iodine deficiency	8521.3	31.9	36.3	-0.2(-0.4,0.0)	4951.7	37.1	41.9	-0.1(-0.3,0.1)	3569.6	26.7	30.7	-0.3(-0.5,-0.1)
	Vitamin A deficiency	11299.9	42.3	30.3	-2.4(-2.6,-2.2)	5205.2	39.0	28.3	-1.9(-2.2,-1.6)	6094.7	45.7	32.2	-2.7(-3.0,-2.5)
	Dietary iron deficiency	135032.7	505.9	498.5	-0.8(-1.1,-0.6)	68210.7	511.3	537.2	-0.8(-1.1,-0.6)	66822.0	500.6	456.9	-0.9(-1.2,-0.6)

	Other nutritional deficiencies	10243.4	38.4	54.1	-4.4(-4.6,-4.1)	6567.6	49.2	68.4	-4.2(-4.4,-4.0)	3675.8	27.5	39.1	-4.7(-5.0,-4.4)
Malawi	Overall nutritional deficiencies	275694.1	1494.9	1397.7	-4.0(-4.3,-3.7)	143452.6	1514.2	1432.2	-3.8(-4.1,-3.5)	132241.5	1474.5	1367.6	-4.1(-4.4,-3.9)
	Protein-energy malnutrition	127210.3	689.8	665.4	-5.6(-5.9,-5.2)	58575.0	618.3	583.1	-5.7(-6.0,-5.3)	68635.3	765.3	763.8	-5.4(-5.7,-5.1)
	Iodine deficiency	6725.0	36.5	42.2	-2.0(-2.3,-1.7)	3745.1	39.5	45.3	-1.9(-2.2,-1.6)	2979.9	33.2	38.9	-2.1(-2.5,-1.8)
	Vitamin A deficiency	10794.7	58.5	41.0	-2.7(-2.9,-2.5)	5750.8	60.7	43.1	-2.2(-2.4,-1.9)	5043.9	56.2	39.0	-3.2(-3.4,-3.0)
	Dietary iron deficiency	127127.5	689.3	614.7	-0.6(-0.8,-0.5)	73030.4	770.9	721.9	-0.7(-0.9,-0.6)	54097.1	603.2	497.6	-0.6(-0.7,-0.4)
	Other nutritional deficiencies	3836.6	20.8	34.3	-5.3(-5.7,-4.9)	2351.3	24.8	38.8	-5.5(-6.0,-5.1)	1485.3	16.6	28.3	-5.2(-5.5,-4.8)
Mozambique	Overall nutritional deficiencies	464539.5	1573.2	1317.7	-4.6(-4.8,-4.4)	224899.4	1467.3	1271.5	-4.5(-4.7,-4.2)	239640.1	1687.6	1371.5	-4.7(-4.9,-4.5)
	Protein-energy malnutrition	229345.5	776.7	644.1	-6.3(-6.6,-6.1)	110776.3	722.7	581.9	-6.4(-6.6,-6.2)	118569.2	835.0	725.3	-6.2(-6.4,-6.0)
	Iodine deficiency	5192.7	17.6	22.2	-3.8(-4.1,-3.5)	3036.2	19.8	24.2	-3.8(-4.0,-3.5)	2156.5	15.2	20.0	-3.9(-4.2,-3.6)
	Vitamin A deficiency	22856.6	77.4	47.0	-2.8(-3.0,-2.7)	9735.1	63.5	40.0	-2.6(-2.8,-2.4)	13121.5	92.4	54.1	-3.0(-3.1,-2.8)
	Dietary iron deficiency	199371.6	675.2	564.5	-0.5(-0.6,-0.5)	97151.4	633.8	584.3	-0.5(-0.6,-0.4)	102220.2	719.8	534.4	-0.6(-0.7,-0.5)
	Other nutritional deficiencies	7773.1	26.3	39.8	-4.5(-4.9,-4.1)	4200.5	27.4	41.1	-4.5(-4.9,-4.1)	3572.6	25.2	37.7	-4.5(-4.8,-4.1)
Rwanda	Overall nutritional deficiencies	121566.6	958.1	941.0	-5.0(-5.5,-4.5)	65370.0	1003.0	983.6	-4.7(-5.1,-4.2)	56196.5	910.7	894.2	-5.4(-6.0,-4.8)
	Protein-energy malnutrition	71212.6	561.3	536.8	-6.2(-6.9,-5.6)	35012.3	537.2	514.6	-6.1(-6.8,-5.5)	36200.3	586.7	565.2	-6.4(-7.1,-5.6)
	Iodine deficiency	3601.8	28.4	32.3	-2.0(-2.4,-1.6)	2263.4	34.7	38.8	-2.0(-2.4,-1.6)	1338.4	21.7	24.9	-2.0(-2.4,-1.6)
	Vitamin A deficiency	2633.7	20.8	16.4	-3.3(-3.7,-2.8)	1378.9	21.2	17.0	-2.8(-3.3,-2.3)	1254.9	20.3	15.9	-3.7(-4.1,-3.2)
	Dietary iron deficiency	41882.5	330.1	330.1	-1.4(-1.6,-1.2)	25254.9	387.5	382.6	-1.2(-1.4,-1.0)	16627.6	269.5	269.5	-1.6(-1.8,-1.4)
	Other nutritional deficiencies	2235.9	17.6	25.4	-6.6(-7.2,-6.1)	1460.6	22.4	30.5	-5.8(-6.2,-5.3)	775.3	12.6	18.8	-8.0(-8.9,-7.2)
Somalia	Overall nutritional deficiencies	817524.2	4018.7	3205.1	-2.2(-2.9,-1.5)	384655.8	3858.8	3094.2	-2.2(-2.9,-1.5)	432868.4	4172.3	3345.9	-2.1(-2.8,-1.4)
	Protein-energy malnutrition	577176.5	2837.2	2048.1	-2.8(-3.6,-1.9)	256390.6	2572.1	1787.1	-2.9(-3.8,-2.0)	320785.9	3091.9	2355.5	-2.6(-3.4,-1.7)
	Iodine deficiency	44093.4	216.7	274.4	0.2(0.0,0.4)	23759.6	238.4	300.2	0.2(0.0,0.3)	20333.8	196.0	247.3	0.3(0.1,0.4)
	Vitamin A deficiency	27110.7	133.3	80.9	-0.6(-0.7,-0.6)	12266.9	123.1	75.6	-0.6(-0.6,-0.5)	14843.9	143.1	86.0	-0.7(-0.8,-0.6)
	Dietary iron deficiency	161662.4	794.7	739.8	-0.7(-0.8,-0.7)	88399.8	886.8	869.3	-0.9(-1.0,-0.8)	73262.6	706.2	595.4	-0.5(-0.6,-0.4)
	Other nutritional deficiencies	7481.1	36.8	61.9	-3.4(-4.3,-2.4)	3838.9	38.5	62.0	-3.3(-3.4,-3.2)	3642.2	35.1	61.6	-3.7(-5.1,-2.2)
United Republic of Tanzania	Overall nutritional deficiencies	891871.8	1572.0	1297.1	-3.4(-3.6,-3.1)	455425.9	1562.4	1345.2	-3.1(-3.4,-2.9)	436445.9	1582.1	1247.4	-3.6(-3.8,-3.4)
	Protein-energy malnutrition	452220.7	797.1	619.1	-4.5(-4.8,-4.3)	238321.4	817.6	637.5	-4.2(-4.5,-4.0)	213899.3	775.4	602.2	-4.8(-5.1,-4.6)
	Iodine deficiency	4488.3	7.9	9.5	-2.9(-3.3,-2.6)	2742.8	9.4	11.0	-2.8(-3.2,-2.5)	1745.5	6.3	7.8	-3.0(-3.4,-2.7)
	Vitamin A deficiency	21971.7	38.7	27.1	-2.3(-2.4,-2.1)	10951.4	37.6	26.8	-1.6(-1.8,-1.4)	11020.3	39.9	27.5	-2.8(-2.9,-2.7)
	Dietary iron deficiency	402348.6	709.2	613.3	-1.1(-1.3,-0.9)	196829.9	675.2	636.0	-1.2(-1.4,-1.0)	205518.8	745.0	588.3	-1.0(-1.2,-0.9)
	Other nutritional deficiencies	10842.5	19.1	28.1	-4.9(-5.3,-4.6)	6580.4	22.6	33.8	-4.7(-5.1,-4.3)	4262.1	15.4	21.6	-5.4(-5.8,-5.0)
Uganda	Overall nutritional deficiencies	468829.2	1140.2	940.3	-3.5(-3.7,-3.3)	216977.3	1038.4	895.3	-2.8(-3.0,-2.6)	251851.8	1245.4	987.7	-4.1(-4.3,-3.8)
	Protein-energy malnutrition	274430.0	667.4	519.6	-4.5(-4.8,-4.3)	115897.2	554.7	428.5	-3.9(-4.2,-3.7)	158532.8	783.9	623.2	-4.9(-5.2,-4.6)

	Iodine deficiency	6171.9	15.0	18.6	-2.9(-3.1,-2.8)	3653.3	17.5	21.0	-2.8(-3.0,-2.7)	2518.6	12.5	16.0	-3.1(-3.3,-2.9)
	Vitamin A deficiency	11983.7	29.1	18.8	-3.2(-3.3,-3.0)	5908.1	28.3	18.8	-2.7(-2.9,-2.6)	6075.6	30.0	18.8	-3.6(-3.7,-3.4)
	Dietary iron deficiency	170899.5	415.6	362.2	-1.0(-1.2,-0.8)	88337.3	422.8	403.5	-0.9(-1.1,-0.7)	82562.1	408.3	311.9	-1.3(-1.5,-1.1)
	Other nutritional deficiencies	5344.1	13.0	21.1	-4.5(-4.8,-4.2)	3181.4	15.2	23.5	-4.2(-4.5,-4.0)	2162.7	10.7	17.9	-4.9(-5.3,-4.6)
Zambia	Overall nutritional deficiencies	333620.7	1829.3	1680.7	-2.6(-2.7,-2.5)	146989.8	1594.1	1506.5	-2.9(-3.0,-2.9)	186630.9	2069.8	1861.9	-2.3(-2.4,-2.1)
	Protein-energy malnutrition	119981.7	657.9	618.8	-4.7(-4.9,-4.4)	53333.1	578.4	537.1	-5.0(-5.1,-4.8)	66648.5	739.2	705.2	-4.3(-4.7,-4.0)
	Iodine deficiency	3041.6	16.7	19.2	-2.2(-2.7,-1.8)	1751.9	19.0	21.7	-2.2(-2.7,-1.8)	1289.7	14.3	16.6	-2.2(-2.7,-1.8)
	Vitamin A deficiency	7871.0	43.2	31.6	-3.2(-3.6,-2.8)	3445.9	37.4	27.5	-2.8(-3.3,-2.4)	4425.1	49.1	35.8	-3.4(-3.8,-3.0)
	Dietary iron deficiency	199260.7	1092.6	979.7	0.5(0.3,0.6)	86501.0	938.1	884.8	-0.1(-0.1,0.0)	112759.7	1250.6	1077.2	0.9(0.7,1.1)
	Other nutritional deficiencies	3465.7	19.0	31.5	-5.0(-5.3,-4.7)	1957.9	21.2	35.4	-5.3(-5.6,-5.0)	1507.8	16.7	27.0	-4.5(-4.9,-4.1)
Botswana	Overall nutritional deficiencies	21271.1	909.5	933.5	-0.7(-0.9,-0.5)	10614.2	893.4	923.7	-0.6(-0.9,-0.4)	10656.9	926.1	947.1	-0.9(-1.0,-0.7)
	Protein-energy malnutrition	13174.2	563.3	583.5	-0.3(-0.6,0.1)	5850.2	492.4	514.5	0.0(-0.4,0.5)	7324.0	636.5	660.4	-0.6(-0.8,-0.3)
	Iodine deficiency	72.9	3.1	3.1	-2.1(-2.8,-1.4)	43.8	3.7	3.7	-2.0(-2.7,-1.3)	29.2	2.5	2.6	-2.2(-3.0,-1.5)
	Vitamin A deficiency	477.6	20.4	19.7	-2.7(-2.9,-2.6)	223.5	18.8	18.5	-2.4(-2.6,-2.3)	254.0	22.1	20.9	-3.0(-3.2,-2.9)
	Dietary iron deficiency	7295.4	311.9	313.8	-1.2(-1.4,-1.1)	4362.4	367.2	373.6	-1.2(-1.3,-1.1)	2933.1	254.9	249.9	-1.2(-1.4,-1.1)
	Other nutritional deficiencies	250.9	10.7	13.4	-2.4(-2.5,-2.3)	134.3	11.3	13.5	-2.0(-2.3,-1.7)	116.6	10.1	13.4	-2.7(-3.0,-2.4)
Lesotho	Overall nutritional deficiencies	22594.2	1080.2	1110.4	-1.1(-1.5,-0.7)	10650.4	1006.1	1033.8	-1.3(-1.8,-0.9)	11943.8	1156.2	1186.9	-0.9(-1.3,-0.6)
	Protein-energy malnutrition	12643.3	604.5	624.9	-1.3(-1.9,-0.7)	5281.5	498.9	513.8	-1.7(-2.5,-0.8)	7361.8	712.6	745.3	-1.0(-1.5,-0.5)
	Iodine deficiency	1030.7	49.3	50.3	-4.0(-4.7,-3.3)	602.0	56.9	57.9	-4.0(-4.7,-3.3)	428.8	41.5	42.2	-4.0(-4.7,-3.3)
	Vitamin A deficiency	673.0	32.2	30.1	-2.0(-2.1,-1.9)	271.4	25.6	24.3	-2.1(-2.3,-2.0)	401.5	38.9	35.9	-1.9(-2.0,-1.8)
	Dietary iron deficiency	7986.2	381.8	388.8	0.0(-0.2,0.2)	4348.2	410.8	420.6	-0.1(-0.3,0.1)	3637.9	352.2	348.9	0.1(0.0,0.3)
	Other nutritional deficiencies	261.1	12.5	16.3	-1.0(-1.4,-0.5)	147.3	13.9	17.2	-0.7(-1.3,-0.1)	113.7	11.0	14.6	-1.5(-1.9,-1.2)
Namibia	Overall nutritional deficiencies	21470.5	893.4	830.7	-2.4(-2.6,-2.1)	9862.2	795.6	742.6	-2.3(-2.5,-2.0)	11608.3	997.7	934.2	-2.4(-2.6,-2.2)
	Protein-energy malnutrition	12299.0	511.8	472.0	-2.7(-3.1,-2.2)	5026.0	405.5	368.3	-2.5(-3.1,-1.8)	7273.1	625.1	592.2	-2.7(-3.1,-2.4)
	Iodine deficiency	230.2	9.6	10.1	-1.5(-1.6,-1.4)	144.5	11.7	12.2	-1.5(-1.5,-1.4)	85.7	7.4	7.9	-1.6(-1.7,-1.5)
	Vitamin A deficiency	409.1	17.0	14.8	-2.4(-2.7,-2.2)	188.1	15.2	13.4	-2.3(-2.6,-1.9)	221.1	19.0	16.2	-2.6(-2.8,-2.3)
	Dietary iron deficiency	8276.9	344.4	320.4	-1.7(-1.9,-1.5)	4368.5	352.4	335.8	-1.9(-2.1,-1.7)	3908.5	335.9	303.6	-1.5(-1.7,-1.3)
	Other nutritional deficiencies	255.1	10.6	13.5	-3.3(-3.4,-3.2)	135.2	10.9	13.0	-3.5(-3.7,-3.3)	119.9	10.3	14.3	-3.1(-3.2,-2.9)
South Africa	Overall nutritional deficiencies	299660.0	539.1	575.0	-2.8(-3.1,-2.5)	168580.8	594.4	629.0	-2.3(-2.6,-2.1)	131079.1	481.4	518.2	-3.3(-3.6,-3.0)
	Protein-energy malnutrition	149540.6	269.0	294.9	-3.7(-4.3,-3.1)	64975.3	229.1	256.0	-3.6(-4.2,-3.0)	84565.3	310.6	337.3	-3.9(-4.5,-3.3)
	Iodine deficiency	3555.0	6.4	6.3	-1.9(-2.2,-1.7)	2196.2	7.7	7.5	-1.9(-2.2,-1.6)	1358.8	5.0	4.9	-2.0(-2.3,-1.7)
	Vitamin A deficiency	5237.8	9.4	9.7	-3.2(-3.4,-3.1)	2504.6	8.8	9.2	-2.7(-2.9,-2.5)	2733.2	10.0	10.2	-3.7(-3.8,-3.5)
	Dietary iron deficiency	135248.1	243.3	252.7	-1.1(-1.4,-0.8)	96669.7	340.9	348.1	-0.9(-1.1,-0.7)	38578.3	141.7	150.7	-1.5(-1.9,-1.2)

	Other nutritional deficiencies	6078.4	10.9	11.5	-4.0(-4.3,-3.7)	2234.9	7.9	8.2	-4.5(-4.8,-4.1)	3843.5	14.1	15.0	-3.8(-4.1,-3.4)
Eswatini	Overall nutritional deficiencies	10350.6	906.3	847.2	-1.6(-1.8,-1.3)	4632.0	792.8	768.0	-1.5(-1.8,-1.2)	5718.6	1025.1	927.3	-1.6(-1.8,-1.4)
	Protein-energy malnutrition	6618.6	579.5	523.3	-1.9(-2.2,-1.6)	2618.6	448.2	410.3	-1.9(-2.4,-1.5)	3999.9	717.0	646.4	-1.9(-2.1,-1.6)
	Iodine deficiency	102.7	9.0	9.9	-2.4(-2.9,-1.8)	64.6	11.0	11.8	-2.2(-2.6,-1.7)	38.1	6.8	7.7	-2.6(-3.2,-2.1)
	Vitamin A deficiency	227.7	19.9	17.0	-2.8(-2.9,-2.7)	98.5	16.9	15.0	-2.5(-2.6,-2.3)	129.3	23.2	19.1	-3.0(-3.1,-2.9)
	Dietary iron deficiency	3306.3	289.5	285.5	-0.6(-0.8,-0.4)	1801.3	308.3	319.9	-0.6(-0.8,-0.4)	1505.0	269.8	242.1	-0.6(-0.8,-0.4)
	Other nutritional deficiencies	95.3	8.3	11.5	-2.4(-2.6,-2.1)	49.1	8.4	11.0	-2.7(-3.0,-2.4)	46.3	8.3	11.9	-2.0(-2.2,-1.7)
Zimbabwe	Overall nutritional deficiencies	257248.8	1713.8	1604.1	1.3(0.8,1.9)	126018.1	1614.9	1525.4	1.9(1.1,2.6)	131230.7	1820.8	1710.9	1.0(0.6,1.3)
	Protein-energy malnutrition	183486.4	1222.4	1130.3	1.6(0.9,2.3)	82526.8	1057.5	960.2	2.7(1.4,3.9)	100959.7	1400.8	1344.4	1.1(0.7,1.5)
	Iodine deficiency	4153.2	27.7	31.7	-0.3(-0.7,0.1)	2481.9	31.8	35.6	-0.3(-0.6,0.1)	1671.3	23.2	27.3	-0.4(-0.8,0.0)
	Vitamin A deficiency	6303.5	42.0	31.9	-0.2(-0.5,0.2)	3079.1	39.5	31.0	0.2(-0.2,0.6)	3224.4	44.7	32.9	-0.5(-0.8,-0.2)
	Dietary iron deficiency	61229.9	407.9	390.8	1.1(0.8,1.4)	36915.4	473.0	480.7	1.0(0.7,1.4)	24314.5	337.4	285.3	1.0(0.7,1.4)
	Other nutritional deficiencies	2075.8	13.8	19.5	-0.4(-0.9,0.1)	1015.0	13.0	18.0	0.2(-0.6,0.9)	1060.8	14.7	21.1	-0.9(-1.2,-0.6)
Benin	Overall nutritional deficiencies	144838.8	1143.5	862.0	-4.2(-4.5,-3.9)	77012.4	1196.0	945.9	-4.0(-4.3,-3.6)	67826.4	1089.3	773.5	-4.5(-4.8,-4.2)
	Protein-energy malnutrition	64289.3	507.6	359.9	-6.4(-6.7,-6.0)	32730.3	508.3	362.4	-6.3(-6.7,-5.9)	31559.0	506.8	358.2	-6.5(-6.8,-6.1)
	Iodine deficiency	1939.7	15.3	18.7	-1.5(-1.6,-1.4)	1212.3	18.8	22.5	-1.4(-1.5,-1.3)	727.4	11.7	14.6	-1.7(-1.8,-1.5)
	Vitamin A deficiency	10911.3	86.1	54.3	-1.7(-2.0,-1.4)	4172.5	64.8	42.1	-1.3(-1.6,-0.9)	6738.8	108.2	66.1	-1.9(-2.1,-1.7)
	Dietary iron deficiency	64879.4	512.2	405.3	-0.1(-0.3,0.1)	37187.8	577.5	489.2	-0.2(-0.4,0.1)	27691.6	444.7	316.8	0.0(-0.3,0.2)
	Other nutritional deficiencies	2819.2	22.3	23.8	-3.9(-4.2,-3.7)	1709.5	26.5	29.7	-4.2(-4.4,-4.0)	1109.7	17.8	17.7	-3.2(-3.6,-2.8)
Burkina Faso	Overall nutritional deficiencies	594414.5	2619.5	1801.9	-1.2(-1.7,-0.7)	314292.6	2696.1	1925.2	-0.9(-1.2,-0.5)	280121.9	2538.6	1672.4	-1.5(-2.2,-0.9)
	Protein-energy malnutrition	321172.5	1415.4	901.2	-2.3(-3.1,-1.5)	176561.4	1514.6	981.4	-2.0(-2.5,-1.4)	144611.1	1310.5	826.6	-2.7(-3.7,-1.7)
	Iodine deficiency	3724.0	16.4	20.2	-2.6(-2.8,-2.4)	2248.1	19.3	23.0	-2.5(-2.6,-2.3)	1475.9	13.4	17.0	-2.8(-3.0,-2.6)
	Vitamin A deficiency	24172.6	106.5	66.6	-2.3(-2.4,-2.1)	10073.0	86.4	56.4	-1.6(-1.9,-1.4)	14099.6	127.8	76.6	-2.7(-2.9,-2.5)
	Dietary iron deficiency	230226.5	1014.6	753.4	1.6(1.5,1.7)	114585.7	983.0	779.2	2.0(1.8,2.1)	115640.8	1048.0	718.9	1.2(1.1,1.3)
	Other nutritional deficiencies	15118.8	66.6	60.6	-1.8(-2.1,-1.4)	10824.4	92.9	85.3	-1.7(-1.9,-1.5)	4294.5	38.9	33.5	-1.8(-2.6,-1.0)
Cameroon	Overall nutritional deficiencies	220718.4	758.4	671.8	-2.2(-2.4,-2.1)	115010.3	787.0	720.6	-2.1(-2.3,-2.0)	105708.1	729.6	622.7	-2.3(-2.4,-2.1)
	Protein-energy malnutrition	66149.6	227.3	210.7	-4.5(-4.7,-4.3)	35625.8	243.8	222.0	-4.5(-4.8,-4.3)	30523.8	210.7	199.3	-4.4(-4.7,-4.2)
	Iodine deficiency	3624.6	12.5	14.2	-0.5(-0.7,-0.2)	2241.9	15.3	17.3	-0.4(-0.6,-0.2)	1382.7	9.5	11.1	-0.6(-0.8,-0.3)
	Vitamin A deficiency	16302.8	56.0	39.4	-2.8(-2.9,-2.7)	5404.2	37.0	26.7	-3.3(-3.5,-3.2)	10898.6	75.2	51.6	-2.5(-2.7,-2.3)
	Dietary iron deficiency	129752.7	445.9	386.7	0.4(0.2,0.6)	68697.9	470.1	428.8	0.7(0.4,0.9)	61054.8	421.4	345.1	0.1(-0.1,0.3)
	Other nutritional deficiencies	4888.7	16.8	20.8	-3.1(-3.2,-3.0)	3040.4	20.8	25.8	-3.5(-3.5,-3.4)	1848.2	12.8	15.5	-2.4(-2.6,-2.2)
Cabo Verde	Overall nutritional deficiencies	3132.1	555.8	571.5	-3.3(-3.6,-3.1)	1725.9	617.0	627.8	-3.5(-3.8,-3.2)	1406.1	495.4	523.7	-3.1(-3.2,-2.9)
	Protein-energy malnutrition	505.6	89.7	99.6	-7.3(-7.9,-6.8)	230.9	82.5	88.0	-8.4(-9.0,-7.8)	274.7	96.8	113.5	-6.2(-6.7,-5.7)

	Iodine deficiency	98.4	17.5	17.3	-3.0(-3.4,-2.6)	62.6	22.4	22.3	-2.6(-2.9,-2.3)	35.8	12.6	12.5	-3.4(-4.0,-2.8)
	Vitamin A deficiency	66.2	11.8	11.9	-4.9(-5.1,-4.6)	30.4	10.9	11.1	-3.5(-3.5,-3.4)	35.8	12.6	12.7	-5.8(-6.1,-5.5)
	Dietary iron deficiency	2394.5	424.9	430.0	-0.9(-1.0,-0.8)	1367.5	488.9	493.9	-0.8(-0.9,-0.7)	1027.0	361.8	372.1	-0.9(-1.0,-0.7)
	Other nutritional deficiencies	67.4	12.0	12.7	-7.0(-8.9,-5.1)	34.5	12.3	12.6	-8.0(-10.0,-5.9)	32.9	11.6	12.9	-5.5(-7.1,-4.0)
Chad	Overall nutritional deficiencies	362616.1	2211.2	1496.4	-4.3(-4.4,-4.1)	179597.8	2173.8	1521.4	-4.1(-4.3,-4.0)	183018.2	2249.2	1471.6	-4.4(-4.6,-4.3)
	Protein-energy malnutrition	194589.4	1186.6	747.5	-5.9(-6.2,-5.7)	96389.2	1166.7	733.1	-5.8(-6.1,-5.6)	98200.2	1206.8	760.2	-6.0(-6.3,-5.8)
	Iodine deficiency	2826.6	17.2	22.5	-3.1(-3.2,-2.9)	1630.8	19.7	25.4	-2.9(-3.1,-2.8)	1195.8	14.7	19.4	-3.2(-3.4,-3.0)
	Vitamin A deficiency	19433.4	118.5	67.0	-1.6(-1.8,-1.5)	7017.8	84.9	49.4	-1.8(-2.1,-1.6)	12415.6	152.6	84.3	-1.5(-1.6,-1.5)
	Dietary iron deficiency	140224.9	855.1	621.8	-0.1(-0.2,0.0)	71261.1	862.5	663.6	-0.1(-0.2,0.0)	68963.8	847.5	581.0	-0.1(-0.2,0.0)
	Other nutritional deficiencies	5541.7	33.8	37.7	-3.2(-3.4,-3.1)	3298.8	39.9	49.7	-3.2(-3.3,-3.0)	2242.9	27.6	26.6	-2.9(-3.1,-2.8)
C 幺 te d'Ivoire	Overall nutritional deficiencies	252050.5	963.1	823.7	-2.3(-2.5,-2.0)	125105.5	988.1	873.2	-2.3(-2.6,-2.0)	126945.0	939.6	780.0	-2.3(-2.5,-2.0)
	Protein-energy malnutrition	55065.9	210.4	184.8	-5.4(-5.7,-5.0)	28932.6	228.5	193.3	-5.3(-5.6,-5.0)	26133.4	193.4	175.8	-5.5(-5.8,-5.1)
	Iodine deficiency	4006.3	15.3	17.3	-1.8(-2.0,-1.6)	2916.4	23.0	26.0	-1.9(-2.0,-1.7)	1089.9	8.1	9.2	-1.5(-1.7,-1.2)
	Vitamin A deficiency	14312.4	54.7	38.7	-2.5(-2.7,-2.4)	6623.0	52.3	36.9	-2.1(-2.3,-1.9)	7689.5	56.9	40.4	-2.9(-3.0,-2.7)
	Dietary iron deficiency	175222.3	669.5	567.3	0.2(0.0,0.4)	84512.9	667.5	596.6	0.0(-0.2,0.2)	90709.5	671.4	543.5	0.4(0.3,0.6)
	Other nutritional deficiencies	3443.5	13.2	15.6	-3.4(-3.5,-3.3)	2120.6	16.7	20.5	-3.2(-3.3,-3.1)	1322.9	9.8	11.1	-3.7(-3.9,-3.5)
Gambia	Overall nutritional deficiencies	32354.7	1440.6	1242.1	-2.5(-2.6,-2.3)	17408.8	1525.7	1382.9	-2.2(-2.3,-2.0)	14946.0	1352.8	1097.7	-2.8(-3.0,-2.7)
	Protein-energy malnutrition	7170.0	319.3	294.3	-5.3(-5.6,-5.1)	3483.3	305.3	279.6	-5.3(-5.6,-5.0)	3686.7	333.7	309.0	-5.4(-5.7,-5.1)
	Iodine deficiency	960.5	42.8	48.8	-1.3(-1.7,-1.0)	593.6	52.0	59.5	-1.4(-1.7,-1.1)	366.9	33.2	37.6	-1.4(-1.7,-1.1)
	Vitamin A deficiency	1685.5	75.0	52.8	-2.7(-2.7,-2.7)	692.1	60.7	43.8	-2.3(-2.4,-2.3)	993.4	89.9	61.7	-2.9(-3.0,-2.9)
	Dietary iron deficiency	22096.1	983.9	820.4	-0.3(-0.4,-0.1)	12381.5	1085.1	968.3	-0.2(-0.3,0.0)	9714.6	879.3	669.7	-0.5(-0.6,-0.3)
	Other nutritional deficiencies	442.6	19.7	25.7	-2.7(-2.9,-2.4)	258.3	22.6	31.7	-2.9(-3.1,-2.6)	184.3	16.7	19.7	-2.4(-2.8,-2.1)
Ghana	Overall nutritional deficiencies	319554.5	1013.3	964.0	-2.7(-2.9,-2.5)	166842.2	1028.2	1019.9	-2.7(-2.9,-2.5)	152712.3	997.5	891.3	-2.7(-3.0,-2.5)
	Protein-energy malnutrition	120827.8	383.1	373.1	-4.5(-4.9,-4.2)	59496.8	366.7	366.9	-4.5(-4.8,-4.3)	61331.0	400.6	377.4	-4.5(-4.9,-4.1)
	Iodine deficiency	9269.6	29.4	31.3	-2.8(-3.0,-2.6)	5863.0	36.1	38.0	-2.8(-3.1,-2.6)	3406.6	22.3	23.8	-2.9(-3.1,-2.7)
	Vitamin A deficiency	13874.0	44.0	35.8	-3.0(-3.3,-2.8)	5119.7	31.6	26.9	-2.9(-3.3,-2.5)	8754.3	57.2	44.3	-3.1(-3.3,-2.9)
	Dietary iron deficiency	169559.7	537.7	499.6	0.4(0.3,0.6)	92001.6	567.0	554.5	0.1(-0.1,0.2)	77558.1	506.6	433.4	0.8(0.6,0.9)
	Other nutritional deficiencies	6023.4	19.1	24.1	-3.2(-3.3,-3.2)	4361.0	26.9	33.6	-3.8(-3.9,-3.7)	1662.4	10.9	12.4	-1.8(-2.1,-1.6)
Guinea	Overall nutritional deficiencies	235557.6	1863.1	1345.0	-3.2(-3.3,-3.1)	121746.4	1868.4	1428.2	-3.3(-3.4,-3.1)	113811.2	1857.5	1255.2	-3.2(-3.3,-3.1)
	Protein-energy malnutrition	134715.1	1065.5	702.7	-4.5(-4.7,-4.4)	67305.6	1032.9	709.4	-4.7(-4.9,-4.6)	67409.5	1100.2	695.9	-4.3(-4.5,-4.1)
	Iodine deficiency	4294.7	34.0	40.7	-3.5(-3.8,-3.3)	2575.1	39.5	46.6	-3.5(-3.8,-3.2)	1719.6	28.1	34.1	-3.6(-3.9,-3.4)
	Vitamin A deficiency	10196.6	80.6	51.4	-1.8(-1.8,-1.7)	3845.9	59.0	39.5	-1.5(-1.7,-1.4)	6350.7	103.6	63.1	-1.9(-2.0,-1.9)
	Dietary iron deficiency	82774.6	654.7	521.3	0.3(0.3,0.4)	45686.4	701.2	593.6	0.3(0.2,0.4)	37088.2	605.3	444.0	0.4(0.3,0.5)

	Other nutritional deficiencies	3576.6	28.3	28.9	-2.7(-2.8,-2.6)	2333.4	35.8	39.2	-2.5(-2.6,-2.5)	1243.2	20.3	18.2	-2.9(-3.0,-2.7)
Guinea-Bissau	Overall nutritional deficiencies	23634.3	1243.1	1102.2	-3.9(-4.1,-3.7)	12119.9	1241.2	1136.3	-3.6(-3.7,-3.4)	11514.4	1245.2	1063.9	-4.3(-4.4,-4.1)
	Protein-energy malnutrition	6292.4	331.0	327.2	-7.1(-7.3,-6.9)	2924.3	299.5	292.1	-7.1(-7.3,-6.9)	3368.2	364.2	367.1	-7.1(-7.3,-6.9)
	Iodine deficiency	833.2	43.8	50.2	-1.8(-2.0,-1.6)	533.5	54.6	61.8	-1.8(-2.0,-1.6)	299.7	32.4	37.1	-1.7(-1.9,-1.5)
	Vitamin A deficiency	1528.7	80.4	55.8	-2.0(-2.1,-2.0)	578.1	59.2	42.8	-1.8(-1.9,-1.7)	950.5	102.8	68.5	-2.1(-2.2,-2.1)
	Dietary iron deficiency	14573.5	766.5	639.6	0.8(0.6,1.0)	7803.9	799.2	700.7	0.9(0.7,1.0)	6769.5	732.1	572.3	0.8(0.6,1.0)
	Other nutritional deficiencies	406.5	21.4	29.3	-4.3(-4.5,-4.2)	280.0	28.7	38.8	-4.2(-4.3,-4.1)	126.5	13.7	18.8	-4.5(-4.8,-4.3)
Liberia	Overall nutritional deficiencies	38687.3	807.7	783.2	-5.5(-5.8,-5.2)	19136.0	804.8	819.8	-5.3(-5.6,-5.0)	19551.3	810.5	750.0	-5.7(-6.1,-5.4)
	Protein-energy malnutrition	17802.5	371.7	384.8	-7.3(-7.7,-7.0)	8549.2	359.6	383.2	-7.2(-7.5,-6.8)	9253.3	383.6	385.7	-7.5(-7.9,-7.1)
	Iodine deficiency	652.3	13.6	15.5	-2.4(-3.1,-1.7)	409.3	17.2	19.4	-2.2(-2.9,-1.5)	243.1	10.1	11.6	-2.7(-3.4,-1.9)
	Vitamin A deficiency	1742.7	36.4	27.8	-3.6(-3.9,-3.3)	523.8	22.0	17.4	-3.0(-3.5,-2.5)	1219.0	50.5	37.8	-3.9(-4.1,-3.7)
	Dietary iron deficiency	17472.0	364.8	324.3	-1.2(-1.3,-1.1)	8918.2	375.1	353.3	-1.5(-1.6,-1.4)	8553.7	354.6	298.6	-0.7(-0.8,-0.6)
	Other nutritional deficiencies	1017.8	21.2	30.9	-4.4(-4.6,-4.1)	735.5	30.9	46.5	-4.1(-4.4,-3.9)	282.2	11.7	16.3	-4.8(-5.0,-4.6)
Mali	Overall nutritional deficiencies	1483830.1	6770.1	4323.2	-1.1(-1.7,-0.6)	893145.8	8098.4	5442.1	-0.5(-1.0,0.1)	590684.3	5424.7	3235.5	-2.0(-2.5,-1.4)
	Protein-energy malnutrition	1164200.6	5311.7	3205.7	-1.5(-2.2,-0.8)	725699.3	6580.1	4163.9	-0.7(-1.4,0.0)	438501.3	4027.1	2276.9	-2.6(-3.3,-2.0)
	Iodine deficiency	3037.0	13.9	17.5	-2.4(-2.6,-2.3)	1879.0	17.0	21.1	-2.3(-2.5,-2.2)	1158.0	10.6	13.7	-2.6(-2.7,-2.4)
	Vitamin A deficiency	26432.8	120.6	73.1	-1.7(-1.8,-1.7)	10822.9	98.1	60.6	-1.4(-1.5,-1.3)	15609.9	143.4	85.2	-1.9(-2.0,-1.8)
	Dietary iron deficiency	272652.9	1244.0	929.7	0.7(0.4,0.9)	142892.3	1295.6	1039.2	0.5(0.3,0.8)	129760.6	1191.7	820.6	0.9(0.7,1.2)
	Other nutritional deficiencies	17506.9	79.9	97.3	-1.0(-1.6,-0.4)	11852.3	107.5	157.2	-0.1(-0.8,0.6)	5654.5	51.9	39.0	-3.0(-3.4,-2.5)
Mauritania	Overall nutritional deficiencies	42547.7	1059.9	996.4	-4.1(-4.5,-3.7)	21930.6	1074.7	1058.0	-3.7(-4.1,-3.4)	20617.1	1044.7	927.7	-4.5(-5.0,-4.0)
	Protein-energy malnutrition	12104.5	301.5	343.9	-7.2(-8.0,-6.3)	6038.8	295.9	352.5	-6.6(-7.3,-5.9)	6065.7	307.3	335.7	-7.7(-8.7,-6.7)
	Iodine deficiency	1620.1	40.4	45.7	-1.8(-2.0,-1.5)	1005.5	49.3	55.6	-1.7(-1.9,-1.5)	614.6	31.1	35.1	-1.8(-2.1,-1.6)
	Vitamin A deficiency	1394.1	34.7	26.1	-3.2(-3.2,-3.1)	578.5	28.3	22.0	-2.5(-2.6,-2.4)	815.6	41.3	30.1	-3.5(-3.6,-3.5)
	Dietary iron deficiency	26627.8	663.3	553.9	-0.6(-0.7,-0.5)	13818.6	677.2	594.7	-0.7(-0.8,-0.7)	12809.2	649.0	506.8	-0.5(-0.6,-0.4)
	Other nutritional deficiencies	801.2	20.0	26.8	-5.4(-6.3,-4.5)	489.2	24.0	33.2	-5.5(-6.3,-4.7)	312.0	15.8	19.9	-5.2(-6.2,-4.2)
Niger	Overall nutritional deficiencies	461511.1	1981.1	1238.6	-4.2(-4.5,-4.0)	253527.9	2161.4	1395.8	-4.0(-4.3,-3.8)	207983.2	1798.3	1079.9	-4.5(-4.7,-4.2)
	Protein-energy malnutrition	218767.5	939.1	501.6	-6.4(-6.7,-6.0)	116939.7	997.0	540.2	-6.2(-6.6,-5.8)	101827.8	880.4	464.5	-6.5(-6.9,-6.1)
	Iodine deficiency	8752.4	37.6	49.4	-1.0(-1.2,-0.8)	4868.4	41.5	54.5	-0.9(-1.1,-0.7)	3884.0	33.6	43.9	-1.1(-1.4,-0.9)
	Vitamin A deficiency	37771.3	162.1	88.7	-1.1(-1.1,-1.0)	16269.4	138.7	77.7	-0.9(-1.0,-0.8)	21501.8	185.9	99.5	-1.2(-1.2,-1.1)
	Dietary iron deficiency	188977.2	811.2	575.3	-0.8(-0.9,-0.6)	111048.8	946.7	694.2	-0.8(-1.0,-0.7)	77928.3	673.8	454.3	-0.7(-0.8,-0.5)
	Other nutritional deficiencies	7242.8	31.1	23.5	-4.2(-4.4,-4.0)	4401.5	37.5	29.3	-4.0(-4.2,-3.9)	2841.2	24.6	17.7	-4.5(-4.6,-4.3)
Nigeria	Overall nutritional deficiencies	2495048.4	1161.4	886.3	-1.4(-1.6,-1.3)	1286684.3	1153.4	919.7	-1.2(-1.4,-1.1)	1208364.0	1170.1	846.3	-1.7(-1.8,-1.5)
	Protein-energy malnutrition	543266.3	252.9	179.1	-4.2(-4.5,-3.8)	265713.2	238.2	173.1	-4.2(-4.5,-3.9)	277553.1	268.8	186.1	-4.1(-4.4,-3.7)

	Iodine deficiency	22048.0	10.3	12.5	-3.1(-3.6,-2.6)	16074.8	14.4	17.2	-3.4(-4.0,-2.8)	5973.2	5.8	7.1	-3.1(-3.6,-2.5)
	Vitamin A deficiency	52694.2	24.5	17.5	-3.4(-3.7,-3.0)	26939.9	24.1	17.6	-2.6(-3.0,-2.1)	25754.3	24.9	17.3	-4.1(-4.4,-3.7)
	Dietary iron deficiency	1825622.1	849.8	656.2	0.2(0.1,0.3)	952659.5	854.0	691.6	0.6(0.4,0.7)	872962.6	845.4	613.9	-0.2(-0.2,-0.1)
	Other nutritional deficiencies	51417.8	23.9	21.0	-1.9(-2.0,-1.8)	25296.9	22.7	20.2	-2.1(-2.2,-2.0)	26120.8	25.3	21.9	-1.8(-2.0,-1.6)
Sao Tome and Principe	Overall nutritional deficiencies	1496.2	728.5	706.3	-3.6(-4.3,-3.0)	719.6	702.9	703.9	-3.5(-4.1,-2.8)	776.6	753.8	709.2	-3.8(-4.5,-3.1)
	Protein-energy malnutrition	432.1	210.4	226.0	-6.5(-7.3,-5.8)	212.5	207.6	223.2	-6.2(-6.9,-5.6)	219.6	213.2	228.5	-6.8(-7.6,-6.0)
	Iodine deficiency	29.6	14.4	15.4	-1.1(-1.3,-0.8)	18.0	17.6	18.7	-1.0(-1.2,-0.7)	11.6	11.3	12.1	-1.1(-1.4,-0.9)
	Vitamin A deficiency	74.8	36.4	30.0	-4.0(-4.1,-3.8)	22.3	21.8	18.6	-3.8(-4.0,-3.5)	52.4	50.9	41.3	-4.0(-4.2,-3.9)
	Dietary iron deficiency	925.3	450.5	414.5	0.1(-0.7,0.8)	445.5	435.2	418.0	-0.1(-0.8,0.7)	479.8	465.8	411.9	0.2(-0.5,0.9)
	Other nutritional deficiencies	34.3	16.7	20.4	-3.8(-4.2,-3.4)	21.2	20.8	25.3	-3.9(-4.2,-3.6)	13.1	12.7	15.4	-3.6(-4.2,-2.9)
Senegal	Overall nutritional deficiencies	174405.1	1152.4	992.2	-2.5(-2.6,-2.4)	87922.7	1165.0	1052.1	-2.2(-2.3,-2.1)	86482.4	1139.9	923.6	-2.9(-3.0,-2.8)
	Protein-energy malnutrition	28643.6	189.3	163.6	-6.1(-6.4,-5.8)	14556.2	192.9	164.2	-5.8(-6.1,-5.5)	14087.4	185.7	163.7	-6.3(-6.6,-6.0)
	Iodine deficiency	3734.2	24.7	27.8	-1.9(-2.1,-1.7)	2116.1	28.0	31.3	-1.8(-2.0,-1.6)	1618.1	21.3	24.1	-2.0(-2.2,-1.7)
	Vitamin A deficiency	6125.2	40.5	29.9	-4.2(-4.5,-3.8)	2012.5	26.7	20.5	-3.4(-3.6,-3.2)	4112.7	54.2	38.7	-4.6(-5.1,-4.2)
	Dietary iron deficiency	133757.4	883.8	756.3	-0.6(-0.7,-0.4)	68010.7	901.2	819.1	-0.3(-0.4,-0.2)	65746.7	866.6	685.2	-0.8(-1.0,-0.7)
	Other nutritional deficiencies	2144.7	14.2	14.6	-3.0(-3.2,-2.8)	1227.1	16.3	17.0	-3.2(-3.4,-2.9)	917.6	12.1	12.1	-2.7(-3.0,-2.4)
Sierra Leone	Overall nutritional deficiencies	163783.5	1976.9	1519.1	-3.3(-3.6,-3.1)	78832.0	1884.7	1487.7	-3.2(-3.3,-3.0)	84951.4	2070.9	1551.8	-3.5(-3.8,-3.2)
	Protein-energy malnutrition	94215.8	1137.2	819.0	-4.7(-5.1,-4.3)	45443.0	1086.5	796.9	-4.5(-4.8,-4.3)	48772.8	1189.0	839.9	-4.8(-5.3,-4.3)
	Iodine deficiency	1859.4	22.4	25.4	-1.2(-1.8,-0.6)	1110.3	26.5	30.0	-1.2(-1.7,-0.6)	749.1	18.3	20.8	-1.2(-1.8,-0.6)
	Vitamin A deficiency	6723.2	81.2	57.7	-1.9(-2.1,-1.8)	2694.2	64.4	46.5	-1.4(-1.6,-1.2)	4029.0	98.2	69.0	-2.2(-2.4,-2.1)
	Dietary iron deficiency	58173.5	702.2	585.1	0.2(0.2,0.3)	27775.5	664.1	570.5	0.3(0.3,0.4)	30398.0	741.0	602.0	0.1(0.1,0.2)
	Other nutritional deficiencies	2811.5	33.9	31.8	-2.6(-2.8,-2.4)	1809.1	43.3	43.8	-2.0(-2.2,-1.7)	1002.5	24.4	20.1	-3.6(-3.8,-3.4)
Togo	Overall nutritional deficiencies	75461.1	952.6	849.8	-2.9(-3.2,-2.7)	41317.4	1021.8	938.1	-2.7(-3.0,-2.4)	34143.8	880.5	757.9	-3.2(-3.5,-3.0)
	Protein-energy malnutrition	14656.7	185.0	190.3	-6.6(-6.9,-6.3)	7664.6	189.5	189.9	-6.6(-6.9,-6.3)	6992.1	180.3	191.3	-6.6(-6.9,-6.2)
	Iodine deficiency	2459.8	31.1	34.5	-2.0(-2.3,-1.8)	1479.9	36.6	39.8	-2.0(-2.2,-1.7)	979.9	25.3	28.5	-2.1(-2.4,-1.9)
	Vitamin A deficiency	4903.4	61.9	45.4	-2.7(-2.8,-2.6)	1835.3	45.4	34.9	-2.0(-2.1,-1.8)	3068.0	79.1	55.5	-3.1(-3.2,-3.0)
	Dietary iron deficiency	52189.5	658.8	559.6	0.4(0.1,0.7)	29589.3	731.7	650.3	0.4(0.2,0.7)	22600.2	582.8	466.0	0.4(0.1,0.6)
	Other nutritional deficiencies	1251.8	15.8	20.1	-3.8(-4.0,-3.7)	748.2	18.5	23.2	-4.3(-4.5,-4.2)	503.6	13.0	16.7	-2.9(-3.2,-2.6)
American Samoa	Overall nutritional deficiencies	240.0	432.3	469.7	-1.3(-1.4,-1.2)	157.9	572.8	616.5	-1.2(-1.3,-1.0)	82.1	293.7	309.7	-1.7(-1.8,-1.5)
	Protein-energy malnutrition	60.6	109.3	132.2	-2.5(-2.7,-2.3)	38.9	141.0	167.1	-2.2(-2.5,-1.8)	21.8	78.0	90.1	-3.1(-3.5,-2.7)
	Iodine deficiency	0.2	0.4	0.5	-1.2(-1.3,-1.1)	0.1	0.5	0.5	-1.0(-1.1,-1.0)	0.1	0.3	0.4	-1.5(-1.6,-1.4)
	Vitamin A deficiency	2.6	4.7	4.8	-1.6(-1.9,-1.4)	0.9	3.4	3.5	-1.7(-2.0,-1.5)	1.7	6.1	6.0	-1.6(-1.8,-1.4)
	Dietary iron deficiency	170.3	306.8	320.3	-0.6(-0.7,-0.5)	114.5	415.3	432.0	-0.7(-0.8,-0.6)	55.8	199.7	202.7	-0.7(-0.8,-0.5)

	Other nutritional deficiencies	6.2	11.1	11.9	-1.8(-2.3,-1.4)	3.5	12.7	13.3	-1.4(-1.9,-0.9)	2.7	9.5	10.6	-2.3(-2.8,-1.9)
Tuvalu	Overall nutritional deficiencies	67.5	572.2	608.1	-2.8(-3.0,-2.6)	42.5	749.7	786.9	-2.5(-2.7,-2.3)	25.0	407.9	430.1	-3.1(-3.4,-2.8)
	Protein-energy malnutrition	12.7	107.5	131.2	-6.0(-6.4,-5.7)	7.5	132.4	158.4	-5.8(-6.1,-5.5)	5.2	84.4	100.5	-6.4(-6.9,-6.0)
	Iodine deficiency	0.2	1.7	1.7	-4.1(-4.2,-3.9)	0.1	2.1	2.1	-3.9(-4.1,-3.7)	0.1	1.3	1.3	-4.1(-4.3,-4.0)
	Vitamin A deficiency	2.2	18.8	19.1	-1.8(-2.0,-1.6)	0.8	14.6	15.0	-1.9(-2.1,-1.7)	1.4	22.6	22.9	-1.8(-2.0,-1.6)
	Dietary iron deficiency	51.3	435.0	446.1	-1.0(-1.0,-0.9)	33.5	590.1	600.5	-0.8(-0.9,-0.8)	17.8	291.4	296.7	-0.9(-0.9,-0.8)
	Other nutritional deficiencies	1.1	9.3	9.9	-1.1(-1.6,-0.6)	0.6	10.5	10.9	-0.8(-1.3,-0.3)	0.5	8.1	8.8	-1.5(-2.0,-1.0)
South Sudan	Overall nutritional deficiencies	239317.4	2578.0	1957.4	-5.0(-5.3,-4.8)	121643.8	2631.1	2072.8	-4.7(-4.8,-4.5)	117673.6	2525.4	1853.6	-5.4(-5.7,-5.1)
	Protein-energy malnutrition	167525.8	1804.7	1273.3	-6.0(-6.3,-5.7)	86082.2	1861.9	1330.2	-5.6(-5.8,-5.4)	81443.6	1747.8	1219.5	-6.4(-6.7,-6.0)
	Iodine deficiency	1901.0	20.5	24.5	0.2(0.1,0.4)	1108.8	24.0	28.1	0.2(0.0,0.3)	792.2	17.0	20.7	0.2(0.1,0.3)
	Vitamin A deficiency	4736.1	51.0	33.1	-2.4(-2.5,-2.2)	2211.1	47.8	32.2	-1.9(-2.0,-1.9)	2525.0	54.2	33.9	-2.7(-2.9,-2.5)
	Dietary iron deficiency	62812.5	676.6	594.7	-0.4(-0.5,-0.4)	31001.0	670.5	645.3	-0.4(-0.5,-0.3)	31811.4	682.7	552.8	-0.5(-0.5,-0.4)
	Other nutritional deficiencies	2341.9	25.2	31.8	-7.0(-7.4,-6.7)	1240.6	26.8	37.0	-6.0(-6.3,-5.8)	1101.3	23.6	26.7	-8.0(-8.4,-7.5)
Sudan	Overall nutritional deficiencies	280934.2	688.4	605.2	-3.5(-3.6,-3.5)	144786.0	719.4	670.3	-3.4(-3.4,-3.3)	136148.3	658.3	543.3	-3.7(-3.9,-3.6)
	Protein-energy malnutrition	92403.5	226.4	187.2	-5.9(-6.1,-5.7)	41654.5	207.0	172.8	-6.2(-6.4,-6.1)	50749.0	245.4	200.8	-5.7(-5.9,-5.4)
	Iodine deficiency	16593.4	40.7	42.3	-2.7(-3.1,-2.4)	8848.9	44.0	45.4	-2.8(-3.2,-2.4)	7744.5	37.4	39.2	-2.6(-3.0,-2.3)
	Vitamin A deficiency	9776.3	24.0	18.6	-3.4(-3.7,-3.1)	4191.0	20.8	16.4	-3.0(-3.3,-2.7)	5585.3	27.0	20.6	-3.7(-4.0,-3.4)
	Dietary iron deficiency	154417.4	378.4	337.7	-1.1(-1.2,-1.0)	86716.8	430.8	418.7	-1.0(-1.1,-1.0)	67700.6	327.4	260.8	-1.2(-1.2,-1.1)
	Other nutritional deficiencies	7743.7	19.0	19.5	-2.4(-2.6,-2.2)	3374.9	16.8	17.0	-1.8(-2.1,-1.6)	4368.8	21.1	21.8	-2.8(-3.0,-2.7)

SECTION 2 EFIGURES

Figure S1. The trend of age-sex specific incidence rates and DALYs rates for high-risk subcategories in low-and middle-income countries (LMICs) from 1990 to 2019.

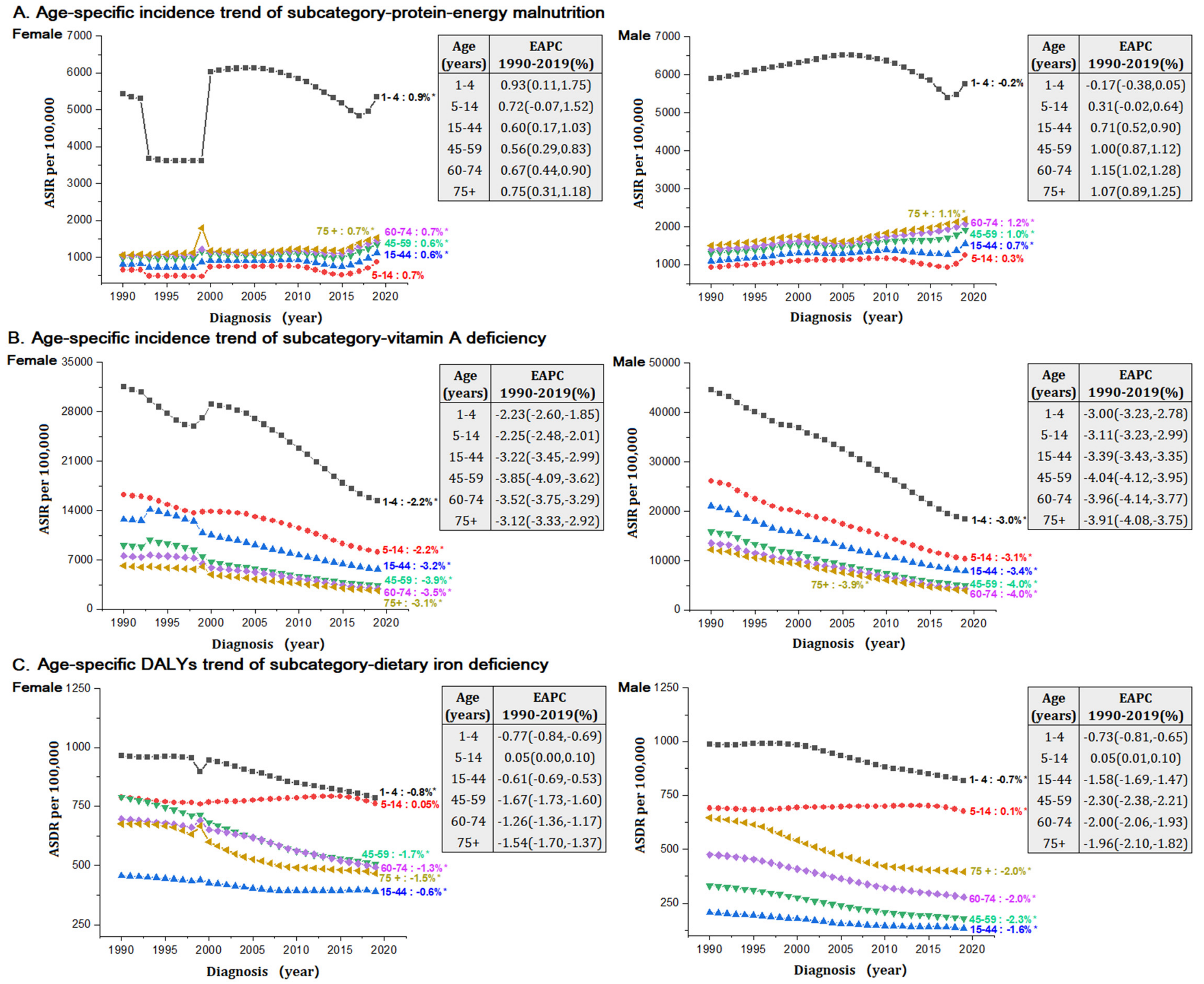

A. Female

Age (years)	EAPC 1990-2019(%)
1-4	0.93(0.11,1.75)
5-14	0.72(-0.07,1.52)
15-44	0.60(0.17,1.03)
45-59	0.56(0.29,0.83)
60-74	0.67(0.44,0.90)
75+	0.75(0.31,1.18)

A. Male

Age (years)	EAPC 1990-2019(%)
1-4	-0.17(-0.38,0.05)
5-14	0.31(-0.02,0.64)
15-44	0.71(0.52,0.90)
45-59	1.00(0.87,1.12)
60-74	1.15(1.02,1.28)
75+	1.07(0.89,1.25)

B. Female

Age (years)	EAPC 1990-2019(%)
1-4	-2.23(-2.60,-1.85)
5-14	-2.25(-2.48,-2.01)
15-44	-3.22(-3.45,-2.99)
45-59	-3.85(-4.09,-3.62)
60-74	-3.52(-3.75,-3.29)
75+	-3.12(-3.33,-2.92)

B. Male

Age (years)	EAPC 1990-2019(%)
1-4	-3.00(-3.23,-2.78)
5-14	-3.11(-3.23,-2.99)
15-44	-3.39(-3.43,-3.35)
45-59	-4.04(-4.12,-3.95)
60-74	-3.96(-4.14,-3.77)
75+	-3.91(-4.08,-3.75)

C. Female

Age (years)	EAPC 1990-2019(%)
1-4	-0.77(-0.84,-0.69)
5-14	0.05(0.00,0.10)
15-44	-0.61(-0.69,-0.53)
45-59	-1.67(-1.73,-1.60)
60-74	-1.26(-1.36,-1.17)
75+	-1.54(-1.70,-1.37)

C. Male

Age (years)	EAPC 1990-2019(%)
1-4	-0.73(-0.81,-0.65)
5-14	0.05(0.01,0.10)
15-44	-1.58(-1.69,-1.47)
45-59	-2.30(-2.38,-2.21)
60-74	-2.00(-2.06,-1.93)
75+	-1.96(-2.10,-1.82)

A. Age-standardized incidence rate (ASIR) for protein-energy malnutrition; B. Age-standardized incidence rate (ASIR) for vitamin A deficiency; C. Age-standardized DALYs rate (ASDR) for dietary iron deficiency.

DALYs, disability-adjusted life years; EAPC, estimated annual percent change. Note: (*) Indicates statistically significant trend (*P*< .05).

Figure S2. Age-standardized incidence rate (ASIR) in 2019 and its estimated annual percentage change (EAPC) during 1990-2019 due to protein-energy malnutrition for all ages stratified by sex at national level in low-and middle-income countries (LMICs).

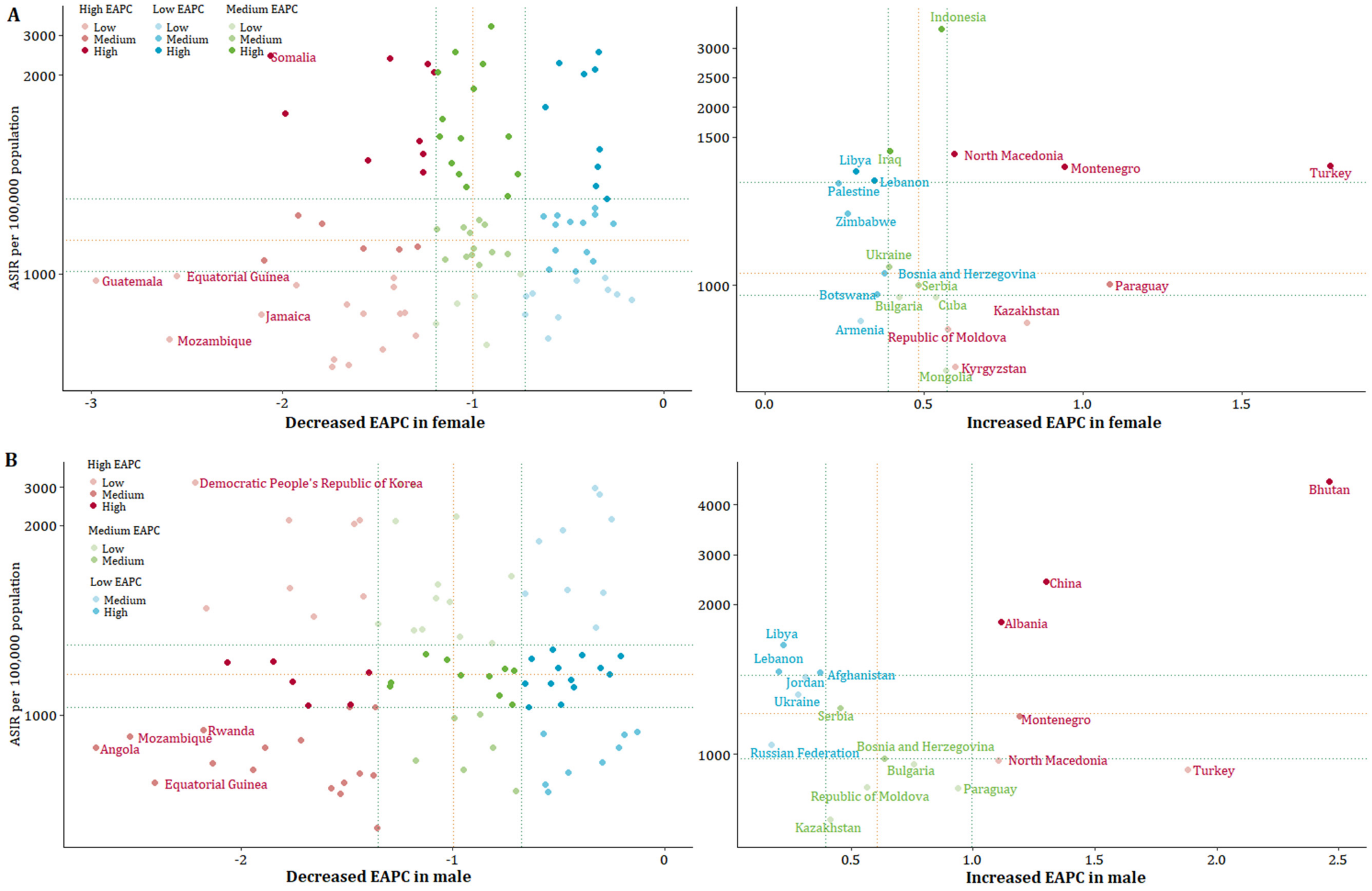

A. Female; B. Male. DALYs, disability-adjusted life years.

Note: Dots represent countries, colour coded according to quadrants defined by lower and upper terciles (33 and 66 percentiles) of the distribution of countries according to the ASIR per 100000 population and the estimated

annual percentage change of the ASIR per 100000 population in 1990–2019.

Figure S3. Age-standardized incidence rate (ASIR) and Age-standardized DALYs rate (ASDR) in 2019 and their estimated annual percentage change (EAPC) during 1990-2019 due to iodine deficiency stratified by sex at national level in low-and middle-income countries (LMICs).

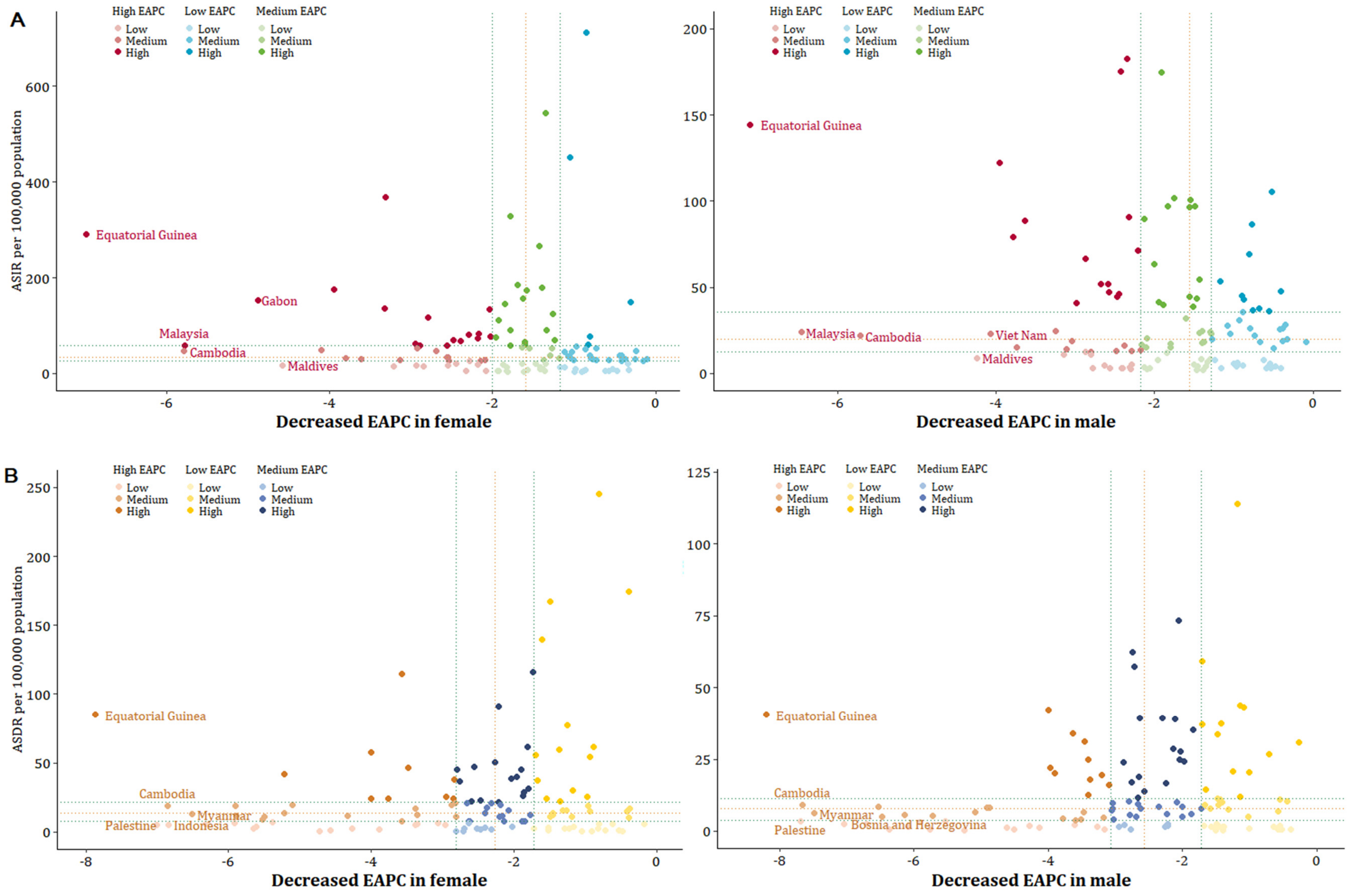

A. Age-standardized incidence rate (ASIR); B. Age-standardized DALYs rate (ASDR). DALYs, disability-adjusted life years.

Note: Dots represent countries, colour coded according to quadrants defined by lower and upper terciles (33 and 66 percentiles) of the distribution of countries according to the ASIR per 100000 population and the estimated

annual percentage change of the ASIR and ASDR per 100000 population in 1990–2019.

Figure S4. Age-standardized incidence rate (ASIR) and Age-standardized DALYs rate (ASDR) in 2019 and their estimated annual percentage change (EAPC) during 1990-2019 due to vitamin A deficiency stratified by sex at national level in low-and middle-income countries (LMICs).

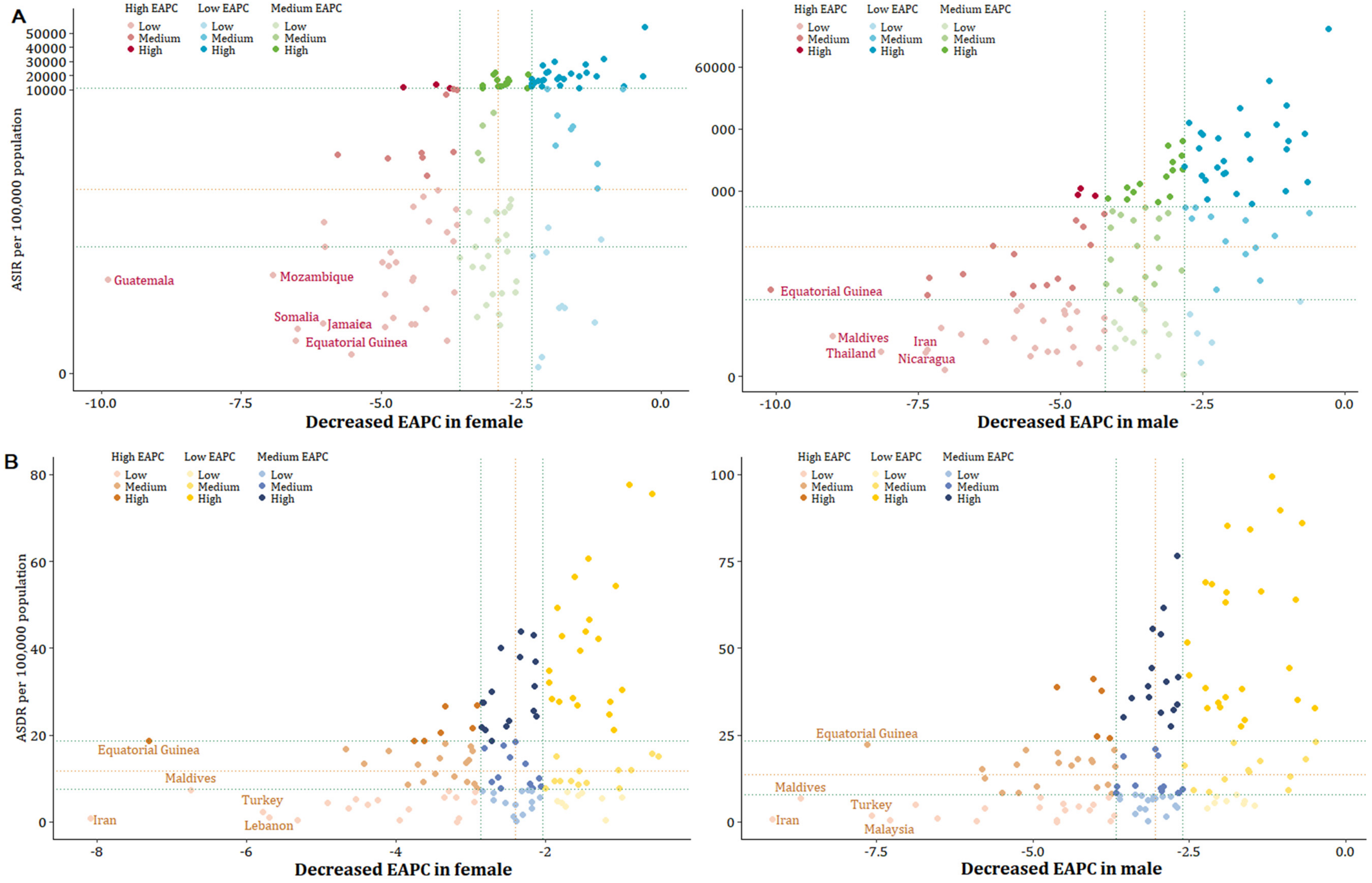

A. Age-standardized incidence rate (ASIR); B. Age-standardized DALYs rate (ASDR). DALYs, disability-adjusted life years.

Note: Dots represent countries, colour coded according to quadrants defined by lower and upper terciles (33 and 66 percentiles) of the distribution of countries according to the ASIR per 100000 population and the estimated annual percentage change of the ASIR and ASDR per 100000 population in 1990–2019.

Figure S5. Age-standardized DALYs rate (ASDR) in 2019 and its estimated annual percentage change (EAPC) in 1990-2019 due to dietary iron deficiency stratified by sex at national level in low-and middle-income countries (LMICs).

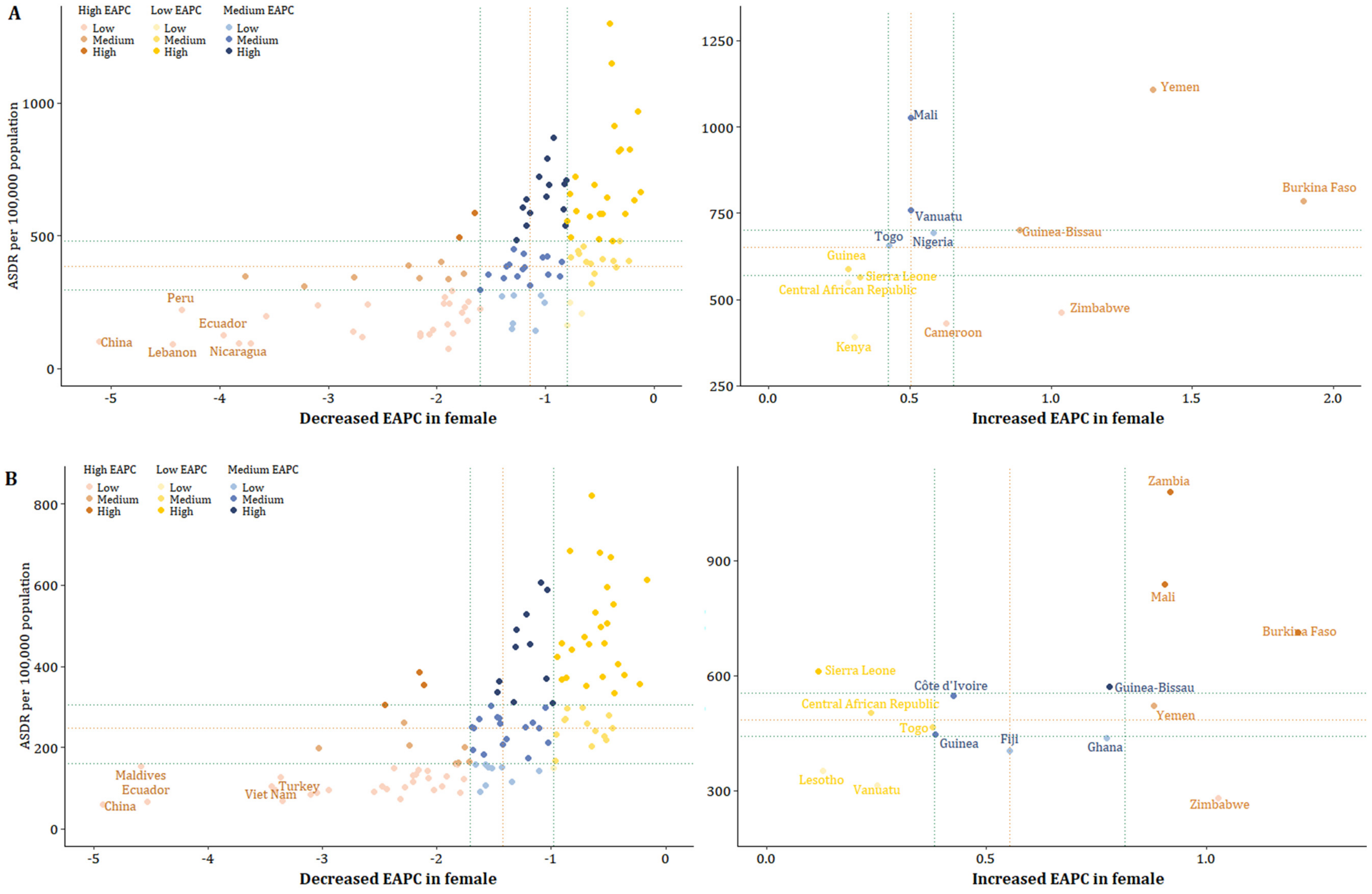

A. Female; B. Male. DALYs, disability-adjusted life years.

Note: Dots represent countries, colour coded according to quadrants defined by lower and upper terciles (33 and 66 percentiles) of the distribution of countries according to the ASIR per 100000 population and the estimated

annual percentage change of the ASDR per 100000 population in 1990–2019.

Figure S6. The correlation between sex-specific age-standardized incidence rates (ASIR) and age-standardized DALYs rates (ASDR) for nutritional deficiencies (NDs) with Gender development index (GDI) in low-and middle-income countries (LMICs) in 2019.

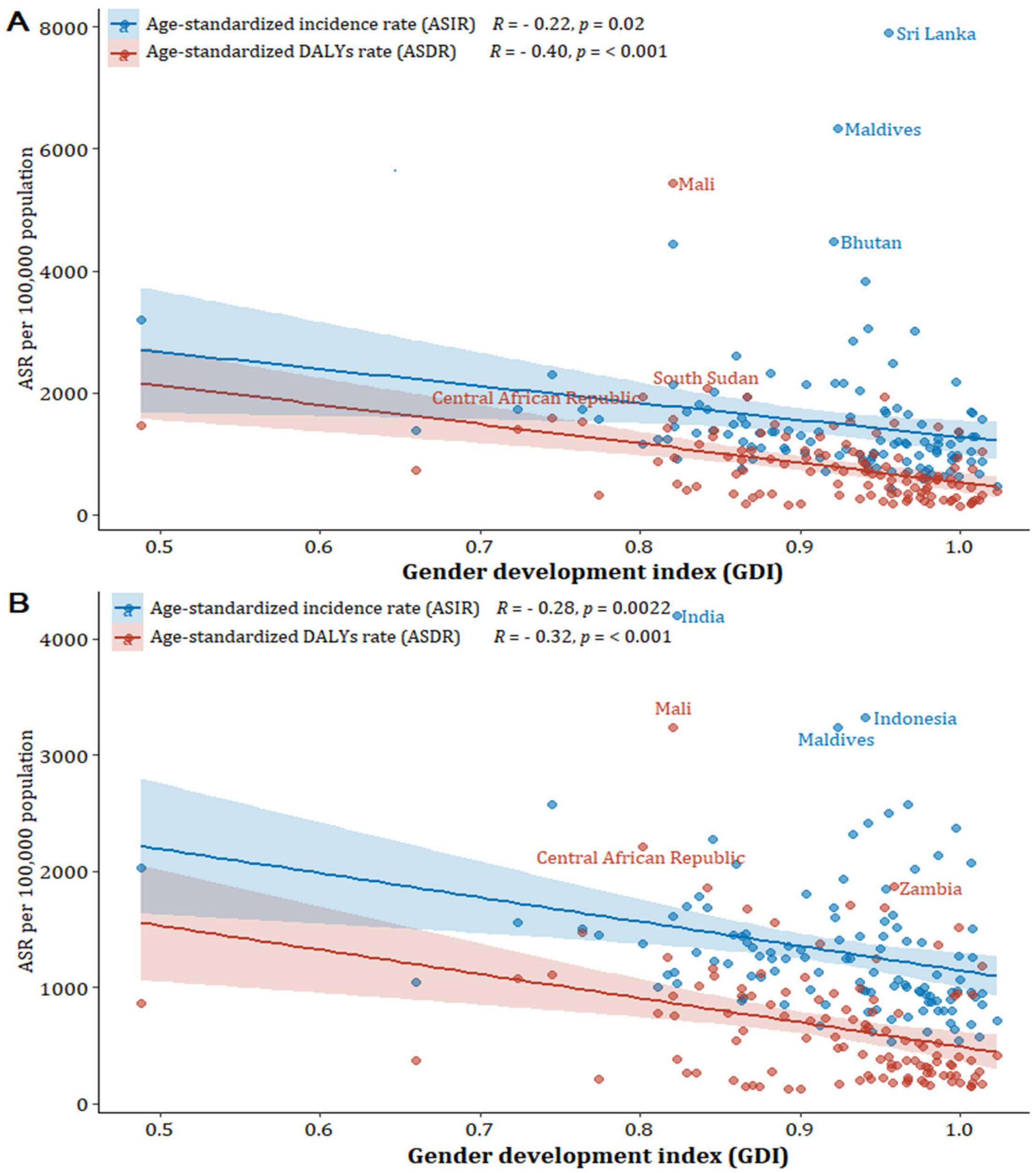

A. Female; B. Male.

Figure S7. The correlation between sex-specific age-standardized incidence rates (ASIR) and age-standardized DALYs rates (ASDR) for nutritional deficiencies with Gender development index (GDI) in 2019.

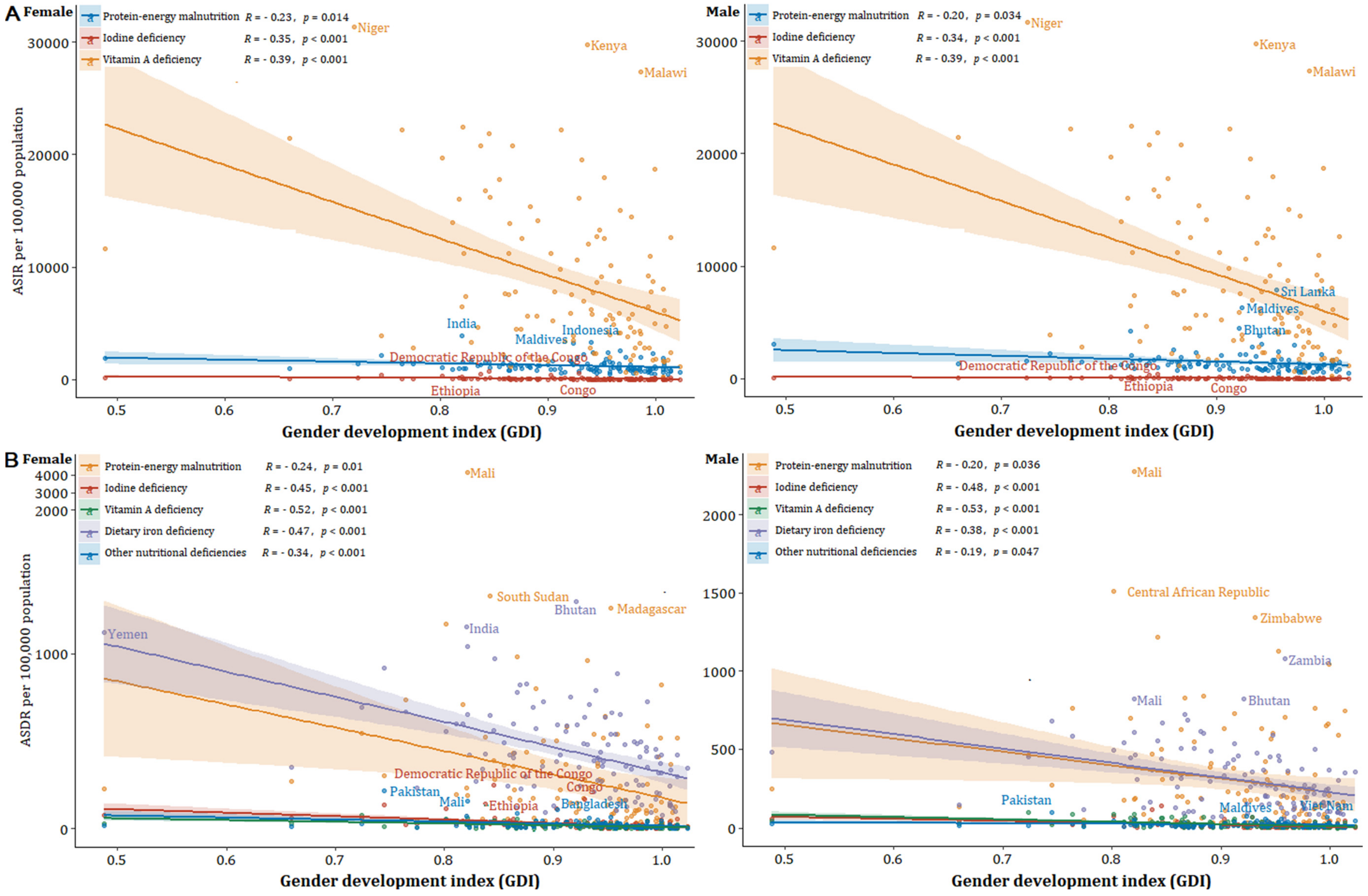

A. Age-standardized incidence rates (ASIR); B. Age-standardized DALYs rates (ASDR). DALYs, disability-adjusted life years.
